# Ru-catalyzed activation of free phenols in a one-step Suzuki–Miyaura cross-coupling under mechanochemical conditions†

**DOI:** 10.1039/d4sc01704h

**Published:** 2024-08-15

**Authors:** Satenik Mkrtchyan, Michał Jakubczyk, Sehrish Sarfaraz, Khurshid Ayub, Viktor O. Iaroshenko

**Affiliations:** a Department of Chemistry, Faculty of Natural Sciences, Matej Bel University Tajovského 40 97401 Banska Bystrica Slovakia iva108@gmail.com Iaroshenko.V@gust.edu.kw viktor.iaroshenko@umb.sk; b University Centre for Research & Development, Chandigarh University Mohali Punjab 140413 India; c Institute of Inorganic Chemistry, Czech Academy of Sciences Husinec-Řež č.p. 1001 250 68 Husinec-Řež Czech Republic; d Laboratory of Molecular Assays and Imaging, Institute of Bioorganic Chemistry, Polish Academy of Sciences Noskowskiego 12/14 61-704 Poznań Poland; e Department of Chemistry, COMSATS University, Abbottabad Campus Abbottabad KPK 22060 Pakistan; f Department of Fiber and Polymer Technology, Division of Wood Chemistry and Pulp Technology, School of Chemistry, Biotechnology and Health, KTH Royal Institute of Technology Teknikringen 56-58 SE-100 44 Stockholm Sweden; g Functional Materials Group, Gulf University for Science and Technology Mubarak Al-Abdullah 32093 Kuwait; h Centre of Research Impact and Outcome, Chitkara University Institute of Engineering and Technology, Chitkara University Rajpura-140401 Punjab India

## Abstract

Activation of phenols by a Ru-catalyst allows for the resulting η^5^-phenoxo complex to selectively react with a variety of nucleophiles under mechanochemical conditions. Conversion of phenolic hydroxy groups without derivatization is important for late-stage modifications of pharmaceuticals and in the context of lignin-material processing. We present a one-step, Ru-catalyzed cross-coupling of phenols with boronic acids, aryl trialkoxysilanes and potassium benzoyltrifluoroborates under mechano-chemical conditions. The protocol accepts a wide scope of starting materials and allows for gram-scale synthesis in excellent yields. The developed approach constitutes a very interesting and waste-limiting alternative to the known methods.

## Abstract 2

Activation of phenols by a Ru-catalyst allows for the resulting η^5^-phenoxo complex to selectively react with a variety of nucleophiles under mechanochemical conditions.

## Graphical abstract

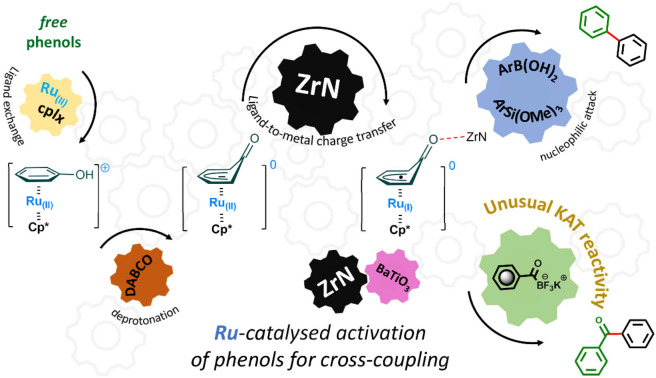

Concise characteristics, selectivity, and simplicity of the Suzuki–Miyaura cross-coupling made it one of the most important methods in organic synthesis for medicinal chemistry^1,2^ and other fields,^3^ as indicated by the 2010 Chemistry Nobel Prize. Continuous development in this field is also manifested by the systematic broadening of the scope of starting materials, allowing for more flexible and convenient synthetic route design.^4^ One attractive and relatively accessible alternative to aryl halides are phenol-based substrates, complementing the general scope of starting materials as they are derived from precursors of a different chemical origin. As an abundant motive in naturally occurring structures, phenols have been considered in cross-coupling reactions as electrophiles with a variety of nucleophilic partners.^5–10^ The most commonly used derivatives are triflates and more stable to hydrolysis, sulphur-containing reagents: mesylates, tosylates and sulphamates. Esters, carbamates, carbonates, ethers and silyl ethers present a very interesting set of substrates, easily accessible, more stable than triflates and widely present as functional groups.

The great majority of all activated phenolic starting materials enter the TM-catalyzed protocols according to a classical oxidative-addition mechanism. Methods utilizing a *free* phenol have not been introduced to date. Recently, a one-pot, three-component approach was presented, in which an *in situ* generated dichloroimidazolidinedione derivative of phenol enters the reaction in the presence of Pd-MOF heterogenic catalyst, according to the same mechanism.^11^ In another recent example, the SuFEx approach is utilized to convert phenols into tosylates *in situ* and react them in a Ni-catalyzed protocol in the presence of water under N_2_ atmosphere.^12^ On the other hand, ruthenium,^13^ iridium^14,15^ and rhodium^16^ cyclopentadienyl complexes easily exchange a more electron deficient arene to electron-rich *free* phenol (Scheme 1). As shown by Shi *et al.* in their recent paper on Rh-catalyzed amination,^16^ this can be exploited to activate the phenolic C–O bond into a C=O bond what allows for a nucleophilic attack on the resulting transient η^5^-phenoxo complex.

**Scheme 1:**
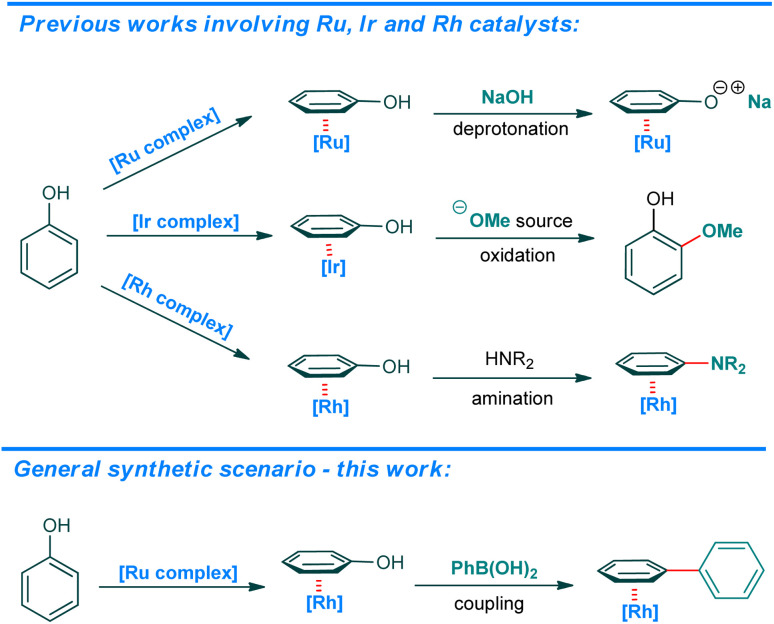
General pathways for the formation and reactivity of η^6^-phenol complexes with ruthenium, iridium, and rhodium and scenario for the title process using ruthenium catalyst under ball-milling conditions.

Encouraged by our previous experiences with catalytical transformations under ball-milling conditions^17–22^ we considered the possibility of phenolic C–O bond activation for coupling in the solid-phase without prior derivatization, according to a π-coordination/activation mechanism.^23–30^ Studies on solid-phase cross-couplings have been ongoing over the last two decades.^31^ Peters *et al.*, in their works on Suzuki reaction, obtained high yields of the expected products.^32^ Furthermore, a study on cross-coupling using *in situ* generated solid base (KF–Al_2_O_3_) by Ondruschka *et al.* indicated the superiority of ball-milling over microwave irradiation as two modes of energy introduction, considering the yield against the power consumption.^33^ In the recent work by Szostak *et al.* a chemoselective Suzuki–Miyaura protocol was presented to obtain ketones from acyl chlorides under ball-milling conditions.^34^ As a follow-up to the work of Mack *et al.* on the solvent-free Sonogashira coupling,^35^ Stolle *et al.* introduced a Cu-free method resulting in high yields in shorter reaction times.^36^ In recent works, Browne *et al.* described a solid-phase, Ni-catalyzed method^37,38^ and Ito and Kubota *et al.* published a study on solid-state cross-coupling of insoluble aryl halides^39^ and very comprehensive papers on mechanochemical C–N cross-coupling.^40,41^

For our protocol, we selected the Cp*Ru(PhCl)BF_4_ catalyst, following the recently introduced direct deoxyfluorination method^42,43^ that utilized the Cp*Ru(Napht)BF_4_ catalyst. Both, the naphthalene as well as chlorobenzene undergo arene exchange by substituted benzene derivatives under photochemical and thermal conditions, including phenols.^44–46^ Herein we present the first, truly direct, one-step, Ru-catalyzed Suzuki–Miyaura cross-coupling of *free* phenols with arylboronic acids (according to the scenario presented in Scheme 2), further extending the methodology to include aryl trialkoxysilanes as nucleophilic partners, realizing a Hiyama-type cross-coupling. We also present the unusual reactivity of potassium benzoyltrifluoroborates under the conditions of our solid-phase protocol, yielding benzophenones.

**Scheme 2:**
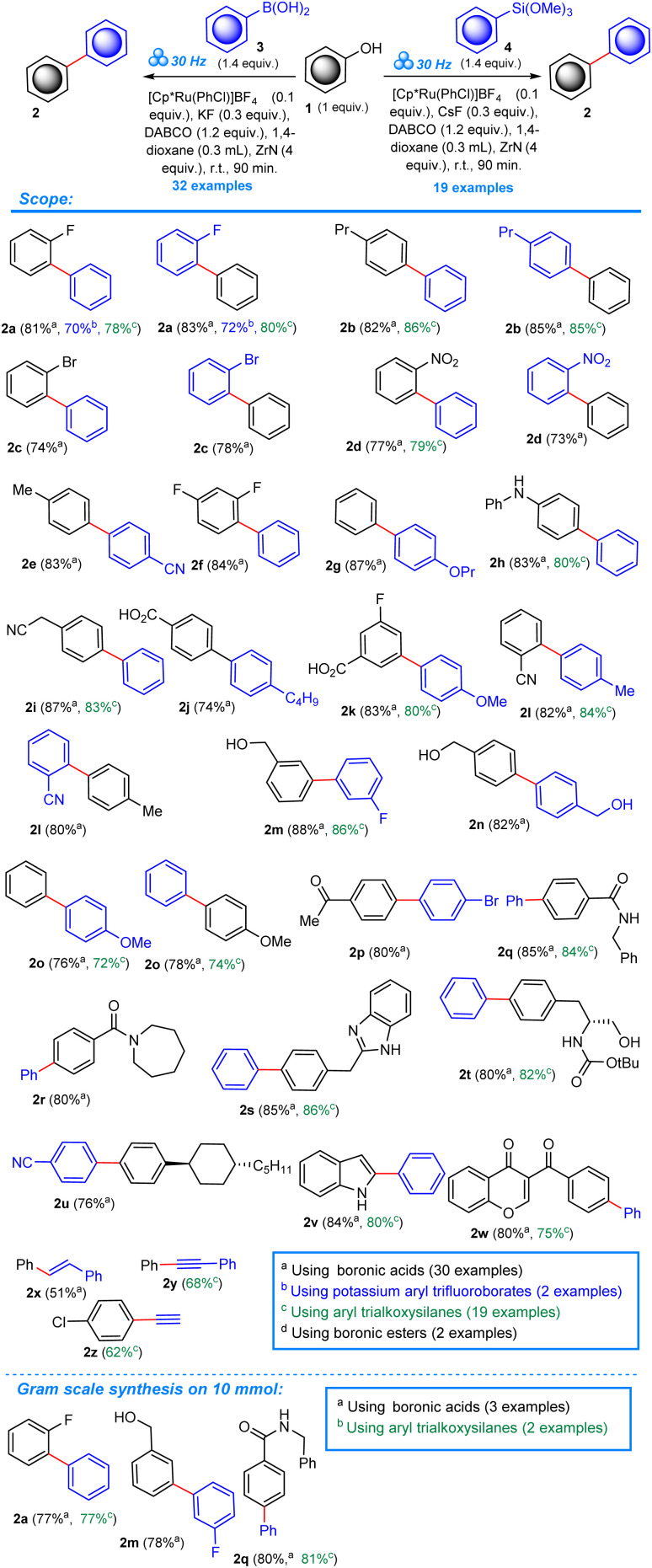
Reaction scope and the results of the gram-scale experiments.

Our initial experiments included the use of Cp*Ru(Napht)BF_4_ catalyst. The trial run from model starting materials (*ortho*-fluorophenol 1b and phenylboronic acid 3a) in the presence of CsF (0.3 equiv.), DABCO (1.2 equiv.) and ZrN (4 equiv.), ground at 30 Hz at room temperature for 90 min gave only 12% yield of the biphenyl product 2a (Table S1 in the ESI†). The crude reaction mixture seemed to be dry and brittle. Therefore, we added a small amount (0.3 mL) of 1,4-dioxane to see if the LAG (liquid-assisted grinding) approach would give a better result. To our great content, the yield jumped to 43% (Table S1,† Entry 2). The changes to the fluoride salt from CsF to KF and NaF resulted in 45% and 28% respectively (Table S1,† Entries 3 and 4), highlighting the importance of a bigger cation. The use of dimeric and tetrameric catalyst forms completely crippled the reaction (Table S1,† Entries 5, 6, and 8), with the exception of the [(*p*-cymene)RuCl_2_]_2_ (yield 22%, Table S1,† Entry 7). The acetonitrile complex (Table S1,† Entry 10) gave only 21% of the product, which shows that MeCN is hardly exchanging for phenol. In contrast, the chlorobenzene complex forms (Table S1,† Entries 11, 12, 13, and 14) exchange for phenol easily, also in comparison to the Napht complexes. Ultimately, the ORCs consisting of 0.1 equiv. [Cp*Ru(PhCl)]BF_4_, 0.3 equiv. KF, 1.2 equiv. DABCO, 0.3 mL 1,4-dioxane and 4 equiv. ZrN, ground at 30 Hz for 90 min at room temp. gave 81% of 2a. Of note, reactions utilizing the same reactants as the above ORCs conducted in solutions of toluene (at reflux), benzene (reflux), 1,4-dioxane (reflux), xylenes (130 °C) and in neat phenol (at 165 °C) gave no product (Table S1,† Entries 15–19). The above ORCs applied to a process involving aryl trialkoxysilanes 4a in place of arylboronic acids 3a resulted in 52% yield of the biphenyl 2a (Table S2,† Entry 1). Much better result was obtained with the CsF salt, 78% (Table S2,† Entry 3). As previously, reactions conducted in solution gave no product (Entries 4–8).

Having established the ORCs for two types of nucleophiles we moved to potassium benzoyltrifluoroborates. Following our recent work on trifluoromethoxyarenes as halide surrogates in cross-couplings under mechanochemical conditions,^19^ we hypothesized that if only the phenolic C–O bond undergoes activation, the reactivity of KATs could be achieved. Extensive optimization using 1a and 5a as model starting materials revealed no reactivity in the presence of ZrN, HfN nor BaBeO_2_ alone (Table S3,† Entries 1–9). However, combination of ZrN and a second *piezo additive* BaBeO_2_, LiNbO_3_, SrTiO_3_, ZnO and BaTiO_3_ gave 17%, 29%, 11%, trace and 90% yield of the 6a benzophenone product (Table S3,† Entries 10–14). Finally, after balancing the equivalent ratios, the ORCs for this process consisting of 0.1 equiv. [Cp*Ru(Napht)]BF_4_, 0.3 equiv. KF, 1.2 equiv. DABCO, 0.3 mL 1,4-dioxane, 2 equiv. ZrN and 2 equiv. BaTiO_3_ ground at 30 Hz for 120 min at room temp. resulted in 88% yield of 6a (Table S3,† Entry 15). In this case as well, reactions conducted in solutions of toluene, benzene, 1,4-dioxane, xylenes and in neat phenol resulted in no product formation (Table S3,† Entries 18–22).

With the ORCs established, we moved to assessing the scope of the reaction (shown in the Scheme 2). In total, a series of 32 phenols with simple, as well as more voluminous substituents, were subjected to the mechanochemical protocol under the ORCs, including arenes bearing useful and reactive functionalities, for example: –Br, –NO_2_, –COOH, –CN, –CH_2_OH, –COOMe. In order to present the robustness and generality of our concept and the effect of a variety of substituents on the reaction outcome we made an effort to double the number of the experiments in some cases, reversing the reacting groups in respect to the coupled scaffolds. In this way, one can choose the more efficient set of coupling handles depending on the presence of desired substituents at both aryl scaffolds. The yields of the obtained biphenyls within the investigated scope oscillate around 80%. The double experiments in cases 2a, 2b, 2c, 2d, 2l and 2o resulted in virtually the same efficiency of biphenyl products regardless of the presence of substituents relative to reacting moieties. A number of more complex phenols including a chromone derivative 1w were included in the study to expand the scope and evidence the synthetic potential of the title transformation. Three unsaturated compounds 2x, 2y, and 2z were also obtained in lower yields (51%, 68%, and 62% respectively) to show the performance of our protocol applied to this type of starting materials. The lower yields at a high substrate conversion could be explained by the low stability of the formed unsaturated products under the reaction conditions.

As a standard practice during our investigations, to document the scale-up applicability of our mechanochemical protocol, we conducted a gram scale synthesis of three biphenyls 2a, 2m, and 2q, obtaining them in only slightly lower yields (few percent).

In our recent work, we showed the unexpected reactivity of KATs in Ni-catalyzed cross-coupling with Ar-OCF_3_ starting materials in solid phase.^19^ The inert nature of KATs in the presence of Pd-catalyst was previously confirmed in solution.^47^ However, there are no attempts using Ru-catalysts documented to date. Under the ORCs of our mechanochemical protocol, potassium benzoyltrifluoroborate 5b in combination with 1a yields benzophenone 6a in 89% as the major product (Scheme 3). In the present work we included three starting materials 5a, 5b, and 5c in our study. As previously, we conducted two experiments in case 6a, switching the reacting partners with respect to the consecutive moieties. Both attempts however resulted in very good yield of 6a. Overall, the benzophenone products 6a–6i were obtained in very good to excellent yields (75–91%). The gram-scale attempts for 6a (from phenol 1a) and 6d (from KAT 5a) resulted in 77% and 79% yields respectively, what confirms the usefulness and generality of our protocol.

**Scheme 3:**
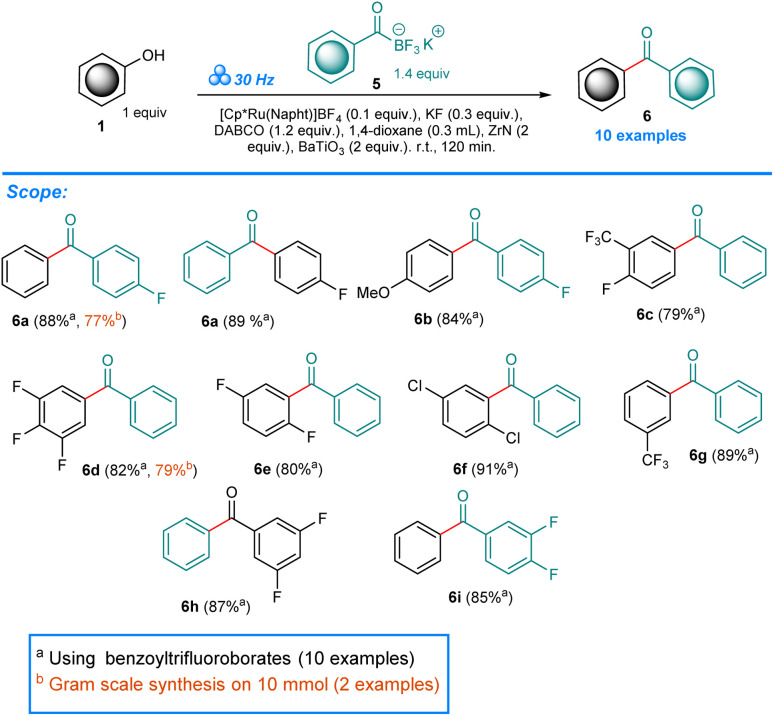
Synthesis of benzophenones 6 utilizing the method developed.

In Scheme 4 we present a mechanistic hypothesis for the title transformation, partially based on the previously mentioned paper by Shi *et al.* on Rh-catalyzed amination^16^ and our findings from DFT calculations. Initially, the chlorobenzene in the Cpx-0 sandwich complex (designated as R-reference in energy = 0 kcal mol^−1^) undergoes exchange by phenol to form Cpx-I (the Int1 is 4.2 kcal mol^−1^ more stable with respect to chlorobenzene-Ru-Cp* complex R). Deprotonation of the Ru-coordinated phenol in Cpx-I in the presence of DABCO base results in a neutral Cpx-IIa form (not calculated) that tautomerizes to the η^5^-phenoxo complex Cpx-II (Int2, which is about 12.3 kcal mol^−1^ more stable than R). At Int1, the C–OH bond distance is 1.36 Å, which decreases to 1.24 Å in the case of Int2 (C=O bond). Removal of oxygen from complex Int2 is not feasible without an additional additive. In this regard, zirconium nitride a low-cost and earth-abundant element is a promising candidate to replace platinum-based catalysts, which are quite expensive and scarce.^48^ The low cost and versatile nature of ZrN offers promising potential for extensive applications in energy conversion processes.^49^ The addition of ZrN facilitates the introduction of the aryl (Ar) group *via* formal activation of the C=O bond of the η^5^-phenoxo complex. At Int3, the arylboronic acid is introduced to Ru-complex with ZrN additive, which is energetically stabilized at −13.6 kcal mol^−1^ with respect to the starting reactants (R). The oxygen atom of the C=O bond interacts with Zr of ZrN at the bond distance of 2.05 Å, whereas the C–B bond of arylboronic acid has a bond distance of 1.53 Å (not shown). At this stage, we performed natural bond orbital (NBO) analysis to confirm the mode of charge transfer, while the mechanism proceeds from Int2 to Int3. At Int2, the amount NBO charge calculated at ruthenium is −0.014*e*^−^, whereas at Int3 this charge is −0.302*e*^−^. Therefore, the accumulation of NBO charge is observed over Ru metal while heading towards Int3, which suggests that the mechanism follows ligand-to-metal charge transfer. LMCT has been described and extensively studied also for sandwich-type complexes.^50–53^ In our case, it would be the first example of using mechanochemistry instead of photochemistry to induce LMCT in a sandwich catalyst complex. Nucleophilic attack on the *δ*^+^ carbonyl carbon in C=O by the *δ*^−^ carbon in the C–B of boronic acid results in a transition state (TS), with the activation barrier of 26.2 kcal mol^−1^ with reference to Int3, and 12.6 kcal mol^−1^ with respect to the starting point (R). At the transition state (TS), the aryl group interacts with the C atom of the phenoxo complex at a distance of 2.11 Å. Similarly, the C–B bond distance of arylboronic acid increases from 1.53 Å in Int3 to 1.76 Å at the TS. Moreover, the C=O bond length is also increased from 1.32 Å in Int3 to 1.38 Å at the transition state. The product of this reaction is Int4, which is stable by 20.8 kcal mol^−1^ with respect to the transition state and is located at 8.2 kcal mol^−1^ with respect to initial reactants (R). At Int4, the Ar–Ar (C–C) bond length is further reduced to 1.57 Å from 2.11 Å (TS). After Int4, the reaction follows the replacement of ZrN with boronic group –B(OH)_2_. Upon bond rearrangement forms Cpx-III (Int5), which is stable at 3.3 kcal mol^−1^ with respect to initial reactant (R). At Int5, the Ar–Ar (C–C) bond length is further reduced to 1.55 Å from 1.57 Å (Int4), after the removal of ZrN. Cpx-III (Int5) liberates formally boric acid upon interaction with HB. Cpx-IV (Int6) is thermodynamically stable by 31.2 kcal mol^−1^ of energy with respect to R. The C–C (Ar–Ar) bond length further reduces from 1.55 Å (Int5) to 1.49 Å (Int6) with the removal of boric acid. Protonation of Int5 forces the electron density to fall back and Ru(i) returns to Ru(ii) oxidation state. The resultant positively charged η^6^-biphenyl complex Cpx-IV (Int6) undergoes an aryl exchange step, consuming the next equivalent of phenol, liberating the biphenyl product (P) and restoring the active complex Cpx-I. The final product (P) in our case is stable by 26.2 kcal mol^−1^ with respect to that of the initial reactants (R).

**Scheme 4:**
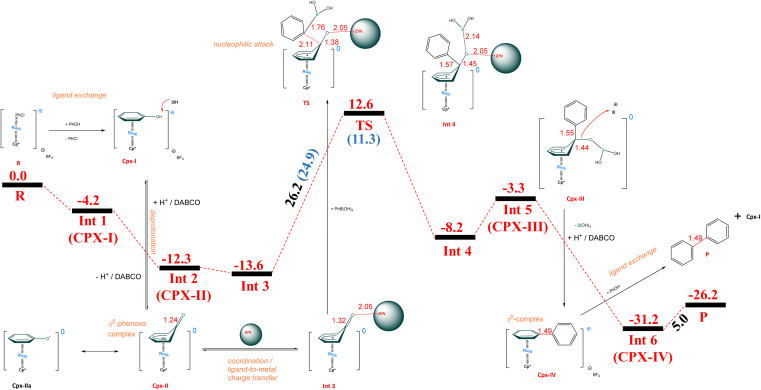
Proposed plausible mechanism of the title transformation with boronic acids including DFT energy profile for Ru-catalysed formal activation of Ph–OH bond, transformed to biphenyl. All the reported energy values are presented in kcal mol^−1^ with reference initial reactant (R) at 0.0 kcal mol^−1^ at B3LYP/Def2TZVPP level of theory. Measured bond lengths are reported in Angstroms (Å). Values in blue color present dispersion corrected energies at B3LYP-D3 functional.

Additionally, we also performed calculations for the inner-sphere mechanism of C–OH bond activation to biphenyl (Ar–Ar), presented in Fig. S3.† As per the energy profile, the inner-sphere mechanism for converting free phenols to biphenyl includes three transition states. The reaction pathway begins with the coordination of phenol to Ru-cyclopentadienyl complex, resulting in the formation of phenol-Ru-cyclopentadienyl sandwich complex (Int1). The phenol-Ru-cyclopentadienyl sandwich complex in this case (Int1) is 8.6 kcal mol^−1^ more stable with respect to R. Then the coordination of the OH group proceeds with the location of transition sate (TS1), which is located at the barrier height of 29.4 kcal mol^−1^ with respect to Int1 and 20.8 kcal mol^−1^ with respect to R. At Int1, C–OH and Ru–OH bond distances are 1.40 Å and 2.31 Å, respectively. Whereas at TS1 C–OH bond distance increases to 1.88 Å and Ru–OH bond distance decreases to 2.16 Å. The of this reaction is Int2, which is 6.7 kcal mol^−1^ more stable than R. The mechanism further proceeds with the simultaneous addition of arylboronic acid and removal of the OH group. For this step, the transition state (TS2) is located at the barrier height of 58.1 kcal mol^−1^ with respect to Int3 and 47.5 kcal mol^−1^ with reference to R. The interaction distances are 1.62 Å, 1.87 Å, 1.92 Å and 2.10 Å for C–B, O–B, Ru–O and Ru–C bonds, respectively. The product of this reaction (Int4) is 8.4 kcal mol^−1^ more stable than R. After the removal of boronic acid [B(OH)_3_], Int5 is obtained at −4.6 kcal mol^−1^. The Ar–Ar (C–C) bond length at Int5 is 2.35 Å. Formation of C–C bond for Ar–Ar compound formation, transition state (TS3) is obtained at the barrier height of 23.3 kcal mol^−1^ with respect to Int5. The Ar–Ar (C–C) bond length decreased to 1.93 Å (TS3) from 2.35 Å (Int5). The product of TS3 (Int6) is observed at 7.3 kcal mol^−1^ reference to R. The C–C bond length for Ar–Ar compound in the final product is 27.7 kcal mol^−1^.

Overall, the inner-sphere mechanism reveals that the activation barriers for the transition state (TS1) and (TS3) are 29.4 and 23.3 kcal mol^−1^, respectively. These activation barriers are somehow achievable under mechanochemical conditions. However, TS2 is located for the simultaneous addition of aryl and removal of OH group at the barrier height of 58.1 kcal mol^−1^. This step is thermodynamically not feasible to achieve under mechanochemical conditions. Therefore, the outer-sphere mechanism of Ru-catalyzed activation of phenols in a one-step under mechanochemical conditions to biphenyl (Ar–Ar) is considered as more plausible reaction mechanism. DFT study reveals that the Ru-catalyzed formal activation of the C–OH bond (free phenols) relies on a combination of Ru sandwich complex and ZrN as additive. The kinetic aspects of the studied steps indicate that the Ru-catalyzed activation of phenols (–OH groups) to biphenyl products is a thermodynamically viable process due to the accessible energy barrier (24.07 kcal mol^−1^) under mechanochemical reaction conditions.^54^

In the case of aryl trialkoxysilanes a similar mechanism can be postulated. The ArSi(OMe)_3_ forms an anion in reaction with CsF prior to the attack on the *δ*^+^ carbonyl carbon in C=O. Loss of F^−^ results in a neutral complex with both phenyl and OSi(OMe)_3_ moieties attached to the η^5^-phenoxo radical complex. Elimination of formally HOSi(OMe)_3_ upon interaction with HB gives the Cpx-IV complex.

In the case of KATs, the catalytic nature of this process requiring ZrN and a second solid additive (piezoelectric BaTiO_3_), as well as lack of reactivity in solution are not clear. The requirement for a piezoelectric material under mechanochemical conditions suggests a net redox-neutral cycle mechanism (polarisation *via* crystal deformation of BaTiO_3_ material enables single electron reduction and subsequent oxidation upon return to the ground state), that is similar to the oxidative quenching cycle of a photoredox catalyst.^55^ The investigation of the mechanistic details of this KAT process expands beyond the scope of this paper and will be the subject of future investigations in our group.^56^

Concluding, we have developed the first direct, Ru-catalyzed Suzuki–Miyaura and Hiyama-type cross-coupling of phenols with arylboronic acids and aryl trialkoxysilanes as nucleophilic partners under mechanochemical conditions. The reaction relies on a combination of Ruthenium sandwich complex, mechanochemical conditions and ZrN as additive delivering simple and complex biphenyl products in very good yields. We also documented the unusual reactivity of potassium benzoyltrifluoroborates within the conditions of our protocol, resulting in benzophenones.

## Data availability

Data for this article, including [procedures, NMR spectra description, PDFs of NMR spectra, DFT results] are available at the ESI.†

## Author contributions

Conceptualization: V. O. I.; methodology: S. M., V. O. I.; investigation: S. M., M. J., S. S., K. A., and V. O. I.; writing – original draft: S. M., M. J., and V. O. I.; writing – review & editing: S. M., M. J., and V. O. I.; funding acquisition: V. O. I.; resources: V. O. I.; supervision: V. O. I.

## Conflicts of interest

The authors declare no competing financial interests.

## Supplementary Material

SC-OLF-D4SC01704H-s001
