# Supplementary material for: Ru-catalyzed activation of free phenols in a one-step Suzuki–Miyaura cross-coupling under mechanochemical conditions
Source: Chem Sci. 2024 Aug 15;15(36):14798–805. doi: 10.1039/d4sc01704h (PMC11342143; doi:10.1039/d4sc01704h)
Supplement: SC-015-D4SC01704H-s001 [file SC-015-D4SC01704H-s001.pdf]

Supplementary Information (SI) for Chemical Science.
This journal is © The Royal Society of Chemistry 2024

Ru-catalyzed activation of free phenols in a one-step Suzuki-Miyaura cross-coupling under mechanochemical conditions.

Satenik Mkrtchyan[a,b], Michał Jakubczyk[c,d], Sehrish Sarfaraz[e], Khurshid Ayub[e], Viktor O. Iaroshenko[a,b,f,g,h]*

[a]Department of Chemistry, Faculty of Natural Sciences, Matej Bel University, Tajovského 40, 97401, Banska Bystrica, Slovakia.

[b]University Centre for Research & Development, Chandigarh University, Mohali, Punjab, 140413, India.

[c]Institute of Inorganic Chemistry, Czech Academy of Sciences, Husinec-Řež č.p. 1001, 250 68 Husinec-Řež, Czech Republic.

[d]Laboratory of Molecular Assays and Imaging, Institute of Bioorganic Chemistry, Polish Academy of Sciences, Noskowskiego 12/14, 61-704, Poznań, Poland.

[e]Department of Chemistry, COMSATS University, Abbottabad Campus, Abbottabad, KPK, 22060, Pakistan.

[f]Division of Wood Chemistry and Pulp Technology, Department of Fiber and Polymer Technology, School of Chemistry, Biotechnology and Health, KTH Royal Institute of Technology, Teknikringen 56-58, SE-100 44 Stockholm, Sweden.

[g]Functional Materials Group, Gulf University for Science and Technology, Mubarak Al-Abdullah 32093, Kuwait.

[h]Centre of Research Impact and Outcome, Chitkara University Institute of Engineering and Technology, Chitkara University, Rajpura-140401, Punjab, India.

*Corresponding author, Email: iva108@gmail.com , Iaroshenko.V@gust.edu.kw , viktor.iaroshenko@umb.sk

Table of Contents

(A) Experimental Section S2
I. Scope of starting materials used S3
II. Reaction conditions screening S5
III. Reaction procedures with optimised reaction conditions S7
(B) Characterization of products S9
(C) Copies ^{1}H and ^{13}C NMR spectra S36
(D) DFT Study S107

(A) Experimental Section.

Commercially available starting materials, reagents, catalysts, anhydrous and degassed solvents were used without further purification. Flash column chromatography was performed with Merck Silica gel 60 (230-400 mesh). The solvents for column chromatography were distilled before the use. Thin layer chromatography was carried out using Merck TLC Silica gel 60 F_{254} and visualized by short-wavelength ultraviolet light or by treatment with potassium permanganate ($KMnO_4$) stain. ^{1}H, ^{13}C and ^{19}F NMR spectra were recorded on a Bruker 400 and 500 MHz at 20ºC. All ^{1}H NMR spectra are reported in parts per million (ppm) downfield of TMS and were measured relative to the signals for $CHCl_3$ (7.26 ppm) and DMSO (2.50 ppm). All $^{13}C\{^{1}H\}$ NMR spectra were reported in ppm relative to residual $CHCl_3$ (77.00 ppm) or DMSO (39.70 ppm) and were obtained with ^{1}H decoupling. Coupling constants, *J*, are reported in Hertz (Hz). Gas chromatographic analyses was performed on Gas Chromatograph Mass Spectrometer GCMS-QP2010 Ultra instrument. Mechanochemical synthesis was performed using the Retsch MM400 mill using the standard kit. Liquid chemicals were dosed using gas tight micro syringes. Isolation of obtained compounds was achieved by column chromatography on Silica gel. All commercially available compounds were purchased from appropriate vendors.

A-1. Scope of the starting materials used.

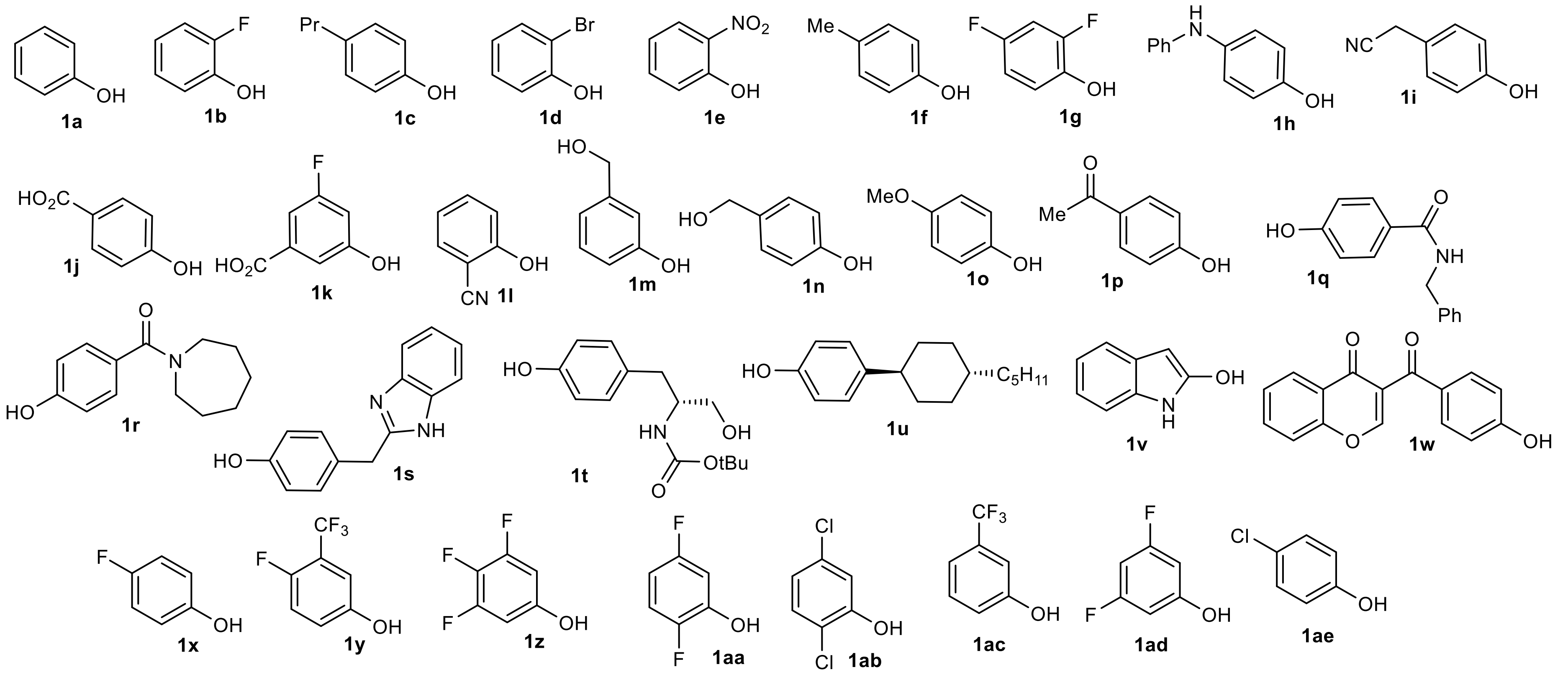

Scheme 1. Scope of phenols **1** used.

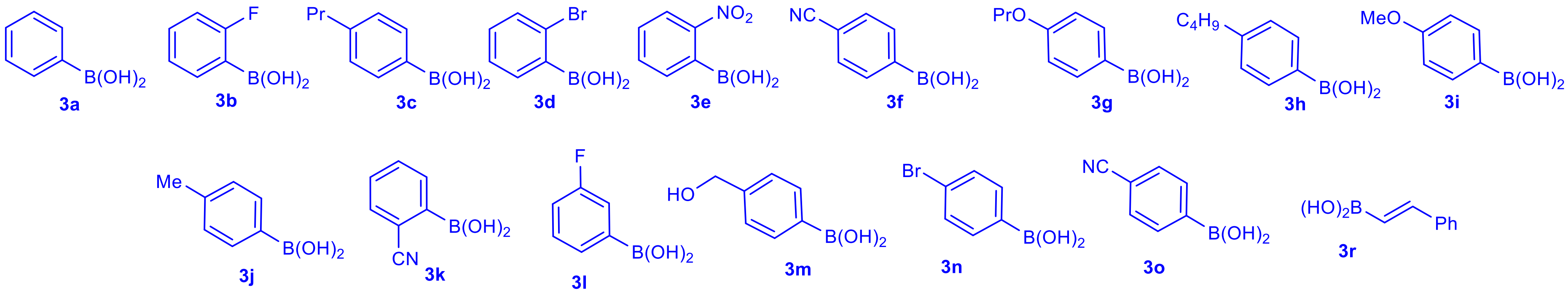

Scheme 2. Scope of boronic acids **3** used.

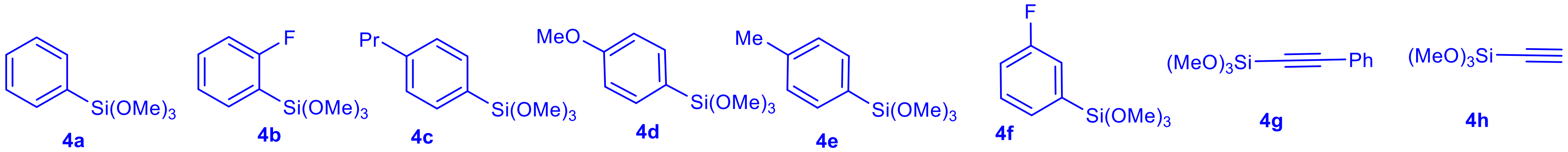

Scheme 3. Scope of aryl trialkoxysilanes **4** used.

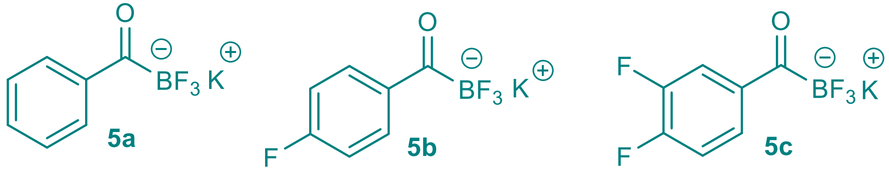

Scheme 4. Scope of potassium benzoyltrifluoroborates used.

A-2. Reaction condition screening.

Table S1: Optimization of the mechanochemical reaction conditions.

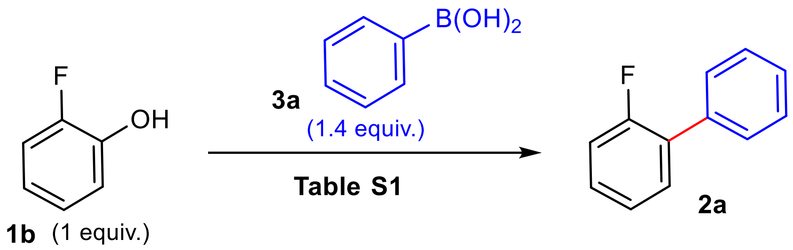

Table S1			
Entr.	Reaction components	Frequency/Time/ Temperature	yield (%) **2a**
1	[Cp*Ru(Napht)]BF_4 (0.1 equiv.), CsF (0.3 equiv.), DABCO (1.2 equiv.), ZrN (4 equiv.).	30Hz/90min/r.t.	12
2	[Cp*Ru(Napht)]BF_4 (0.1 equiv.), CsF (0.3 equiv.), DABCO (1.2 equiv.), 1,4-dioxane (0.3 mL), ZrN (4 equiv.).	30Hz/90min/r.t.	43
3	[Cp*Ru(Napht)]BF_4 (0.1 equiv.), KF (0.3 equiv.), DABCO (1.2 equiv.), 1,4-dioxane (0.3 mL), ZrN (4 equiv.).	30Hz/90min/r.t.	45
4	[Cp*Ru(Napht)]BF_4 (0.1 equiv.), NaF (0.3 equiv.), DABCO (1.2 equiv.), 1,4-dioxane (0.3 mL), ZrN (4 equiv.).	30Hz/90min/r.t.	28
5	[Cp*$RuCl_2$]$_2$ (0.1 equiv.), KF (0.3 equiv.), DABCO (1.2 equiv.), 1,4-dioxane (0.3 mL), ZrN (4 equiv.).	30Hz/90min/r.t.	0
6	[Cp*RuCl]$_4$ (0.1 equiv.), KF (0.3 equiv.), DABCO (1.2 equiv.), 1,4-dioxane (0.3 mL), ZrN (4 equiv.).	30Hz/90min/r.t.	0
7	[(*p*-cymene)$RuCl_2$]$_2$ (0.1 equiv.), KF (0.3 equiv.), DABCO (1.2 equiv.), 1,4-dioxane (0.3 mL), ZrN (4 equiv.).	30Hz/90min/r.t.	22
8	[$(C_6H_6)_2RuCl_2$]$_2$ (0.1 equiv.), KF (0.3 equiv.), DABCO (1.2 equiv.), 1,4-dioxane (0.3 mL), ZrN (4 equiv.).	30Hz/90min/r.t.	0
9	[$(C_6H_6)_2$Ru]$(BF_4)_2$ (0.1 equiv.), KF (0.3 equiv.), DABCO (1.2 equiv.), 1,4-dioxane (0.3 mL), ZrN (4 equiv.).	30Hz/90min/r.t.	17
10	[Cp*Ru$(MeCN)_3$]$(BF_4)_2$ (0.1 equiv.), KF (0.3 equiv.), DABCO (1.2 equiv.), 1,4-dioxane (0.3 mL), ZrN (4 equiv.).	30Hz/90min/r.t.	21
11	[Cp*Ru(PhCl)]PF_6 (0.1 equiv.), KF (0.3 equiv.), DABCO (1.2 equiv.), 1,4-dioxane (0.3 mL), ZrN (4 equiv.).	30Hz/90min/r.t.	61
**12**	**[Cp*Ru(PhCl)]BF_4 (0.1 equiv.), KF (0.3 equiv.), DABCO (1.2 equiv.), 1,4-dioxane (0.3 mL), ZrN (4 equiv.).**	**30Hz/90min/r.t.**	**81**
13	[Cp*Ru(PhCl)]BF_4 (0.1 equiv.), NaF (0.3 equiv.), DABCO (1.2 equiv.), 1,4-dioxane (0.3 mL), ZrN (4 equiv.).	30Hz/90min/r.t.	33
14	[Cp*Ru(PhCl)]BF_4 (0.1 equiv.), CsF (0.3 equiv.), DABCO (1.2 equiv.), 1,4-dioxane (0.3 mL), ZrN (4 equiv.).	30Hz/90min/r.t.	67
	**Reactions in solution**		
15	[Cp*Ru(PhCl)]BF_4 (0.1 equiv.), KF (0.3 equiv.), DABCO (1.2 equiv.) ZrN (4 equiv.), toluene, reflux.	------/24 h	0
16	[Cp*Ru(PhCl)]BF_4 (0.1 equiv.), KF (0.3 equiv.), DABCO (1.2 equiv.) ZrN (4 equiv.), benzene, reflux.	------/24 h	0
17	[Cp*Ru(PhCl)]BF_4 (0.1 equiv.), KF (0.3 equiv.), DABCO (1.2 equiv.) ZrN (4 equiv.), 1,4-dioxan, reflux.	------/24h	0
18	[Cp*Ru(PhCl)]BF_4 (0.1 equiv.), KF (0.3 equiv.), DABCO (1.2 equiv.) ZrN (4 equiv.), xylenes, 130 °C.	------/24h	0
19	[Cp*Ru(PhCl)]BF_4 (0.1 equiv.), KF (0.3 equiv.), DABCO (1.2 equiv.) ZrN (4 equiv.), neat, 165 °C.	------/24h	0

Table S2: Optimization of the mechanochemical reaction conditions.

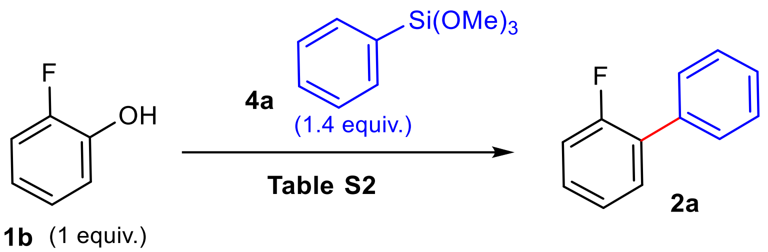

Table S2			
Entr.	Reaction components	Frequency/Time/ Temperature	yield (%) **2a**
1	$[Cp^{*}Ru(PhCl)]BF_4$ (0.1 equiv.), KF (0.3 equiv.), DABCO (1.2 equiv.), 1,4-dioxane (0.3 mL), ZrN (4 equiv.).	30Hz/90min/r.t.	52
2	$[Cp^{*}Ru(PhCl)]BF_4$ (0.1 equiv.), NaF (0.3 equiv.), DABCO (1.2 equiv.), 1,4-dioxane (0.3 mL), ZrN (4 equiv.).	30Hz/90min/r.t.	29
**3**	**$[Cp^{*}Ru(PhCl)]BF_4$ (0.1 equiv.), CsF (0.3 equiv.), DABCO (1.2 equiv.), 1,4-dioxane (0.3 mL), ZrN (4 equiv.).**	**30Hz/90min/r.t.**	**78**
	**Reactions in solution**		
4	$[Cp^{*}Ru(PhCl)]BF_4$ (0.1 equiv.), CsF (0.3 equiv.), DABCO (1.2 equiv.) ZrN (4 equiv.), toluene, reflux.	------/24 h	0
5	$[Cp^{*}Ru(PhCl)]BF_4$ (0.1 equiv.), CsF (0.3 equiv.), DABCO (1.2 equiv.) ZrN (4 equiv.), benzene, reflux.	------/24 h	0
6	$[Cp^{*}Ru(PhCl)]BF_4$ (0.1 equiv.), CsF (0.3 equiv.), DABCO (1.2 equiv.) ZrN (4 equiv.), 1,4-dioxan, reflux.	------/24h	0
7	$[Cp^{*}Ru(PhCl)]BF_4$ (0.1 equiv.), CsF (0.3 equiv.), DABCO (1.2 equiv.) ZrN (4 equiv.), xylenes, 130 °C.	------/24h	0
8	$[Cp^{*}Ru(PhCl)]BF_4$ (0.1 equiv.), CsF (0.3 equiv.), DABCO (1.2 equiv.) ZrN (4 equiv.), neat, 165 °C.	------/24h	0

Table S3: Optimization of the mechanochemical reaction conditions.

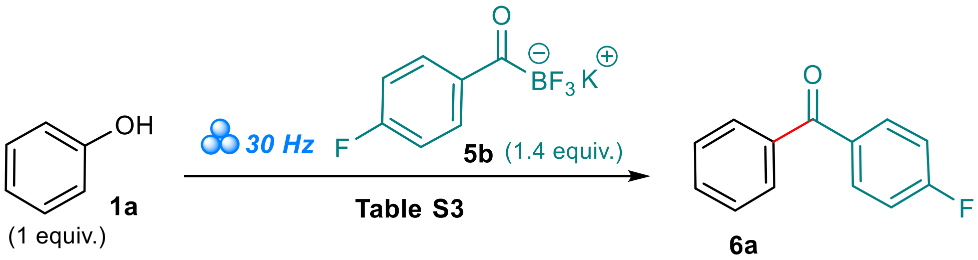

Table S3			
Entr.	Reaction components	Frequency/Time/ Temperature	yield (%) **6a**

1	[Cp*Ru(PhCl)]PF_6 (0.1 equiv.), KF (0.3 equiv.), DABCO (1.2 equiv.), 1,4-dioxane (0.3 mL), ZrN (4 equiv.).	30Hz/90min/r.t.	0
2	[Cp*Ru(PhCl)]BF_4 (0.1 equiv.), KF (0.3 equiv.), DABCO (1.2 equiv.), 1,4-dioxane (0.3 mL), ZrN (4 equiv.).	30Hz/90min/r.t.	0
3	[Cp*Ru(PhCl)]BF_4 (0.1 equiv.), NaF (0.3 equiv.), DABCO (1.2 equiv.), 1,4-dioxane (0.3 mL), ZrN (4 equiv.).	30Hz/90min/r.t.	0
4	[Cp*Ru(PhCl)]BF_4 (0.1 equiv.), CsF (0.3 equiv.), DABCO (1.2 equiv.), 1,4-dioxane (0.3 mL), ZrN (4 equiv.).	30Hz/90min/r.t.	0
5	[Cp*Ru(PhCl)]BF_4 (0.1 equiv.), CsF (0.3 equiv.), DABCO (1.2 equiv.), 1.4-dioxane (0.3mL), $BaBeO_2$ (2 equiv.).	30Hz/90min/r.t.	0
6	[Cp*Ru(Napht)]BF_4 (0.1 equiv.), KF (0.3 equiv.), DABCO (1.2 equiv.), 1,4-dioxane (0.3 mL), ZrN (4 equiv.).	30Hz/90min/r.t.	0
7	[Cp*Ru(Napht)]BF_4 (0.1 equiv.), NaF (0.3 equiv.), DABCO (1.2 equiv.), 1,4-dioxane (0.3 mL), ZrN (4 equiv.).	30Hz/90min/r.t.	0
8	[Cp*Ru(Napht)]BF_4 (0.1 equiv.), CsF (0.3 equiv.), DABCO (1.2 equiv.), 1,4-dioxane (0.3 mL), ZrN (4 equiv.).	30Hz/90min/r.t.	0
9	[Cp*Ru(Napht)]BF_4 (0.1 equiv.), KF (0.3 equiv.), DABCO (1.2 equiv.), 1,4-dioxane (0.3 mL), HfN (4 equiv.).	30Hz/90min/r.t.	0
10	[Cp*Ru(Napht)]BF_4 (0.1 equiv.), KF (0.3 equiv.), DABCO (1.2 equiv.), 1,4-dioxane (0.3 mL), ZrN (4 equiv.), $BaBeO_2$ (2 equiv.).	30Hz/90min/r.t.	17
11	[Cp*Ru(Napht)]BF_4 (0.1 equiv.), KF (0.3 equiv.), DABCO (1.2 equiv.), 1,4-dioxane (0.3 mL), ZrN (4 equiv.), $LiNbO_3$ (2 equiv.).	30Hz/90min/r.t.	29
12	[Cp*Ru(Napht)]BF_4 (0.1 equiv.), KF (0.3 equiv.), DABCO (1.2 equiv.), 1,4-dioxane (0.3 mL), ZrN (4 equiv.), $SrTiO_3$ (2 equiv.).	30Hz/90min/r.t.	11
13	[Cp*Ru(Napht)]BF_4 (0.1 equiv.), KF (0.3 equiv.), DABCO (1.2 equiv.), 1,4-dioxane (0.3 mL), ZrN (4 equiv.), ZnO (2 equiv.).	30Hz/90min/r.t.	Trace
14	[Cp*Ru(Napht)]BF_4 (0.1 equiv.), KF (0.3 equiv.), DABCO (1.2 equiv.), 1,4-dioxane (0.3 mL), ZrN (4 equiv.), $BaTiO_3$ (2 equiv.).	30Hz/90min/r.t.	90
**15**	**[Cp*Ru(Napht)]BF_4 (0.1 equiv.), KF (0.3 equiv.), DABCO (1.2 equiv.), 1,4-dioxane (0.3 mL), ZrN (2 equiv.), $BaTiO_3$ (2 equiv.).**	**30Hz/120min/r.t.**	**89**
16	[Cp*Ru(Napht)]BF_4 (0.1 equiv.), NaF (0.3 equiv.), DABCO (1.2 equiv.), 1,4-dioxane (0.3 mL), ZrN (2 equiv.), $BaTiO_3$ (2 equiv.).	30Hz/90min/r.t.	54
17	[Cp*Ru(Napht)]BF_4 (0.1 equiv.), CsF (0.3 equiv.), DABCO (1.2 equiv.), 1,4-dioxane (0.3 mL), ZrN (2 equiv.), $BaTiO_3$ (2 equiv.).	30Hz/90min/r.t.	89
	**Reactions in solution**		
18	[Cp*Ru(Napht)]BF_4 (0.1 equiv.), KF (0.3 equiv.), DABCO (1.2 equiv.), ZrN (2 equiv.), $BaTiO_3$ (2 equiv.), toluene, reflux.	------/24 h	0
19	[Cp*Ru(Napht)]BF_4 (0.1 equiv.), KF (0.3 equiv.), DABCO (1.2 equiv.), ZrN (2 equiv.), $BaTiO_3$ (2 equiv.), benzene, reflux.	------/24 h	0
20	[Cp*Ru(Napht)]BF_4 (0.1 equiv.), KF (0.3 equiv.), DABCO (1.2 equiv.), ZrN (2 equiv.), $BaTiO_3$ (2 equiv.), 1,4-dioxan, reflux.	------/24h	0
21	[Cp*Ru(Napht)]BF_4 (0.1 equiv.), KF (0.3 equiv.), DABCO (1.2 equiv.), ZrN (2 equiv.), $BaTiO_3$ (2 equiv.), xylenes, 130 ^{o}C.	------/24h	0
22	[Cp*Ru(Napht)]BF_4 (0.1 equiv.), KF (0.3 equiv.), DABCO (1.2 equiv.), ZrN (2 equiv.), $BaTiO_3$ (2 equiv.), neat, 165 ^{o}C.	------/24h	0

A-3. Reaction procedure with optimised reaction conditions.

General procedure for the synthesis of biaryls 2 starting from phenols 1 and boronic acids 3:
In a glovebox under the constant purge of argon, 5 mL grinding vessel (made of stainless) equipped with two balls (made of stainless, diameter: 5 mm) was loaded consecutively with the starting phenol **1** (1 mmol, 1 equiv.), catalyst [Cp*Ru(PhCl)]BF_4 (43 mg, 0.1 mmol, 0.1 equiv.), an appropriate boronic acid (1.4 mmol, 1.4 equiv.), KF (17 mg, 0.3 mmol, 0.3 equiv.), DABCO (135 mg, 1.2 mmol, 1.2 equiv.), ZrN (420 mg, 4 mmol, 4 equiv.), and 0.3 mL of 1,4-dioxane. The reaction vessel was properly capped. Finally, the vessel was installed on the mill and subjected to milling at 30Hz for 90 minutes at room temperature. The

content of the vessel was generously treated with distilled water, filtrated and properly dried in vacuum. The resulting crude product was directly subjected to gradient flash chromatography on silica gel to isolate the desired product **2**.

The gram scale synthesis was performed on 10 mmol of the starting phenol **1** in 25 mL grinding vessel using three 10 mm balls.

General procedure for the synthesis of biaryls arenes 2 starting from phenols 1 and aryl trialkoxysilanes 4:

In a glovebox under the constant purge of argon, 5 mL grinding vessel (made of stainless) equipped with two balls (made of stainless, diameter: 5 mm) was loaded consecutively with the starting phenol **1** (1 mmol, 1 equiv.), catalyst [Cp*Ru(PhCl)]BF_4 (43 mg, 0.1 mmol, 0.1 equiv.), an appropriate aryl trialkoxysilanes (1.4 mmol, 1.4 equiv.), CsF (46 mg, 0.3 mmol, 0.3 equiv.), DABCO (135 mg, 1.2 mmol, 1.2 equiv.), ZrN (420 mg, 4 mmol, 4 equiv.), and 0.3 mL of 1,4-dioxane. The reaction vessel was properly capped. Finally, the vessel was installed on the mill and subjected to milling at 30Hz for 90 minutes at room temperature. The content of the vessel was generously treated with distilled water, filtrated and properly dried in vacuum. The resulting crude product was directly subjected to gradient flash chromatography on silica gel to isolate the desired product **2**.

The gram scale synthesis was performed on 10 mmol of the starting phenol **1** in 25 mL grinding vessel using three 10 mm balls.

General procedure for the synthesis of benzophenones 6 starting from phenols 1 and benzoyltrifluoroborates 5:

In a glovebox under the constant purge of argon, 5 mL grinding vessel (made of stainless) equipped with two balls (made of stainless, diameter: 5 mm) was loaded consecutively with the starting phenol **1** (1 mmol, 1 equiv.), catalyst [Cp*Ru(Napht)]BF_4 (44 mg, 0.1 mmol, 0.1 equiv.), an appropriate benzoyltrifluoroborate (1.4 mmol, 1.4 equiv.), KF (17 mg, 0.3 mmol, 0.3 equiv.), DABCO (135 mg, 1.2 mmol, 1.2 equiv.), ZrN (210 mg, 2 mmol, 2 equiv.), $BaTiO_3$ (466 mg, 2 mmol, 2 equiv.) and 0.3 mL of 1,4-dioxane. The reaction vessel was properly capped. Finally, the vessel was installed on the mill and subjected to milling at 30Hz for 120 minutes at room temperature. The content of the vessel was generously treated with distilled water, filtrated and properly dried in vacuum. The resulting crude product was directly subjected to gradient flash chromatography on silica gel to isolate the desired product **6**.

The gram scale synthesis was performed on 10 mmol of the starting phenol **1** in 25 mL grinding vessel using three 10 mm balls.

(B) Characterization of products.

2-fluoro-1,1'-biphenyl 2a

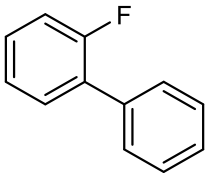

The title compound was prepared starting from phenol **1b** (112 mg, 1 mmol, 1 equiv.), catalyst [Cp*Ru(PhCl)]BF_4 (43 mg, 0.1 mmol, 0.1 equiv.), boronic acid **3a** (171 mg, 1.4 mmol, 1.4 equiv.), KF (17 mg, 0.3 mmol, 0.3 equiv.), DABCO (135 mg, 1.2 mmol, 1.2 equiv.), ZrN (420 mg, 4 mmol, 4 equiv.), and 0.3 mL of 1,4-dioxane. The purification was accomplished by column chromatography on silica gel to provide the desired product **2a** (139 mg, 0.81 mmol, 81%). The gram scale synthesis was performed on 10 mmol of the starting **1b** and **2a** was prepared in 77% yield (1.32 g, 7.7 mmol).

Alternatively the title compound was prepared starting fromphenol **1b** (112 mg, 1 mmol, 1 equiv.), [Cp*Ru(PhCl)]BF_4 (43 mg, 0.1 mmol, 0.1 equiv.), potassium trifluoro(phenyl)borate (256 mg, 1.4 mmol, 1.4 equiv.), KF (17 mg, 0.3 mmol, 0.3 equiv.), DABCO (135 mg, 1.2 mmol, 1.2 equiv.), ZrN (420 mg, 4 mmol, 4 equiv.), and 0.3 mL of 1,4-dioxane. The purification was accomplished by column chromatography on silica gel to provide the desired product **2a** (120 mg, 0.70 mmol, 70%).

Alternatively the title compound was prepared starting from phenol **1b** (112 mg, 1 mmol, 1 equiv.), [Cp*Ru(PhCl)]BF_4 (43 mg, 0.1 mmol, 0.1 equiv.), aryl trialkoxysilanes **4a** (277 mg, 1.4 mmol, 1.4 equiv.), CsF (46 mg, 0.3 mmol, 0.3 equiv.), DABCO (135 mg, 1.2 mmol, 1.2 equiv.), ZrN (420 mg, 4 mmol, 4 equiv.), and 0.3 mL of 1,4-dioxane. The purification was accomplished by column chromatography on silica gel to provide the desired product **2a** (134 mg, 0.78 mmol, 78%). The gram scale synthesis was performed on 10 mmol of the starting **1b** and **2a** was prepared in 77% yield (1.32 g, 7.7 mmol).

Alternatively the title compound was prepared starting from phenol **1a** (94 mg, 1 mmol, 1 equiv.), [Cp*Ru(PhCl)]BF_4 (43 mg, 0.1 mmol, 0.1 equiv.), boronic acid **3b** (196 mg, 1.4 mmol, 1.4 equiv.), KF (17 mg, 0.3 mmol, 0.3 equiv.), DABCO (135 mg, 1.2 mmol, 1.2 equiv.), ZrN (420 mg, 4 mmol, 4 equiv.), and 0.3 mL of 1,4-dioxane. The purification was accomplished by column chromatography on silica gel to provide the desired product **2a** (143 mg, 0.83 mmol, 83%).

Alternatively the title compound was prepared starting fromphenol **1a** (94 mg, 1 mmol, 1 equiv.), [Cp*Ru(PhCl)]BF_4 (43 mg, 0.1 mmol, 0.1 equiv.), potassium trifluoro(2-fluorophenyl)borate (283 mg, 1.4 mmol, 1.4 equiv.), KF (17 mg, 0.3 mmol, 0.3 equiv.), DABCO (135 mg, 1.2 mmol, 1.2 equiv.), ZrN (420 mg, 4 mmol, 4 equiv.), and 0.3 mL of 1,4-dioxane. The purification was accomplished by column chromatography on silica gel to provide the desired product **2a** (124 mg, 0.72 mmol, 72%).

The title compound was prepared starting from phenol **1a** (94 mg, 1 mmol, 1 equiv.), [Cp*Ru(PhCl)]BF_4 (43 mg, 0.1 mmol, 0.1 equiv.), aryl trialkoxysilanes **4b** (302 mg, 1.4 mmol, 1.4 equiv.), CsF (46 mg, 0.3 mmol, 0.3 equiv.), DABCO (135 mg, 1.2 mmol, 1.2 equiv.), ZrN (420 mg, 4 mmol, 4 equiv.), and 0.3 mL of 1,4-dioxane. The purification was accomplished by column chromatography on silica gel to provide the desired product **2a** (137 mg, 0.80 mmol, 80%).

White solid, mp 173 - 175 °C. **^{1}H NMR** (500 MHz, DMSO-d_6): δ = 7.28 – 7.32 (m, 2H, CH_{Ar}), 7.38 – 7.42 (m, 2H, CH_{Ar}), 7.46 – 7.49 (m, 2H, CH_{Ar}), 7.51 – 7.55 (m, 3H, CH_{Ar}).

^{13}C{^{1}H} NMR (126 MHz, DMSO-d_6): δ= 116.1 (d, J_{CF} = 20.0 Hz), 124.9 (d, J_{CF} = 3.4 Hz), 127.8, 128.2 (d, J_{CF} = 12.7 Hz), 128.6, 128.8 (m), 129.5 (d, J_{CF} = 9.2 Hz), 130.7 (d, J_{CF} = 3.0 Hz), 135.1, 159.1 (d, $^1J_{CF}$ = 252.7 Hz).

MS (GC, 70eV): m/z (%) = 172 (M^+, 100), 85 (17).

Anal. calcd. for $C_{12}H_9F$: C, 83.70; H, 5.27. Found: C, 83.76; H, 5.32.

4-propyl-1,1'-biphenyl 2b

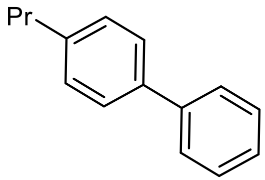

The title compound was prepared starting from phenol **1c** (136 mg, 1 mmol, 1 equiv.), [Cp*Ru(PhCl)]BF_4 (43 mg, 0.1 mmol, 0.1 equiv.), boronic acid **3a** (171 mg, 1.4 mmol, 1.4 equiv.), KF (17 mg, 0.3 mmol, 0.3 equiv.), DABCO (135 mg, 1.2 mmol, 1.2 equiv.), ZrN (420 mg, 4 mmol, 4 equiv.), and 0.3 mL of 1,4-dioxane. The purification was accomplished by column chromatography on silica gel to provide the desired product **2b** (161 mg, 0.82 mmol, 82%).

Alternatively the title compound was prepared starting from phenol **1c** (136 mg, 1 mmol, 1 equiv.), [Cp*Ru(PhCl)]BF_4 (43 mg, 0.1 mmol, 0.1 equiv.), aryl trialkoxysilanes **4a** (277 mg, 1.4 mmol, 1.4 equiv.), CsF (46 mg, 0.3 mmol, 0.3 equiv.), DABCO (135 mg, 1.2 mmol, 1.2 equiv.), ZrN (420 mg, 4 mmol, 4 equiv.), and 0.3 mL of 1,4-dioxane. The purification was accomplished by column chromatography on silica gel to provide the desired product **2b** (168 mg, 0.86 mmol, 86%).

Alternatively the title compound was prepared starting from phenol **1a** (94 mg, 1 mmol, 1 equiv.), [Cp*Ru(PhCl)]BF_4 (43 mg, 0.1 mmol, 0.1 equiv.), boronic acid **3c** (230 mg, 1.4 mmol, 1.4 equiv.), KF (17 mg, 0.3 mmol, 0.3 equiv.), DABCO (135 mg, 1.2 mmol, 1.2 equiv.), ZrN (420 mg, 4 mmol, 4 equiv.), and 0.3 mL of 1,4-dioxane. The purification was accomplished by column chromatography on silica gel to provide the desired product **2b** (167 mg, 0.85 mmol, 85%).

The title compound was prepared starting from phenol **1a** (94 mg, 1 mmol, 1 equiv.), [Cp*Ru(PhCl)]BF_4 (43 mg, 0.1 mmol, 0.1 equiv.), aryl trialkoxysilanes **4c** (336 mg, 1.4 mmol, 1.4 equiv.), CsF (46 mg, 0.3 mmol, 0.3 equiv.), DABCO (135 mg, 1.2 mmol, 1.2 equiv.), ZrN (420 mg, 4 mmol, 4 equiv.), and 0.3 mL of 1,4-dioxane. The purification was accomplished by column chromatography on silica gel to provide the desired product **2b** (167 mg, 0.85 mmol, 85%).

Yellow low melting solid. **^{1}H NMR** (500 MHz, *CDCl$_3$*): δ = 1.15 (dt, 3H, ^3J = 7.3 Hz, ^4J = 1.2 Hz, CH_3), 1.82 – 1.87 (m, 2H, CH_2), 2.79 (t, 2H, ^3J = 7.3 Hz, CH_2), 7.41 (d, 2H, ^3J = 8.0 Hz, CH_{Ar}), 7.47 – 7.49 (m, 1H, CH_{Ar}), 7.57 (t, 2H, ^3J = 7.7 Hz, CH_{Ar}), 7.68 (d, 2H, ^3J = 8.0 Hz, CH_{Ar}), 7.75 (d, 2H, ^3J = 8.0 Hz, CH_{Ar}).

^{13}C{^{1}H} NMR (126 MHz, *CDCl$_3$*): δ= 13.9, 24.5, 37.7, 126.9, 128.7, 128.9, 138.5, 141.7, 141.8.

MS (GC, 70eV): m/z (%) = 196 (M^+, 26), 167 (100), 152 (12).

Anal. calcd. for $C_{15}H_{16}$: C, 91.78; H, 8.22. Found: C, 91.69; H, 8.31.

2-bromo-1,1'-biphenyl 2c

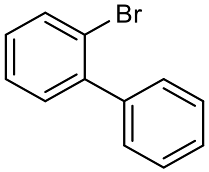

The title compound was prepared starting from phenol **1d** (173 mg, 1 mmol, 1 equiv.), catalyst [Cp*Ru(PhCl)]BF_4 (43 mg, 0.1 mmol, 0.1 equiv.), boronic acid **3a** (171 mg, 1.4 mmol, 1.4 equiv.), KF (17 mg, 0.3 mmol, 0.3 equiv.), DABCO (135 mg, 1.2 mmol, 1.2 equiv.), ZrN (420 mg, 4 mmol, 4 equiv.), and 0.3 mL of 1,4-dioxane. The purification was accomplished by column chromatography on silica gel to provide the desired product **2c** (172 mg, 0.74 mmol, 74%).
Alternatively the title compound was prepared starting from phenol **1a** (94 mg, 1 mmol, 1 equiv.), catalyst [Cp*Ru(PhCl)]BF_4 (43 mg, 0.1 mmol, 0.1 equiv.), boronic acid **3d** (280 mg, 1.4 mmol, 1.4 equiv.), KF (17 mg, 0.3 mmol, 0.3 equiv.), DABCO (135 mg, 1.2 mmol, 1.2 equiv.), ZrN (420 mg, 4 mmol, 4 equiv.), and 0.3 mL of 1,4-dioxane. The purification was accomplished by column chromatography on silica gel to provide the desired product **2c** (182 mg, 0.78 mmol, 78%).

Yellow liquid **^{1}H NMR** (500 MHz, DMSO-d_6): δ = 7.28 – 7.31 (m, 1H, CH_{Ar}), 7.35 – 7.40 (m, 4H, CH_{Ar}), 7.40 – 7.46 (m, 3H, CH_{Ar}), 7.72 (d, 1H, ^3J = 8.1 Hz, CH_{Ar}).

^{13}C{^{1}H} NMR (126 MHz, DMSO-d_6): δ= 122.3, 128.1, 128.4, 128.6, 129.6, 129.8, 131.9, 133.4, 140.9, 142.3.

MS (GC, 70eV): m/z (%) = 233 (M^+, 17), 232 (64), 181 (21), 152 (100).

Anal. calcd. for $C_{12}H_9Br$: C, 61.83; H, 3.89. Found: C, 61.89; H, 3.91.

2-nitro-1,1'-biphenyl 2d

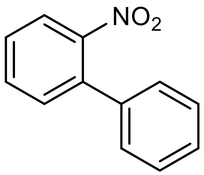

The title compound was prepared starting from phenol **1e** (139 mg, 1 mmol, 1 equiv.), catalyst [Cp*Ru(PhCl)]BF_4 (43 mg, 0.1 mmol, 0.1 equiv.), boronic acid **3a** (171 mg, 1.4 mmol, 1.4 equiv.), KF (17 mg, 0.3 mmol, 0.3 equiv.), DABCO (135 mg, 1.2 mmol, 1.2 equiv.), ZrN (420 mg, 4 mmol, 4 equiv.), and 0.3 mL of 1,4-dioxane. The purification was accomplished by column chromatography on silica gel to provide the desired product **2d** (153 mg, 0.77 mmol, 77%).
Alternatively the title compound was prepared starting from phenol **1e** (139 mg, 1 mmol, 1 equiv.), catalyst [Cp*Ru(PhCl)]BF_4 (43 mg, 0.1 mmol, 0.1 equiv.), aryl trialkoxysilanes **4a** (277 mg, 1.4 mmol, 1.4 equiv.), CsF (46 mg, 0.3 mmol, 0.3 equiv.), DABCO (135 mg, 1.2 mmol, 1.2 equiv.), ZrN (420 mg, 4 mmol, 4 equiv.),

and 0.3 mL of 1,4-dioxane. The purification was accomplished by column chromatography on silica gel to provide the desired product **2d** (157 mg, 0.79 mmol, 79%).

Alternatively the title compound was prepared starting from phenol **1a** (94 mg, 1 mmol, 1 equiv.), catalyst [Cp*Ru(PhCl)]BF_4 (43 mg, 0.1 mmol, 0.1 equiv.), boronic acid **3e** (234 mg, 1.4 mmol, 1.4 equiv.), KF (17 mg, 0.3 mmol, 0.3 equiv.), DABCO (135 mg, 1.2 mmol, 1.2 equiv.), ZrN (420 mg, 4 mmol, 4 equiv.), and 0.3 mL of 1,4-dioxane. The purification was accomplished by column chromatography on silica gel to provide the desired product **2d** (145 mg, 0.73 mmol, 73%).

Yellow solid, mp 36 - 38 °C. **^{1}H NMR** (500 MHz, *CDCl$_3$*): δ = 7.32 – 7.35 (m, 2H, CH_{Ar}), 7.41 – 7.51 (m, 5H, CH_{Ar}), 7.62 (dt, 1H, ^3J = 7.6 Hz, ^4J = 1.2 Hz, CH_{Ar}), 7.86 (dd, 1H, ^3J = 8.1 Hz, ^4J = 1.4 Hz, CH_{Ar}).

^{13}C{^{1}H} NMR (126 MHz, *CDCl$_3$*): δ= 124.0, 127.8, 128.1, 128.2, 128.6, 131.9, 132.5, 136.2, 137.3, 149.2.

MS (GC, 70eV): m/z (%) = 199 (M^+, 36), 182 (59), 171 (75), 152 (88), 115 (100).

Anal. calcd. for $C_{12}H_9NO_2$: C, 72.35; H, 4.55; N, 7.03. Found: C, 72.49; H, 4.39; N, 7.11.

4'-methyl-[1,1'-biphenyl]-4-carbonitrile 2e

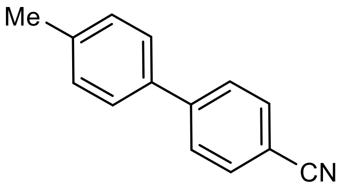

The title compound was prepared starting from phenol **1f** (108 mg, 1 mmol, 1 equiv.), catalyst [Cp*Ru(PhCl)]BF_4 (43 mg, 0.1 mmol, 0.1 equiv.), boronic acid **3f** (206 mg, 1.4 mmol, 1.4 equiv.), KF (17 mg, 0.3 mmol, 0.3 equiv.), DABCO (135 mg, 1.2 mmol, 1.2 equiv.), ZrN (420 mg, 4 mmol, 4 equiv.), and 0.3 mL of 1,4-dioxane. The purification was accomplished by column chromatography on silica gel to provide the desired product **2e** (160 mg, 0.83 mmol, 83%).

White solid, mp 108 - 110 °C. **^{1}H NMR** (500 MHz, DMSO-*d$_6$*): δ = 2.34 (s, 3H, Me), 7.28 (d, 2H, ^3J = 8.2 Hz, CH_{Ar}), 7.60 (d, 2H, ^3J = 8.2 Hz, CH_{Ar}), 7.81 (d, 2H, ^3J = 8.1 Hz, CH_{Ar}), 7.86 (d, 2H, ^3J = 8.2 Hz, CH_{Ar}).

^{13}C{^{1}H} NMR (126 MHz, DMSO-*d$_6$*): δ= 20.7, 109.7, 118.9, 126.8, 127.2, 129.7, 132.7, 135.3, 138.3, 144.5.

MS (GC, 70eV): m/z (%) = 193 (M^+, 100), 165 (24).

Anal. calcd. for $C_{14}H_{11}N$: C, 87.01; H, 5.74; N, 7.25. Found: C, 87.09; H, 5.63; N, 7.28.

2,4-difluoro-1,1'-biphenyl 2f

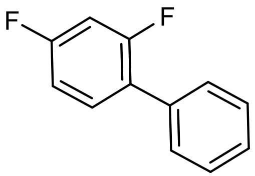

The title compound was prepared starting from phenol **1g** (130 mg, 1 mmol, 1 equiv.), catalyst [Cp*Ru(PhCl)]BF_4 (43 mg, 0.1 mmol, 0.1 equiv.), boronic acid **3a** (171 mg, 1.4 mmol, 1.4 equiv.), KF (17 mg, 0.3 mmol, 0.3 equiv.), DABCO (135 mg, 1.2 mmol, 1.2 equiv.), ZrN (420 mg, 4 mmol, 4 equiv.), and 0.3 mL of 1,4-dioxane. The purification was accomplished by column chromatography on silica gel to provide the desired product **2f** (160 mg, 0.84 mmol, 84%).

White solid, mp 61 - 63 °C. **^{1}H NMR** (500 MHz, DMSO-d_6): δ = 7.16 (ddd, 1H, ^3J = 8.7 Hz, J = 2.7 Hz, J = 0.8 Hz, CH_{Ar}), 7.30 – 7.34 (m, 1H, CH_{Ar}), 7.39 (dt, 1H, ^3J = 7.1 Hz, J = 2.1 Hz, CH_{Ar}), 7.44 – 7.57 (m, 5H, CH_{Ar}).

^{13}C{^{1}H} NMR (126 MHz, DMSO-d_6): δ= 104.5 (t, J_{CF} = 26.9 Hz), 111.9 (dd, J_{CF} = 21.1 Hz, J_{CF} = 3.5 Hz), 124.9 (dd, J_{CF} = 13.6 Hz, J_{CF} = 3.1 Hz), 127.9, 128.6, 128.7 (t, J_{CF} = 2.1 Hz), 131.8 (m), 134.3, 159.1 (dd, $^1J_{CF}$ = 249.0 Hz, J_{CF} = 12.6 Hz), 161.7 (dd, $^1J_{CF}$ = 245.5 Hz, J_{CF} = 12.4 Hz).

MS (GC, 70eV): m/z (%) = 190 (M^+, 100), 135 (28).

Anal. calcd. for $C_{12}H_8F_2$: C, 75.78; H, 4.24. Found: C, 75.86; H, 4.33.

4-propoxy-1,1'-biphenyl 2g

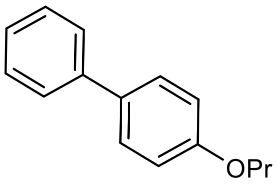

The title compound was prepared starting from phenol **1a** (94 mg, 1 mmol, 1 equiv.), catalyst [Cp*Ru(PhCl)]BF_4 (43 mg, 0.1 mmol, 0.1 equiv.), boronic acid **3g** (252 mg, 1.4 mmol, 1.4 equiv.), KF (17 mg, 0.3 mmol, 0.3 equiv.), DABCO (135 mg, 1.2 mmol, 1.2 equiv.), ZrN (420 mg, 4 mmol, 4 equiv.), and 0.3 mL of 1,4-dioxane. The purification was accomplished by column chromatography on silica gel to provide the desired product **2g** (184 mg, 0.87 mmol, 87%).

White solid, mp 76 - 77 ^oC. **^{1}H NMR** (500 MHz, DMSO-d_6): δ = 0.93 (s, 3H, CH_3), 1.64 (m, 2H, CH_2), 3.00 (m, 2H, CH_2), 7.42 – 7.49 (m, 3H, CH_{Ar}), 7.72 – 7.80 (m, 4H, CH_{Ar}), 8.02 – 8.03 (m, 2H, CH_{Ar}).

^{13}C{^{1}H} NMR (126 MHz, DMSO-d_6): δ= 13.6, 17.3, 126.9, 127.0, 128.3, 128.6, 129.0, 135.5, 138.9, 144.4, 199.5.

Anal. calcd. for $C_{12}H_9NO_2$: C, 72.35; H, 4.55; N, 7.03. Found: C, 72.49; H, 4.39; N, 7.11.

N-phenyl-[1,1'-biphenyl]-4-amine 2h

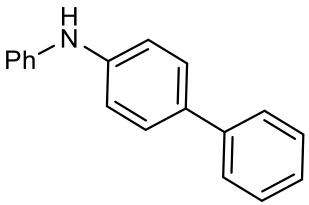

The title compound was prepared starting from phenol **1h** (185 mg, 1 mmol, 1 equiv.), catalyst [Cp*Ru(PhCl)]BF_4 (43 mg, 0.1 mmol, 0.1 equiv.), boronic acid **3a** (171 mg, 1.4 mmol, 1.4 equiv.), KF (17 mg, 0.3 mmol, 0.3 equiv.), DABCO (135 mg, 1.2 mmol, 1.2 equiv.), ZrN (420 mg, 4 mmol, 4 equiv.), and 0.3 mL of 1,4-dioxane. The purification was accomplished by column chromatography on silica gel to provide the desired product **2h** (203 mg, 0.83 mmol, 83%).

Alternatively the title compound was prepared starting from phenol **1h** (185 mg, 1 mmol, 1 equiv.), catalyst [Cp*Ru(PhCl)]BF_4 (43 mg, 0.1 mmol, 0.1 equiv.), aryl trialkoxysilanes **4a** (277 mg, 1.4 mmol, 1.4 equiv.), CsF (46 mg, 0.3 mmol, 0.3 equiv.), DABCO (135 mg, 1.2 mmol, 1.2 equiv.), ZrN (420 mg, 4 mmol, 4 equiv.), and 0.3 mL of 1,4-dioxane. The purification was accomplished by column chromatography on silica gel to provide the desired product **2h** (196 mg, 0.80 mmol, 80%).

Light Brown solid, mp 113 - 114 ^oC. **^{1}H NMR** (500 MHz, *CDCl$_3$*): δ = 5.77 (s, 1H, NH), 6.98 (t, 1H, ^3J = 7.4 Hz, CH_{Ar}), 7.15 (t, 4H, ^3J = 7.4 Hz, CH_{Ar}), 7.30 – 7.34 (m, 3H, CH_{Ar}), 7.44 (t, 2H, ^3J = 8.2 Hz, CH_{Ar}), 7.53 (d, 2H, ^3J = 8.6 Hz, CH_{Ar}), 7.59 (d, 1H, ^3J = 7.4 Hz, CH_{Ar}).

^{13}C{^{1}H} NMR (126 MHz, *CDCl$_3$*): δ= 117.8, 118.1, 121.2, 126.5, 126.6, 128.0, 128.7, 129.4, 133.7, 140.8, 142.6, 142.8.

MS (GC, 70eV): m/z (%) = 245 (M^+, 100).

Anal. calcd. for $C_{18}H_{15}N$: C, 88.13; H, 6.16; N, 5.71. Found: C, 88.19; H, 6.13; N, 5.68.

2-([1,1'-biphenyl]-4-yl)acetonitrile 2i

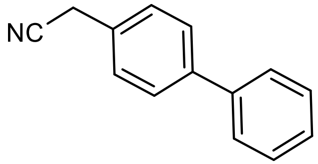

The title compound was prepared starting from phenol **1i** (133 mg, 1 mmol, 1 equiv.), catalyst [Cp*Ru(PhCl)]BF_4 (43 mg, 0.1 mmol, 0.1 equiv.), boronic acid **3a** (171 mg, 1.4 mmol, 1.4 equiv.), KF (17 mg, 0.3 mmol, 0.3 equiv.), DABCO (135 mg, 1.2 mmol, 1.2 equiv.), ZrN (420 mg, 4 mmol, 4 equiv.), and 0.3 mL of 1,4-dioxane. The purification was accomplished by column chromatography on silica gel to provide the desired product **2i** (203 mg, 0.87 mmol, 87%).

Alternatively the title compound was prepared starting from phenol **1i** (133 mg, 1 mmol, 1 equiv.), [Cp*Ru(PhCl)]BF_4 (43 mg, 0.1 mmol, 0.1 equiv.), aryl trialkoxysilanes **4a** (277 mg, 1.4 mmol, 1.4 equiv.), CsF (46 mg, 0.3 mmol, 0.3 equiv.), DABCO (135 mg, 1.2 mmol, 1.2 equiv.), ZrN (420 mg, 4 mmol, 4 equiv.), and 0.3 mL of 1,4-dioxane. The purification was accomplished by column chromatography on silica gel to provide the desired product **2i** (196 mg, 0.83 mmol, 83%).

Pink solid, mp 161 - 162 °C. **^{1}H NMR** (500 MHz, *CDCl$_3$*): δ = 3.78 (m, 2H, CH_2), 7.40 – 7.42 (m, 3H, CH_{Ar}), 7.46 – 7.50 (m, 2H, CH_{Ar}), 7.60 – 7.64 (m, 4H, CH_{Ar}).

^{13}C{^{1}H} NMR (126 MHz, *CDCl$_3$*): δ= 23.2, 118.0, 127.1, 127.7, 127.9, 128.4, 129.0, 140.2, 141.1.

MS (GC, 70eV): m/z (%) = 193 (M^+, 100), 165 (39).

Anal. calcd. for $C_{14}H_{11}N$: C, 87.01; H, 5.74; N, 7.25. Found: C, 86.95; H, 5.69; N, 7.36.

4'-butyl-[1,1'-biphenyl]-4-carboxylic acid 2j

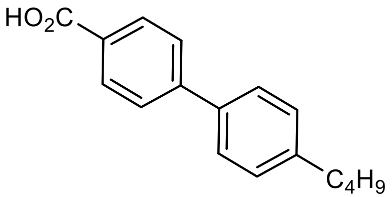

The title compound was prepared starting from phenol **1j** (138 mg, 1 mmol, 1 equiv.), catalyst [Cp*Ru(PhCl)]BF_4 (43 mg, 0.1 mmol, 0.1 equiv.), boronic acid **3h** (249 mg, 1.4 mmol, 1.4 equiv.), KF (17 mg, 0.3 mmol, 0.3 equiv.), DABCO (135 mg, 1.2 mmol, 1.2 equiv.), ZrN (420 mg, 4 mmol, 4 equiv.), and 0.3 mL of 1,4-dioxane. The purification was accomplished by column chromatography on silica gel to provide the desired product **2j** (188 mg, 0.74 mmol, 74%).

Colourless liquid **^{1}H NMR** (500 MHz, DMSO-d_6): δ = 0.91 (t, 3H, ^3J = 7.6 Hz, CH_3), 1.31 – 1.35 (m, 2H, CH_2), 1.56 – 1.60 (m, 2H, CH_2), 2.62 (t, 2H, ^3J = 8.3 Hz, CH_2), 7.31 (d, 2H, ^3J = 7.6 Hz, H_{Ar}), 7.64 (d, 2H, ^3J = 7.7 Hz, CH_{Ar}), 7.77 (d, 2H, ^3J = 8.6 Hz, CH_{Ar}), 8.00 (d, 2H, ^3J = 8.4 Hz, CH_{Ar}), 12.94 (s, 1H, OH).

^{13}C{^{1}H} NMR (126 MHz, DMSO-d_6): δ= 13.7, 21.7, 33.0, 34.4, 126.5, 126.8, 136.3, 142.6, 144.2, 167.1.

Anal. calcd. for $C_{17}H_{18}O_2$: C, 80.28; H, 7.13. Found: C, 80.39; H, 7.16.

5-fluoro-4'-methoxy-[1,1'-biphenyl]-3-carboxylic acid 2k

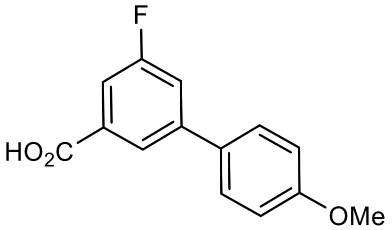

The title compound was prepared starting from phenol **1k** (156 mg, 1 mmol, 1 equiv.), catalyst [Cp*Ru(PhCl)]BF_4 (43 mg, 0.1 mmol, 0.1 equiv.), boronic acid **3i** (213 mg, 1.4 mmol, 1.4 equiv.), KF (17 mg, 0.3 mmol, 0.3 equiv.), DABCO (135 mg, 1.2 mmol, 1.2 equiv.), ZrN (420 mg, 4 mmol, 4 equiv.), and 0.3 mL of 1,4-dioxane. The purification was accomplished by column chromatography on silica gel to provide the desired product **2k** (204 mg, 0.83 mmol, 83%).

Alternatively the title compound was prepared starting from phenol **1k** (156 mg, 1 mmol, 1 equiv.), catalyst [Cp*Ru(PhCl)]BF_4 (43 mg, 0.1 mmol, 0.1 equiv.), aryl trialkoxysilanes **4d** (319 mg, 1.4 mmol, 1.4 equiv.), CsF (46 mg, 0.3 mmol, 0.3 equiv.), DABCO (135 mg, 1.2 mmol, 1.2 equiv.), ZrN (420 mg, 4 mmol, 4 equiv.),

and 0.3 mL of 1,4-dioxane. The purification was accomplished by column chromatography on silica gel to provide the desired product **2k** (197 mg, 0.80 mmol, 80%).

White liquid. ^{1}**H NMR** (500 MHz, DMSO-d_6): δ = 3.81 (s, 3H, OMe), 7.04 (d, 2H, ^{3}J = 8.6 Hz, CH_{Ar}), 7.58 (d, 1H, ^{3}J = 8.6 Hz, CH_{Ar}), 7.69 (d, 2H, ^{3}J = 8.6 Hz, CH_{Ar}), 7.75 (d, 1H, ^{3}J = 10.6 Hz, CH_{Ar}), 7.99 (s, 1H, CH_{Ar}), 13.37 (s, 1H, OH).

^{13}**C{^{1}H} NMR** (126 MHz, DMSO-d_6): δ= 55.2, 113.8 5 (d, J_{CF} = 22.0 Hz), 114.5, 117.2 (d, J_{CF} = 22.0 Hz), 122.7, 128.1, 130.2, 133.6 (d, J_{CF} = 7.8 Hz), 142.6 (d, J_{CF} = 7.8 Hz), 160.0, 162.5 (d, $^{1}J_{CF}$ = 243.9 Hz), 166.2.

Anal. calcd. for $C_{14}H_{11}FO_3$: C, 68.29; H, 4.50. Found: C, 68.21; H, 4.39.

4'-methyl-[1,1'-biphenyl]-2-carbonitrile 2l

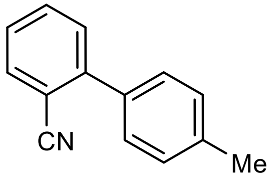

The title compound was prepared starting from phenol **1l** (119 mg, 1 mmol, 1 equiv.), catalyst [Cp*Ru(Napht)]BF_4 (44 mg, 0.1 mmol, 0.1 equiv.), boronic acid **3j** (190 mg, 1.4 mmol, 1.4 equiv.), KF (17 mg, 0.3 mmol, 0.3 equiv.), DABCO (135 mg, 1.2 mmol, 1.2 equiv.), ZrN (420 mg, 4 mmol, 4 equiv.), and 0.3 mL of 1,4-dioxane. The purification was accomplished by column chromatography on silica gel to provide the desired product **2l** (158 mg, 0.82 mmol, 82%).

Alternatively the title compound was prepared starting from phenol **1l** (119 mg, 1 mmol, 1 equiv.), catalyst [Cp*Ru(PhCl)]BF_4 (43 mg, 0.1 mmol, 0.1 equiv.), aryl trialkoxysilanes **4e** (297 mg, 1.4 mmol, 1.4 equiv.), CsF (46 mg, 0.3 mmol, 0.3 equiv.), DABCO (135 mg, 1.2 mmol, 1.2 equiv.), ZrN (420 mg, 4 mmol, 4 equiv.), and 0.3 mL of 1,4-dioxane. The purification was accomplished by column chromatography on silica gel to provide the desired product **2l** (162 mg, 0.84 mmol, 84%).

Alternatively the title compound was prepared starting from phenol **1f** (108 mg, 1 mmol, 1 equiv.), catalyst [Cp*Ru(PhCl)]BF_4 (43 mg, 0.1 mmol, 0.1 equiv.), boronic acid **3k** (206 mg, 1.4 mmol, 1.4 equiv.), KF (17 mg, 0.3 mmol, 0.3 equiv.), DABCO (135 mg, 1.2 mmol, 1.2 equiv.), ZrN (420 mg, 4 mmol, 4 equiv.), and

0.3 mL of 1,4-dioxane. The purification was accomplished by column chromatography on silica gel to provide the desired product **2l** (154 mg, 0.80 mmol, 80%).

Beige solid, mp 50 - 52 °C. **^{1}H NMR** (500 MHz, *$CDCl_3$*): δ = 2.43 (s, 3H, Me), 7.30 (d, 2H, ^3J = 8.1 Hz, CH_{Ar}), 7.42 (t, 1H, ^3J = 7.4 Hz, CH_{Ar}), 7.47 (d, 2H, ^3J = 8.1 Hz, CH_{Ar}), 7.51 (d, 1H, ^3J = 8.1 Hz, CH_{Ar}), 761 – 7.65 (m, 1H, CH_{Ar}), 7.75 (d, 1H, ^3J = 7.7 Hz, CH_{Ar}).

$^{13}C\{^1H\}$ NMR (126 MHz, *$CDCl_3$*): δ= 21.2, 111.2, 118.8, 127.2, 128.6, 129.4, 129.9, 132.7, 133.7, 135.2, 138.6, 145.5.

MS (GC, 70eV): m/z (%) = 193 (M^+, 100), 165 (28).

Anal. calcd. for $C_{14}H_{11}N$: C, 87.01; H, 5.74; N, 7.25. Found: C, 87.16; H, 5.69; N, 7.15.

(3'-fluoro-[1,1'-biphenyl]-3-yl)methanol 2m

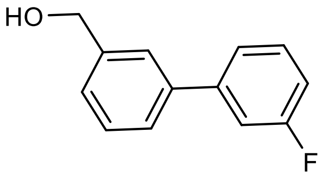

The title compound was prepared starting from phenol **1m** (124 mg, 1 mmol, 1 equiv.), catalyst [Cp*Ru(PhCl)]BF_4 (43 mg, 0.1 mmol, 0.1 equiv.), boronic acid **3l** (196 mg, 1.4 mmol, 1.4 equiv.), KF (17 mg, 0.3 mmol, 0.3 equiv.), DABCO (135 mg, 1.2 mmol, 1.2 equiv.), ZrN (420 mg, 4 mmol, 4 equiv.), and 0.3 mL of 1,4-dioxane. The purification was accomplished by column chromatography on silica gel to provide the desired product **2m** (178 mg, 0.88 mmol, 88%). The gram scale synthesis was performed on 10 mmol of the starting **1m** and **2m** was prepared in 78% yield (1.57 g, 7.8 mmol).

Alternatively the title compound was prepared starting from phenol **1m** (124 mg, 1 mmol, 1 equiv.), catalyst [Cp*Ru(PhCl)]BF_4 (43 mg, 0.1 mmol, 0.1 equiv.), aryl trialkoxysilanes **4f** (302 mg, 1.4 mmol, 1.4 equiv.), CsF (46 mg, 0.3 mmol, 0.3 equiv.), DABCO (135 mg, 1.2 mmol, 1.2 equiv.), ZrN (420 mg, 4 mmol, 4 equiv.), and 0.3 mL of 1,4-dioxane. The purification was accomplished by column chromatography on silica gel to provide the desired product **2m** (174 mg, 0.86 mmol, 86%).

Brown liquid. **^{1}H NMR** (500 MHz, *$CDCl_3$*): δ = 1.88 (s, 1H, OH), 4.75 (d, 2H, ^4J = 4.1 Hz, CH_2), 7.02 – 7.07 (m, 3H, CH_{Ar}), 7.72 – 7.80 (m, 2H, CH_{Ar}), 7.28 – 7.31 (m, 1H, CH_{Ar}), 7.36 – 7.40 (m, 2H, CH_{Ar}), 7.41 – 7.46 (m, 2H, CH_{Ar}), 7.50 – 7.51 (m, 1H, CH_{Ar}), 7.58 (s, 1H, CH_{Ar}).

$^{13}C\{^1H\}$ NMR (126 MHz, *$CDCl_3$*): δ= 65.2, 113.9 (d, J_{CF} = 13.5 Hz), 114.1 (d, J_{CF} = 12.3 Hz), 122.7 (d, J_{CF} = 12.8Hz), 125.7, 126.4, 129.1, 130.1 (d, J_{CF} = 8.1 Hz), 140.2, 141.5, 143.2 (d, J_{CF} = 7.9 Hz), 163.2 (d, $^1J_{CF}$ = 246.9 Hz).

MS (GC, 70eV): m/z (%) = 202 (M^+, 100), 183 (29), 173 (24), 152 (31) 107 (13).

Anal. calcd. for $C_{13}H_{11}NO$: C, 77.21; H, 5.48. Found: C, 77.32; H, 5.39.

[1,1'-biphenyl]-4,4'-diyldimethanol 2n

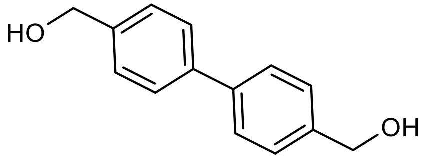

The title compound was prepared starting from phenol **1n** (124 mg, 1 mmol, 1 equiv.), catalyst [Cp*Ru(PhCl)]BF_4 (43 mg, 0.1 mmol, 0.1 equiv.), boronic acid **3m** (213 mg, 1.4 mmol, 1.4 equiv.), KF (17 mg, 0.3 mmol, 0.3 equiv.), DABCO (135 mg, 1.2 mmol, 1.2 equiv.), ZrN (420 mg, 4 mmol, 4 equiv.), and 0.3 mL of 1,4-dioxane. The purification was accomplished by column chromatography on silica gel to provide the desired product **2n** (175 mg, 0.82 mmol, 82%). Yellowish solid, mp 194 - 195 °C. **^1H NMR** (500 MHz, DMSO-*d_6*): δ = 4.55 (br. s, 4H, 2xCH_2), 5.26 (br. s, 2H, 2xOH), 7.40 (d, 4H, ^3J = 7.9 Hz, CH_{Ar}), 7.61 (d, 4H, ^3J = 7.9 Hz, CH_{Ar}).

$^{13}C\{^1H\}$ NMR (126 MHz, DMSO-*d_6*): δ= 62.7, 126.3, 127.1, 138.5, 141.6.

MS (GC, 70eV): m/z (%) = 214 (M^+, 100), 183 (21), 165 (29), 155 (75) 152 (22).

Anal. calcd. for $C_{14}H_{14}O_2$: C, 78.48; H, 6.59. Found: C, 78.51; H, 6.63.

4-methoxy-1,1'-biphenyl 2o

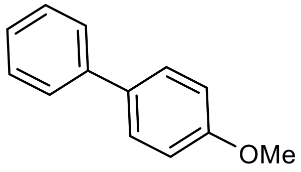

The title compound was prepared starting from phenol **1o** (124 mg, 1 mmol, 1 equiv.), catalyst [Cp*Ru(PhCl)]BF_4 (43 mg, 0.1 mmol, 0.1 equiv.), boronic acid **3a** (171 mg, 1.4 mmol, 1.4 equiv.), KF (17 mg, 0.3 mmol, 0.3 equiv.), DABCO (135 mg, 1.2 mmol, 1.2 equiv.), ZrN (420 mg, 4 mmol, 4 equiv.), and 0.3 mL of 1,4-dioxane. The purification was accomplished by column chromatography on silica gel to provide the desired product **2o** (143 mg, 0.78 mmol, 78%).

Alternatively the title compound was prepared starting from phenol **1o** (124 mg, 1 mmol, 1 equiv.), catalyst [Cp*Ru(PhCl)]BF_4 (43 mg, 0.1 mmol, 0.1 equiv.), aryl trialkoxysilanes **4a** (277 mg, 1.4 mmol, 1.4 equiv.), CsF (46 mg, 0.3 mmol, 0.3 equiv.), DABCO (135 mg, 1.2 mmol, 1.2 equiv.), ZrN (420 mg, 4 mmol, 4 equiv.), and 0.3 mL of 1,4-dioxane. The purification was accomplished by column chromatography on silica gel to provide the desired product **2o** (136 mg, 0.74 mmol, 74%).

Alternatively the title compound was prepared starting from phenol **1a** (94 mg, 1 mmol, 1 equiv.), catalyst [Cp*Ru(PhCl)]BF_4 (43 mg, 0.1 mmol, 0.1 equiv.), boronic acid **3i** (213 mg, 1.4 mmol, 1.4 equiv.), KF (17 mg, 0.3 mmol, 0.3 equiv.), DABCO (135 mg, 1.2 mmol, 1.2 equiv.), ZrN (420 mg, 4 mmol, 4 equiv.), and 0.3 mL of 1,4-dioxane. The purification was accomplished by column chromatography on silica gel to provide the desired product **2o** (140 mg, 0.76 mmol, 76%).

The title compound was prepared starting from phenol **1a** (94 mg, 1 mmol, 1 equiv.), catalyst [Cp*Ru(PhCl)]BF_4 (43 mg, 0.1 mmol, 0.1 equiv.), aryl trialkoxysilanes **4d** (39 mg, 1.4 mmol, 1.4 equiv.), CsF (46 mg, 0.3 mmol, 0.3 equiv.), DABCO (135 mg, 1.2 mmol, 1.2 equiv.), ZrN (420 mg, 4 mmol, 4 equiv.), and 0.3 mL of 1,4-dioxane. The purification was accomplished by column chromatography on silica gel to provide the desired product **2o** (132 mg, 0.72 mmol, 72%).

White solid, mp 88 - 89 ^{o}C. **^{1}H NMR** (500 MHz, $CDCl_3$): δ = 3.88 (s, 3H, OMe), 7.03 (d, 2H, ^{3}J = 8.7 Hz, CH_{Ar}), 7.36 (dt, 1H, ^{3}J = 7.6 Hz, ^{4}J = 1.0 Hz, CH_{Ar}), 7.47 (t, 2H, ^{3}J = 7.7 Hz, CH_{Ar}), 7.58 – 7.62 (m, 4H, CH_{Ar}).

^{13}C{^{1}H} NMR (126 MHz, $CDCl_3$): δ= 55.3, 114.2, 126.6, 128.1, 128.7, 133.7, 140.8, 159.1.

MS (GC, 70eV): m/z (%) = 184 (M^+, 100), 169 (45), 141 (46), 115 (75).

Anal. calcd. for $C_{13}H_{12}O$: C, 84.75; H, 6.57. Found: C, 84.66; H, 6.68.

1-(4'-bromo-[1,1'-biphenyl]-4-yl)ethan-1-one 2p

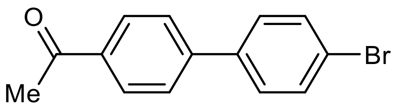

The title compound was prepared starting from phenol **1p** (136 mg, 1 mmol, 1 equiv.), catalyst [Cp*Ru(PhCl)]BF_4 (43 mg, 0.1 mmol, 0.1 equiv.), boronic acid **3n** (280 mg, 1.4 mmol, 1.4 equiv.), KF (17 mg, 0.3 mmol, 0.3 equiv.), DABCO (135 mg, 1.2 mmol, 1.2 equiv.), ZrN (420 mg, 4 mmol, 4 equiv.), and 0.3 mL of 1,4-dioxane. The purification was accomplished by column chromatography on silica gel to provide the desired product **2p** (220 mg, 0.80 mmol, 80%).

Greenish solid, mp 125 - 126 °C. **^{1}H NMR** (500 MHz, *$CDCl_3$*): δ = 2.63 (s, 3H, Me), 7.48 (dt, 2H, ^3J = 8.6 Hz, ^4J = 2.0 Hz, CH_{Ar}), 7.58 (dt, 2H, ^3J = 8.4 Hz, ^4J = 1.8 Hz, CH_{Ar}), 7.63 (dt, 2H, ^3J = 8.3 Hz, ^4J = 1.6 Hz, CH_{Ar}), 8.02 (dt, 2H, ^3J = 8.3 Hz, ^4J = 1.6 Hz, CH_{Ar}).

^{13}C{^{1}H} NMR (126 MHz, *$CDCl_3$*): δ= 26.6, 122.6, 127.0, 128.8, 129.0, 132.1, 136.1, 138.7, 144.5, 197.5.

MS (GC, 70eV): m/z (%) = 276 (M^+, 41), 274 (42), 260 (80), 152 (100).

Anal. calcd. for $C_{12}H_9NO_2$: C, 72.35; H, 4.55; N, 7.03. Found: C, 72.49; H, 4.39; N, 7.11.

N-benzyl-[1,1'-biphenyl]-4-carboxamide 2q

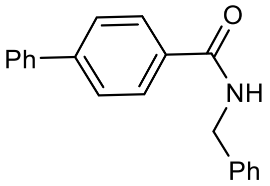

The title compound was prepared starting from phenol **1q** (227 mg, 1 mmol, 1 equiv.), catalyst [Cp*Ru(PhCl)]BF_4 (43 mg, 0.1 mmol, 0.1 equiv.), boronic acid **3a** (171 mg, 1.4 mmol, 1.4 equiv.), KF (17 mg, 0.3 mmol, 0.3 equiv.), DABCO (135 mg, 1.2 mmol, 1.2 equiv.), ZrN (420 mg, 4 mmol, 4 equiv.), and 0.3 mL of 1,4-dioxane. The purification was accomplished by column chromatography on silica gel to provide the desired product **2q** (244 mg, 0.85 mmol, 85%).

The gram scale synthesis was performed on 10 mmol of the starting **1q** and **2q** was prepared in 80% yield (2.30 g, 8.0 mmol).

Alternatively the title compound was prepared starting from phenol **1q** (227 mg, 1 mmol, 1 equiv.), catalyst [Cp*Ru(PhCl)]BF_4 (43 mg, 0.1 mmol, 0.1 equiv.), aryl trialkoxysilanes **4a** (277 mg, 1.4 mmol, 1.4 equiv.), CsF (46 mg, 0.3 mmol, 0.3 equiv.), DABCO (135 mg, 1.2 mmol, 1.2 equiv.), ZrN (420 mg, 4 mmol, 4 equiv.),

and 0.3 mL of 1,4-dioxane. The purification was accomplished by column chromatography on silica gel to provide the desired product **2q** (241 mg, 0.84 mmol, 84%). The gram scale synthesis was performed on 10 mmol of the starting **1q** and **2q** was prepared in 81% yield (2.32 g, 8.1 mmol).

White solid, mp 181 - 182 °C. **^{1}H NMR** (500 MHz, DMSO-*d$_6$*): δ = 4.49 (d, 2H, ^3J = 6.0 Hz, CH_2), 7.19 – 7.22 (m, 1H, CH_{Ar}), 7.28 – 7.32 (m, 4H, CH_{Ar}), 7.35 – 7.38 (m, 1H, CH_{Ar}), 7.45 (t, 2H, ^3J = 7.1 Hz CH_{Ar}), 7.69 (d, 2H, ^3J = 7.3 Hz CH_{Ar}), 7.75 (d, 2H, ^3J = 8.6 Hz, CH_{Ar}), 7.75 (d, 2H, ^3J = 8.3 Hz, CH_{Ar}), 9.11 (t, 1H, ^3J = 7.0 Hz, NH).

^{13}C{^{1}H} NMR (126 MHz, DMSO-*d$_6$*): δ= 42.6, 126.6, 126.7, 126.9, 127.2, 127.9, 128.0, 128.3, 129.0, 133.1, 139.2, 139.7, 142.8.

MS (GC, 70eV): m/z (%) = 287 (M^+, 21), 181 (100), 152 (51).

Anal. calcd. for $C_{20}H_{17}NO$: C, 83.59; H, 5.96; N, 4.87. Found: C, 83.63; H, 6.03; N, 4.71.

[1,1'-biphenyl]-4-yl(azepan-1-yl)methanone 2r

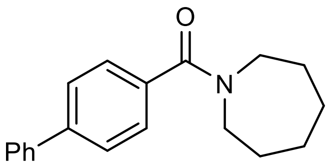

The title compound was prepared starting from phenol **1r** (219 mg, 1 mmol, 1 equiv.), catalyst [Cp*Ru(PhCl)]BF_4 (43 mg, 0.1 mmol, 0.1 equiv.), boronic acid **3a** (171 mg, 1.4 mmol, 1.4 equiv.), KF (17 mg, 0.3 mmol, 0.3 equiv.), DABCO (135 mg, 1.2 mmol, 1.2 equiv.), ZrN (420 mg, 4 mmol, 4 equiv.), and 0.3 mL of 1,4-dioxane. The purification was accomplished by column chromatography on silica gel to provide the desired product **2r** (223 mg, 0.80 mmol, 80%).

Yellowish solid, mp 87 - 88 °C. **^{1}H NMR** (500 MHz, *$CDCl_3$*): δ = 1.60 – 1.64 (m, 6H, CH_2), 1.84 – 1.85 (m, 2H, CH_2), 3.42 (t, 2H, ^3J = 5.8 Hz, CH_2), 3.69 (t, 2H, ^3J = 5.8 Hz, CH_2), 7.35 (t, 1H, ^3J = 7.3 Hz, CH_{Ar}), 7.42 – 7.46 (m, 4H, CH_{Ar}), 7.60 (t, 4H, ^3J = 9.6 Hz CH_{Ar}).

^{13}C{^{1}H} NMR (126 MHz, *$CDCl_3$*): δ= 26.3, 27.1, 27.7, 29.4, 46.2, 49.6, 126.8, 126.9, 127.0, 127.5, 128.7, 136.0, 140.2, 141.8, 171.3.

MS (GC, 70eV): m/z (%) = 279 (M^+, 88), 181 (100), 152 (46).

Anal. calcd. for $C_{19}H_{21}NO$: C, 81.68; H, 7.58; N, 5.01. Found: C, 81.73; H, 7.59; N, 5.03.

2-([1,1'-biphenyl]-4-ylmethyl)-1H-benzo[d]imidazole 2s

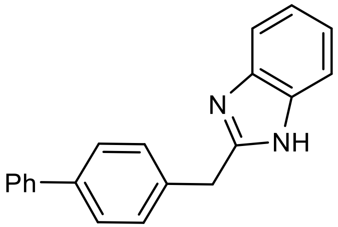

The title compound was prepared starting from phenol **1s** (224 mg, 1 mmol, 1 equiv.), catalyst [Cp*Ru(PhCl)]BF_4 (43 mg, 0.1 mmol, 0.1 equiv.), boronic acid **3a** (171 mg, 1.4 mmol, 1.4 equiv.), KF (17 mg, 0.3 mmol, 0.3 equiv.), DABCO (135 mg, 1.2 mmol, 1.2 equiv.), ZrN (420 mg, 4 mmol, 4 equiv.), and 0.3 mL of 1,4-dioxane. The purification was accomplished by column chromatography on silica gel to provide the desired product **2s** (241 mg, 0.85 mmol, 85%).

Alternatively the title compound was prepared starting from phenol **1s** (224 mg, 1 mmol, 1 equiv.), catalyst [Cp*Ru(PhCl)]BF_4 (43 mg, 0.1 mmol, 0.1 equiv.), aryl trialkoxysilanes **4a** (277 mg, 1.4 mmol, 1.4 equiv.), CsF (46 mg, 0.3 mmol, 0.3 equiv.), DABCO (135 mg, 1.2 mmol, 1.2 equiv.), ZrN (420 mg, 4 mmol, 4 equiv.), and 0.3 mL of 1,4-dioxane. The purification was accomplished by column chromatography on silica gel to provide the desired product **2s** (244 mg, 0.86 mmol, 86%).

White solid, mp 189 - 191 °C. **^{1}H NMR** (500 MHz, DMSO-d_6): δ = 4.21 (s, 2H, CH_2), 7.11 – 7.13 (m, 2H, CH_{Ar}), 7.32 (t, 1H, ^3J = 7.3 Hz CH_{Ar}), 7.41 – 7.46 (m, 6H, CH_{Ar}), 7.60 – 7.64 (m, 4H, CH_{Ar}), 12.33 (br. s, 1H, NH).

^{13}C{^{1}H} NMR (126 MHz, DMSO-d_6): δ= 34.8, 121.3, 126.5, 126.8, 127.4, 129.0, 129.4, 136.9, 138.5, 140.0, 153.5.

MS (GC, 70eV): m/z (%) = 284 (M^+, 100), 165 (22), 152 (46).

Anal. calcd. for $C_{20}H_{16}N_2$: C, 84.48; H, 5.67; N, 9.85. Found: C, 84.31; H, 5.59; N, 10.1.

tert-butyl (R)-(1-([1,1'-biphenyl]-4-yl)-3-hydroxypropan-2-yl)carbamate 2t

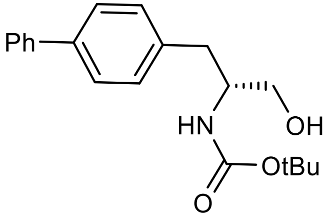

The title compound was prepared starting from phenol **1t** (267 mg, 1 mmol, 1 equiv.), [Cp*Ru(PhCl)]BF_4 (43 mg, 0.1 mmol, 0.1 equiv.), boronic acid **3a** (171 mg, 1.4 mmol, 1.4 equiv.), KF (17 mg, 0.3 mmol, 0.3 equiv.), DABCO (135 mg, 1.2 mmol, 1.2 equiv.), ZrN (420 mg, 4 mmol, 4 equiv.), and 0.3 mL of 1,4-dioxane. The purification was accomplished by column chromatography on silica gel to provide the desired product **2t** (261 mg, 0.80 mmol, 80%).

Alternatively the title compound was prepared starting from phenol **1t** (267 mg, 1 mmol, 1 equiv.), catalyst [Cp*Ru(PhCl)]BF_4 (43 mg, 0.1 mmol, 0.1 equiv.), aryl trialkoxysilanes **4a** (277 mg, 1.4 mmol, 1.4 equiv.), CsF (46 mg, 0.3 mmol, 0.3 equiv.), DABCO (135 mg, 1.2 mmol, 1.2 equiv.), ZrN (420 mg, 4 mmol, 4 equiv.), and 0.3 mL of 1,4-dioxane. The purification was accomplished by column chromatography on silica gel to provide the desired product **2t** (268 mg, 0.82 mmol, 82%).

White solid, mp 154 - 156 ^oC. **^{1}H NMR** (500 MHz, *CDCl₃*): δ = 1.44 (s, 9H, *t*Bu), 2.91 – 2.92 (m, 2H, CH_2), 3.15 – 3.16 (m, 1H, CH), 3.60 – 3.62 (m, 1H, CH_2), 3.69 – 3.71 (m, 1H, CH_2), 3.95 (br. s, 1H, NH), 5.01 (br. s, 1H, OH), 7.31 – 7.37 (m, 3H, CH_{Ar}), 7.44 (t, 2H, ^3J = 7.8 Hz, CH_{Ar}), 7.54 (d, 2H, ^3J = 7.8 Hz, CH_{Ar}), 7.59 (d, 2H, ^3J = 7.4 Hz, CH_{Ar}).

^{13}C{^{1}H} NMR (126 MHz, *$CDCl_3$*): δ= 28.3, 37.0, 53.6, 63.9, 79.6, 126.9, 127.1, 128.7, 129.7, 137.0, 139.3, 140.8., 156.1.

Anal. calcd. for $C_{20}H_{25}NO_3$: C, 73.37; H, 7.70; N, 4.28. Found: C, 73.41; H, 7.72; N, 4.21.

4'-((1s,4r)-4-pentylcyclohexyl)-[1,1'-biphenyl]-4-carbonitrile 5u

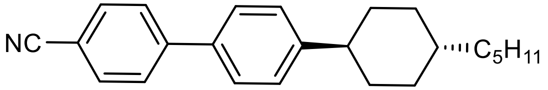

The title compound was prepared starting from phenol **1u** (246 mg, 1 mmol, 1 equiv.), catalyst [Cp*Ru(PhCl)]BF_4 (43 mg, 0.1 mmol, 0.1 equiv.), boronic acid **3o** (206 mg, 1.4 mmol, 1.4 equiv.), KF (17 mg, 0.3 mmol, 0.3 equiv.), DABCO (135 mg, 1.2 mmol, 1.2 equiv.), ZrN (420 mg, 4 mmol, 4 equiv.), and 0.3 mL of 1,4-dioxane. The purification was accomplished by column chromatography on silica gel to provide the desired product **2u** (241 mg, 0.76 mmol, 76%).

White solid, mp 95 - 96 ^oC. **^{1}H NMR** (500 MHz, *$CDCl_3$*): δ = 0.93 (t, 3H, ^3J = 7.4 Hz, CH_3), 1.05 – 1.14 (m, 2H, Aliph.), 1.23 – 1.38 (m, 9H, Aliph.), 1.46 – 1.55 (m, 2H, Aliph.), 1.93 (t, 4H, ^3J = 12.6 Hz, Aliph.), 2.51 – 2.57 (m, 1H, Aliph.), 7.33 (d, 2H, ^3J = 8.5 Hz, CH_{Ar}), 7.53 (d, 2H, ^3J = 8.5 Hz, CH_{Ar}), 7.66 – 7.71 (m, 4H, CH_{Ar}).

^{13}C{^{1}H} NMR (126 MHz, *$CDCl_3$*): δ= 14.1, 22.7, 26.6, 32.2, 33.5, 34.2, 37.2, 37.3, 44.3, 110.4, 118.9, 127.0, 127.4, 127.5, 132.5, 136.5, 145.5, 148.6.

Anal. calcd. for $C_{24}H_{29}N$: C, 86.96; H, 8.82; N, 4.23. Found: C, 87.03; H, 8.69; N, 4.28.

2-phenyl-1H-indole 2v

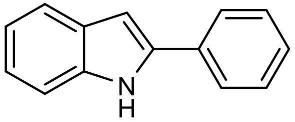

The title compound was prepared starting from phenol **1v** (133 mg, 1 mmol, 1 equiv.), catalyst [Cp*Ru(PhCl)]BF_4 (43 mg, 0.1 mmol, 0.1 equiv.), boronic acid **3a** (171 mg, 1.4 mmol, 1.4 equiv.), KF (17 mg, 0.3 mmol, 0.3 equiv.), DABCO (135 mg, 1.2 mmol, 1.2 equiv.), ZrN (420 mg, 4 mmol, 4 equiv.), and 0.3 mL of 1,4-dioxane. The purification was accomplished by column chromatography on silica gel to provide the desired product **2v** (162 mg, 0.84 mmol, 84%).

Alternatively the title compound was prepared starting from phenol **1v** (133 mg, 1 mmol, 1 equiv.), catalyst [Cp*Ru(PhCl)]BF_4 (43 mg, 0.1 mmol, 0.1 equiv.), aryl trialkoxysilanes **4a** (277 mg, 1.4 mmol, 1.4 equiv.), CsF (46 mg, 0.3 mmol, 0.3 equiv.), DABCO (135 mg, 1.2 mmol, 1.2 equiv.), ZrN (420 mg, 4 mmol, 4 equiv.), and 0.3 mL of 1,4-dioxane. The purification was accomplished by column chromatography on silica gel to provide the desired product **2v** (154 mg, 0.80 mmol, 80%).

White solid, mp 188 - 189 °C. **^{1}H NMR** (500 MHz, DMSO-d_6): δ = 6.90 (d, 1H, ^4J = 1.3 Hz, CH_{Ar}), 7.02 (dt, 1H, ^3J = 7.9 Hz, ^4J = 0.9 Hz, CH_{Ar}), 7.12 (dt, 1H, ^3J = 6.9 Hz, ^4J = 1.3 Hz, CH_{Ar}), 7.31 (t, 1H, ^3J = 7.3 Hz CH_{Ar}), 7.44 – 7.47 (m, 3H, CH_{Ar}), 7.54 (d, 1H, ^3J = 7.9 Hz, CH_{Ar}), 7.88 (dd, 2H, ^3J = 7.9 Hz, ^4J = 1.3 Hz, CH_{Ar}), 11.58 (s, 1H, NH).

^{13}C{^{1}H} NMR (126 MHz, DMSO-d_6): δ= 98.7, 111.4, 119.4, 120.1, 121.6, 125.0, 127.4, 128.7, 128.9, 132.3, 137.2, 137.6.

MS (GC, 70eV): m/z (%) = 193 (M^+, 100), 165 (25).

Anal. calcd. for $C_{14}H_{11}N$: C, 87.01; H, 5.74; N, 7.25. Found: C, 87.13; H, 5.79; N, 7.08.

3-([1,1'-biphenyl]-4-carbonyl)-4H-chromen-4-one 2w

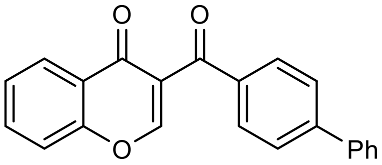

The title compound was prepared starting from phenol **1w** (266 mg, 1 mmol, 1 equiv.), catalyst [Cp*Ru(PhCl)]BF_4 (43 mg, 0.1 mmol, 0.1 equiv.), boronic acid **3a** (171 mg, 1.4 mmol, 1.4 equiv.), KF (17 mg, 0.3 mmol, 0.3 equiv.), DABCO (135 mg, 1.2 mmol, 1.2 equiv.), ZrN (420 mg, 4 mmol, 4 equiv.), and 0.3 mL of 1,4-dioxane. The purification was accomplished by column chromatography on silica gel to provide the desired product **2w** (261 mg, 0.80 mmol, 80%). Alternatively the title compound was prepared starting from phenol **1w** (266 mg, 1 mmol, 1 equiv.), catalyst [Cp*Ru(PhCl)]BF_4 (43 mg, 0.1 mmol, 0.1 equiv.), aryl trialkoxysilanes **4a** (277 mg, 1.4 mmol, 1.4 equiv.), CsF (46 mg, 0.3 mmol, 0.3 equiv.), DABCO (135 mg, 1.2 mmol, 1.2 equiv.), ZrN (420 mg, 4 mmol, 4 equiv.), and 0.3 mL of 1,4-dioxane. The purification was accomplished by column chromatography on silica gel to provide the desired product **2w** (244 mg, 0.75 mmol, 75%).

Yellow solid, mp 179 - 181 °C. **^{1}H NMR** (500 MHz, *$CDCl_3$*): δ = 7.38 – 7.41 (m, 1H, CH_{Ar}), 7.45 – 7.51 (m, 3H, CH_{Ar}), 7.55 (d, 1H, ^3J = 8.6 Hz, CH_{Ar}), 7.62 – 7.64 (m, 2H, CH_{Ar}), 7.68 (d, 2H, ^3J = 8.3 Hz, CH_{Ar}), 7.74 – 7.77 (m, 1H, CH_{Ar}), 7.94 (d, 2H, ^3J = 8.1 Hz, CH_{Ar}), 8.28 (dd, 1H, ^3J = 7.8 Hz, ^4J = 1.6 Hz, CH_{Ar}), 8.33 (s, 1H, CH_{Ar}).

^{13}C{^{1}H} NMR (126 MHz, *$CDCl_3$*): δ= 118.3, 125.0, 125.3, 126.1, 126.5, 127.1, 127.3, 128.2, 128.9, 130.2, 134.4, 135.8, 139.9, 146.2, 156.1, 158.6, 174.8, 191.4.

MS (GC, 70eV): m/z (%) = 326 (M^+, 91), 298 (100), 181 (25), 152 (79), 121 (33).

Anal. calcd. for $C_{22}H_{14}O_3$: C, 80.97; H, 4.32. Found: C, 81.03; H, 4.28.

(E)-1,2-diphenylethen 2x

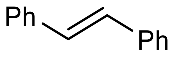

The title compound was prepared starting from phenol **1a** (94 mg, 1 mmol, 1 equiv.), catalyst [Cp*Ru(PhCl)]BF_4 (43 mg, 0.1 mmol, 0.1 equiv.), boronic acid **3r** (207 mg, 1.4 mmol, 1.4 equiv.), KF (17 mg, 0.3 mmol, 0.3 equiv.), DABCO (135 mg, 1.2 mmol, 1.2 equiv.), ZrN (420 mg, 4 mmol, 4 equiv.), and 0.3 mL of 1,4-dioxane. The purification was accomplished by column chromatography on silica gel to provide the desired product **2x** (92 mg, 0.51 mmol, 51%).

Beige solid, mp 123 - 125 °C. **^{1}H NMR** (500 MHz, *$CDCl_3$*): δ = 7.16 (s, 2H, CH_{Ar}), 7.31 (t, 2H, ^3J = 7.5 Hz, CH_{Ar}), 7.41 (t, 4H, ^3J = 7.5 Hz, CH_{Ar}), 7.56 (d, 4H, ^3J = 7.5 Hz, CH_{Ar}).

^{13}C{^{1}H} NMR (126 MHz, *$CDCl_3$*): δ= 126.5, 127.6, 128.7, 137.3.

Anal. calcd. for $C_{14}H_{12}$: C, 93.29; H, 6.71. Found: C, 93.19; H, 6.81.

1,2-diphenylethyne 2y

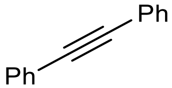

The title compound was prepared starting from phenol **1a** (94 mg, 1 mmol, 1 equiv.), [Cp*Ru(PhCl)]BF_4 (43 mg, 0.1 mmol, 0.1 equiv.), aryl trialkoxysilanes **4g** (311 mg, 1.4 mmol, 1.4 equiv.), CsF (46 mg, 0.3 mmol, 0.3 equiv.), DABCO (135 mg, 1.2 mmol, 1.2 equiv.), ZrN (420 mg, 4 mmol, 4 equiv.), and 0.3 mL of 1,4-dioxane. The purification was accomplished by column chromatography on silica gel to provide the desired product **2y** (121 mg, 0.68 mmol, 68%).

Yellow solid, mp 60 - 61 ^{o}C. **^{1}H NMR** (500 MHz, *$CDCl_3$*): δ = 7.38 – 7.41 (m, 6H, CH_{Ar}), 7.60 – 7.62 (m, 4H, CH_{Ar}).

^{13}C{^{1}H} NMR (126 MHz, *$CDCl_3$*): δ= 123.2, 128.2, 128.3, 131.6.

Anal. calcd. for $C_{14}H_{10}$: C, 94.34; H, 5.66. Found: C, 94.31; H, 5.69.

1-chloro-4-ethynylbenzene 2z

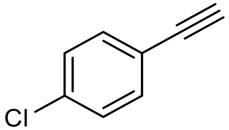

The title compound was prepared starting from phenol **1ae** (128 mg, 1 mmol, 1 equiv.), catalyst [Cp*Ru(PhCl)]BF_4 (43 mg, 0.1 mmol, 0.1 equiv.), aryl trialkoxysilanes **4h** (204 mg, 1.4 mmol, 1.4 equiv.), CsF (46 mg, 0.3 mmol, 0.3 equiv.), DABCO (135 mg, 1.2 mmol, 1.2 equiv.), ZrN (420 mg, 4 mmol, 4 equiv.), and 0.3 mL of 1,4-dioxane. The purification was accomplished by column chromatography on silica gel to provide the desired product **2z** (84 mg, 0.62 mmol, 62%).

Yellow liquid, mp 60 - 61 ^{o}C. **^{1}H NMR** (500 MHz, *$CDCl_3$*): δ = 3.11 (s, 1H, CH), 7.29 – 7.31 (m, 2H, CH_{Ar}), 7.41 – 7.43 (m, 2H, CH_{Ar}).

^{13}C{^{1}H} NMR (126 MHz, *$CDCl_3$*): δ= 78.2, 82.5, 120.6, 128.7, 133.3, 134.9.

Anal. calcd. for C_8H_5Cl: C, 70.35; H, 3.69. Found: C, 70.42; H, 3.59.

(4-fluorophenyl)(phenyl)methanone 6a

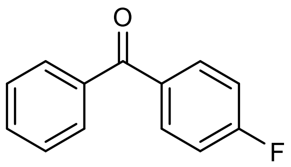

The title compound was prepared starting from phenol **1a** (94 mg, 1 mmol, 1 equiv.), catalyst [Cp*Ru(Napht)]BF_4 (44 mg, 0.1 mmol, 0.1 equiv.), benzoyltrifluoroborate **5b** (322 mg, 1.4 mmol, 1.4 equiv.), KF (17 mg, 0.3 mmol, 0.3 equiv.), DABCO (135 mg, 1.2 mmol, 1.2 equiv.), ZrN (210 mg, 2 mmol, 2 equiv.), $BaTiO_3$ (466 mg, 2 mmol, 2 equiv.) and 0.3 mL of 1,4-dioxane. The purification was accomplished by column chromatography on silica gel to provide the desired product **6a** (176 mg, 0.88 mmol, 88%). The gram scale synthesis was performed on 10 mmol of the starting **1a** and **6a** was prepared in 77% yield (1.54 g, 7.7 mmol).

Alternatively the title compound was prepared starting from phenol **1x** (112 mg, 1 mmol, 1 equiv.), catalyst [Cp*Ru(Napht)]BF_4 (44 mg, 0.1 mmol, 0.1 equiv.), benzoyltrifluoroborate **5a** (297 mg, 1.4 mmol, 1.4 equiv.), KF (17 mg, 0.3 mmol, 0.3 equiv.), DABCO (135 mg, 1.2 mmol, 1.2 equiv.), ZrN (210 mg, 2 mmol, 2 equiv.), $BaTiO_3$ (466 mg, 2 mmol, 2 equiv.) and 0.3 mL of 1,4-dioxane. The purification was accomplished by column chromatography on silica gel to provide the desired product **6a** (179 mg, 0.89 mmol, 89%).

Beige solid, mp 48 - 50 °C. **^{1}H NMR** (500 MHz, $CDCl_3$): δ = 7.16 (tt, 2H, ^3J = 8.7 Hz, ^4J = 1.8 Hz, CH_{Ar}), 7.49 (t, 2H, ^3J = 7.6 Hz, CH_{Ar}), 7.60 (tt, 1H, ^3J = 7.4 Hz, ^4J = 1.8 Hz, CH_{Ar}), 7.76 – 7.80 (m, 2H, CH_{Ar}), 7.83 – 7.87 (m, 2H, CH_{Ar}).

^{13}C{^{1}H} NMR (126 MHz, *$CDCl_3$*): δ= 115.4 (d, J_{CF} = 21.7 Hz), 128.4, 129.9, 132.5, 132.7 (d, J_{CF} = 8.7 Hz), 133.8 (d, J_{CF} = 7.8 Hz), 142.6 (d, J_{CF} = 2.3 Hz), 137.5, 165.4 (d, $^1J_{CF}$ = 258.4 Hz), 195.2.

MS (GC, 70eV): m/z (%) = 200 (M^+, 94), 123 (100), 105 (72).

Anal. calcd. for $C_{13}H_9FO$: C, 77.99; H, 4.53. Found: C, 78.05; H, 4.59.

(4-fluorophenyl)(4-methoxyphenyl)methanone 6b

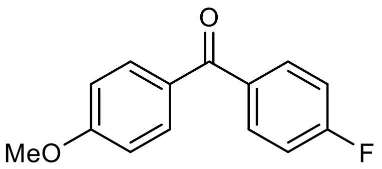

The title compound was prepared starting from phenol **1o** (124 mg, 1 mmol, 1 equiv.), catalyst [Cp*Ru(Napht)]BF_4 (44 mg, 0.1 mmol, 0.1 equiv.), benzoyltrifluoroborate **5b** (322 mg, 1.4 mmol, 1.4 equiv.), KF (17 mg, 0.3 mmol, 0.3 equiv.), DABCO (135 mg, 1.2 mmol, 1.2 equiv.), ZrN (210 mg, 2 mmol, 2 equiv.), $BaTiO_3$ (466 mg, 2 mmol, 2 equiv.) and 0.3 mL of 1,4-dioxane. The purification was accomplished by column chromatography on silica gel to provide the desired product **6a** (193 mg, 0.84 mmol, 84%).

White solid, mp 91 - 93 °C. **^{1}H NMR** (500 MHz, DMSO-d_6): δ = 3.84 (s, 3H, OMe), 7.05 (d, 2H, ^3J = 8.7 Hz, CH_{Ar}), 7.34 (t, 2H, ^3J = 8.7 Hz, CH_{Ar}), 7.72 (dt, 2H, ^3J = 8.7 Hz, ^4J = 1.8 Hz, CH_{Ar}), 7.74 – 7.77 (m, 2H, CH_{Ar}).

^{13}C{^{1}H} NMR (126 MHz, *$CDCl_3$*): δ= 55.5, 113.9, 115.4 (d, J_{CF} = 25.1 Hz), 129.3, 132.1, 132.2, 134.3 (d, J_{CF} = 2.3 Hz), 163.0, 164.4 (d, $^1J_{CF}$ = 252.5 Hz), 193.0.

MS (GC, 70eV): m/z (%) = 230 (M^+, 53), 135 (100), 123 (20).

Anal. calcd. for $C_{14}H_{11}FO_2$: C, 73.03; H, 4.82. Found: C, 73.19; H, 4.91.

(4-fluorophenyl)(4-methoxyphenyl)methanone 6c

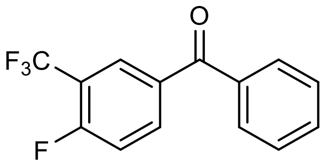

The title compound was prepared starting from phenol **1y** (180 mg, 1 mmol, 1 equiv.), catalyst [Cp*Ru(Napht)]BF_4 (44 mg, 0.1 mmol, 0.1 equiv.), benzoyltrifluoroborate **5a** (297 mg, 1.4 mmol, 1.4 equiv.), KF (17 mg, 0.3 mmol, 0.3 equiv.), DABCO (135 mg, 1.2 mmol, 1.2 equiv.), ZrN (210 mg, 2 mmol, 2 equiv.), $BaTiO_3$ (466 mg, 2 mmol, 2 equiv.) and 0.3 mL of 1,4-dioxane. The purification was accomplished by column chromatography on silica gel to provide the desired product **6c** (212 mg, 0.79 mmol, 79%).

White solid, mp 46 - 48 °C. **^{1}H NMR** (500 MHz, *$CDCl_3$*): δ = 7.33 (t, 1H, ^3J = 9.1 Hz, CH_{Ar}), 7.52 (t, 2H, ^3J = 7.6 Hz, CH_{Ar}), 7.64 (tt, 1H, ^3J = 7.6 Hz, ^4J = 1.3 Hz, CH_{Ar}), 7.76 – 7.78 (m, 2H, CH_{Ar}), 8.01 – 8.05 (m, 1H, CH_{Ar}), 8.11 (dd, 1H, ^3J = 6.7 Hz, ^4J = 1.9 Hz, CH_{Ar}).

^{13}C{^{1}H} NMR (126 MHz, *$CDCl_3$*): δ= 117.1 (d, J_{CF} = 21.6 Hz), 118.8 (dq, $^2J_{CF}$ = 33.4 Hz, J_{CF} = 13.7 Hz), 122.1 (d, $^1J_{CF}$ = 274.1 Hz), 128.7, 129.5 (m), 129.9, 133.1, 133.9 (d, J_{CF} = 3.7 Hz), 135.8 (d, J_{CF} = 9.9 Hz), 136.6, 162.1 (d, $^1J_{CF}$ = 263.9 Hz, J_{CF} = 1.9 Hz), 193.9.

MS (GC, 70eV): m/z (%) = 268 (M^+, 56), 191 (55), 163 (36), 105 (100).

Anal. calcd. for $C_{14}H_8F_4O$: C, 62.69; H, 3.01. Found: C, 62.75; H, 3.19.

phenyl(3,4,5-trifluorophenyl)methanone 6d

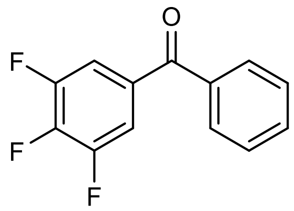

The title compound was prepared starting from phenol **1z** (148 mg, 1 mmol, 1 equiv.), catalyst [Cp*Ru(Napht)]BF_4 (44 mg, 0.1 mmol, 0.1 equiv.), benzoyltrifluoroborate **5a** (297 mg, 1.4 mmol, 1.4 equiv.), KF (17 mg, 0.3 mmol, 0.3 equiv.), DABCO (135 mg, 1.2 mmol, 1.2 equiv.), ZrN (210 mg, 2 mmol, 2 equiv.), $BaTiO_3$ (466 mg, 2 mmol, 2 equiv.) and 0.3 mL of 1,4-dioxane. The purification was accomplished by column chromatography on silica gel to provide the desired product **6d** (193 mg, 0.82 mmol, 82%). The gram scale synthesis was performed on 10 mmol of the starting **1z** and **6d** was prepared in 79% yield (1.86 g, 7.9 mmol).

White solid, mp 78 - 79 °C. **^{1}H NMR** (500 MHz, *$CDCl_3$*): δ = 7.46 – 7.49 (m, 2H, CH_{Ar}), 7.52 (dt, 2H, ^3J = 7.8 Hz, ^4J = 1.5 Hz, CH_{Ar}), 7.64 (tt, 1H, ^3J = 7.4 Hz, ^4J = 1.3 Hz, CH_{Ar}), 7.74 – 7.77 (m, 2H, CH_{Ar}).

^{13}C{^{1}H} NMR (126 MHz, *$CDCl_3$*): δ= 114.5 (dd, J_{CF} = 16.0 Hz, J_{CF} = 6.5 Hz), 128.6, 129.8, 134.0 (m), 133.1, 136.2, 142.7 (dt, $^1J_{CF}$ = 259.3 Hz, J_{CF} = 15.4 Hz), 151.0 (ddd, $^1J_{CF}$ = 253.1 Hz, J_{CF} = 10.4 Hz, J_{CF} = 3.2 Hz), 192.9.

MS (GC, 70eV): m/z (%) = 236 (M^+, 63), 159 (34), 105 (100).

Anal. calcd. for $C_{13}H_7F_3O$: C, 66.11; H, 2.99. Found: C, 66.29; H, 2.81.

(2,5-difluorophenyl)(phenyl)methanone 6e

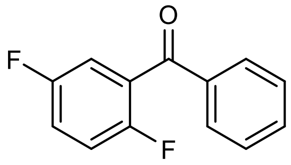

The title compound was prepared starting from phenol **1aa** (130 mg, 1 mmol, 1 equiv.), catalyst [Cp*Ru(Napht)]BF_4 (44 mg, 0.1 mmol, 0.1 equiv.), benzoyltrifluoroborate **5a** (297 mg, 1.4 mmol, 1.4 equiv.), KF (17 mg, 0.3 mmol, 0.3 equiv.), DABCO (135 mg, 1.2 mmol, 1.2 equiv.), ZrN (210 mg, 2 mmol, 2 equiv.), $BaTiO_3$ (466 mg, 2 mmol, 2 equiv.) and 0.3 mL of 1,4-dioxane. The purification was accomplished by column chromatography on silica gel to provide the desired product **6e** (174 mg, 0.80 mmol, 80%).

Colourless liquid **^{1}H NMR** (500 MHz, *$CDCl_3$*): δ = 7.12 (dt, 1H, ^3J = 8.9 Hz, ^4J = 4.1 Hz, CH_{Ar}), 7.18 – 7.25 (m, 2H, CH_{Ar}), 7.47 (t, 2H, ^3J = 7.4 Hz, CH_{Ar}), 7.60 (dt, 1H, ^3J = 7.4 Hz, ^4J = 1.1 Hz, CH_{Ar}), 7.82 (d, 2H, ^3J = 8.3 Hz, CH_{Ar}).

^{13}C{^{1}H} NMR (126 MHz, *$CDCl_3$*): δ= 116.9 (dd, J_{CF} = 25.0 Hz, J_{CF} = 3.5 Hz), 117.5 (dd, J_{CF} = 25.1 Hz, J_{CF} = 8.4 Hz), 119.5 (dd, J_{CF} = 24.4 Hz, J_{CF} = 8.4 Hz), 127.9 (dd, J_{CF} = 17.7 Hz, J_{CF} = 6.4 Hz), 128.5, 129.7, 133.7, 136.7, 155.8 (dd, $^1J_{CF}$ = 246.9 Hz, J_{CF} = 1.4 Hz), 158.3 (dd, $^1J_{CF}$ = 245.0 Hz, J_{CF} = 1.4 Hz), 191.9.

MS (GC, 70eV): m/z (%) = 218 (M^+, 64), 141 (33), 113 (26), 105 (100).

Anal. calcd. for $C_{13}H_8F_2O$: C, 71.56; H, 3.70. Found: C, 71.69; H, 3.79.

(2,5-dichlorophenyl)(phenyl)methanone 6f

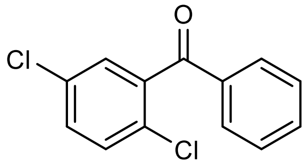

The title compound was prepared starting from phenol **1ab** (163 mg, 1 mmol, 1 equiv.), catalyst [Cp*Ru(Napht)]BF_4 (44 mg, 0.1 mmol, 0.1 equiv.), benzoyltrifluoroborate **5a** (297 mg, 1.4 mmol, 1.4 equiv.), KF (17 mg, 0.3 mmol, 0.3 equiv.), DABCO (135 mg, 1.2 mmol, 1.2 equiv.), ZrN (210 mg, 2 mmol, 2 equiv.), $BaTiO_3$ (466 mg, 2 mmol, 2 equiv.) and 0.3 mL of 1,4-dioxane. The purification was accomplished by column chromatography on silica gel to provide the desired product **6f** (226 mg, 0.91 mmol, 91%).

Beige solid, mp 88 - 89 °C. **^{1}H NMR** (500 MHz, *DMSO-d$_6$*): δ = 7.56 (t, 2H, ^3J = 7.1 Hz, CH_{Ar}), 7.64 – 7.67 (m, 3H, CH_{Ar}), 7.70 – 7.76 (m, 3H, CH_{Ar}).

^{13}C{^{1}H} NMR (126 MHz, *DMSO-d$_6$*): δ= 128.4, 128.5, 129.1, 129.7, 131.4, 131.5, 132.2, 134.4, 135.3, 139.7, 193.0.

MS (GC, 70eV): m/z (%) = 250 (M^+, 35), 173 (17), 105 (100).

Anal. calcd. for $C_{13}H_8Cl_2O$: C, 62.18; H, 3.21. Found: C, 62.39; H, 3.13.

phenyl(3-(trifluoromethyl)phenyl)methanone 6g

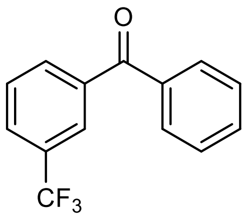

The title compound was prepared starting from phenol **1ac** (162 mg, 1 mmol, 1 equiv.), catalyst [Cp*Ru(Napht)]BF_4 (44 mg, 0.1 mmol, 0.1 equiv.), benzoyltrifluoroborate **5a** (297 mg, 1.4 mmol, 1.4 equiv.), KF (17 mg, 0.3 mmol, 0.3 equiv.), DABCO (135 mg, 1.2 mmol, 1.2 equiv.), ZrN (210 mg, 2 mmol, 2 equiv.), $BaTiO_3$ (466 mg, 2 mmol, 2 equiv.) and 0.3 mL of 1,4-dioxane. The purification was accomplished by column chromatography on silica gel to provide the desired product **6g** (222 mg, 0.89 mmol, 89%).

White solid, mp 53 - 54 °C. **^{1}H NMR** (500 MHz, *CDCl$_3$*): δ = 7.51 (t, 2H, ^3J = 7.4 Hz, CH_{Ar}), 7.62 (t, 2H, ^3J = 7.4 Hz, CH_{Ar}), 7.78 – 7.81 (m, 2H, CH_{Ar}), 7.84 (dd, 1H, ^3J = 7.8 Hz, ^4J = 0.6 Hz, CH_{Ar}), 7.97 (d, 1H, ^3J = 7.7 Hz, CH_{Ar}), 8.07 (s, 1H, CH_{Ar}).

^{13}C{^{1}H} NMR (126 MHz, *CDCl$_3$*): δ= 125.0 (q, $^1J_{CF}$ = 270.0 Hz), 126.6, 128.5, 128.7, 128.9, 130.0, 131.0 (q, $^2J_{CF}$ = 33.0 Hz), 133.0, 136.7, 138.3, 195.1.

Anal. calcd. for $C_{14}H_9F_3O$: C, 67.20; H, 3.63. Found: C, 67.39; H, 3.79.

(3,5-difluorophenyl)(phenyl)methanone 6h

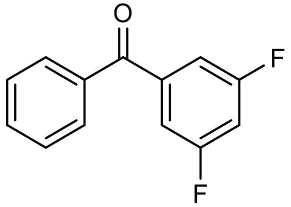

The title compound was prepared starting from phenol **1ad** (130 mg, 1 mmol, 1 equiv.), catalyst [Cp*Ru(Napht)]BF_4 (44 mg, 0.1 mmol, 0.1 equiv.), benzoyltrifluoroborate **5a** (297 mg, 1.4 mmol, 1.4 equiv.), KF (17 mg, 0.3 mmol, 0.3 equiv.), DABCO (135 mg, 1.2 mmol, 1.2 equiv.), ZrN (210 mg, 2 mmol, 2 equiv.), $BaTiO_3$ (466 mg, 2 mmol, 2 equiv.) and 0.3 mL of 1,4-dioxane. The purification was accomplished by column chromatography on silica gel to provide the desired product **6h** (190 mg, 0.87 mmol, 87%).

Yelowish solid, mp 58 - 59 ^oC. **^{1}H NMR** (500 MHz, *$CDCl_3$*): δ = 7.04 (tt, 1H, ^3J = 8.6 Hz, ^4J = 2.3 Hz, CH_{Ar}), 7.30 – 7.32 (m, 2H, CH_{Ar}), 7.51 (t, 2H, ^3J = 7.8 Hz, CH_{Ar}), 7.63 (tt, 1H, ^3J = 7.4 Hz, ^4J = 1.9 Hz, CH_{Ar}), 7.78 – 7.80 (m, 2H, CH_{Ar}).

^{13}C{^{1}H} NMR (126 MHz, *$CDCl_3$*): δ= 107.7 (t, $^2J_{CF}$ = 25.8 Hz), 112.8 (d, J_{CF} = 7.7 Hz), 113.0 (d, J_{CF} = 7.4 Hz), 128.6, 130.0, 133.1, 136.4, 140.6 (d, J_{CF} = 7.8 Hz), 162.7 (dd, $^1J_{CF}$ = 251.3 Hz, $^2J_{CF}$ = 11.4 Hz), 193.9.

MS (GC, 70eV): m/z (%) = 218 (M^+, 60), 141 (20), 113 (21), 105 (100).

Anal. calcd. for $C_{13}H_8F_2O$: C, 71.56; H, 3.70. Found: C, 71.42; H, 3.63.

(3,4-difluorophenyl)(phenyl)methanone 6i

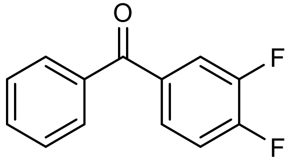

The title compound was prepared starting from phenol **1a** (94 mg, 1 mmol, 1 equiv.), catalyst [Cp*Ru(Napht)]BF_4 (44 mg, 0.1 mmol, 0.1 equiv.), benzoyltrifluoroborate **5c** (347 mg, 1.4 mmol, 1.4 equiv.), KF (17 mg, 0.3 mmol, 0.3 equiv.), DABCO (135 mg, 1.2 mmol, 1.2 equiv.), ZrN (210 mg, 2 mmol, 2 equiv.), $BaTiO_3$ (466 mg, 2 mmol, 2 equiv.) and 0.3 mL of 1,4-dioxane. The purification was accomplished by column chromatography on silica gel to provide the desired product **6h** (185 mg, 0.85 mmol, 85%).

Yellowish solid, mp 54 - 55 °C. **^{1}H NMR** (500 MHz, *CDCl$_3$*): δ = 7.22 – 7.28 (m, 1H, CH_{Ar}), 7.48 (t, 2H, ^3J = 7.7 Hz, CH_{Ar}), 7.55 – 7.60 (m, 2H, CH_{Ar}), 7.64 – 7.69 (m, 1H, CH_{Ar}), 7.73 – 7.75 (m, 1H, CH_{Ar}).

^{13}C{^{1}H} NMR (126 MHz, *CDCl$_3$*): δ= 117.2 (t, J_{CF} = 17.2 Hz), 119.2 (d, J_{CF} = 18.8 Hz), 127.0 (dd, J_{CF} = 7.2 Hz, J_{CF} = 3.8 Hz), 128.5, 129.8, 132.8, 134.4 (m), 136.9, 150.1 (dd, $^1J_{CF}$ = 250.5 Hz, $^2J_{CF}$ = 14.2 Hz), 153.2 (dd, $^1J_{CF}$ = 257.0 Hz, $^2J_{CF}$ = 11.4 Hz), 194.0.

MS (GC, 70eV): m/z (%) = 218 (M^+, 83), 141 (61), 113 (34), 105 (100).

Anal. calcd. for $C_{13}H_8F_2O$: C, 71.56; H, 3.70. Found: C, 71.69; H, 3.78.

(C) Copies ^{1}H and ^{13}C NMR spectra

Compound 2a

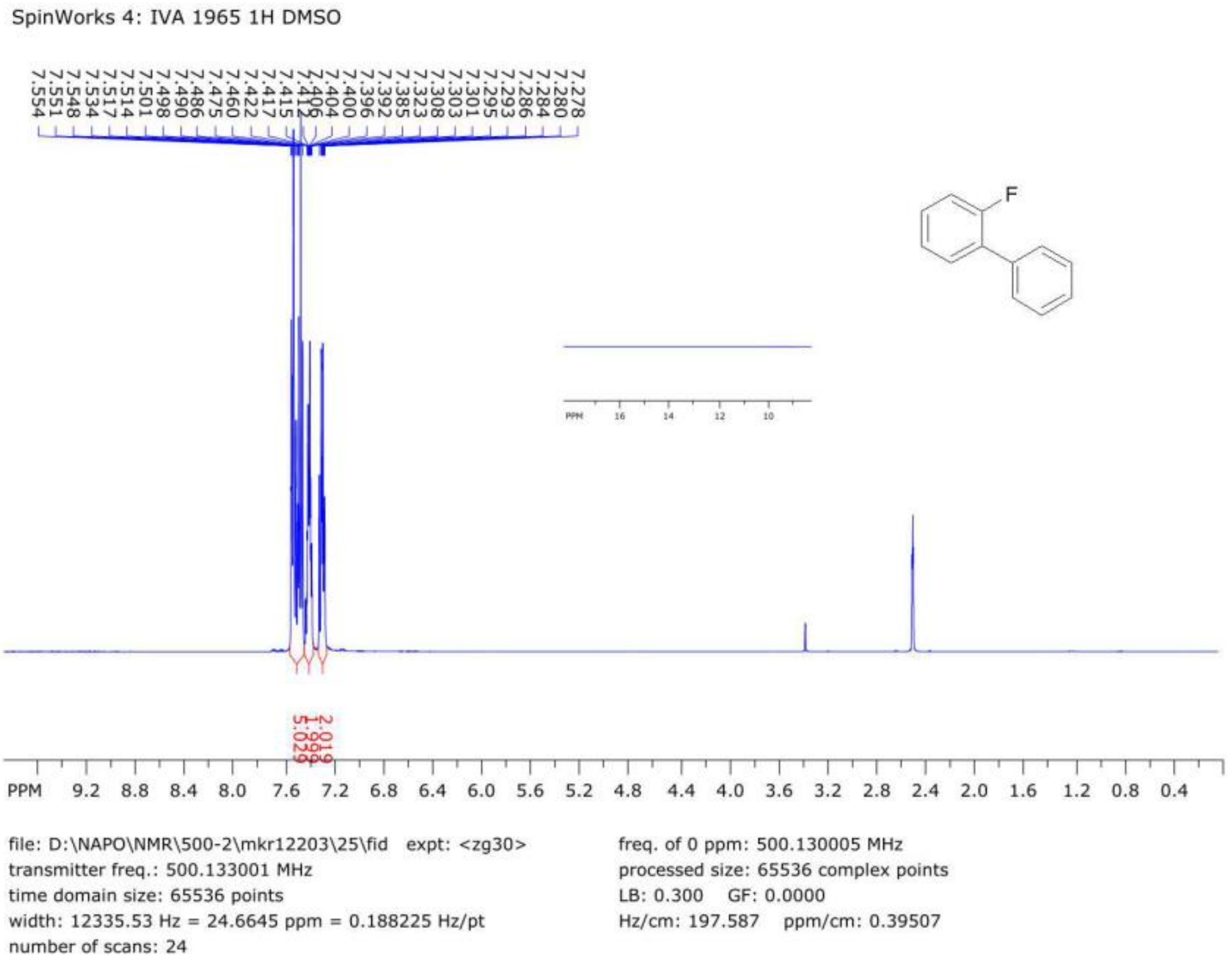
SpinWorks 4: IVA 1965 1H DMSO
7.554 7.551 7.548 7.534 7.517 7.514 7.501 7.498 7.490 7.486 7.475 7.460 7.422 7.417 7.415 7.412 7.406 7.404 7.400 7.396 7.392 7.385 7.323 7.308 7.303 7.301 7.295 7.293 7.286 7.284 7.280 7.278
F
PPM 16 14 12 10
PPM 9.2 8.8 8.4 8.0 7.6 7.2 6.8 6.4 6.0 5.6 5.2 4.8 4.4 4.0 3.6 3.2 2.8 2.4 2.0 1.6 1.2 0.8 0.4
file: D:\NAPO\NMR\500-2\mkr12203\25\fid expt: <zg30>
transmitter freq.: 500.133001 MHz
time domain size: 65536 points
width: 12335.53 Hz = 24.6645 ppm = 0.188225 Hz/pt
number of scans: 24
freq. of 0 ppm: 500.130005 MHz
processed size: 65536 complex points
LB: 0.300 GF: 0.0000
Hz/cm: 197.587 ppm/cm: 0.39507

Compound 2a

SpinWorks 4: IVA 1965 13C DSMO

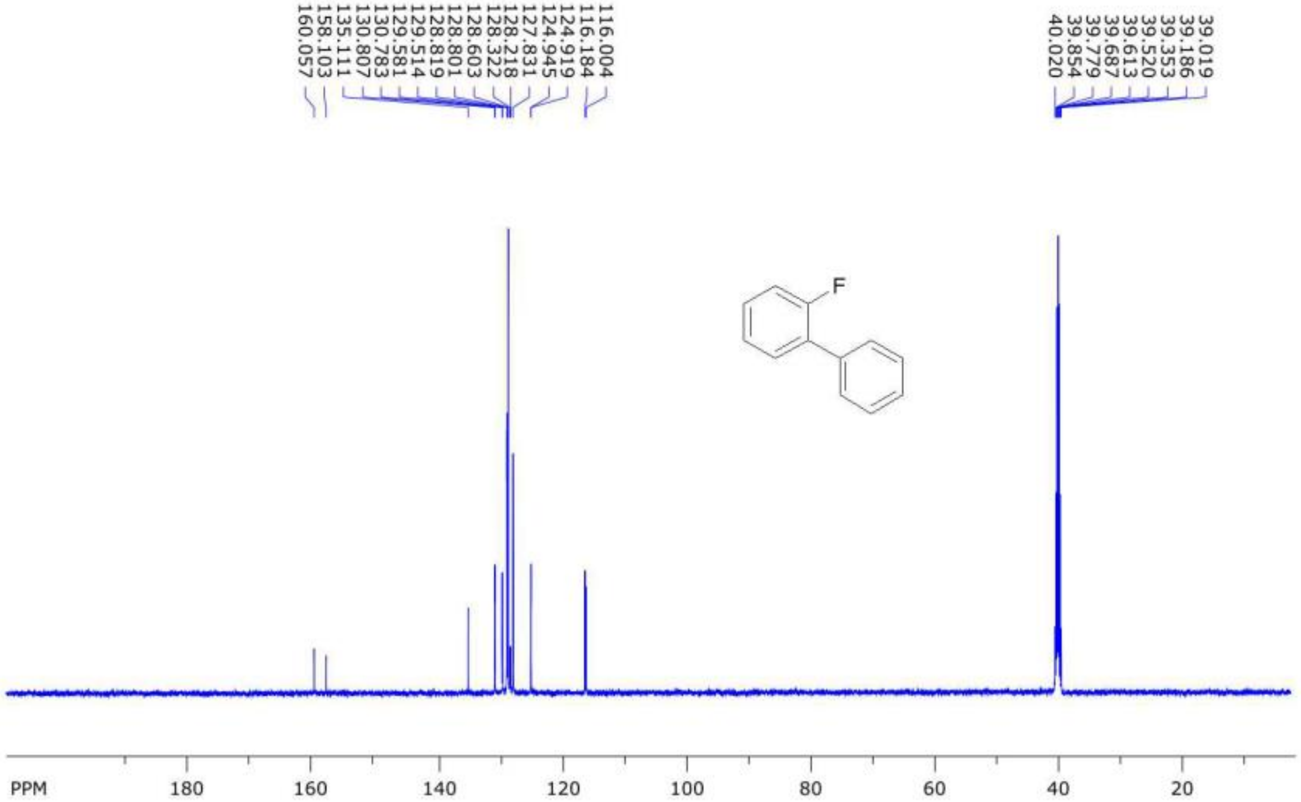

file: D:\NAPO\NMR\500-2\mkr12203\26\fid expt: <zgpg30>
transmitter freq.: 125.772879 MHz
time domain size: 65536 points
width: 36057.69 Hz = 286.6889 ppm = 0.550197 Hz/pt
number of scans: 512

freq. of 0 ppm: 125.757845 MHz
processed size: 32768 complex points
LB: 2.000 GF: 0.0000
Hz/cm: 1046.630 ppm/cm: 8.32159

Compound 2b

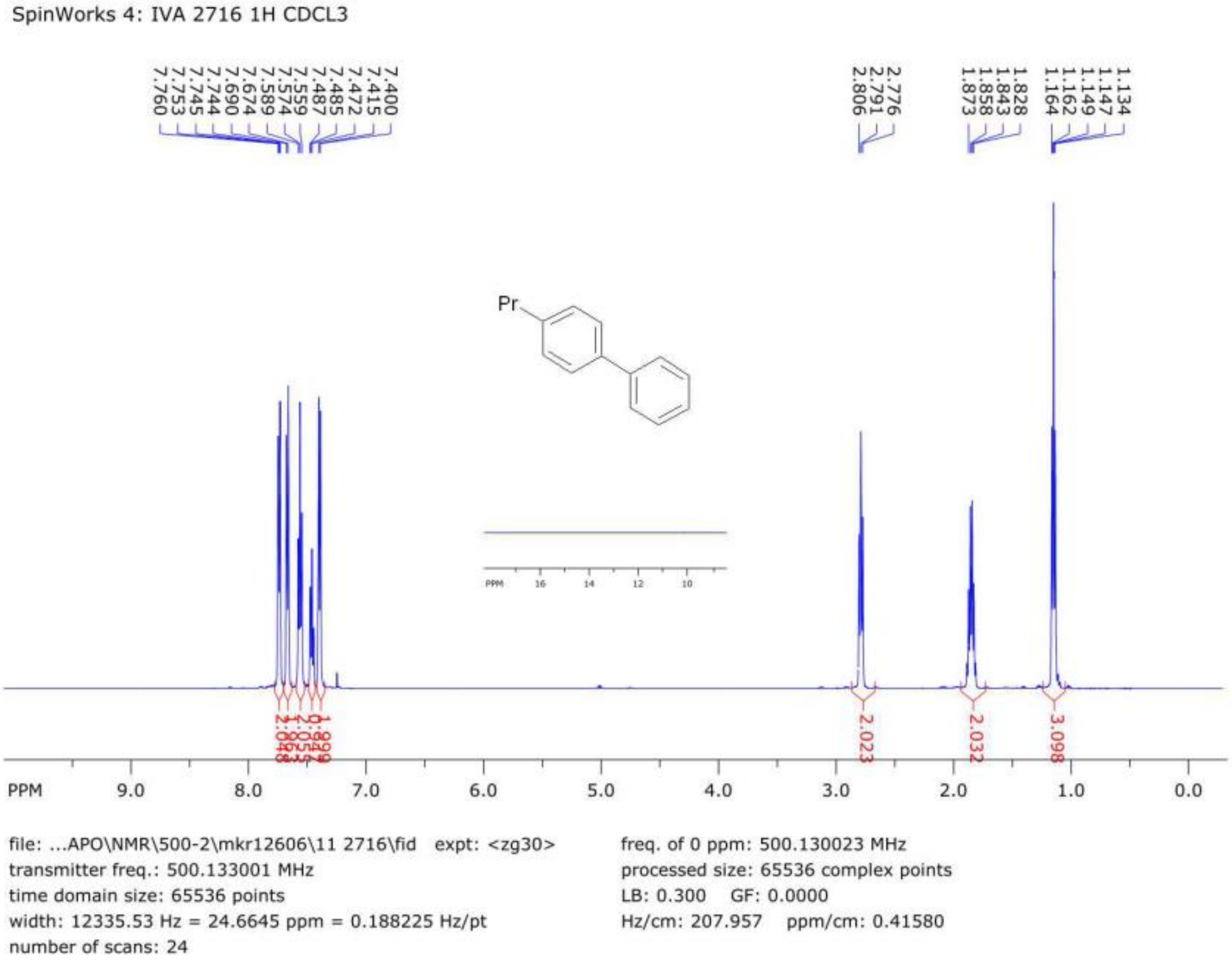
SpinWorks 4: IVA 2716 1H CDCL3
7.760
7.753
7.745
7.744
7.690
7.674
7.589
7.574
7.559
7.487
7.485
7.472
7.415
7.400
2.806
2.791
2.776
1.873
1.858
1.843
1.828
1.164
1.162
1.149
1.147
1.134
Pr
2.023
2.032
3.098
PPM
9.0
8.0
7.0
6.0
5.0
4.0
3.0
2.0
1.0
0.0
file: ...APO\NMR\500-2\mkr12606\11 2716\fid expt: <zg30>
transmitter freq.: 500.133001 MHz
time domain size: 65536 points
width: 12335.53 Hz = 24.6645 ppm = 0.188225 Hz/pt
number of scans: 24
freq. of 0 ppm: 500.130023 MHz
processed size: 65536 complex points
LB: 0.300 GF: 0.0000
Hz/cm: 207.957 ppm/cm: 0.41580

Compound 2b

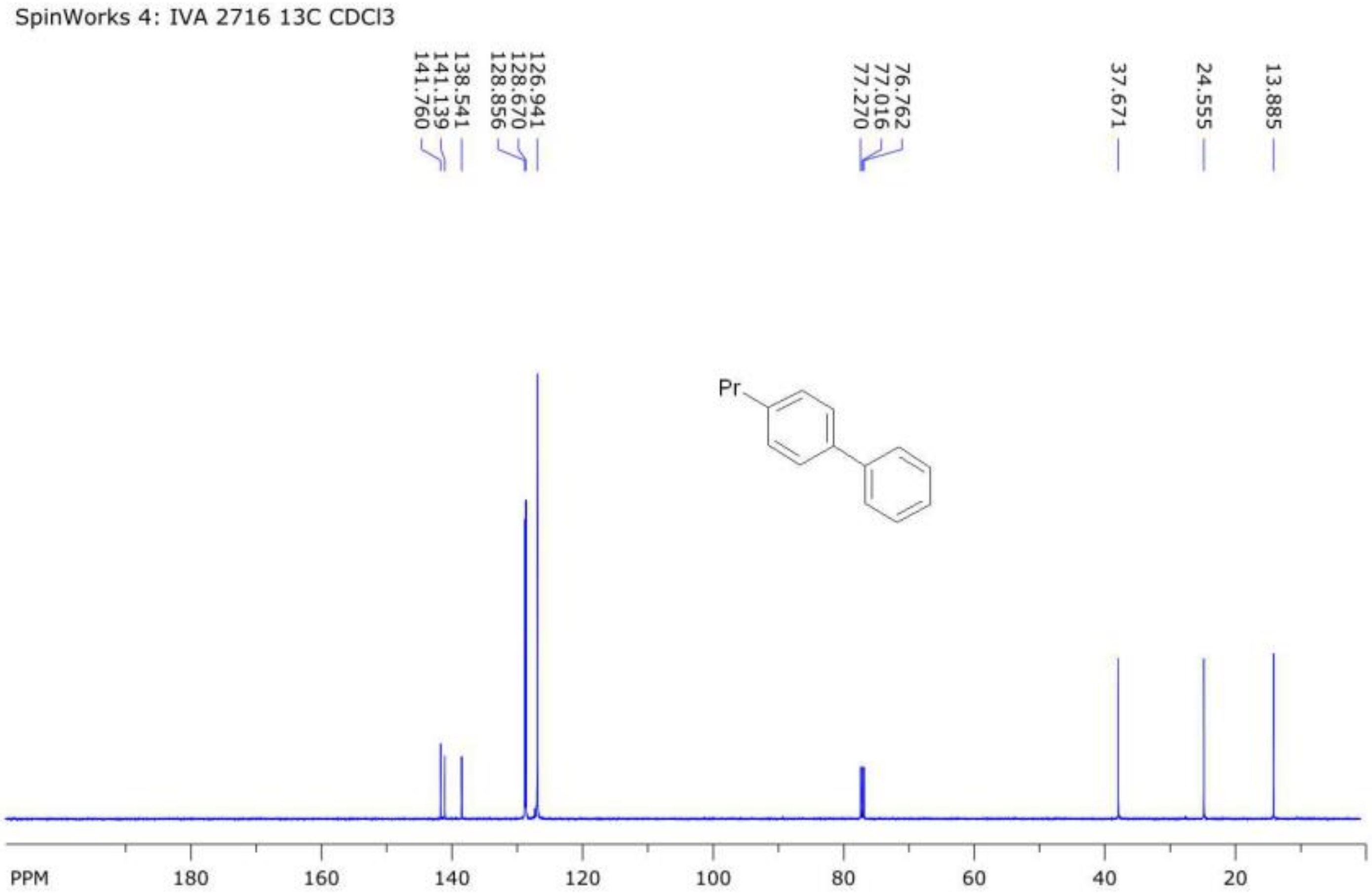

file: D:\NAPO\NMR\500-2\mkr12606\12\fid expt: <zgpg30>
transmitter freq.: 125.772879 MHz
time domain size: 65536 points
width: 36057.69 Hz = 286.6889 ppm = 0.550197 Hz/pt
number of scans: 512

freq. of 0 ppm: 125.757818 MHz
processed size: 32768 complex points
LB: 2.000 GF: 0.0000
Hz/cm: 1046.630 ppm/cm: 8.32159

Compound 2c

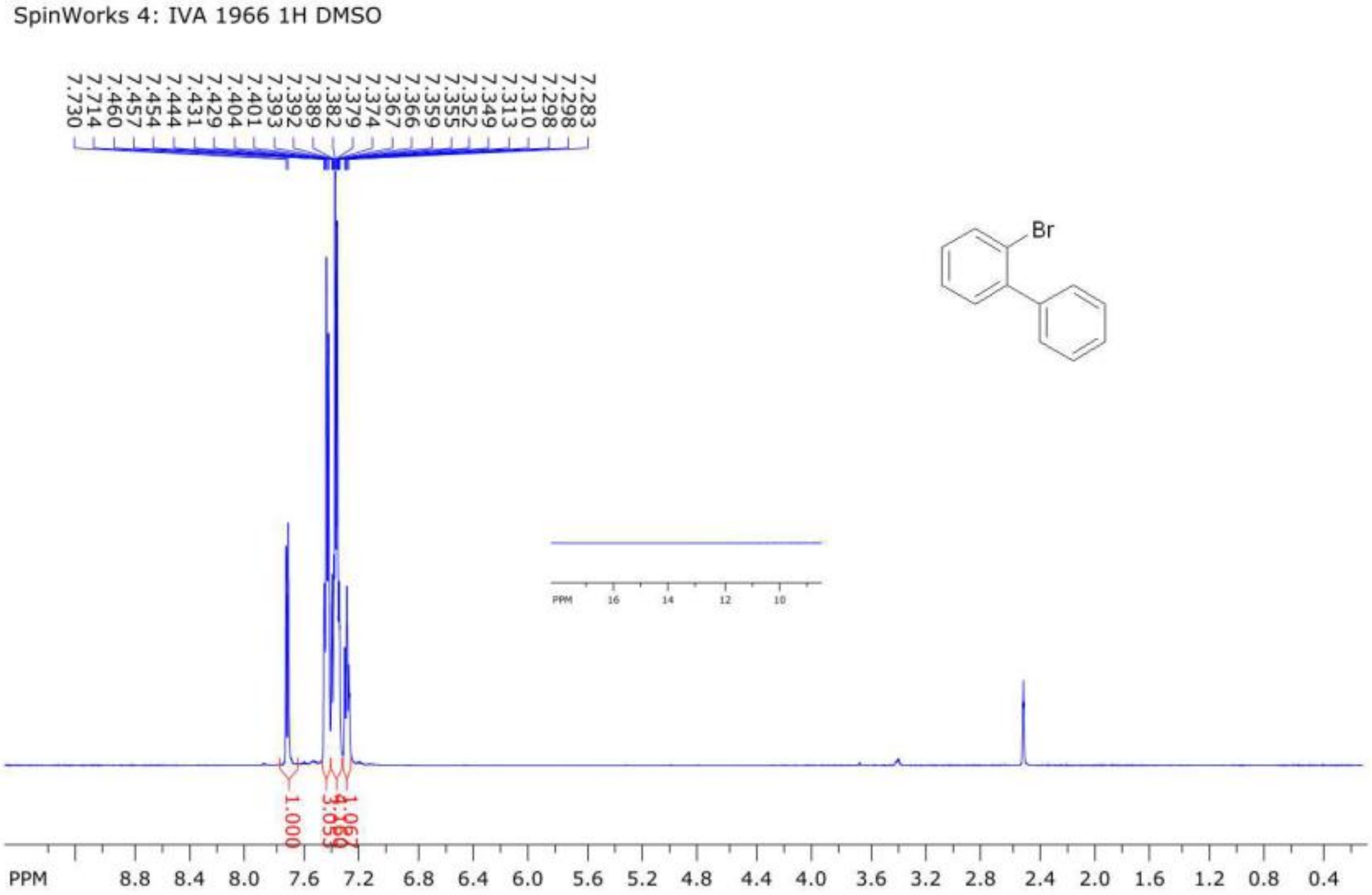

file: D:\NAPO\NMR\500-2\mkr11903\3\fid expt: <zg30>
transmitter freq.: 500.133001 MHz
time domain size: 65536 points
width: 12335.53 Hz = 24.6645 ppm = 0.188225 Hz/pt
number of scans: 24

freq. of 0 ppm: 500.130005 MHz
processed size: 65536 complex points
LB: 0.300 GF: 0.0000
Hz/cm: 192.674 ppm/cm: 0.38525

Compound 2c

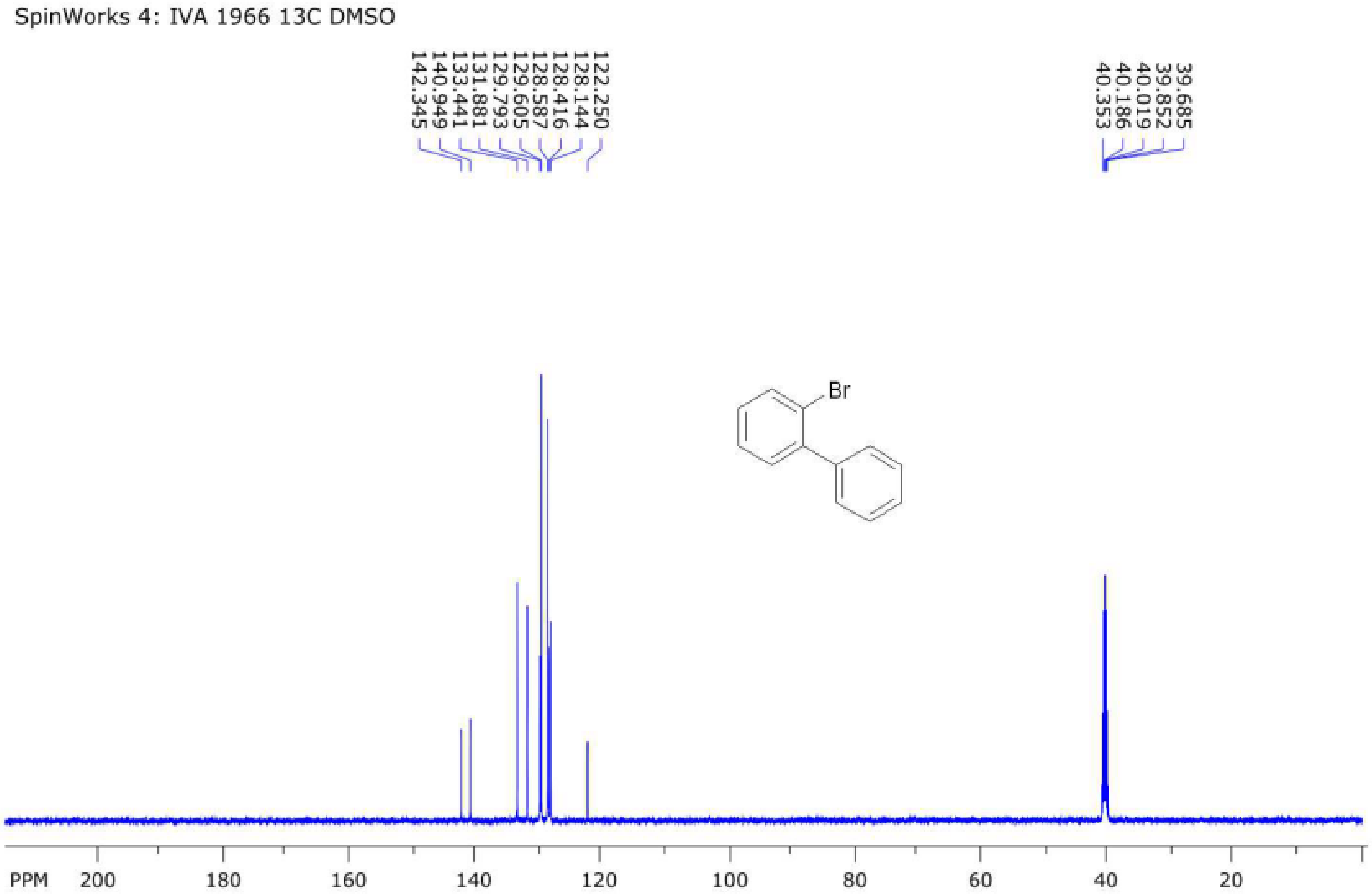

file: D:\NAPO\NMR\500-2\mkr11903\4\fid expt: <zgpg30>
transmitter freq.: 125.772879 MHz
time domain size: 65536 points
width: 36057.69 Hz = 286.6889 ppm = 0.550197 Hz/pt
number of scans: 128

freq. of 0 ppm: 125.757789 MHz
processed size: 32768 complex points
LB: 2.000 GF: 0.0000
Hz/cm: 1084.922 ppm/cm: 8.62604

Compound 2d

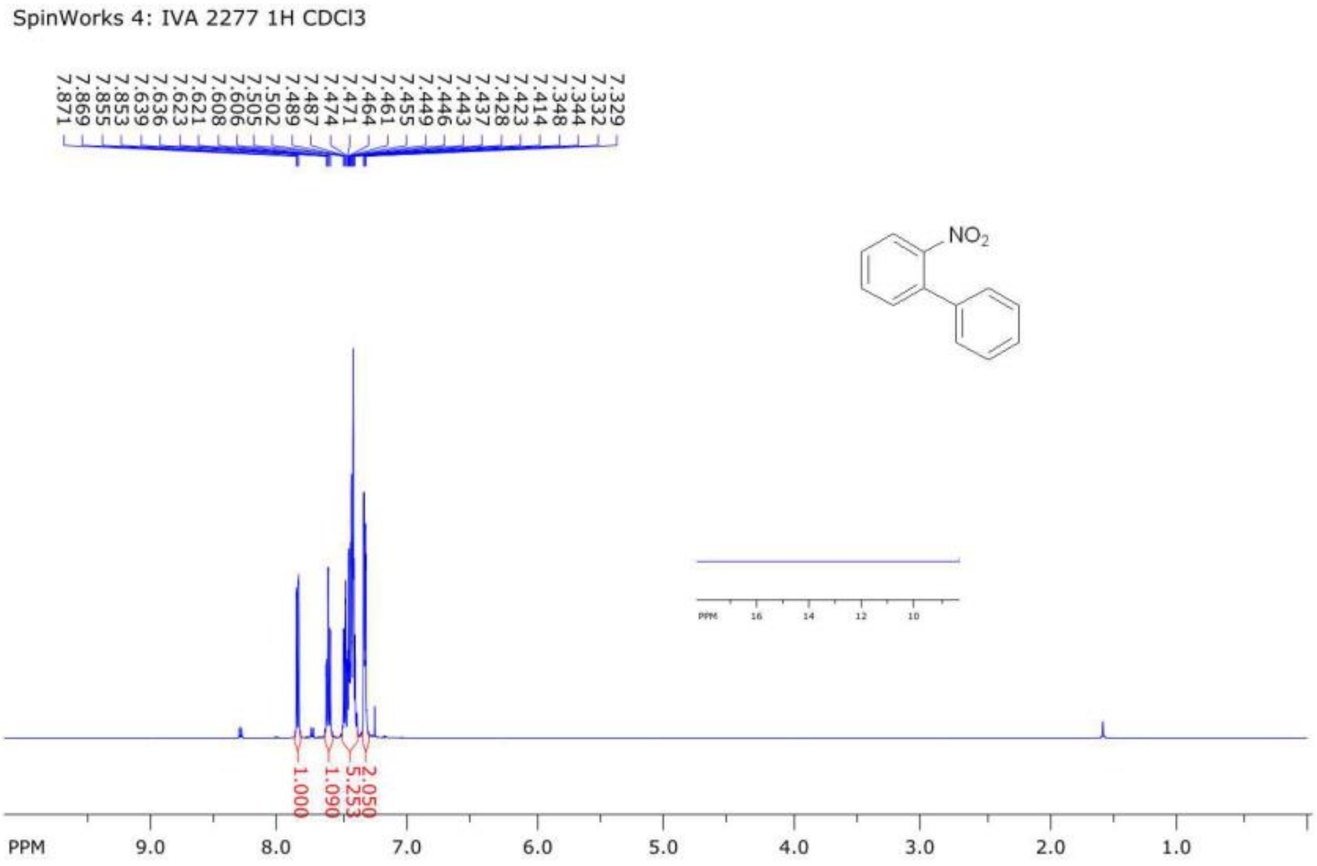

file: D:\NAPO\NMR\500-2\mkr12404\15\fid expt: <zg30>
transmitter freq.: 500.133001 MHz
time domain size: 65536 points
width: 12335.53 Hz = 24.6645 ppm = 0.188225 Hz/pt
number of scans: 24

freq. of 0 ppm: 500.130023 MHz
processed size: 65536 complex points
LB: 0.300 GF: 0.0000
Hz/cm: 203.591 ppm/cm: 0.40707

Compound 2d

SpinWorks 4: IVA 2277 13C CDCl3

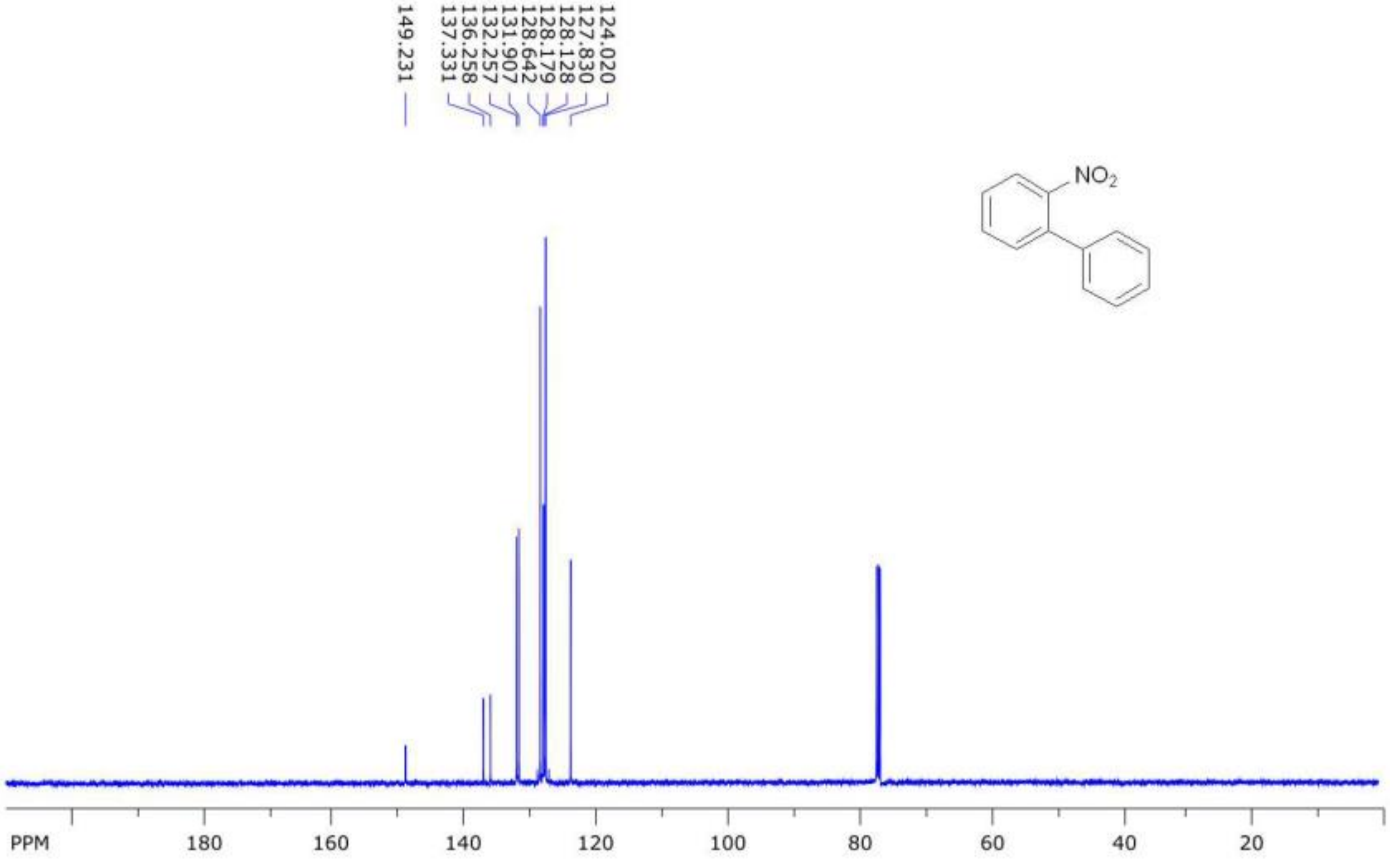

file: D:\NAPO\NMR\500-2\mkr12404\16\fid expt: <zgpg30>
transmitter freq.: 125.772879 MHz
time domain size: 65536 points
width: 36057.69 Hz = 286.6889 ppm = 0.550197 Hz/pt
number of scans: 512

freq. of 0 ppm: 125.757801 MHz
processed size: 32768 complex points
LB: 2.000 GF: 0.0000
Hz/cm: 1054.608 ppm/cm: 8.38502

Compound 2e

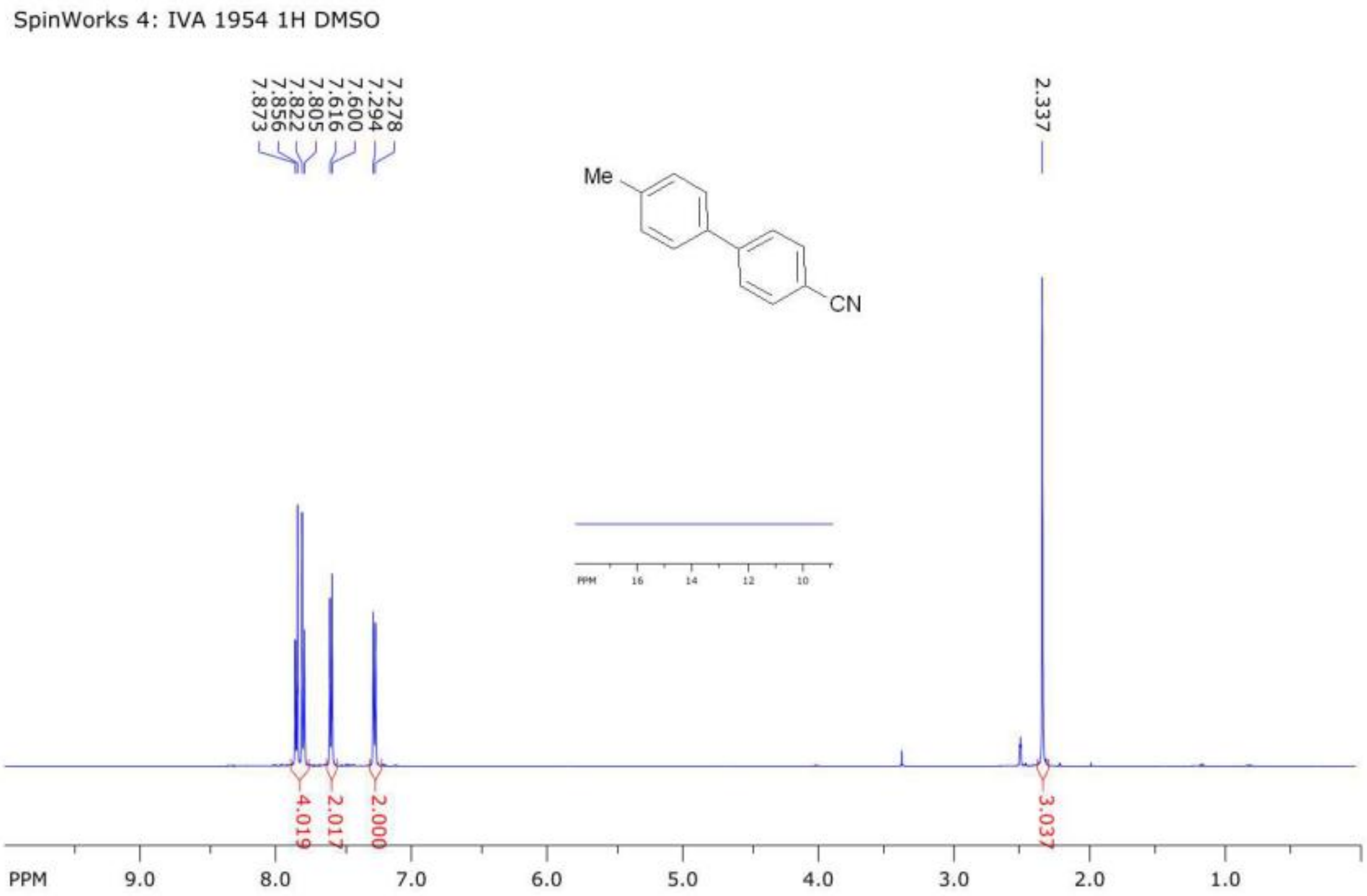

file: D:\NAPO\NMR\500-2\mkr10804\1\fid expt: <zg30>
transmitter freq.: 500.133001 MHz
time domain size: 65536 points
width: 12335.53 Hz = 24.6645 ppm = 0.188225 Hz/pt
number of scans: 24

freq. of 0 ppm: 500.130005 MHz
processed size: 65536 complex points
LB: 0.300 GF: 0.0000
Hz/cm: 200.316 ppm/cm: 0.40053

Compound 2e

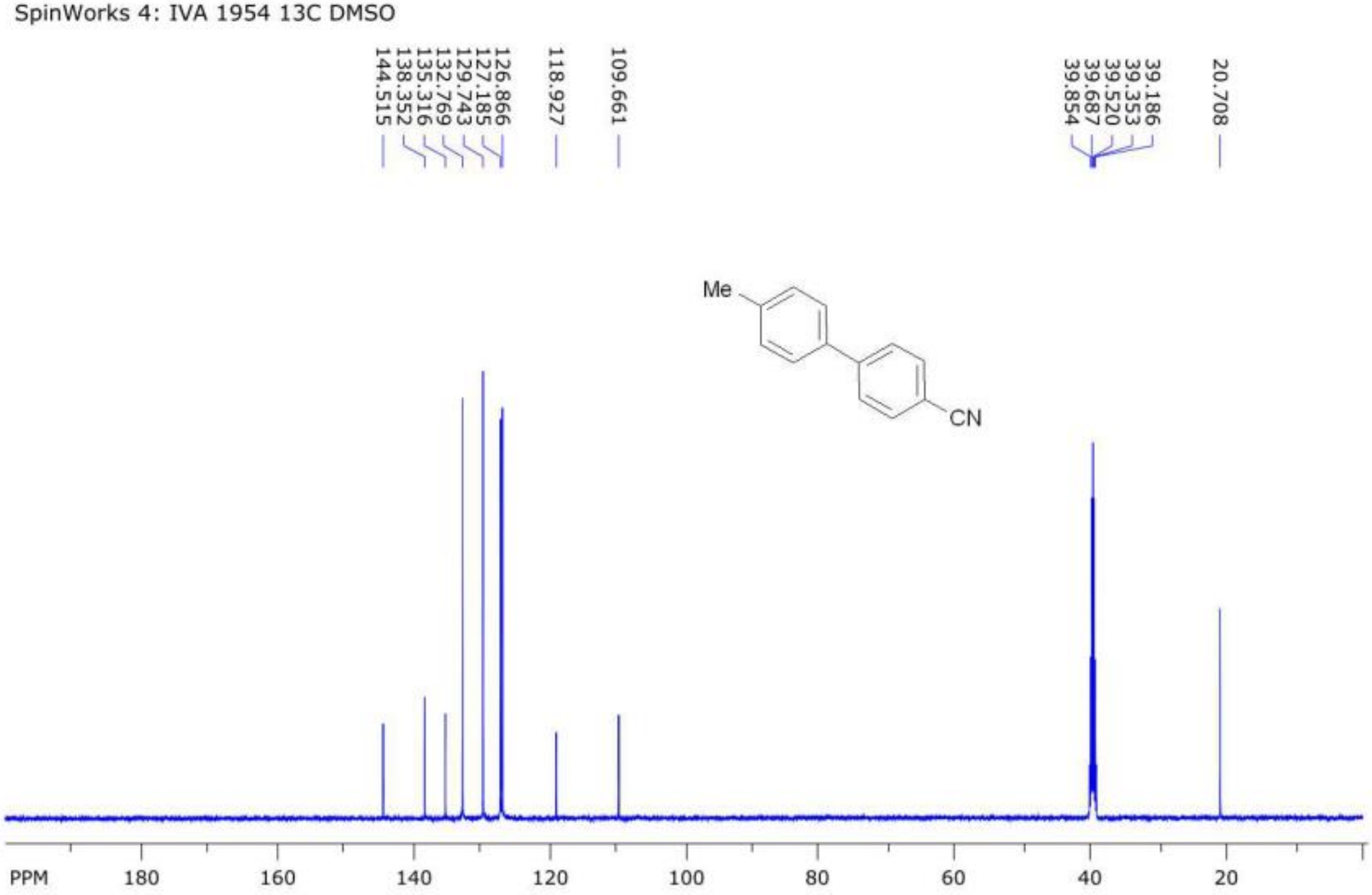

file: D:\NAPO\NMR\500-2\mkr10804\2\fid expt: <zgpg30>
transmitter freq.: 125.772879 MHz
time domain size: 65536 points
width: 36057.69 Hz = 286.6889 ppm = 0.550197 Hz/pt
number of scans: 512

freq. of 0 ppm: 125.757846 MHz
processed size: 32768 complex points
LB: 2.000 GF: 0.0000
Hz/cm: 1009.934 ppm/cm: 8.02983

Compound 2f

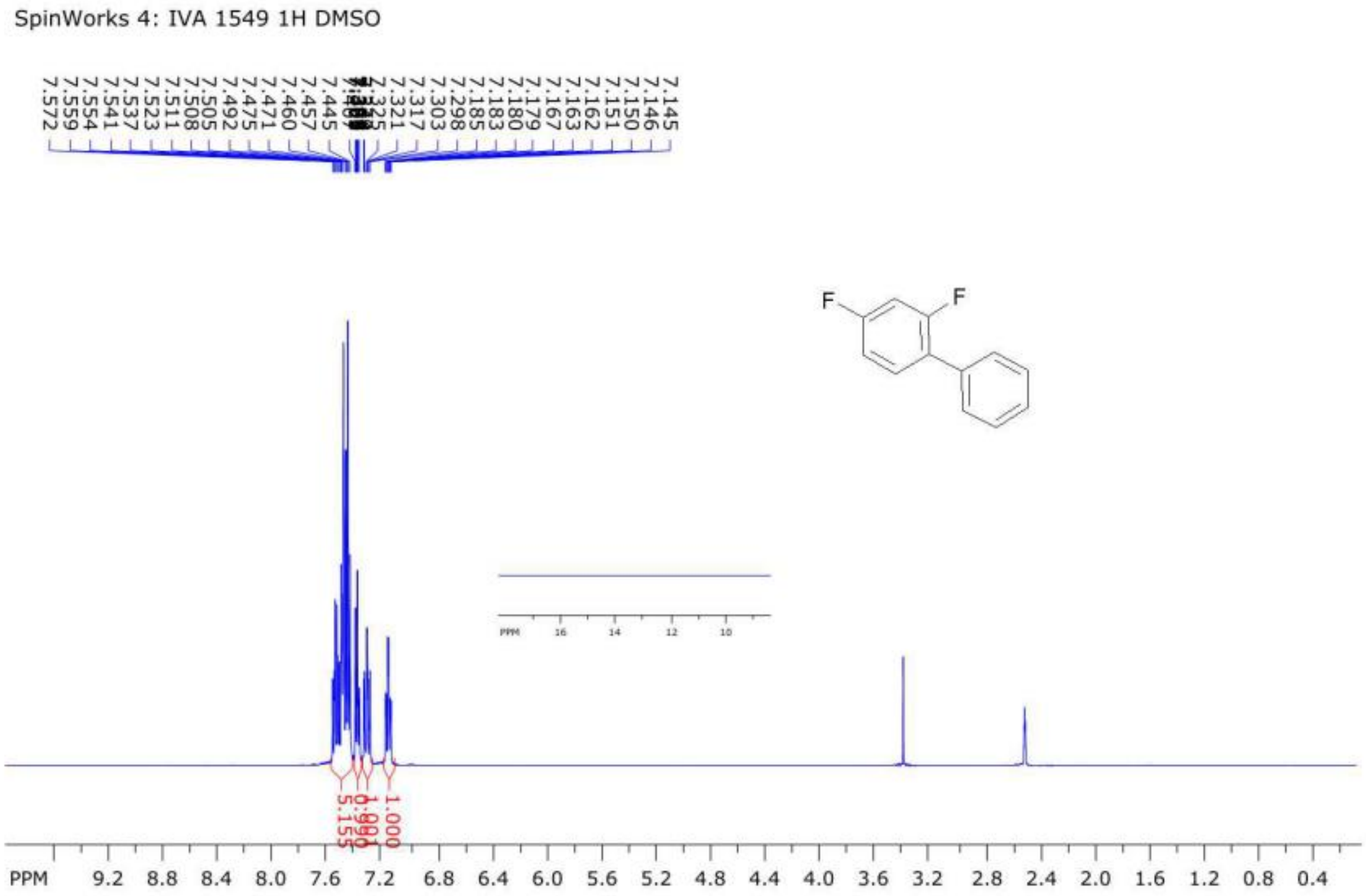

file: D:\NAPO\NMR\500-2\mkr11705\5\fid expt: <zg30>
transmitter freq.: 500.133001 MHz
time domain size: 65536 points
width: 12335.53 Hz = 24.6645 ppm = 0.188225 Hz/pt
number of scans: 24

freq. of 0 ppm: 500.130005 MHz
processed size: 65536 complex points
LB: 0.300 GF: 0.0000
Hz/cm: 198.133 ppm/cm: 0.39616

Compound 2f

SpinWorks 4: IVA 1549 13C DMSO

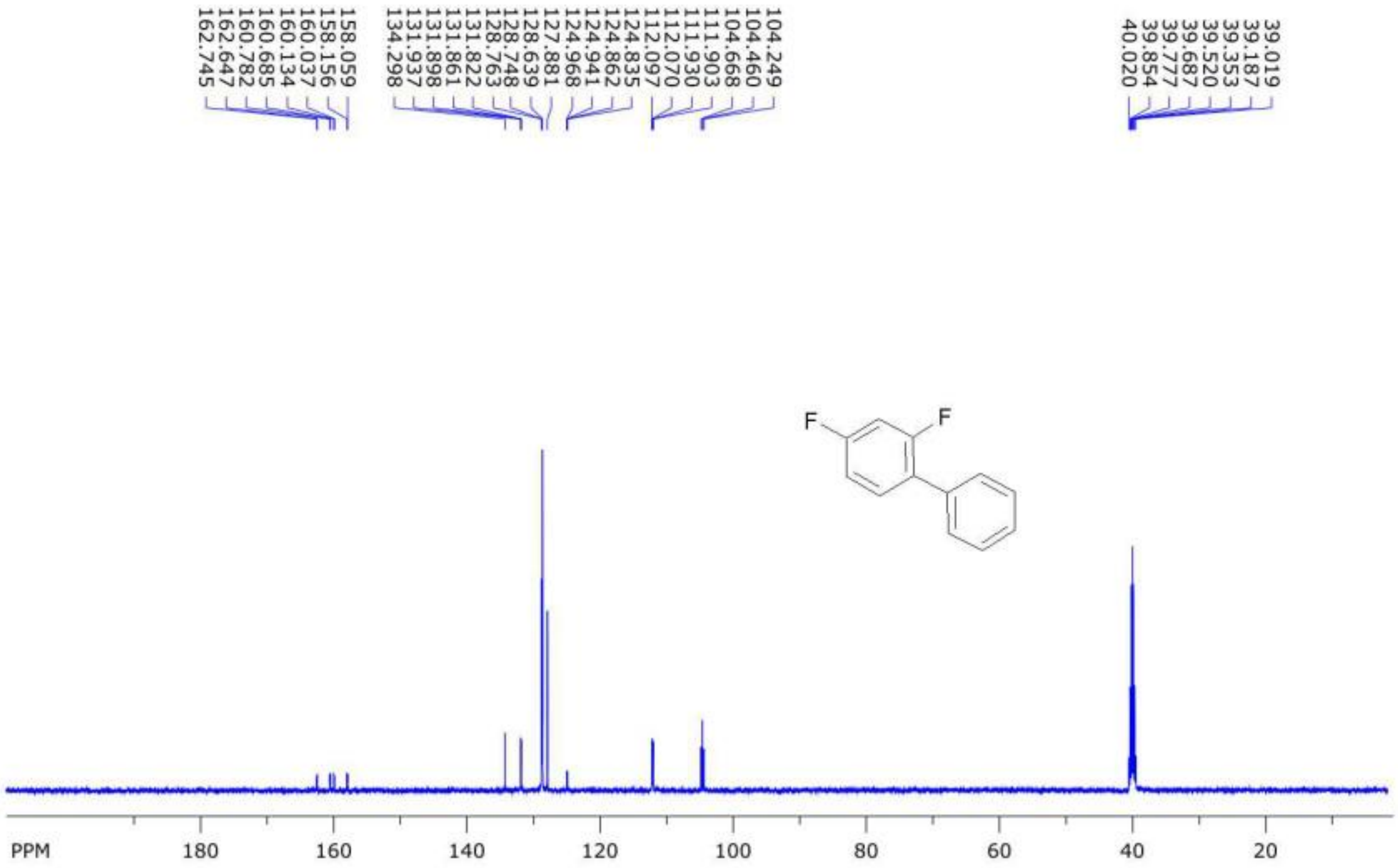

file: D:\NAPO\NMR\500-2\mkr11705\6\fid expt: <zgpg30>
transmitter freq.: 125.772879 MHz
time domain size: 65536 points
width: 36057.69 Hz = 286.6889 ppm = 0.550197 Hz/pt
number of scans: 128

freq. of 0 ppm: 125.757843 MHz
processed size: 32768 complex points
LB: 2.000 GF: 0.0000
Hz/cm: 1049.821 ppm/cm: 8.34696

Compound 2g

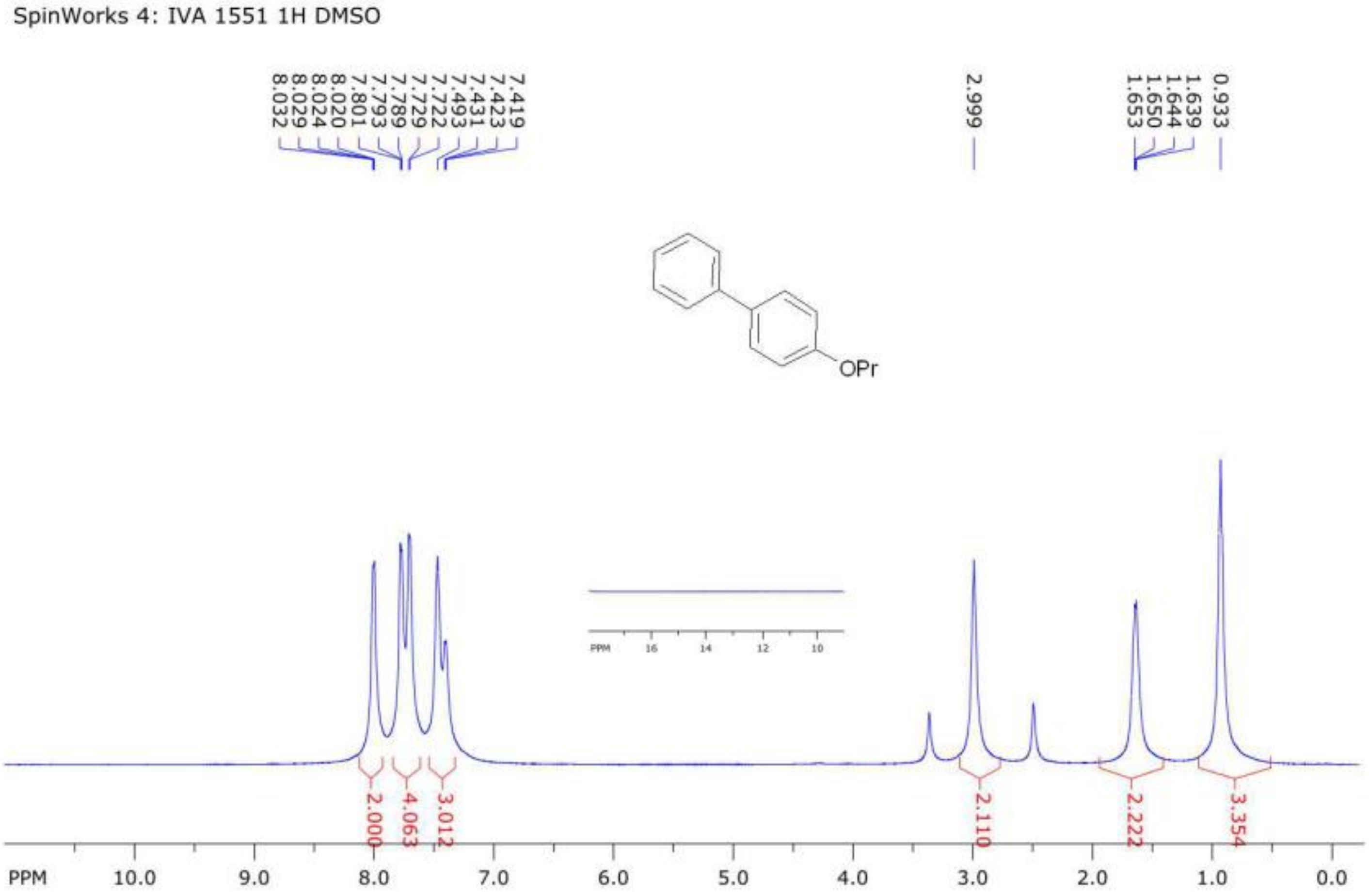

file: ...APO\NMR\500-2\mkr11507\19 1551\fid expt: <zg30>
transmitter freq.: 500.133001 MHz
time domain size: 65536 points
width: 12335.53 Hz = 24.6645 ppm = 0.188225 Hz/pt
number of scans: 24

freq. of 0 ppm: 500.130003 MHz
processed size: 65536 complex points
LB: 0.300 GF: 0.0000
Hz/cm: 227.061 ppm/cm: 0.45400

Compound 2g

SpinWorks 4: IVA 1551 13C DMSO

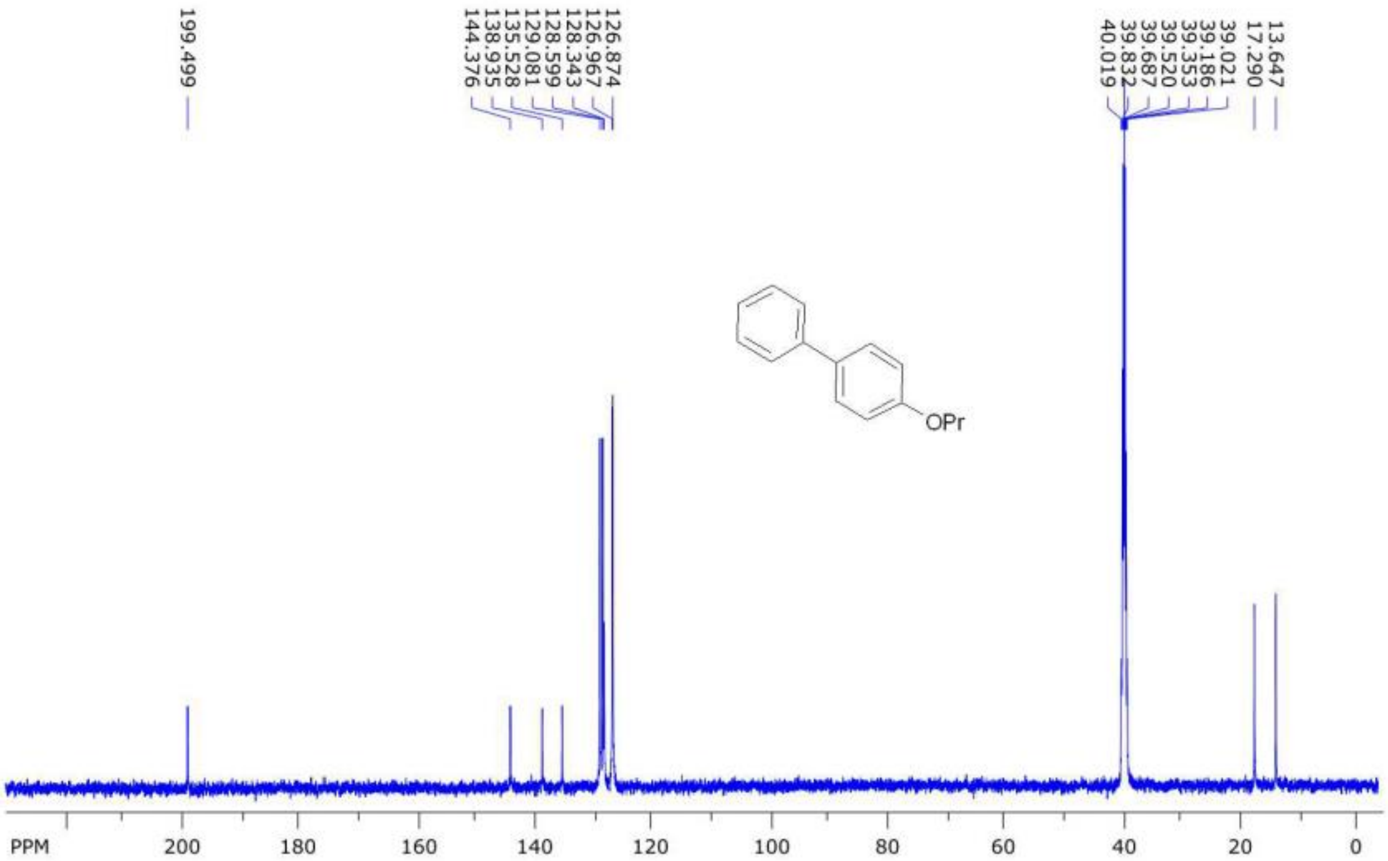

file: D:\NAPO\NMR\500-2\mkr11507\20\fid expt: <zgpg30>
transmitter freq.: 125.772879 MHz
time domain size: 65536 points
width: 36057.69 Hz = 286.6889 ppm = 0.550197 Hz/pt
number of scans: 512

freq. of 0 ppm: 125.757844 MHz
processed size: 32768 complex points
LB: 2.000 GF: 0.0000
Hz/cm: 1179.055 ppm/cm: 9.37447

Compound 2h

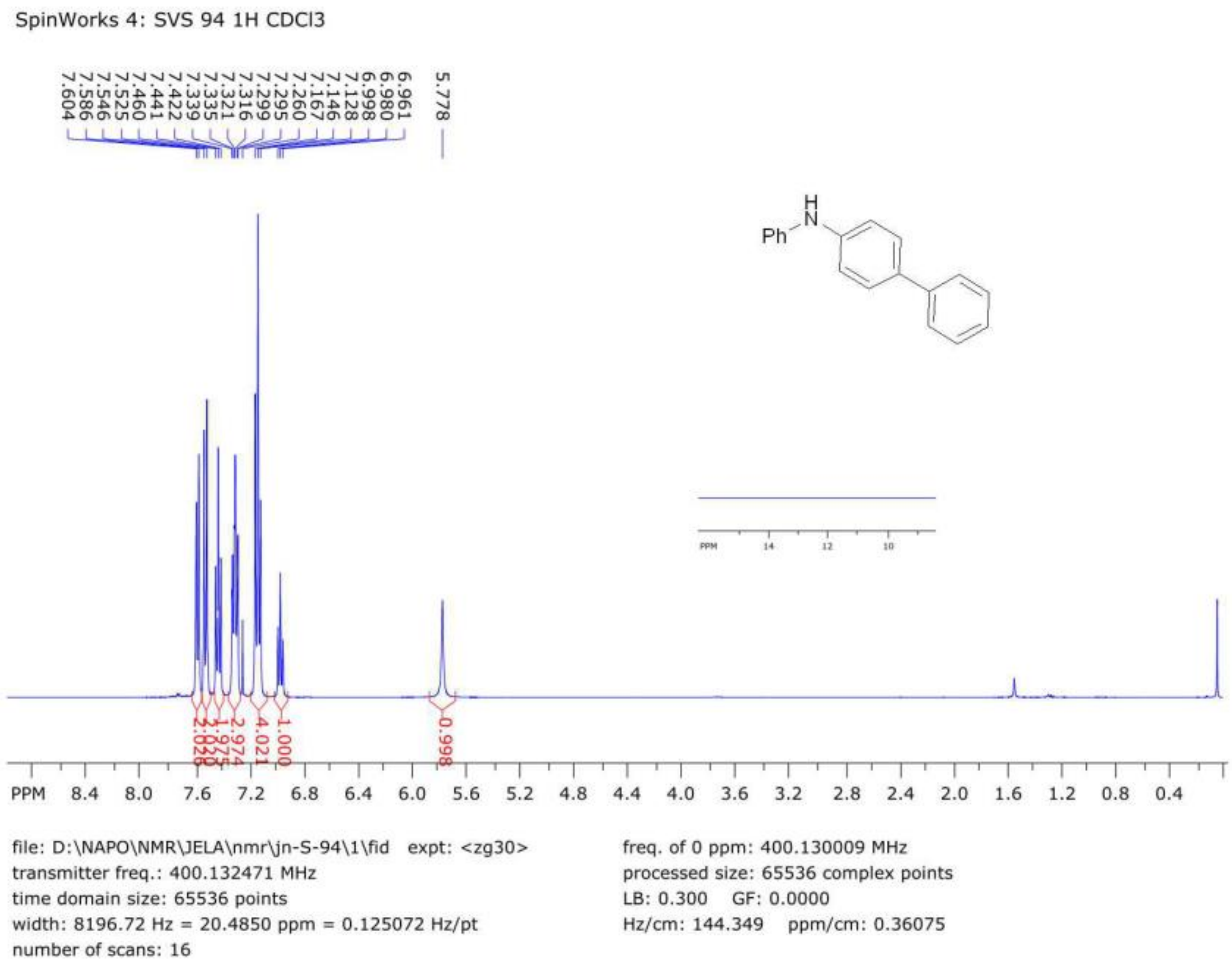
SpinWorks 4: SVS 94 1H CDCl3
7.604
7.586
7.546
7.525
7.460
7.441
7.422
7.339
7.335
7.321
7.316
7.299
7.295
7.260
7.167
7.146
7.128
6.998
6.980
6.961
5.778
Ph
N
H
PPM 14 12 10
2.026
2.020
1.975
2.974
4.021
1.000
0.998
PPM 8.4 8.0 7.6 7.2 6.8 6.4 6.0 5.6 5.2 4.8 4.4 4.0 3.6 3.2 2.8 2.4 2.0 1.6 1.2 0.8 0.4
file: D:\NAPO\NMR\JELA\nmr\jn-S-94\1\fid expt: <zg30>
transmitter freq.: 400.132471 MHz
time domain size: 65536 points
width: 8196.72 Hz = 20.4850 ppm = 0.125072 Hz/pt
number of scans: 16
freq. of 0 ppm: 400.130009 MHz
processed size: 65536 complex points
LB: 0.300 GF: 0.0000
Hz/cm: 144.349 ppm/cm: 0.36075

Compound 2h

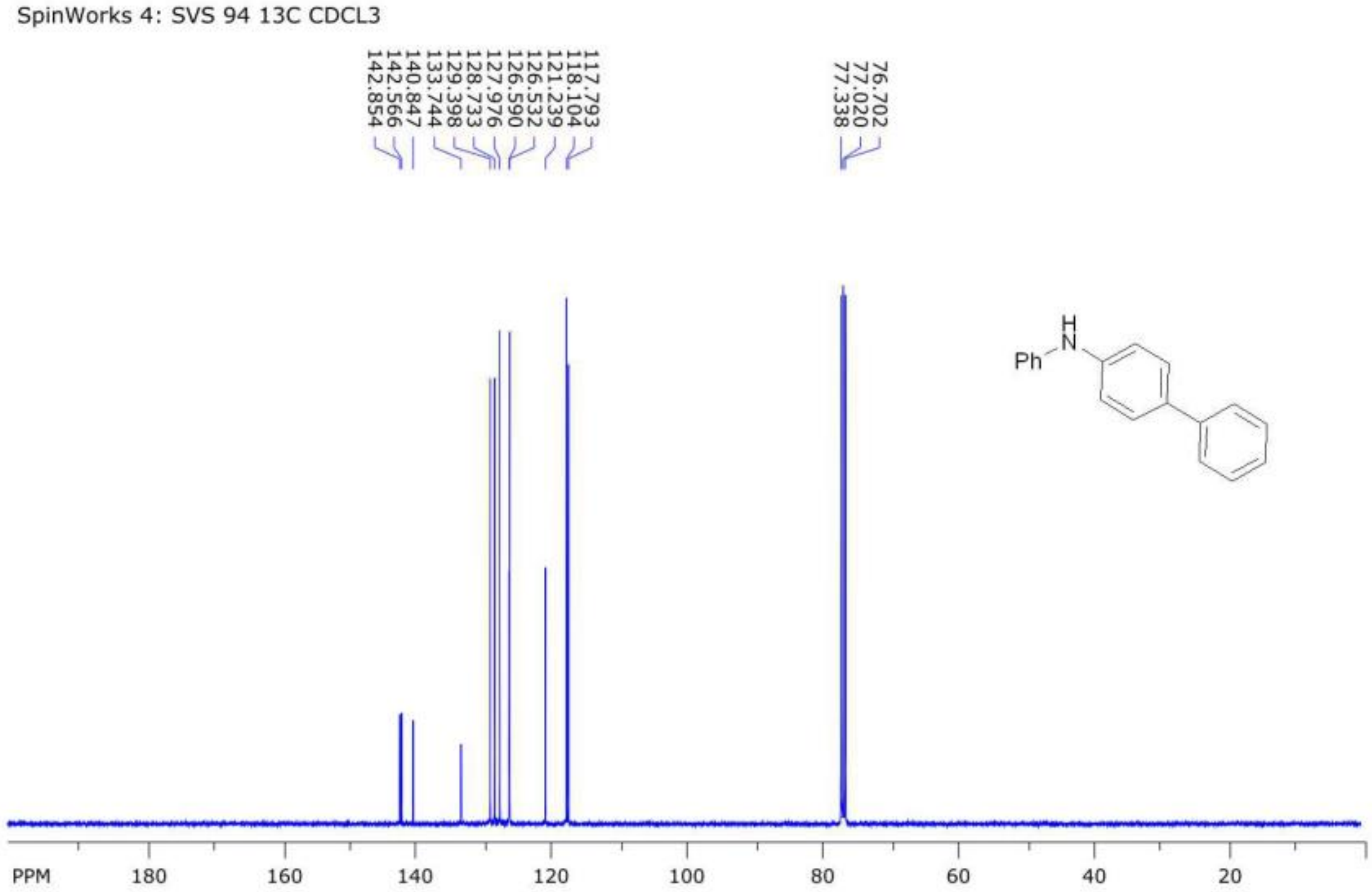

file: D:\NAPO\NMR\JELA\nmr\jn-S-94\2\fid expt: <zgpg30>
transmitter freq.: 100.622830 MHz
time domain size: 65536 points
width: 23809.52 Hz = 236.6215 ppm = 0.363305 Hz/pt
number of scans: 3500

freq. of 0 ppm: 100.612771 MHz
processed size: 32768 complex points
LB: 1.000 GF: 0.0000
Hz/cm: 808.049 ppm/cm: 8.03047

Compound 2i

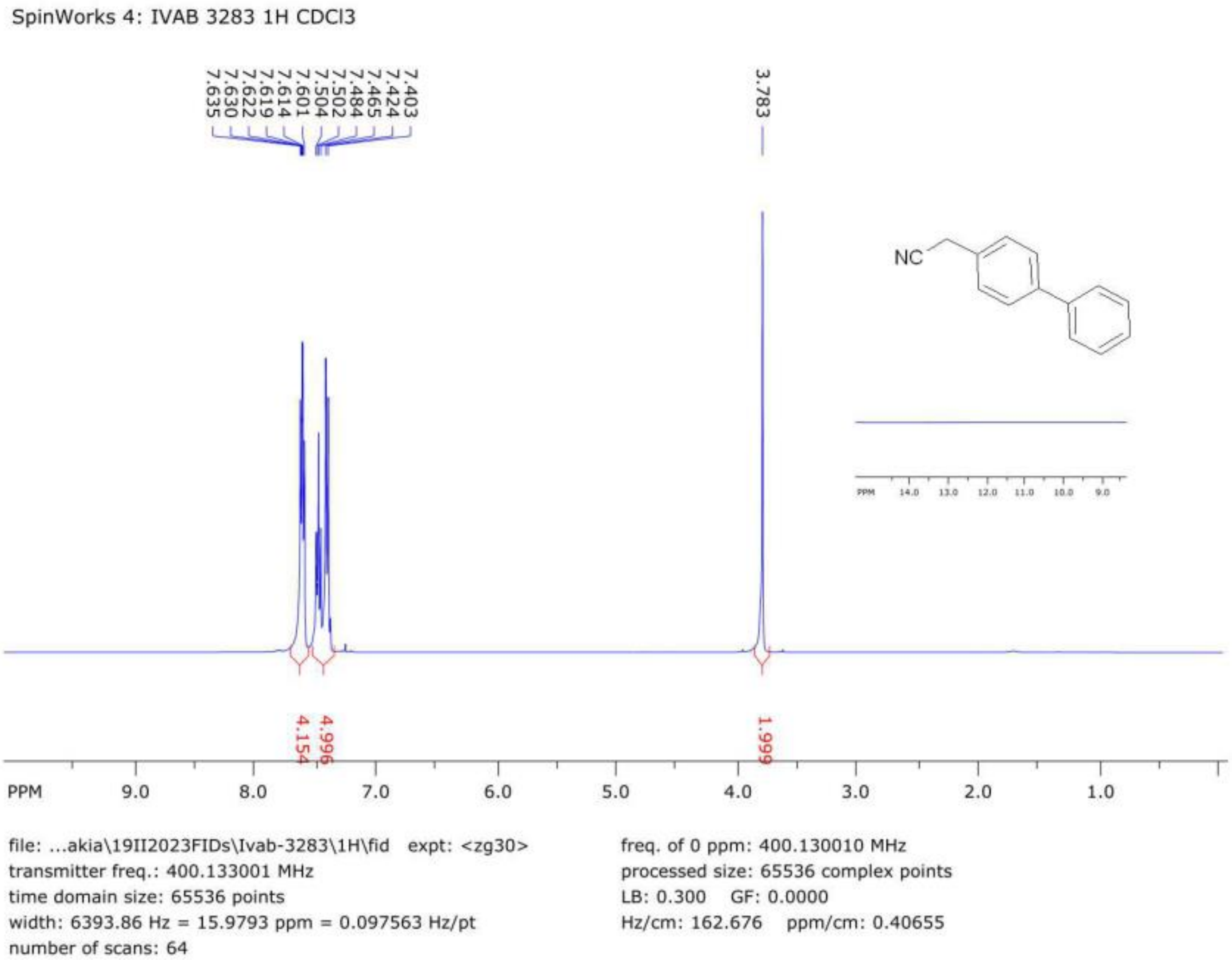

SpinWorks 4: IVAB 3283 1H CDCl3
7.635
7.630
7.622
7.619
7.614
7.601
7.504
7.502
7.484
7.465
7.424
7.403
3.783
NC
4.154
4.996
1.999
PPM
9.0
8.0
7.0
6.0
5.0
4.0
3.0
2.0
1.0
file: ...akia\19II2023FIDs\Ivab-3283\1H\fid expt: <zg30>
transmitter freq.: 400.133001 MHz
time domain size: 65536 points
width: 6393.86 Hz = 15.9793 ppm = 0.097563 Hz/pt
number of scans: 64
freq. of 0 ppm: 400.130010 MHz
processed size: 65536 complex points
LB: 0.300 GF: 0.0000
Hz/cm: 162.676 ppm/cm: 0.40655

Compound 2i

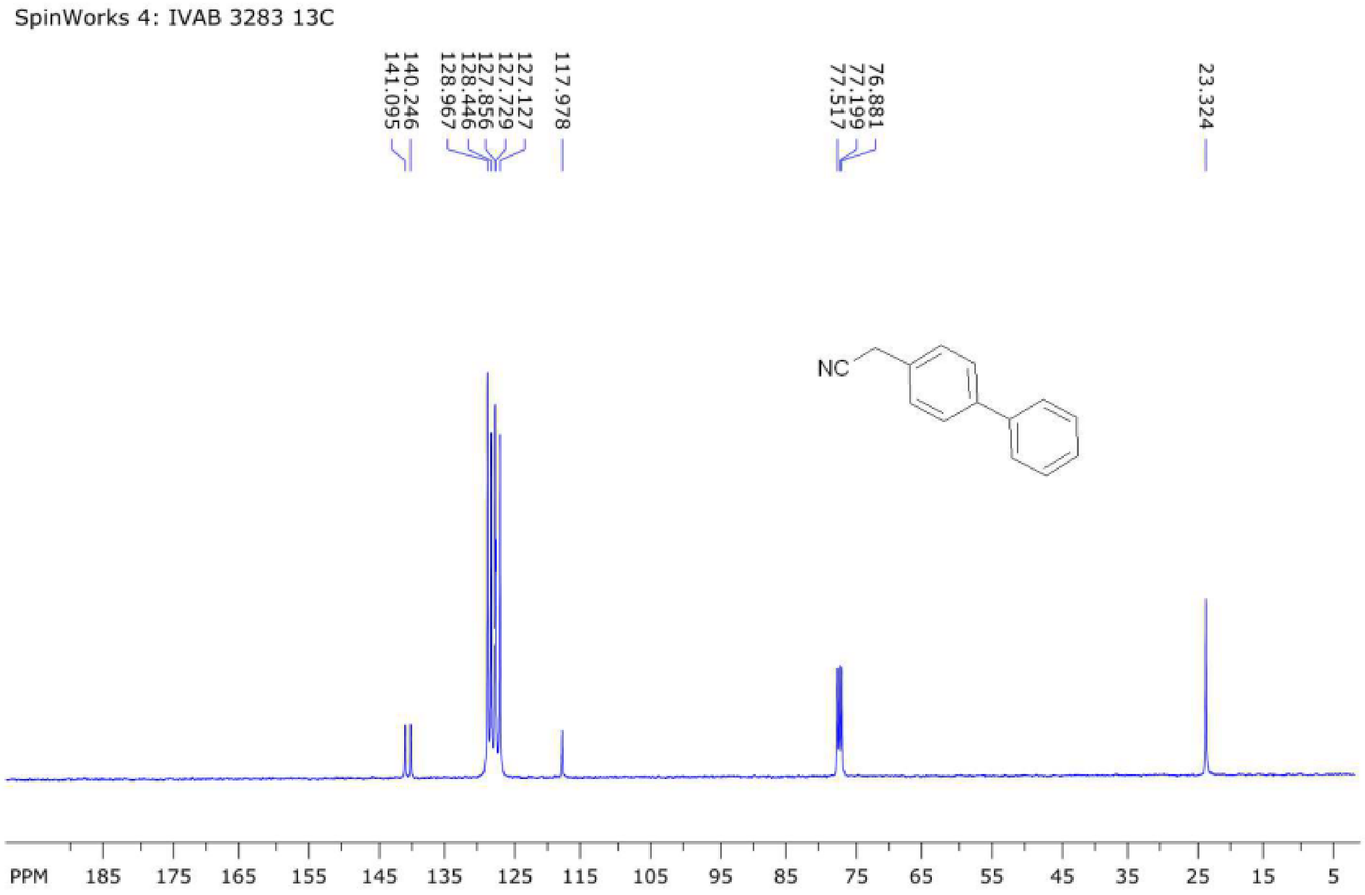

file: ...kia\19II2023FIDs\Ivab-3283\13C\fid expt: <zgpg30>
transmitter freq.: 100.623836 MHz
time domain size: 65536 points
width: 24038.46 Hz = 238.8943 ppm = 0.366798 Hz/pt
number of scans: 751

freq. of 0 ppm: 100.612769 MHz
processed size: 32768 complex points
LB: 10.000 GF: 0.0000
Hz/cm: 798.800 ppm/cm: 7.93848

Compound 2j

SpinWorks 4: SVS 134 1H DMSO

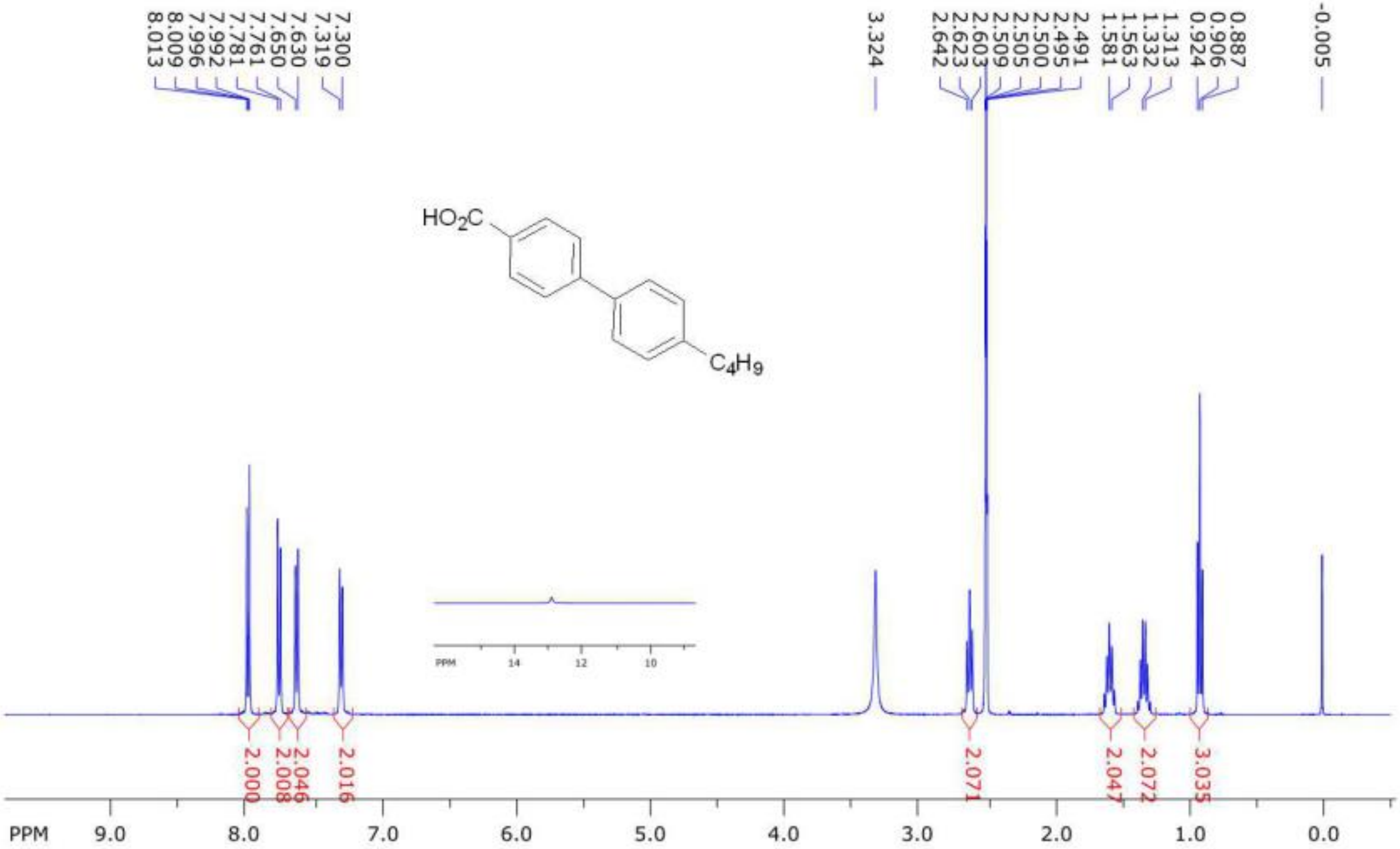

file: D:\NAPO\NMR\JELA\nmr\jn-134\1\fid expt: <zg30>
transmitter freq.: 400.132471 MHz
time domain size: 65536 points
width: 8196.72 Hz = 20.4850 ppm = 0.125072 Hz/pt
number of scans: 16

freq. of 0 ppm: 400.130002 MHz
processed size: 65536 complex points
LB: 0.300 GF: 0.0000
Hz/cm: 165.385 ppm/cm: 0.41333

Compound 2j

SpinWorks 4: SVS 134 13C DMSO

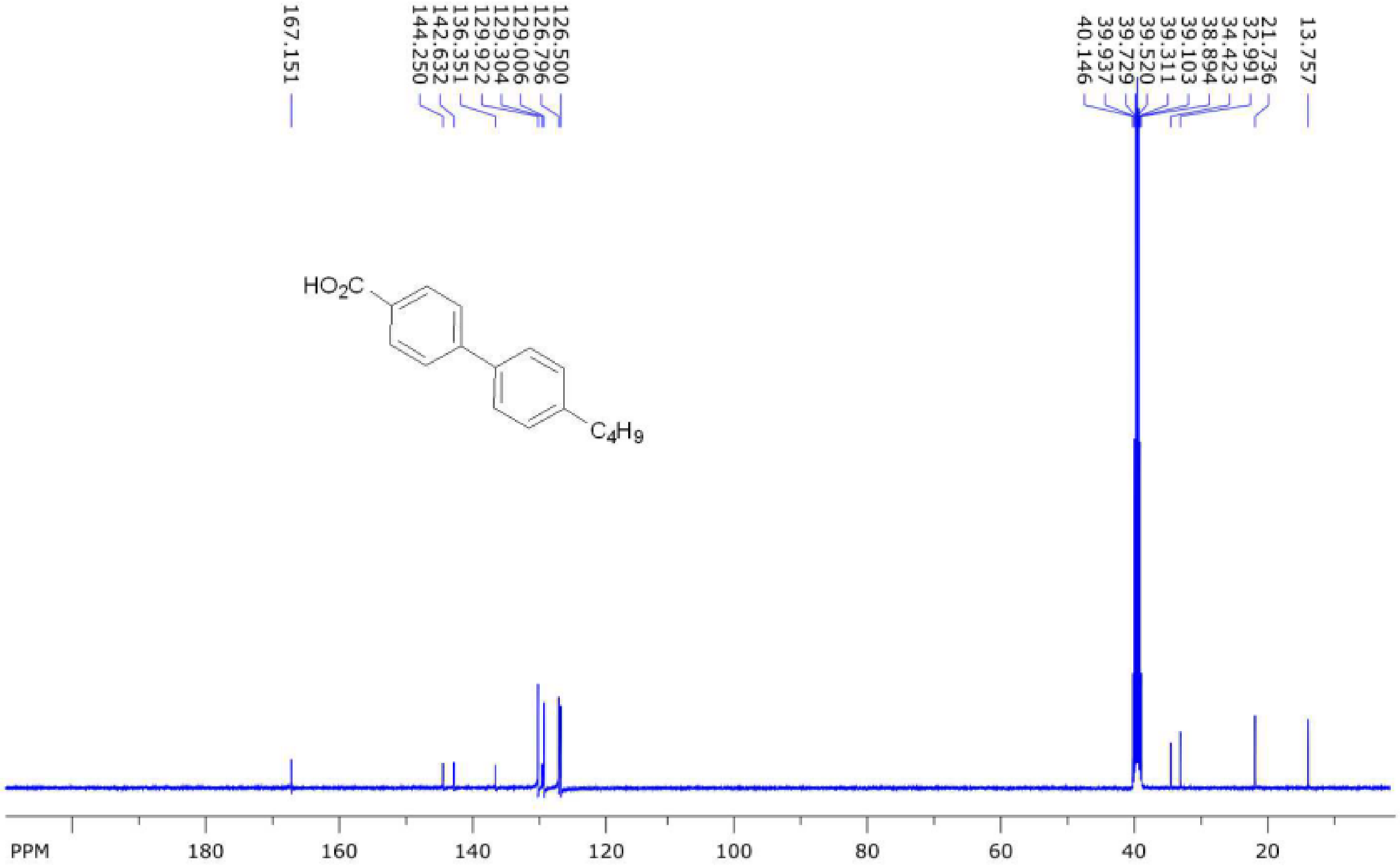

file: D:\NAPO\NMR\JELA\nmr\jn-134\2\fid expt: <zgpg30>
transmitter freq.: 100.622830 MHz
time domain size: 65536 points
width: 23809.52 Hz = 236.6215 ppm = 0.363305 Hz/pt
number of scans: 2000

freq. of 0 ppm: 100.612816 MHz
processed size: 32768 complex points
LB: 1.000 GF: 0.0000
Hz/cm: 840.708 ppm/cm: 8.35504

Compound 2k

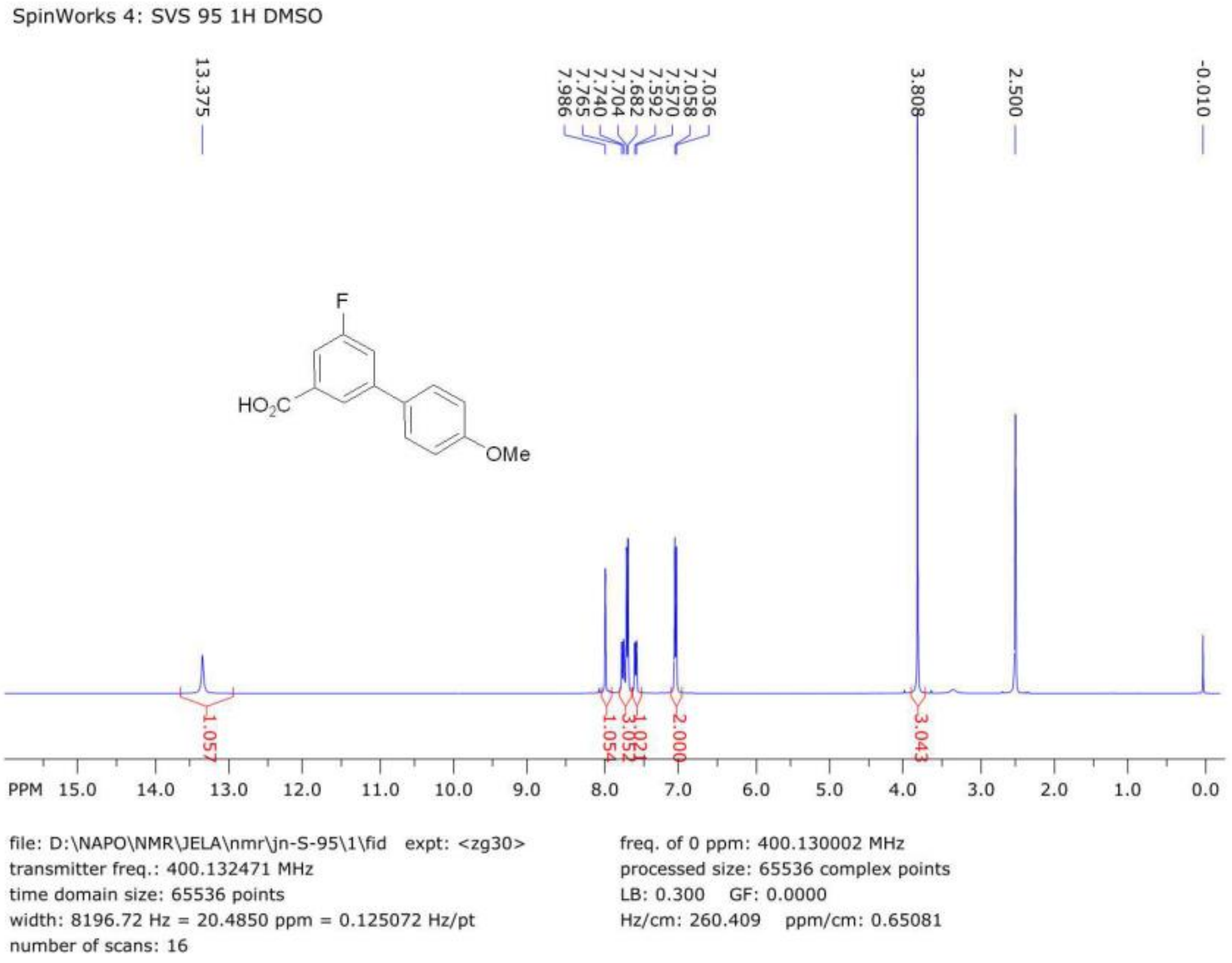
SpinWorks 4: SVS 95 1H DMSO
13.375
7.986
7.765
7.740
7.704
7.682
7.592
7.570
7.058
7.036
3.808
2.500
-0.010
F
HO2C
OMe
1.057
1.054
3.052
1.021
2.000
3.043
PPM 15.0 14.0 13.0 12.0 11.0 10.0 9.0 8.0 7.0 6.0 5.0 4.0 3.0 2.0 1.0 0.0
file: D:\NAPO\NMR\JELA\nmr\jn-S-95\1\fid expt: <zg30>
transmitter freq.: 400.132471 MHz
time domain size: 65536 points
width: 8196.72 Hz = 20.4850 ppm = 0.125072 Hz/pt
number of scans: 16
freq. of 0 ppm: 400.130002 MHz
processed size: 65536 complex points
LB: 0.300 GF: 0.0000
Hz/cm: 260.409 ppm/cm: 0.65081

Compound 2k

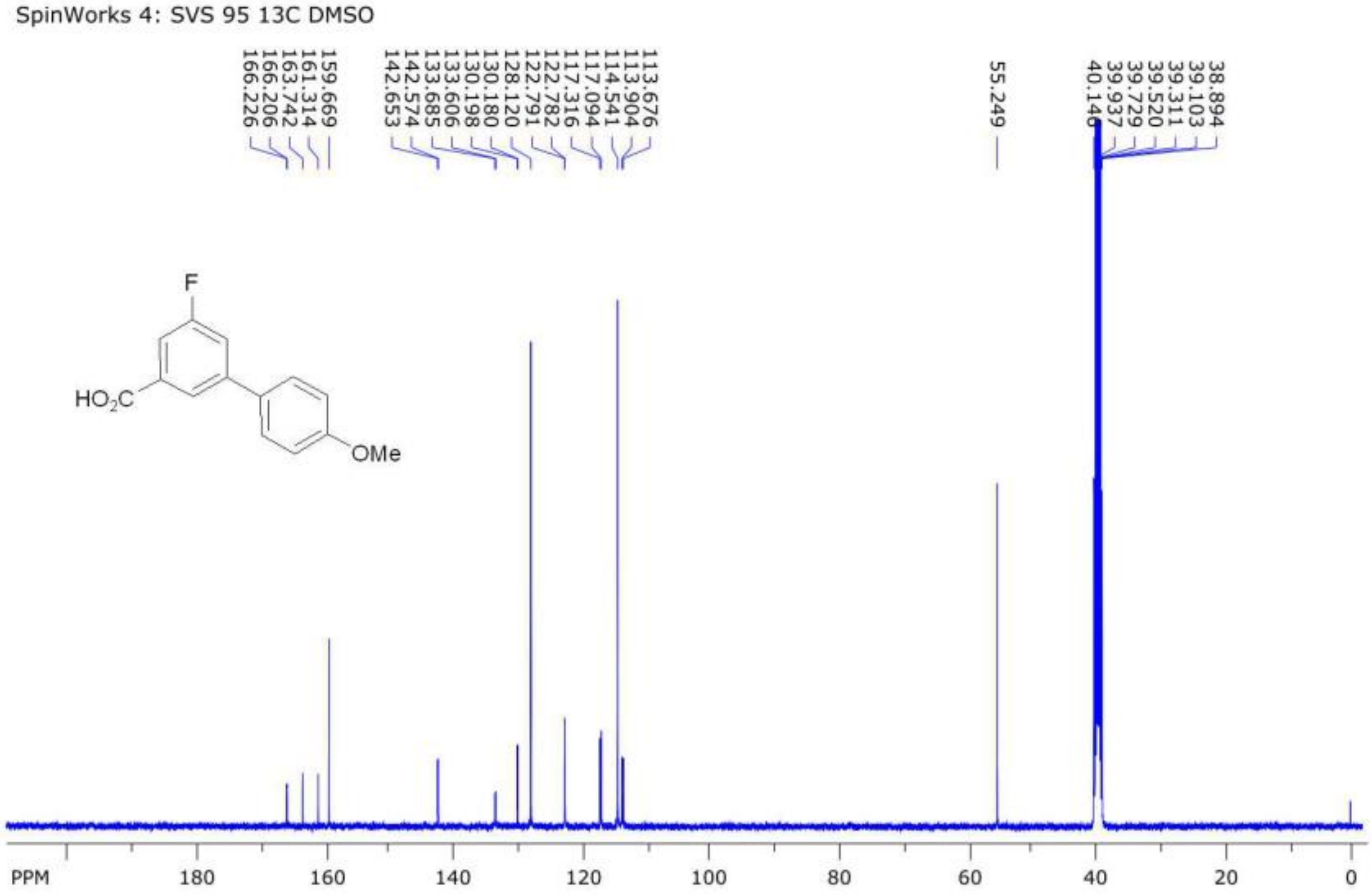

file: D:\NAPO\NMR\JELA\nmr\jn-S-95\2\fid expt: <zgpg30>
transmitter freq.: 100.622830 MHz
time domain size: 65536 points
width: 23809.52 Hz = 236.6215 ppm = 0.363305 Hz/pt
number of scans: 3500

freq. of 0 ppm: 100.612814 MHz
processed size: 32768 complex points
LB: 1.000 GF: 0.0000
Hz/cm: 852.297 ppm/cm: 8.47021

Compound 2l

SpinWorks 4: SVS 93 1H CDCl3

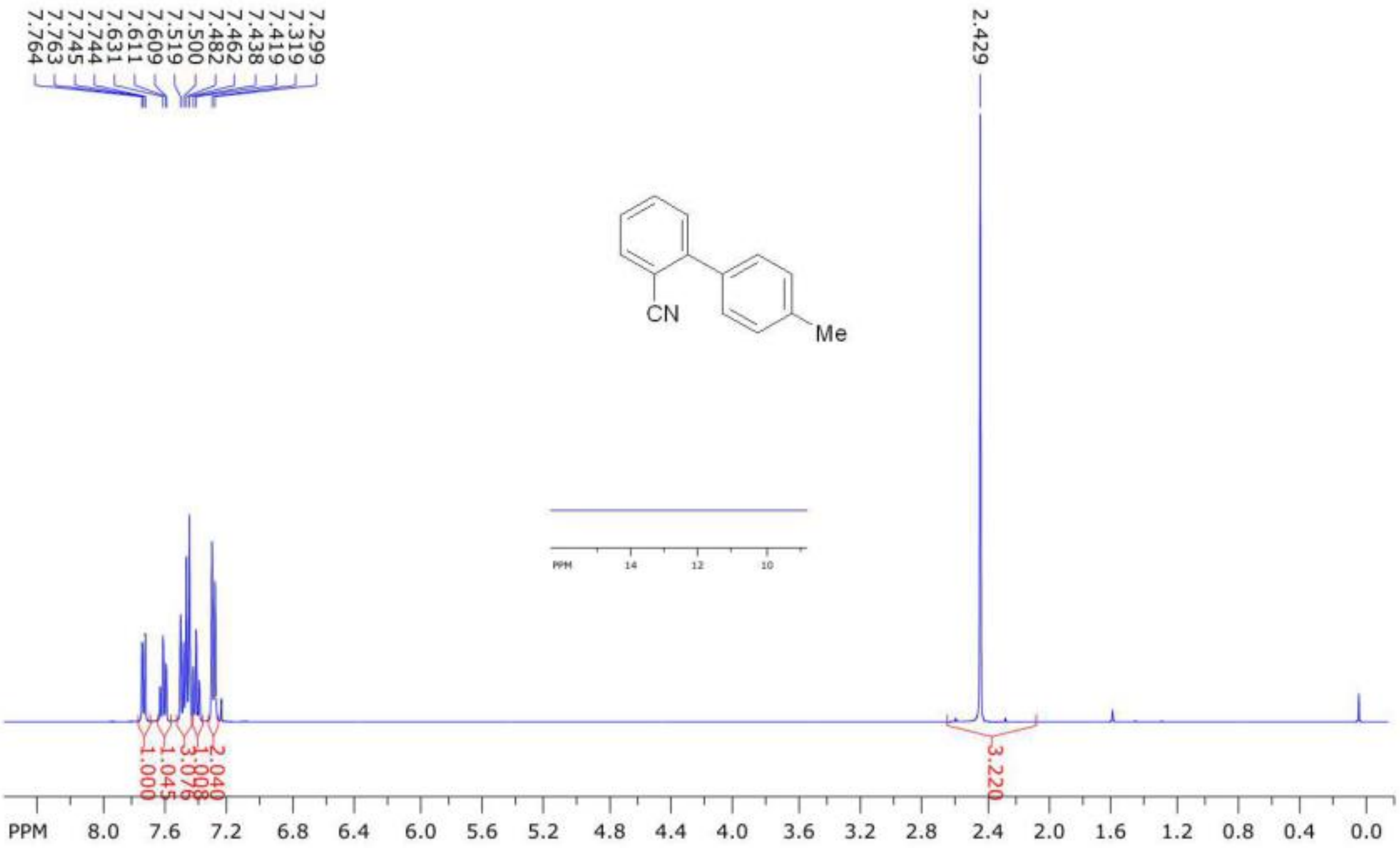

file: D:\NAPO\NMR\JELA\nmr\jn-S-93\1\fid expt: <zg30>
transmitter freq.: 400.132471 MHz
time domain size: 65536 points
width: 8196.72 Hz = 20.4850 ppm = 0.125072 Hz/pt
number of scans: 16

freq. of 0 ppm: 400.130009 MHz
processed size: 65536 complex points
LB: 0.300 GF: 0.0000
Hz/cm: 141.085 ppm/cm: 0.35260

Compound 2l

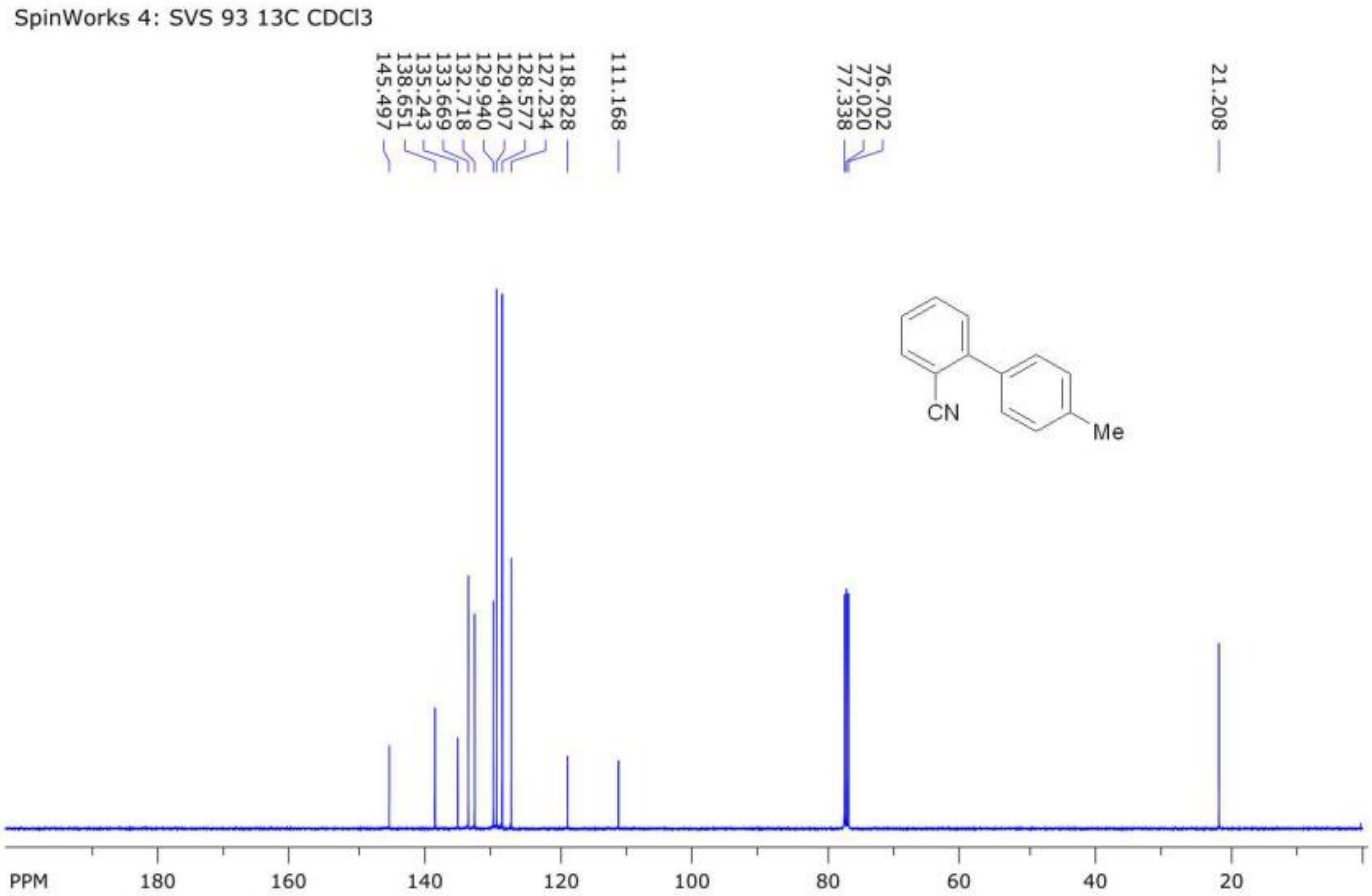

file: D:\NAPO\NMR\JELA\nmr\jn-S-93\3\fid expt: <zgpg30>
transmitter freq.: 100.622830 MHz
time domain size: 65536 points
width: 23809.52 Hz = 236.6215 ppm = 0.363305 Hz/pt
number of scans: 3500

freq. of 0 ppm: 100.612774 MHz
processed size: 32768 complex points
LB: 1.000 GF: 0.0000
Hz/cm: 818.584 ppm/cm: 8.13517

Compound 2m

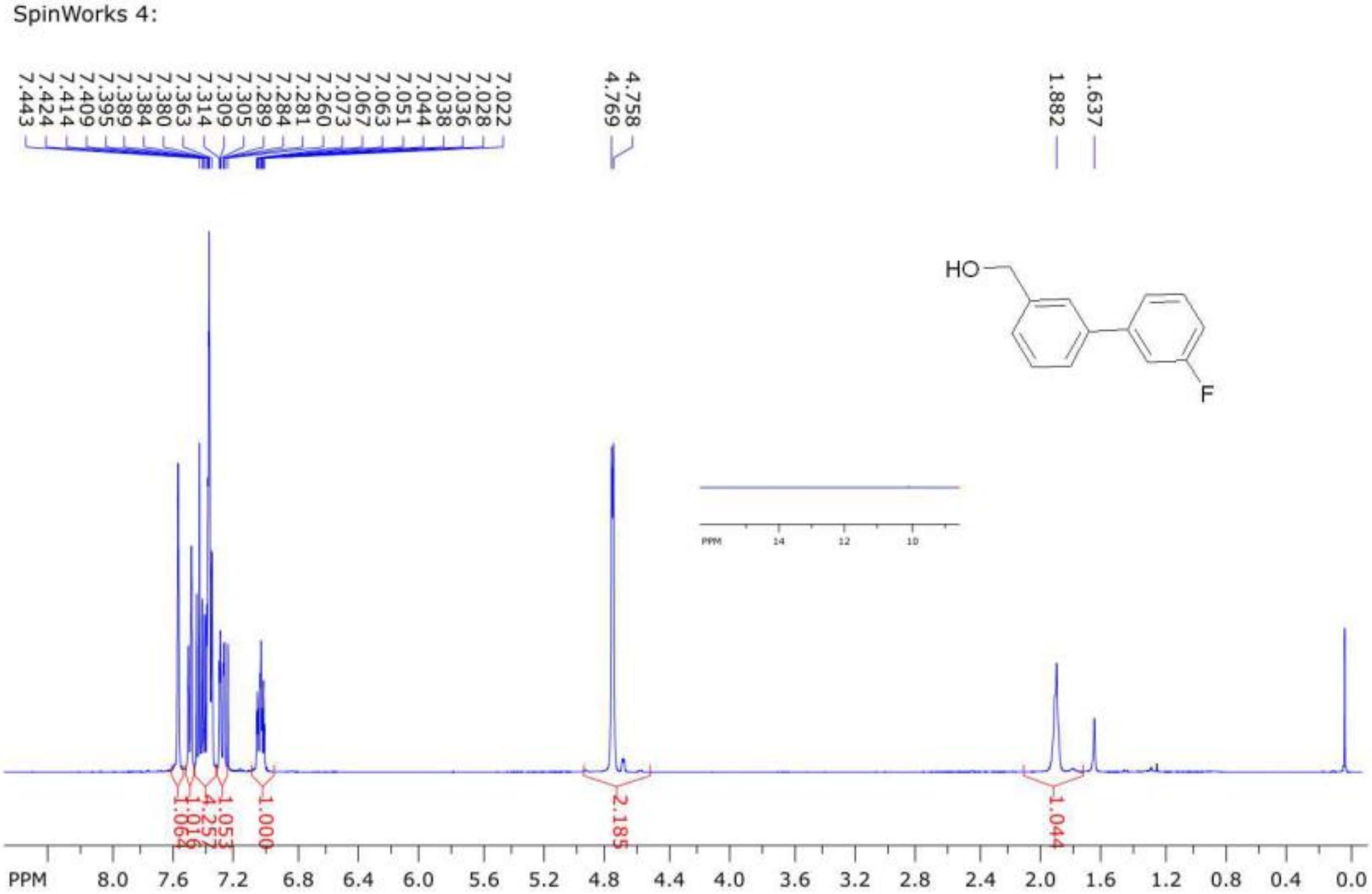

file: D:\NAPO\NMR\JELA\nmr\jn-S-80\1\fid expt: <zg30>
transmitter freq.: 400.132471 MHz
time domain size: 65536 points
width: 8196.72 Hz = 20.4850 ppm = 0.125072 Hz/pt
number of scans: 16

freq. of 0 ppm: 400.130009 MHz
processed size: 65536 complex points
LB: 0.300 GF: 0.0000
Hz/cm: 141.085 ppm/cm: 0.35260

Compound 2m

SpinWorks 4: SVS 80 13C CDCl3

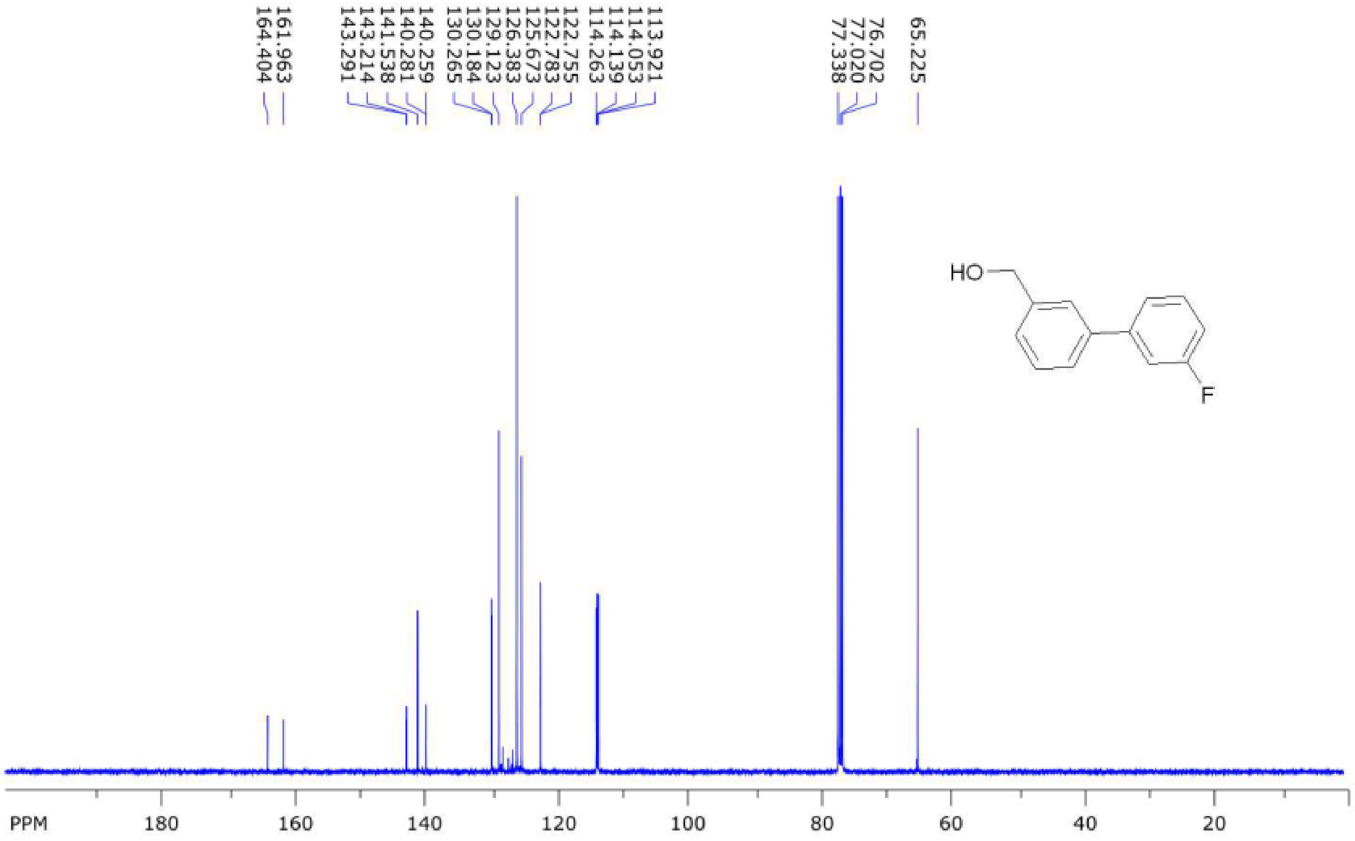

file: D:\NAPO\NMR\JELA\nmr\jn-S-80\2\fid expt: <zgpg30>
transmitter freq.: 100.622830 MHz
time domain size: 65536 points
width: 23809.52 Hz = 236.6215 ppm = 0.363305 Hz/pt
number of scans: 3500

freq. of 0 ppm: 100.612770 MHz
processed size: 32768 complex points
LB: 1.000 GF: 0.0000
Hz/cm: 821.745 ppm/cm: 8.16658

Compound 2n

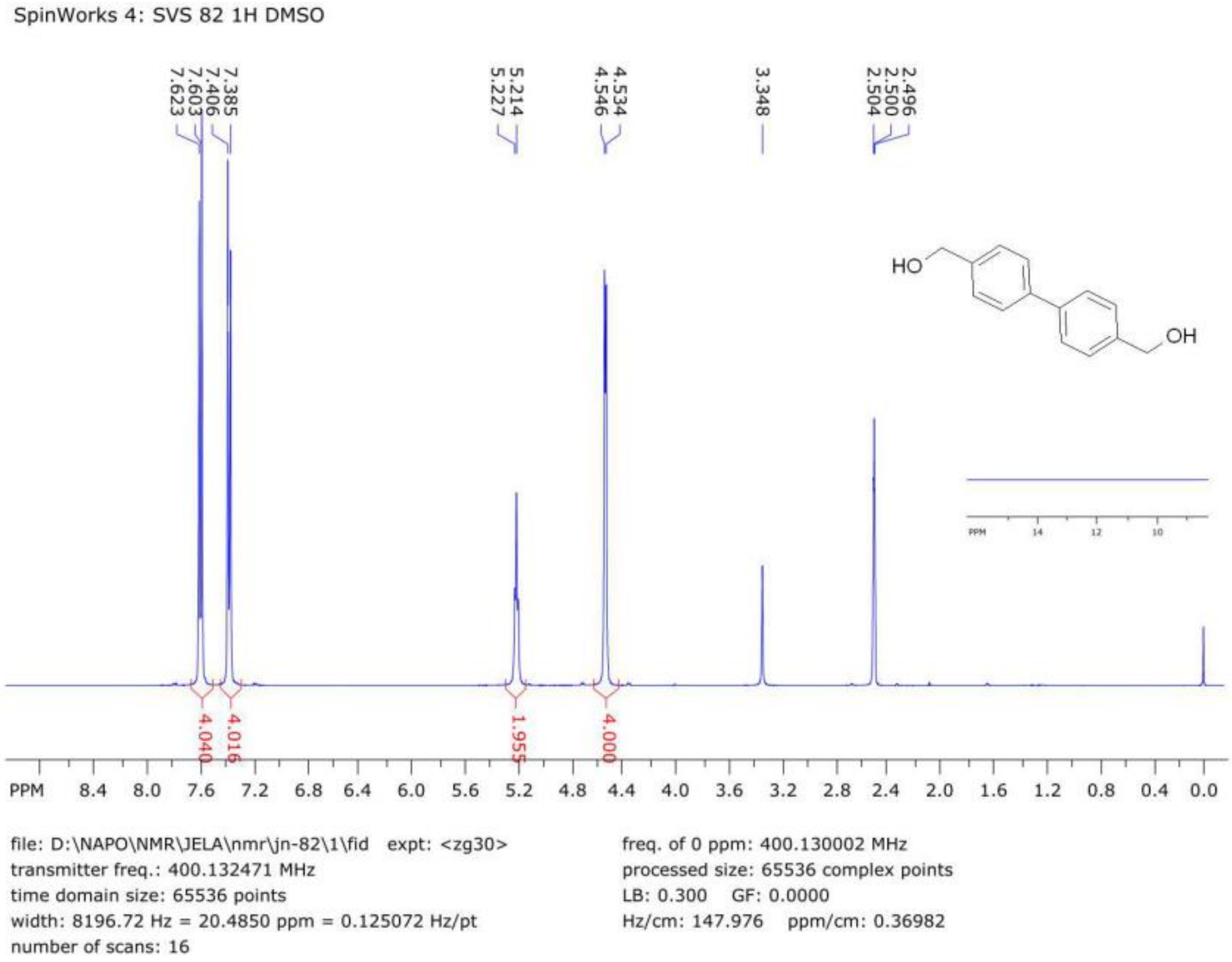
SpinWorks 4: SVS 82 1H DMSO
7.623
7.603
7.406
7.385
5.227
5.214
4.546
4.534
3.348
2.504
2.500
2.496
HO
OH
4.040
4.016
1.955
4.000
PPM
8.4 8.0 7.6 7.2 6.8 6.4 6.0 5.6 5.2 4.8 4.4 4.0 3.6 3.2 2.8 2.4 2.0 1.6 1.2 0.8 0.4 0.0
file: D:\NAPO\NMR\JELA\nmr\jn-82\1\fid expt: <zg30>
transmitter freq.: 400.132471 MHz
time domain size: 65536 points
width: 8196.72 Hz = 20.4850 ppm = 0.125072 Hz/pt
number of scans: 16
freq. of 0 ppm: 400.130002 MHz
processed size: 65536 complex points
LB: 0.300 GF: 0.0000
Hz/cm: 147.976 ppm/cm: 0.36982

Compound 2n

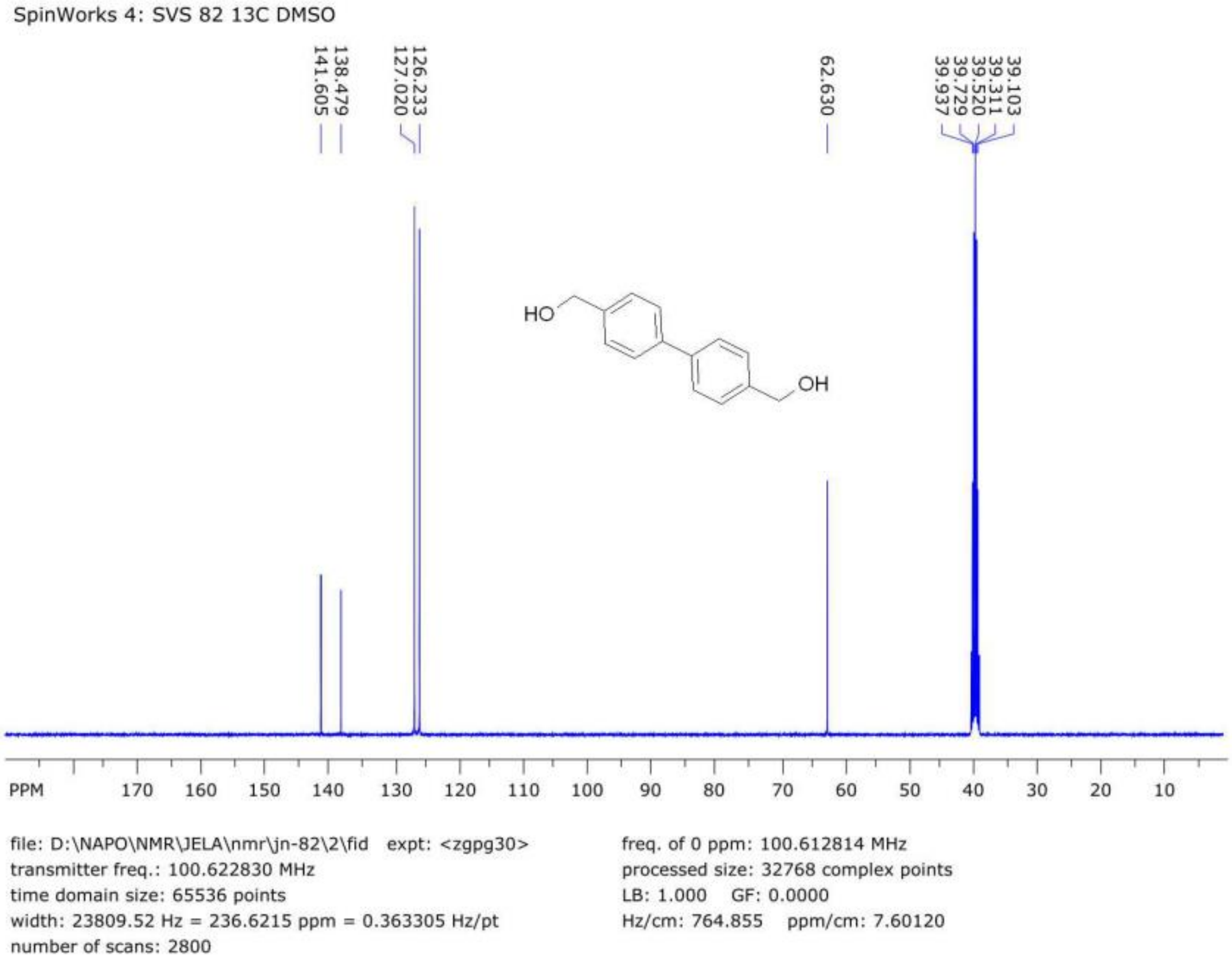

SpinWorks 4: SVS 82 13C DMSO
141.605
138.479
127.020
126.233
62.630
39.937
39.729
39.520
39.311
39.103
HO
OH
PPM
170
160
150
140
130
120
110
100
90
80
70
60
50
40
30
20
10
file: D:\NAPO\NMR\JELA\nmr\jn-82\2\fid expt: <zgpg30>
transmitter freq.: 100.622830 MHz
time domain size: 65536 points
width: 23809.52 Hz = 236.6215 ppm = 0.363305 Hz/pt
number of scans: 2800
freq. of 0 ppm: 100.612814 MHz
processed size: 32768 complex points
LB: 1.000 GF: 0.0000
Hz/cm: 764.855 ppm/cm: 7.60120

Compound 2o

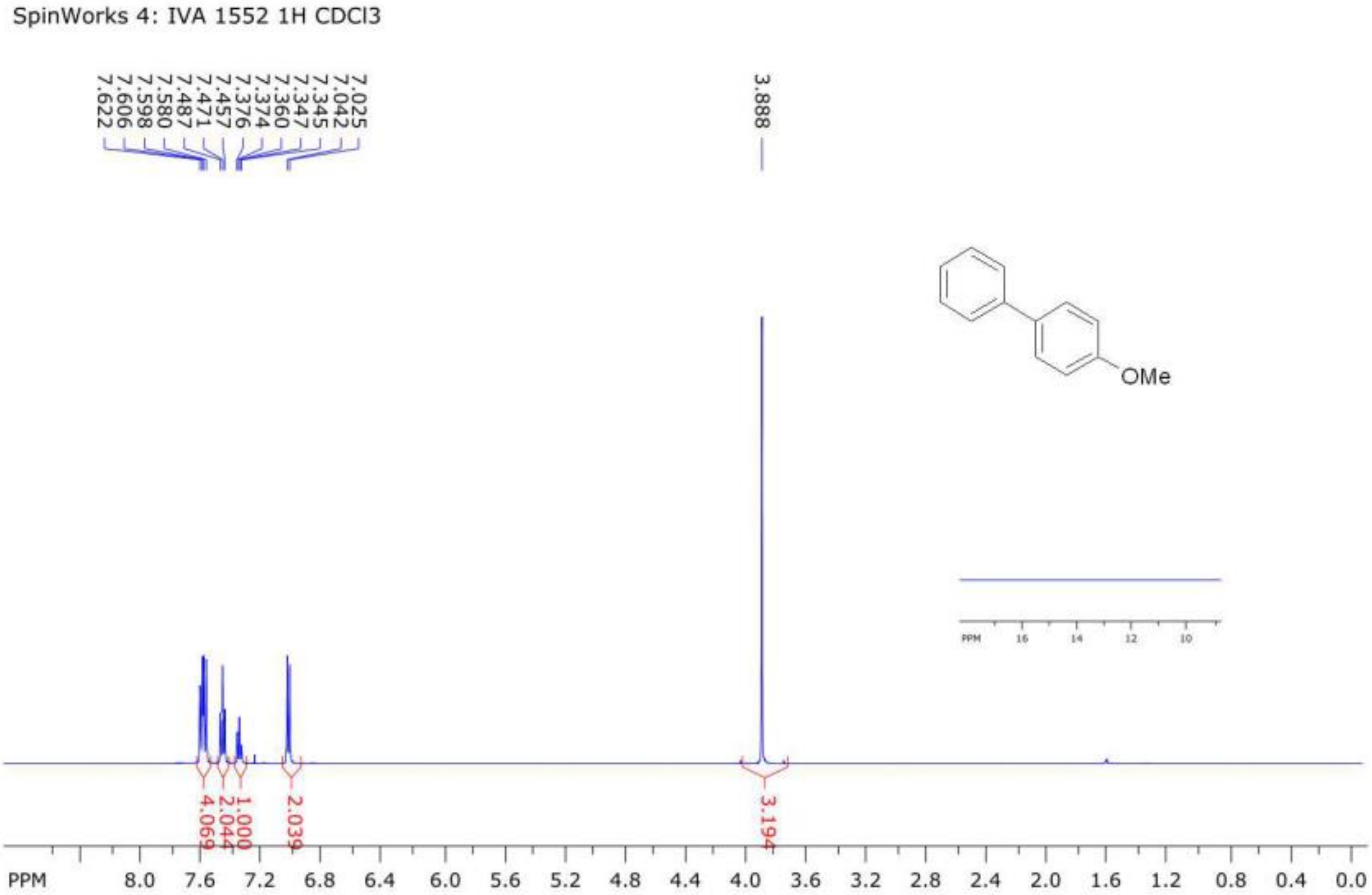

file: D:\NAPO\NMR\500-2\mkr23004\19\fid expt: <zg30>
transmitter freq.: 500.133001 MHz
time domain size: 65536 points
width: 12335.53 Hz = 24.6645 ppm = 0.188225 Hz/pt
number of scans: 24

freq. of 0 ppm: 500.130023 MHz
processed size: 65536 complex points
LB: 0.300 GF: 0.0000
Hz/cm: 180.666 ppm/cm: 0.36124

Compound 2o

SpinWorks 4: IVA 1552 13C CDCl3

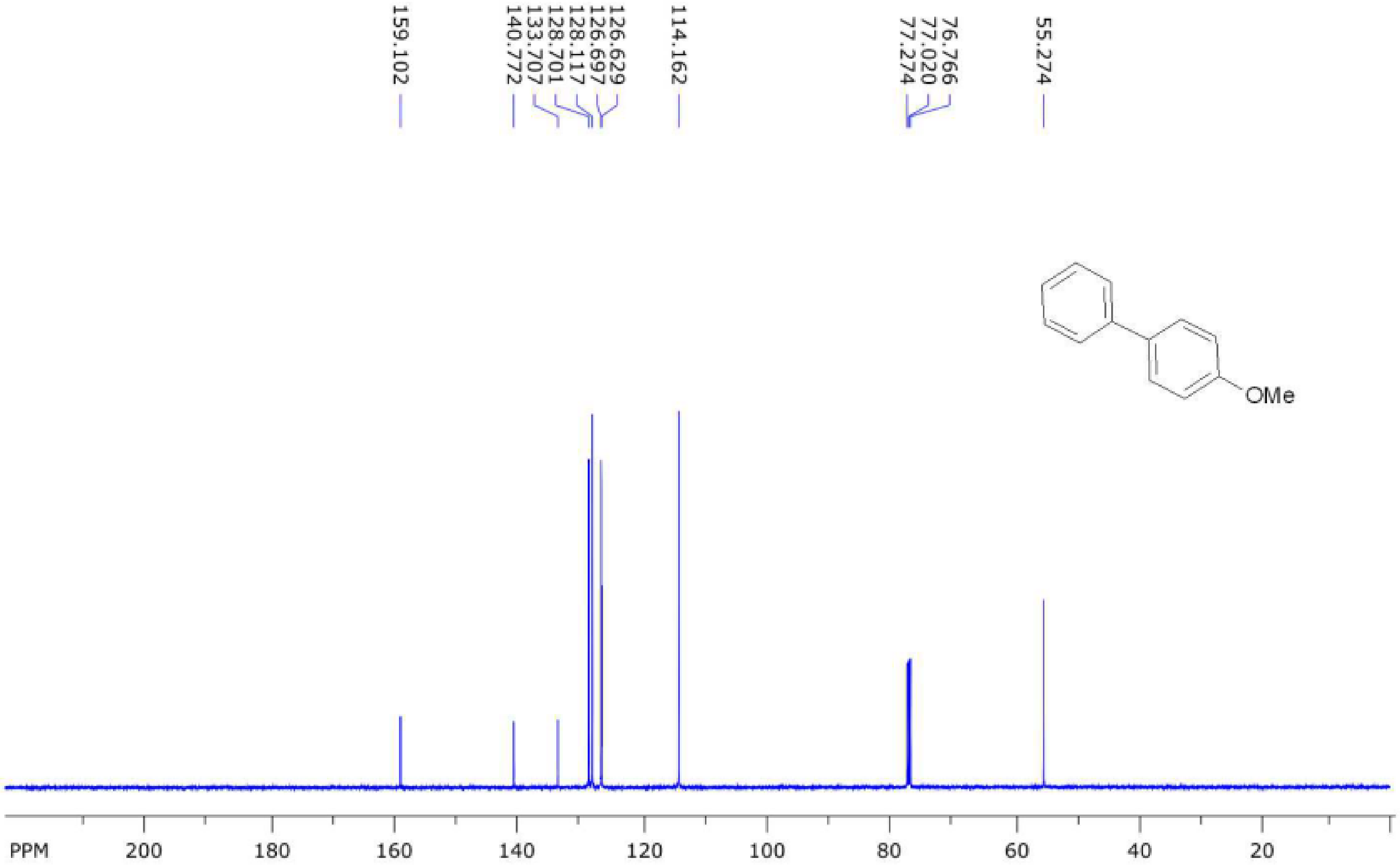

file: D:\NAPO\NMR\500-2\mkr23004\20\fid expt: <zgpg30>
transmitter freq.: 125.772879 MHz
time domain size: 65536 points
width: 36057.69 Hz = 286.6889 ppm = 0.550197 Hz/pt
number of scans: 512

freq. of 0 ppm: 125.757804 MHz
processed size: 32768 complex points
LB: 2.000 GF: 0.0000
Hz/cm: 1124.809 ppm/cm: 8.94317

Compound 2p

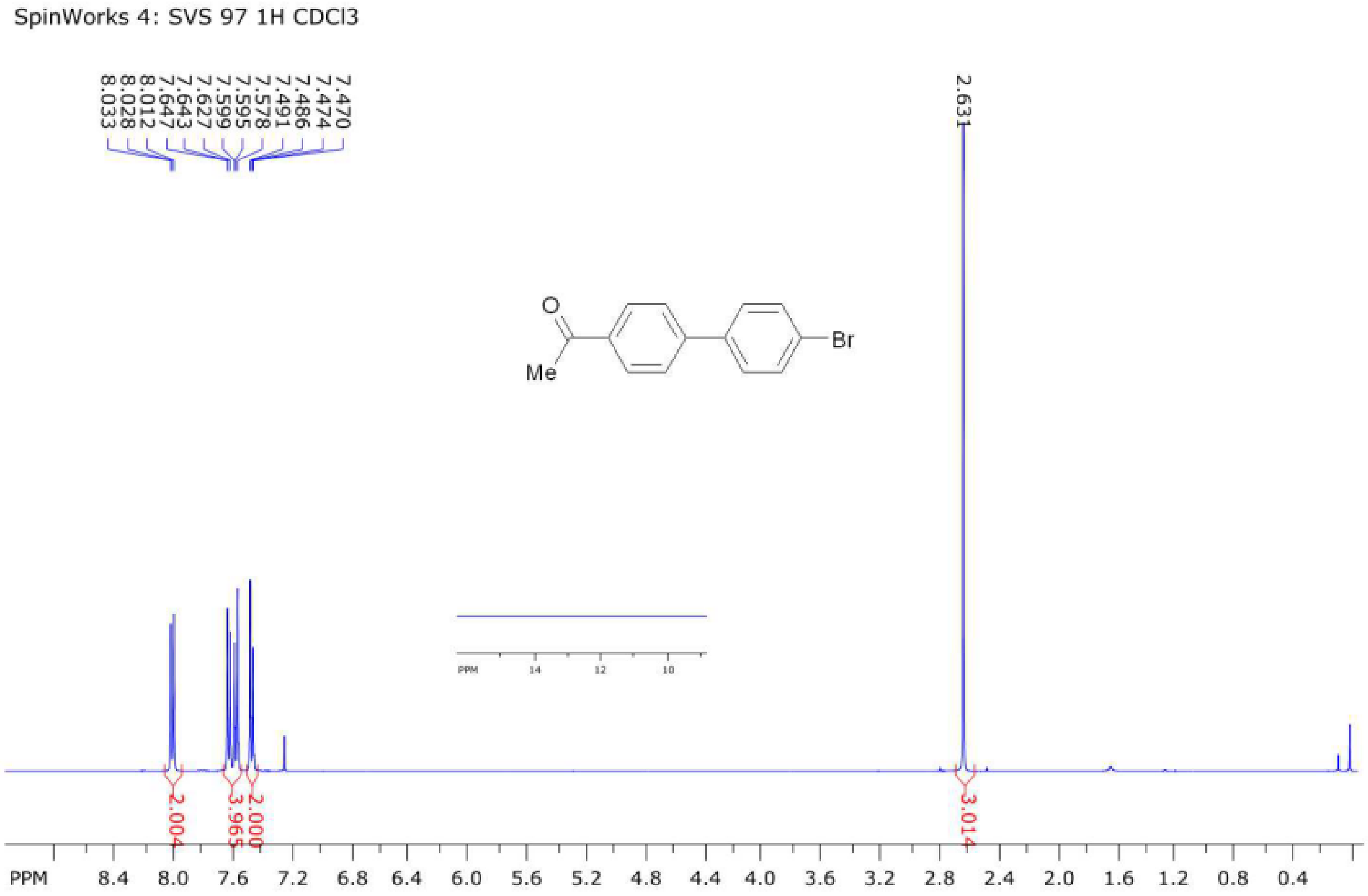

file: D:\NAPO\NMR\JELA\nmr\jn-S-97\1\fid expt: <zg30>
transmitter freq.: 400.132471 MHz
time domain size: 65536 points
width: 8196.72 Hz = 20.4850 ppm = 0.125072 Hz/pt
number of scans: 16

freq. of 0 ppm: 400.130010 MHz
processed size: 65536 complex points
LB: 0.300 GF: 0.0000
Hz/cm: 147.614 ppm/cm: 0.36891

Compound 2p

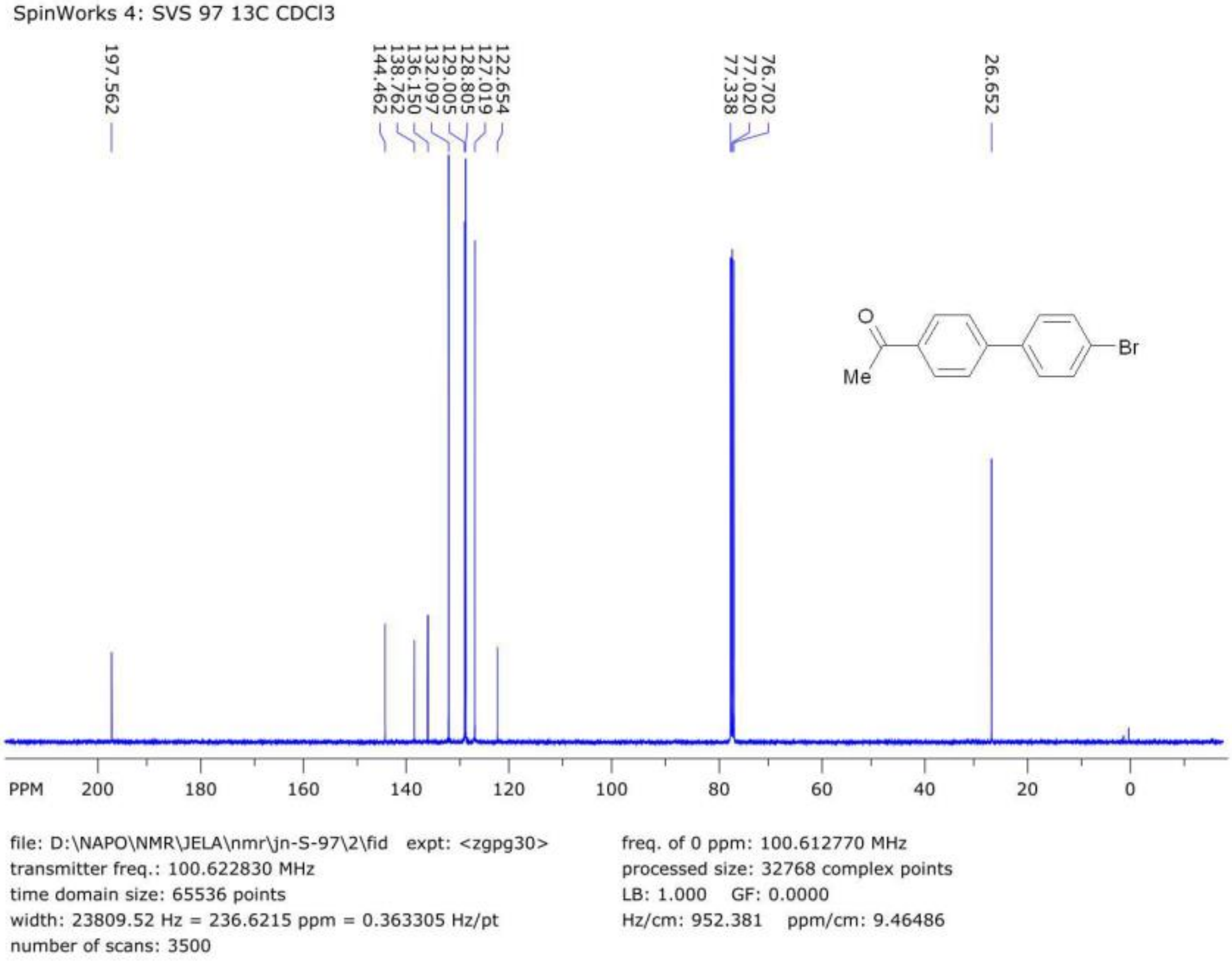
SpinWorks 4: SVS 97 13C CDCl3
197.562
144.462
138.762
136.150
132.097
129.005
128.805
127.019
122.654
77.338
77.020
76.702
26.652
O
Me
Br
PPM
200
180
160
140
120
100
80
60
40
20
0
file: D:\NAPO\NMR\JELA\nmr\jn-S-97\2\fid expt: <zgpg30>
transmitter freq.: 100.622830 MHz
time domain size: 65536 points
width: 23809.52 Hz = 236.6215 ppm = 0.363305 Hz/pt
number of scans: 3500
freq. of 0 ppm: 100.612770 MHz
processed size: 32768 complex points
LB: 1.000 GF: 0.0000
Hz/cm: 952.381 ppm/cm: 9.46486

Compound 2q

SpinWorks 4: IVA 1410 1H DMSO

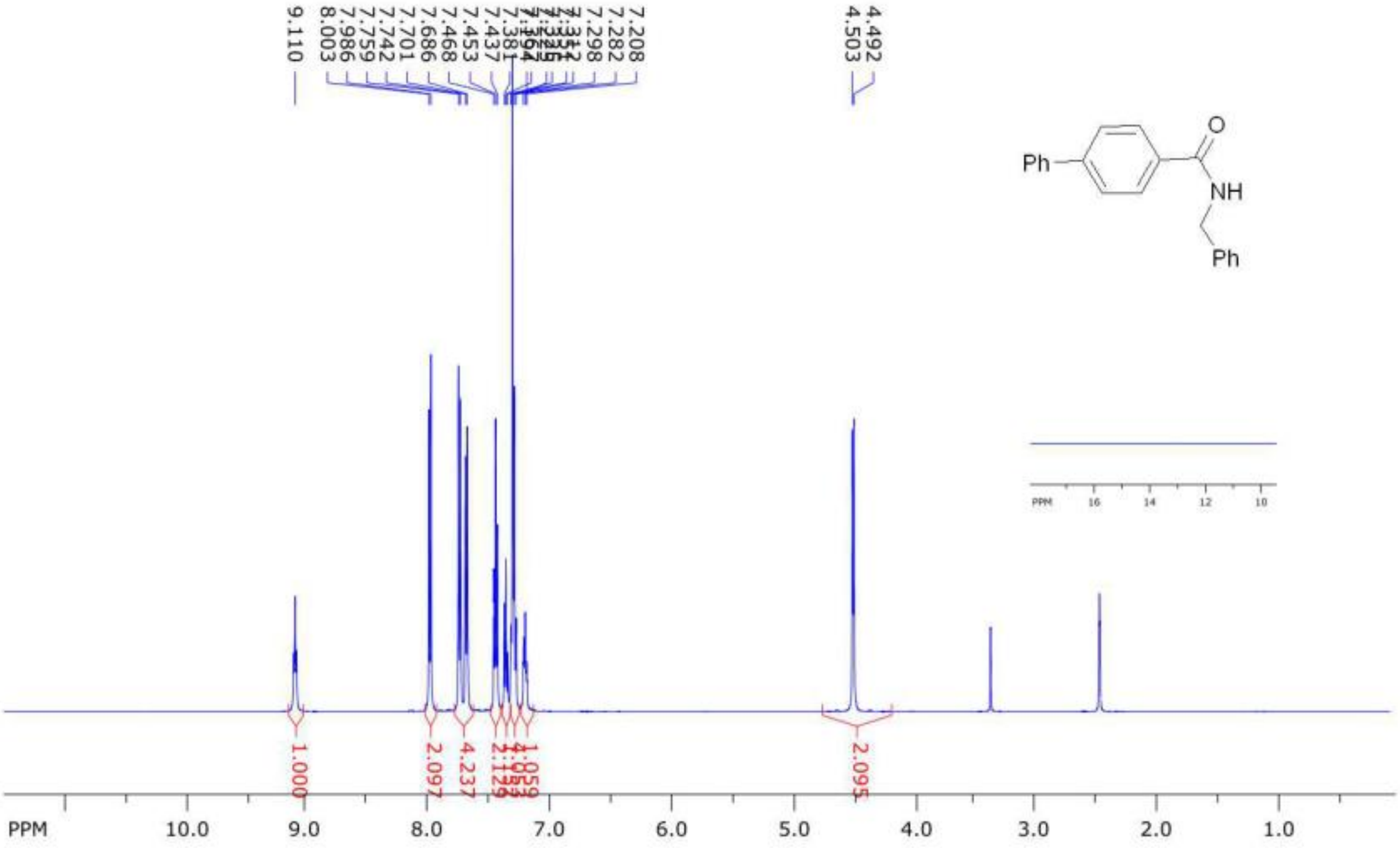

file: D:\NAPO\NMR\500-2\mkr20803\3\fid expt: <zg30>
transmitter freq.: 500.133001 MHz
time domain size: 65536 points
width: 12335.53 Hz = 24.6645 ppm = 0.188225 Hz/pt
number of scans: 24

freq. of 0 ppm: 500.130024 MHz
processed size: 65536 complex points
LB: 0.300 GF: 0.0000
Hz/cm: 229.244 ppm/cm: 0.45837

Compound 2q

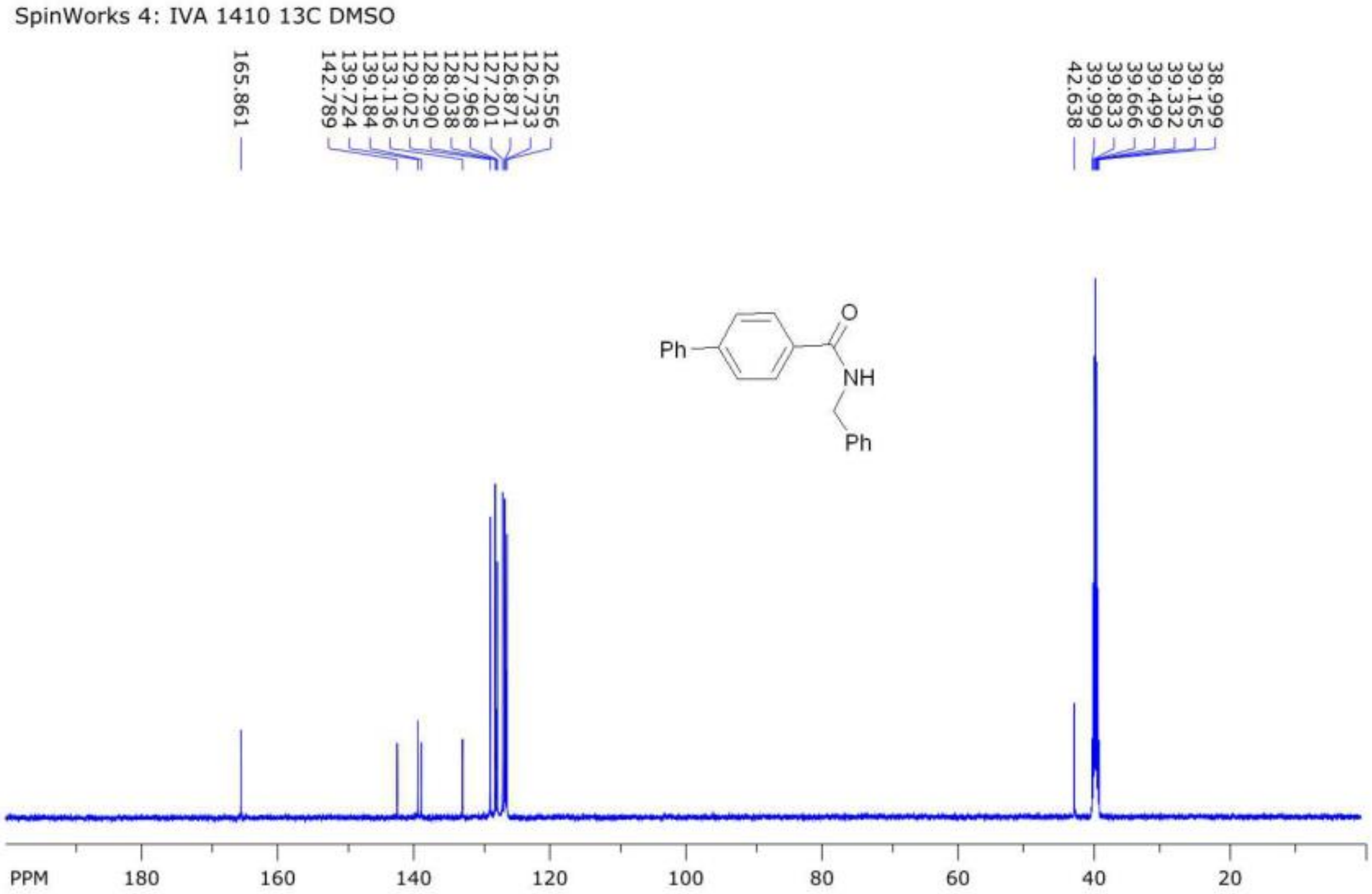

file: D:\NAPO\NMR\500-2\mkr20803\4\fid expt: <zgpg30>
transmitter freq.: 125.772879 MHz
time domain size: 65536 points
width: 36057.69 Hz = 286.6889 ppm = 0.550197 Hz/pt
number of scans: 512

freq. of 0 ppm: 125.757847 MHz
processed size: 32768 complex points
LB: 2.000 GF: 0.0000
Hz/cm: 1008.339 ppm/cm: 8.01714

Compound 2r

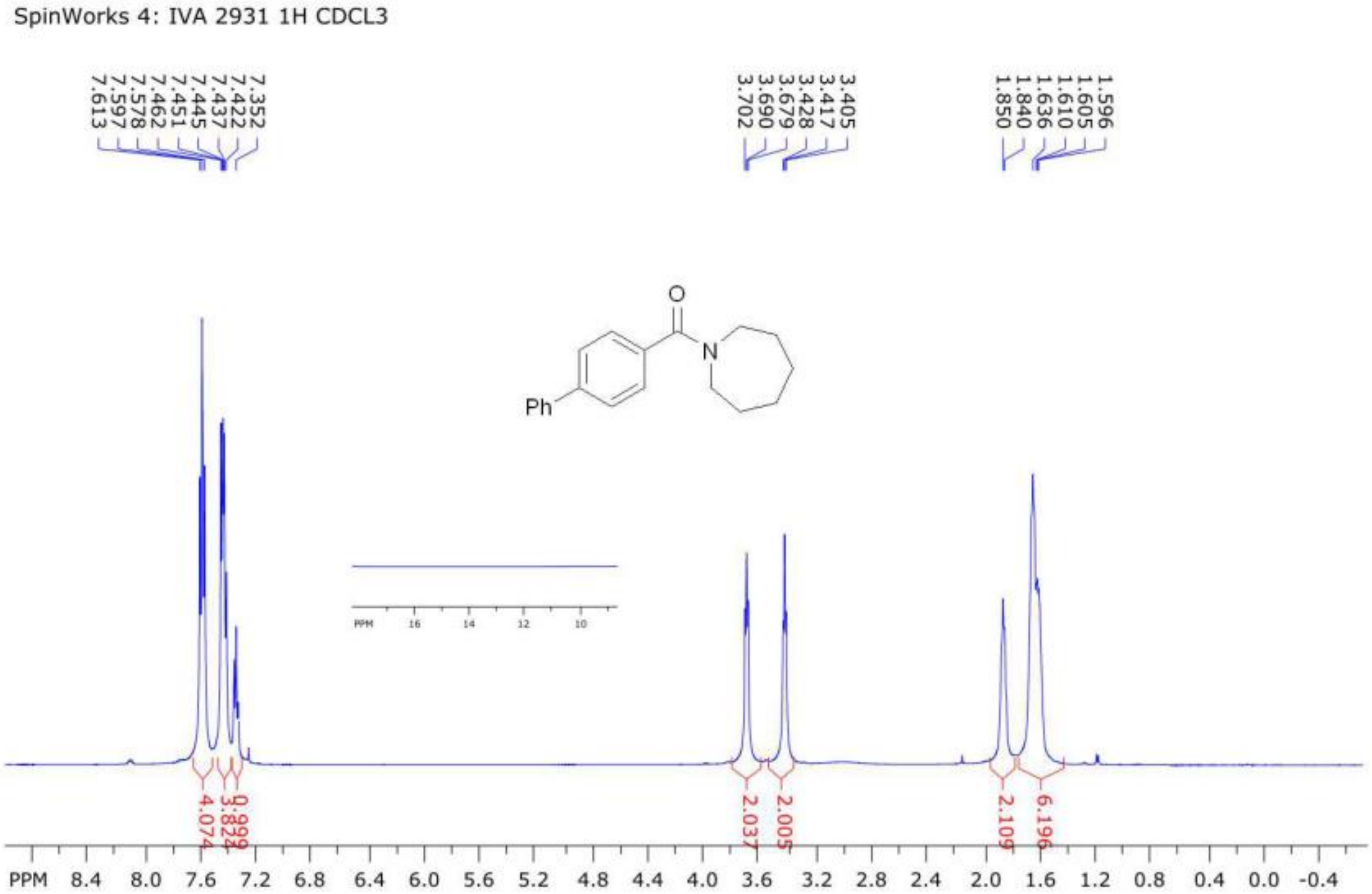

file: ...APO\NMR\500-2\mkr10706\21 2931\fid expt: <zg30>
transmitter freq.: 500.133001 MHz
time domain size: 65536 points
width: 12335.53 Hz = 24.6645 ppm = 0.188225 Hz/pt
number of scans: 24

freq. of 0 ppm: 500.130021 MHz
processed size: 65536 complex points
LB: 0.300 GF: 0.0000
Hz/cm: 194.858 ppm/cm: 0.38961

Compound 2r

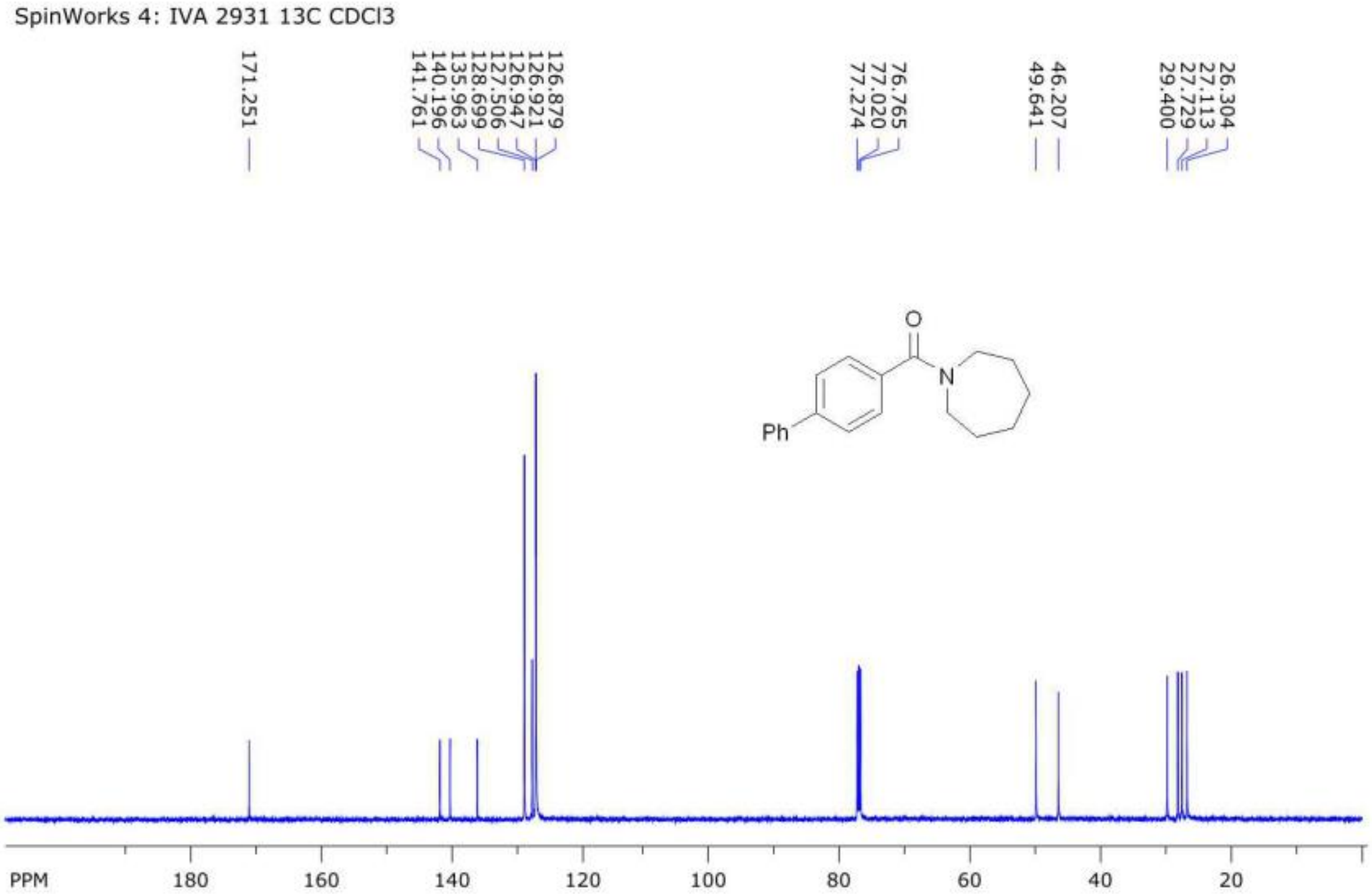

file: D:\NAPO\NMR\500-2\mkr10706\22\fid expt: <zgpg30>
transmitter freq.: 125.772879 MHz
time domain size: 65536 points
width: 36057.69 Hz = 286.6889 ppm = 0.550197 Hz/pt
number of scans: 512

freq. of 0 ppm: 125.757813 MHz
processed size: 32768 complex points
LB: 2.000 GF: 0.0000
Hz/cm: 1054.608 ppm/cm: 8.38502

Compound 2s

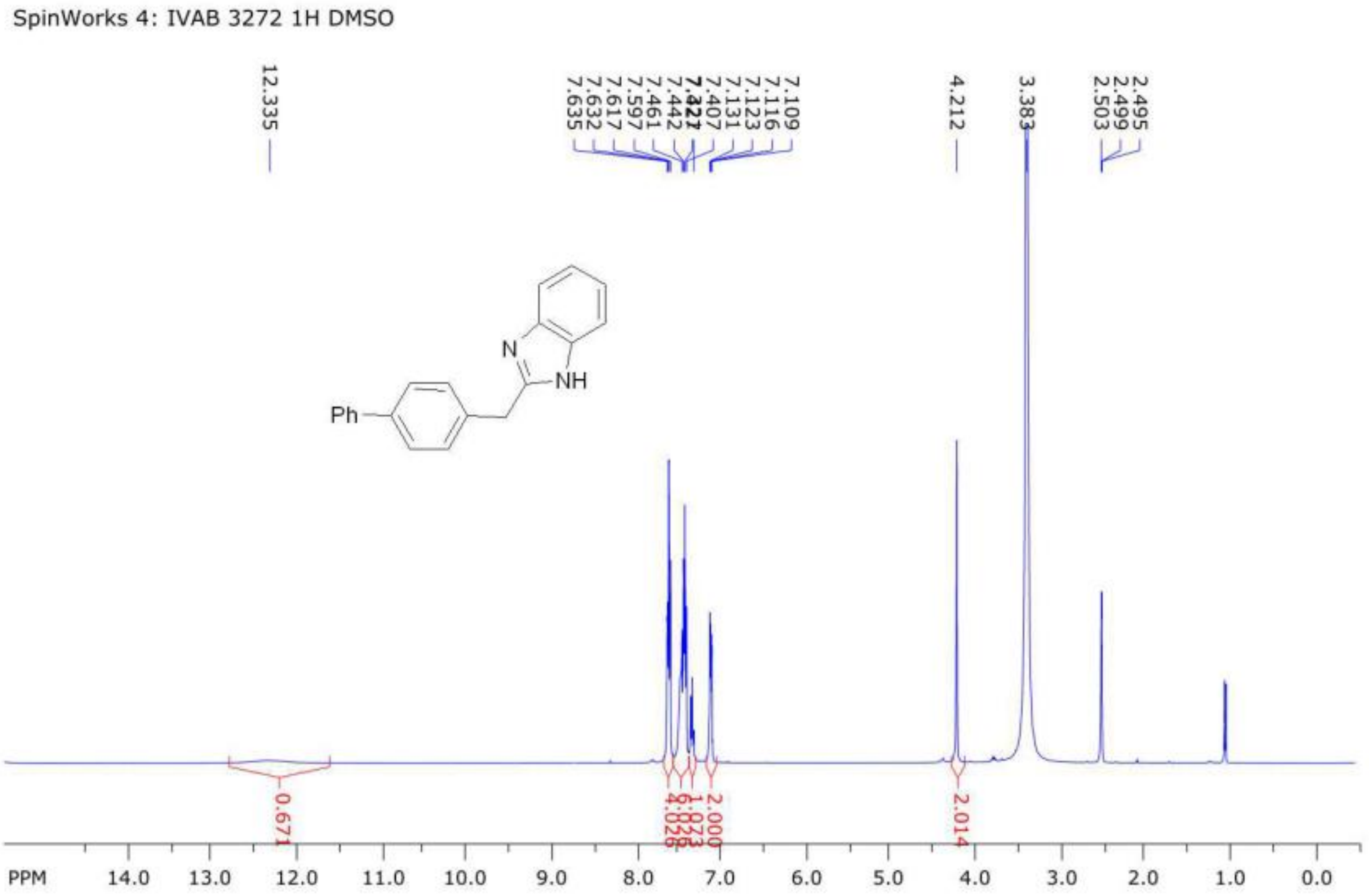

file: ...akia\19II2023FIDs\Ivab-3272\1H\fid expt: <zg30>
transmitter freq.: 400.133001 MHz
time domain size: 65536 points
width: 6393.86 Hz = 15.9793 ppm = 0.097563 Hz/pt
number of scans: 64

freq. of 0 ppm: 400.130003 MHz
processed size: 65536 complex points
LB: 0.300 GF: 0.0000
Hz/cm: 255.754 ppm/cm: 0.63917

Compound 2s

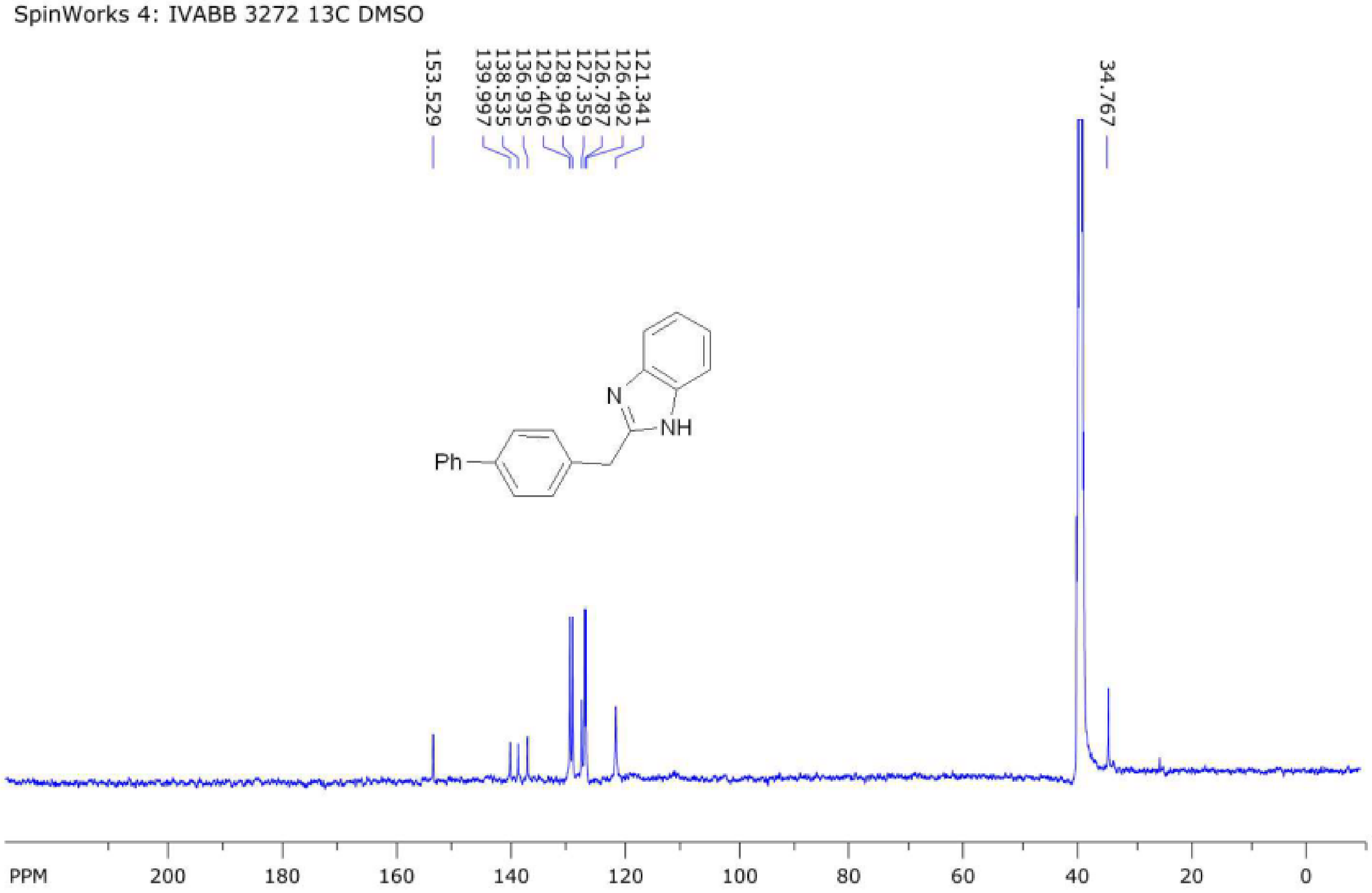

file: ...kia\19II2023FIDs\Ivab-3272\13C\fid expt: <zgpg30>
transmitter freq.: 100.623836 MHz
time domain size: 65536 points
width: 24038.46 Hz = 238.8943 ppm = 0.366798 Hz/pt
number of scans: 2135

freq. of 0 ppm: 100.612812 MHz
processed size: 32768 complex points
LB: 10.000 GF: 0.0000
Hz/cm: 961.538 ppm/cm: 9.55577

Compound 2t

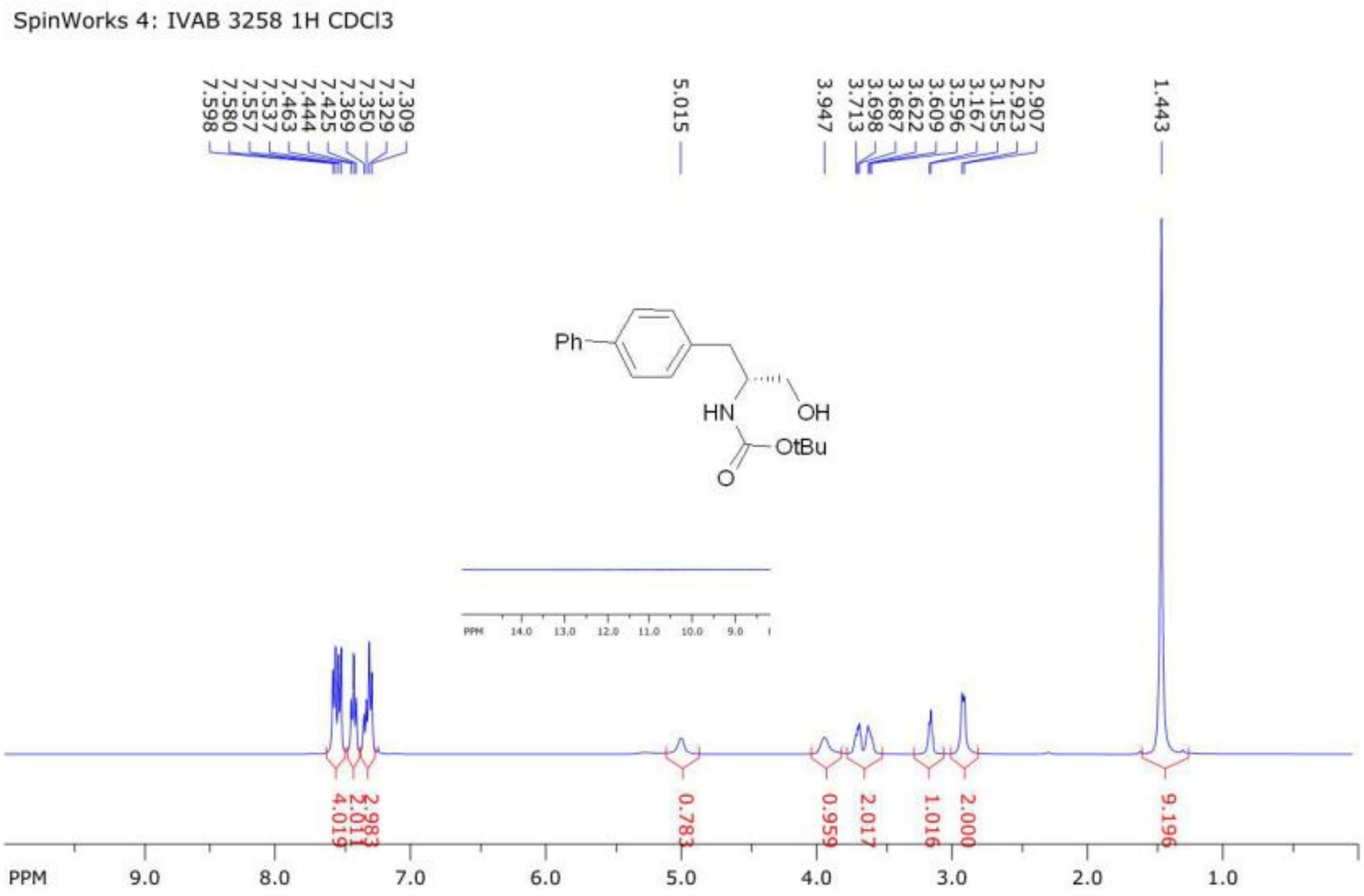

file: ...akia\19II2023FIDs\Ivab-3258\1H\fid expt: <zg30>
transmitter freq.: 400.133001 MHz
time domain size: 65536 points
width: 6393.86 Hz = 15.9793 ppm = 0.097563 Hz/pt
number of scans: 64

freq. of 0 ppm: 400.130008 MHz
processed size: 65536 complex points
LB: 0.300 GF: 0.0000
Hz/cm: 160.412 ppm/cm: 0.40090

Compound 2t

SpinWorks 4: IVAB 3258 13C CDCl3

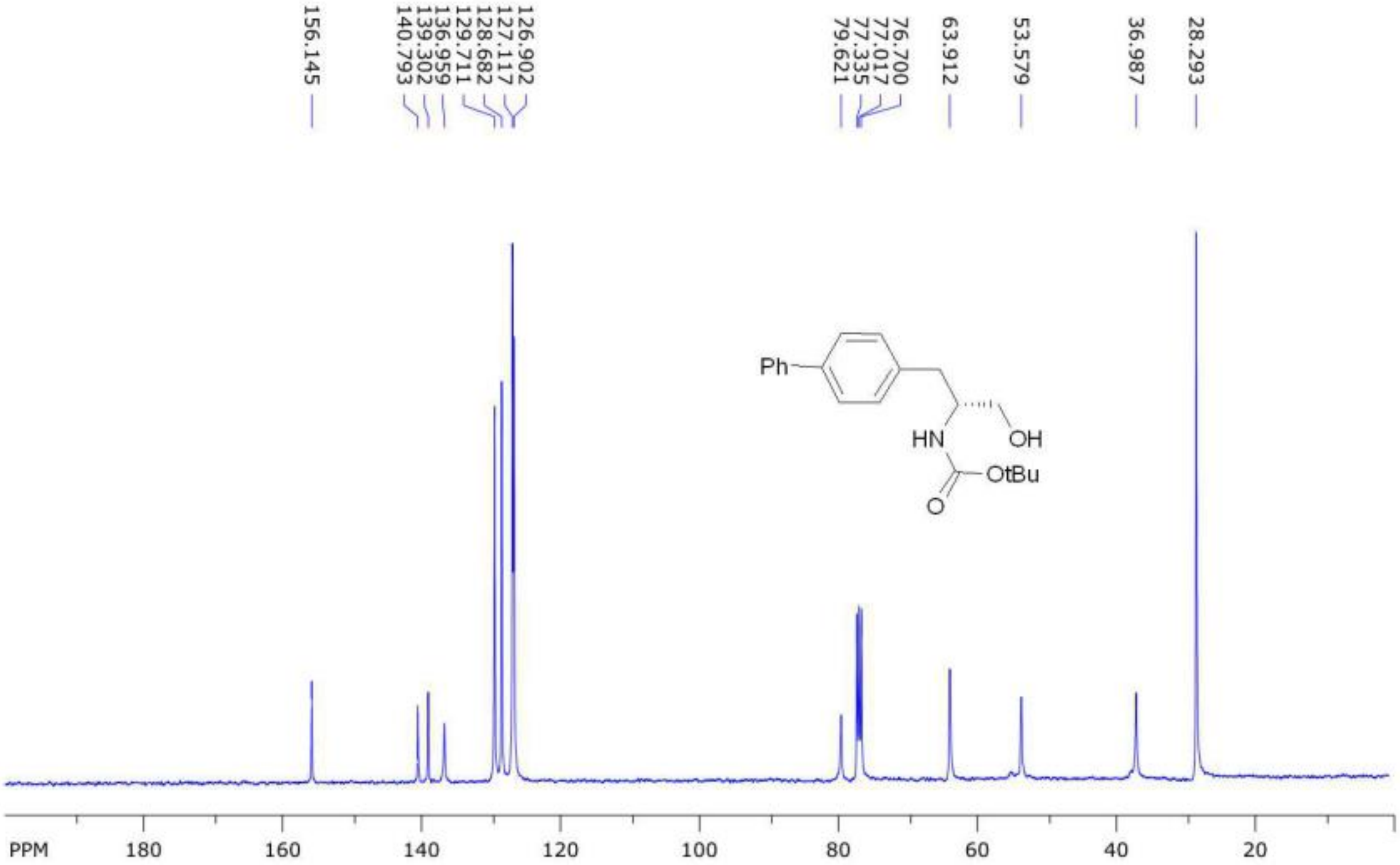

file: ...kia\19II2023FIDs\Ivab-3258\13C\fid expt: <zgpg30>
transmitter freq.: 100.623836 MHz
time domain size: 65536 points
width: 24038.46 Hz = 238.8943 ppm = 0.366798 Hz/pt
number of scans: 747

freq. of 0 ppm: 100.612780 MHz
processed size: 32768 complex points
LB: 10.000 GF: 0.0000
Hz/cm: 805.182 ppm/cm: 8.00190

Compound 2u

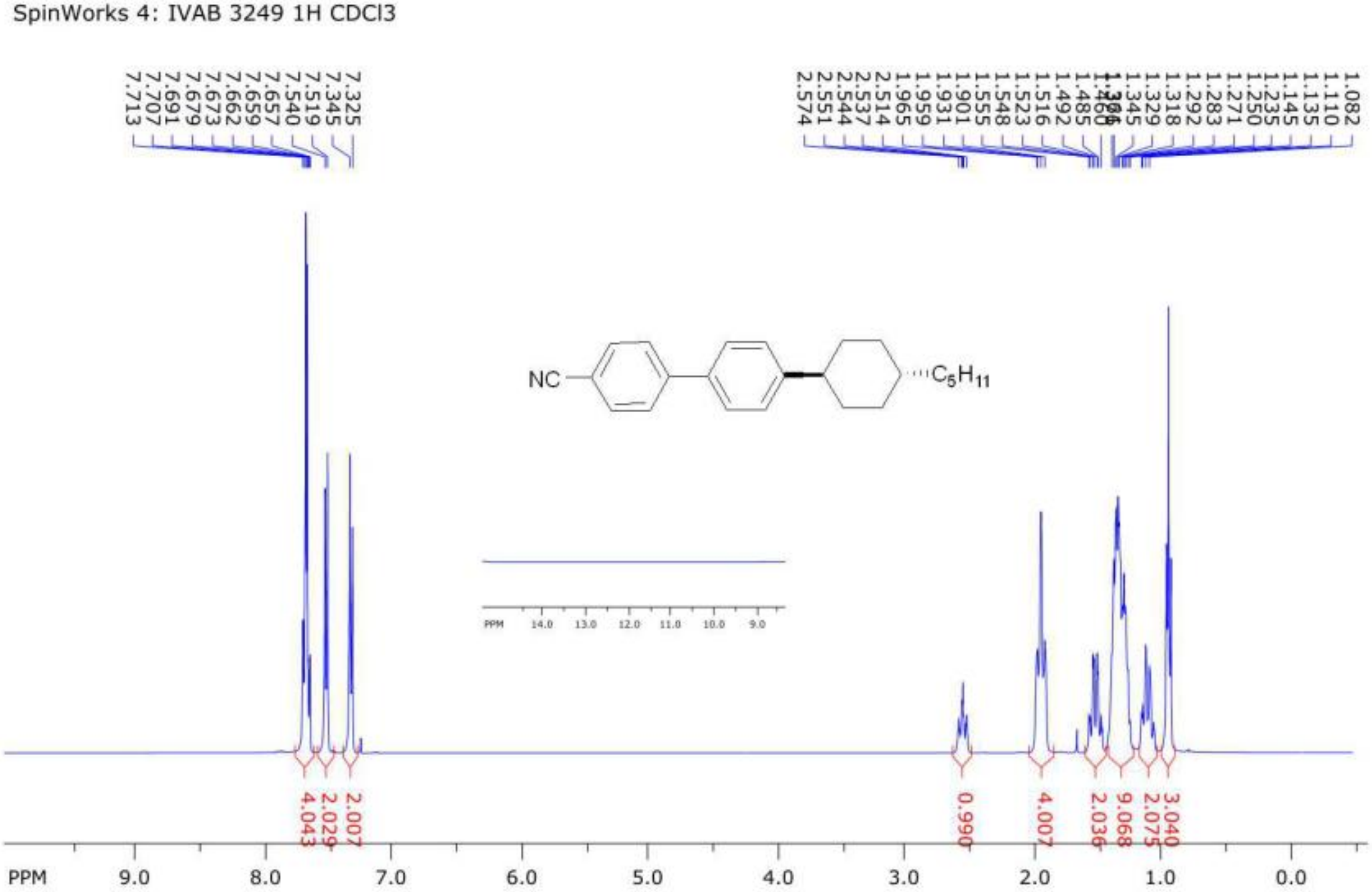

file: ...akia\19II2023FIDs\Ivab-3249\1H\fid expt: <zg30>
transmitter freq.: 400.133001 MHz
time domain size: 65536 points
width: 6393.86 Hz = 15.9793 ppm = 0.097563 Hz/pt
number of scans: 64

freq. of 0 ppm: 400.130010 MHz
processed size: 65536 complex points
LB: 0.300 GF: 0.0000
Hz/cm: 170.314 ppm/cm: 0.42564

Compound 2u

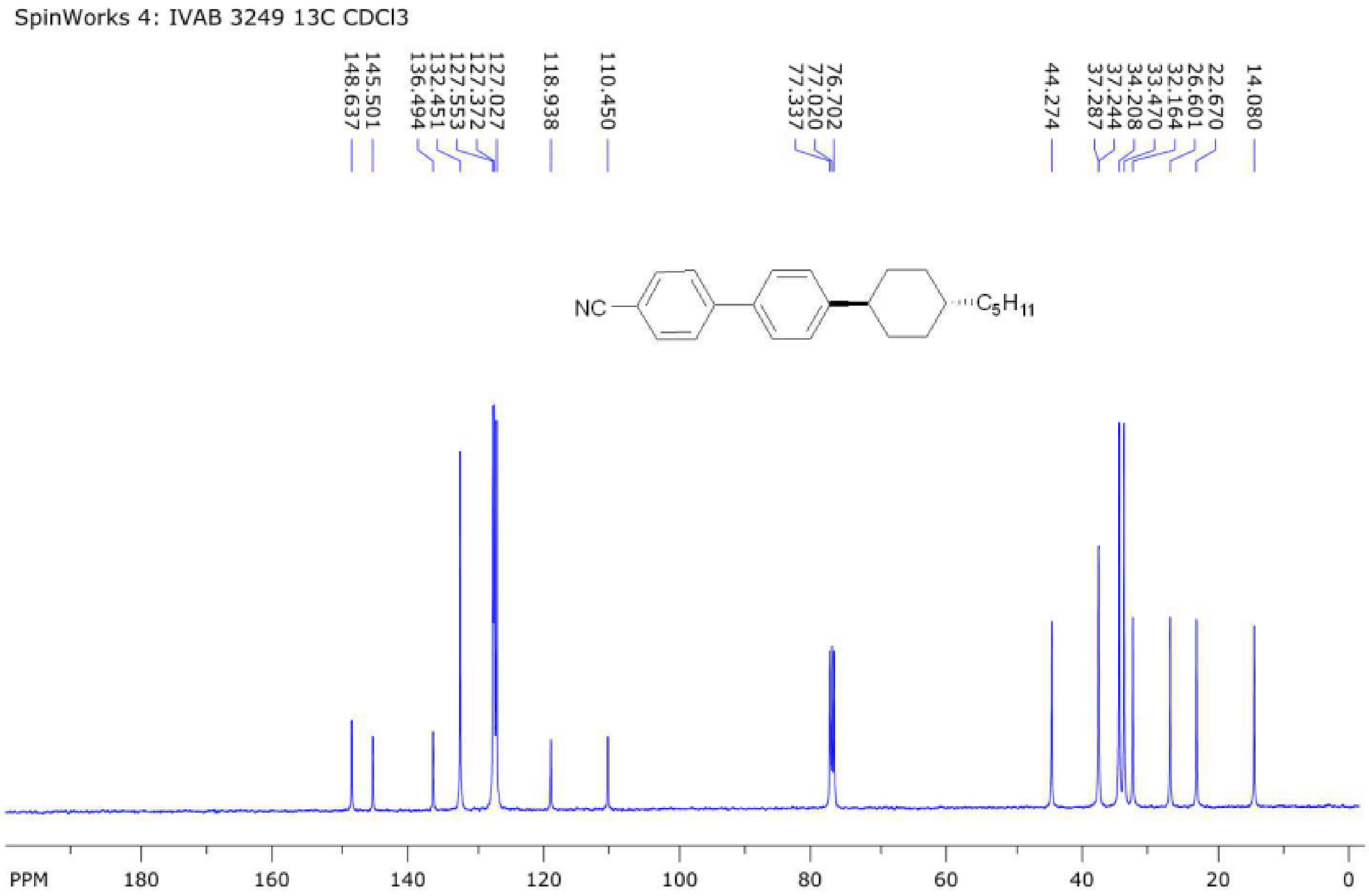

file: ...kia\19II2023FIDs\Ivab-3249\13C\fid expt: <zgpg30>
transmitter freq.: 100.623836 MHz
time domain size: 65536 points
width: 24038.46 Hz = 238.8943 ppm = 0.366798 Hz/pt
number of scans: 873

freq. of 0 ppm: 100.612779 MHz
processed size: 32768 complex points
LB: 10.000 GF: 0.0000
Hz/cm: 812.628 ppm/cm: 8.07590

Compound 2v

SpinWorks 4: IVA 1516 1H DMSO

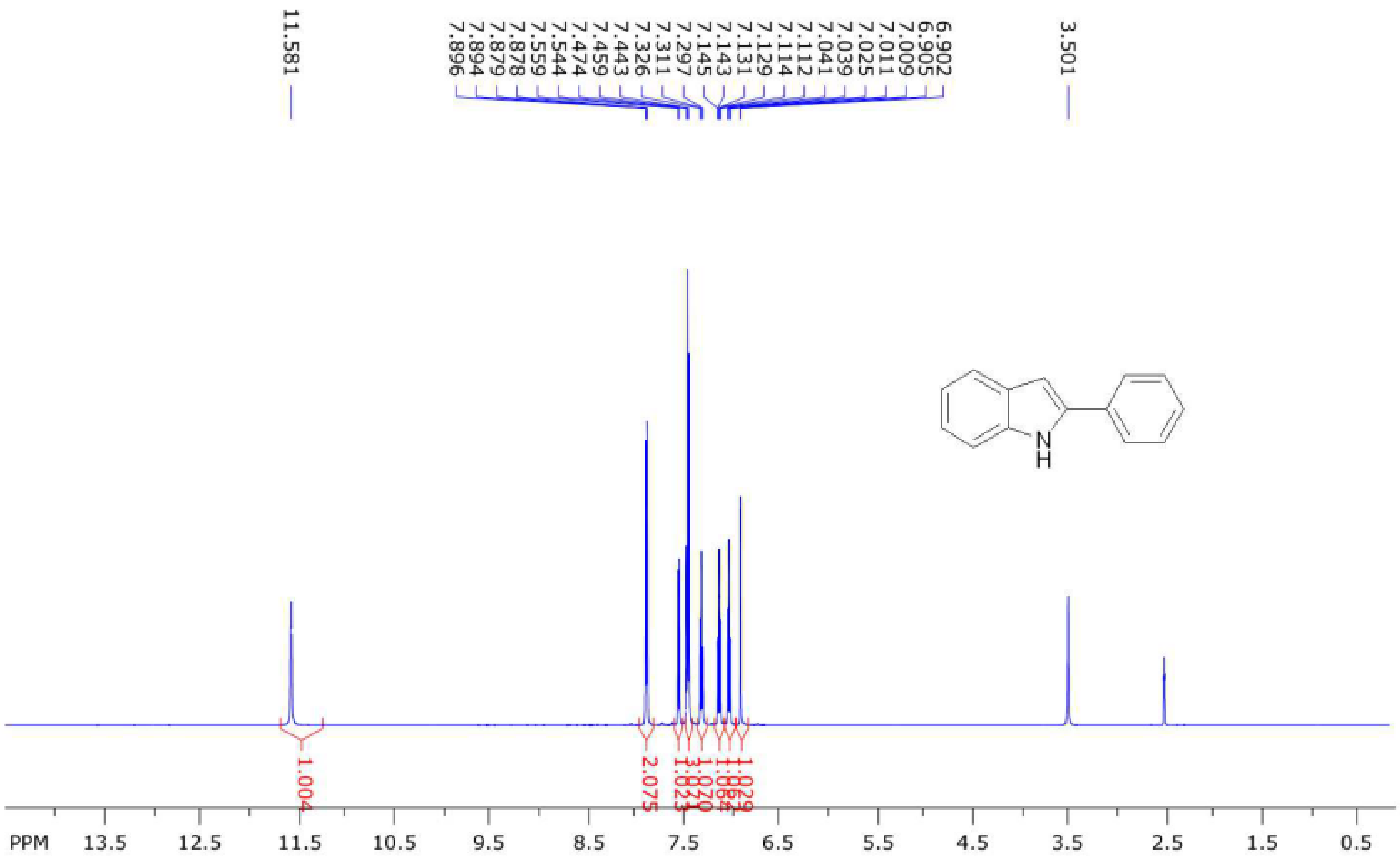

file: D:\NAPO\NMR\500-2\mkr11705\27\fid expt: <zg30>
transmitter freq.: 500.133001 MHz
time domain size: 65536 points
width: 12335.53 Hz = 24.6645 ppm = 0.188225 Hz/pt
number of scans: 24

freq. of 0 ppm: 500.130005 MHz
processed size: 65536 complex points
LB: 0.300 GF: 0.0000
Hz/cm: 288.193 ppm/cm: 0.57623

Compound 2v

SpinWorks 4: IVA 1516 13C DMSO

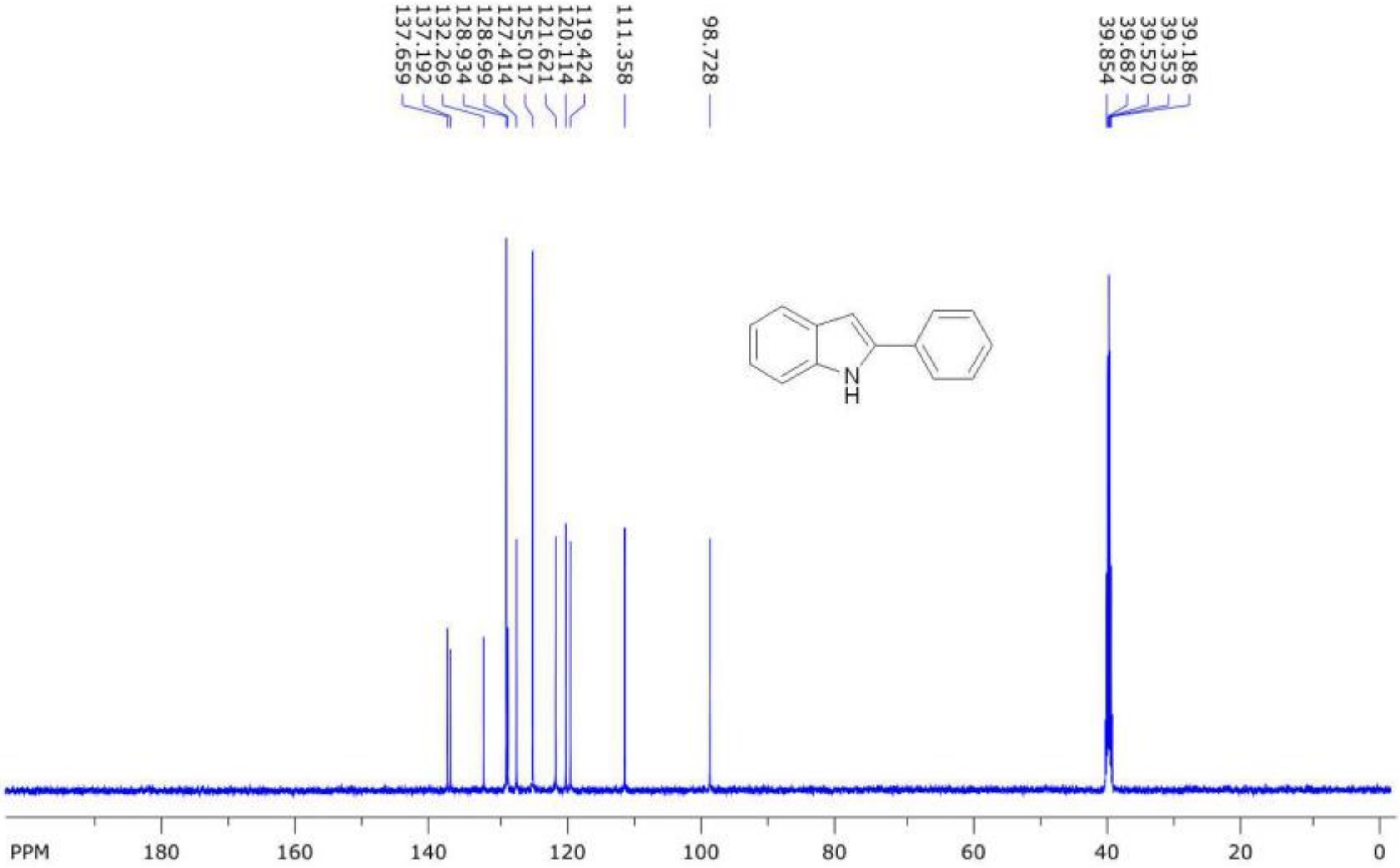

file: D:\NAPO\NMR\500-2\mkr11705\28\fid expt: <zgpg30>
transmitter freq.: 125.772879 MHz
time domain size: 65536 points
width: 36057.69 Hz = 286.6889 ppm = 0.550197 Hz/pt
number of scans: 224

freq. of 0 ppm: 125.757843 MHz
processed size: 32768 complex points
LB: 2.000 GF: 0.0000
Hz/cm: 1033.910 ppm/cm: 8.22045

Compound 2w

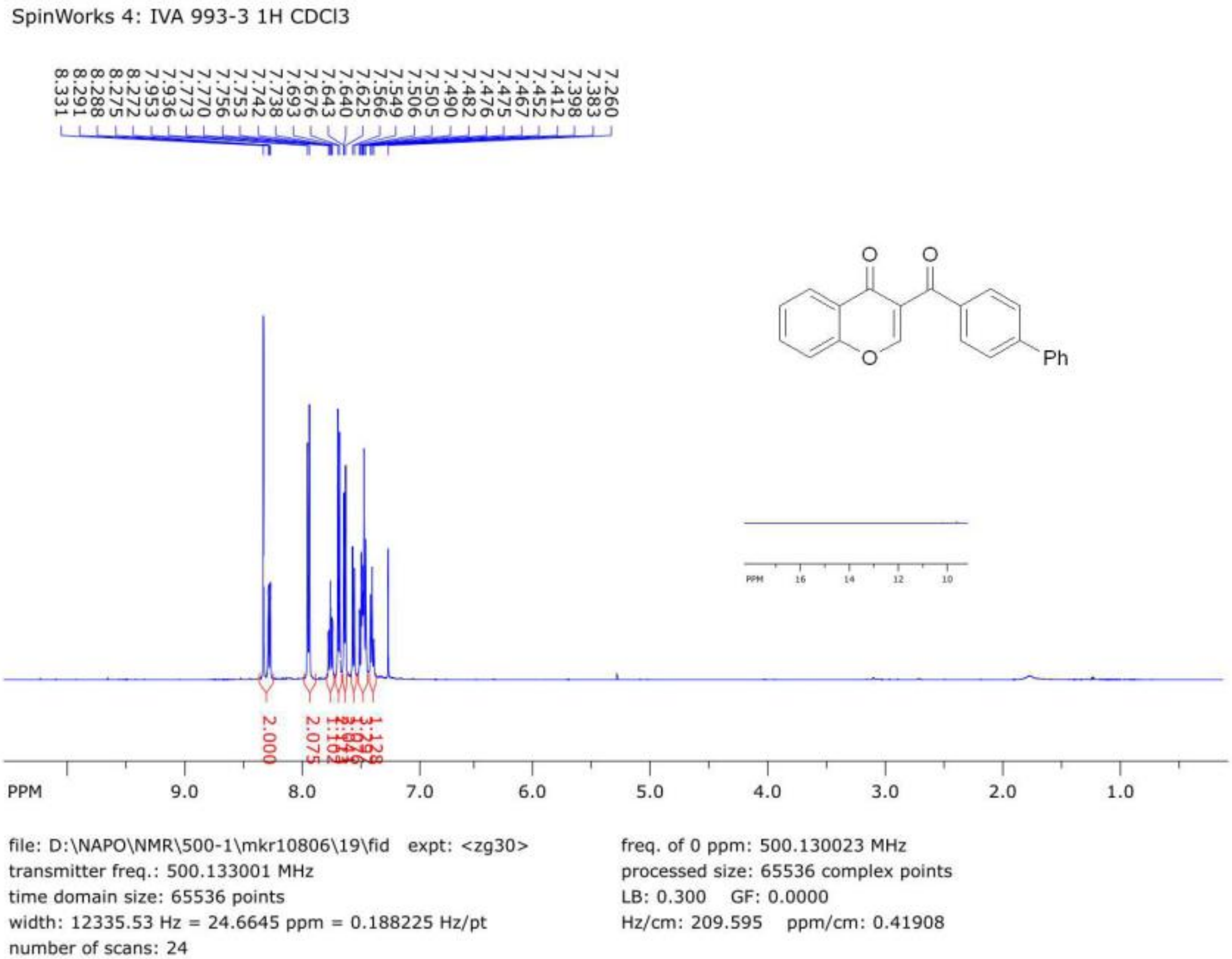
SpinWorks 4: IVA 993-3 1H CDCl3
8.331
8.291
8.288
8.275
8.272
7.953
7.936
7.773
7.770
7.756
7.753
7.742
7.738
7.693
7.676
7.643
7.640
7.625
7.566
7.549
7.506
7.505
7.490
7.482
7.476
7.475
7.467
7.452
7.412
7.398
7.383
7.260
Ph
PPM
9.0
8.0
7.0
6.0
5.0
4.0
3.0
2.0
1.0
2.000
2.075
file: D:\NAPO\NMR\500-1\mkr10806\19\fid expt: <zg30>
transmitter freq.: 500.133001 MHz
time domain size: 65536 points
width: 12335.53 Hz = 24.6645 ppm = 0.188225 Hz/pt
number of scans: 24
freq. of 0 ppm: 500.130023 MHz
processed size: 65536 complex points
LB: 0.300 GF: 0.0000
Hz/cm: 209.595 ppm/cm: 0.41908

Compound 2w

SpinWorks 4: IVA 993-3 13C CDCl3

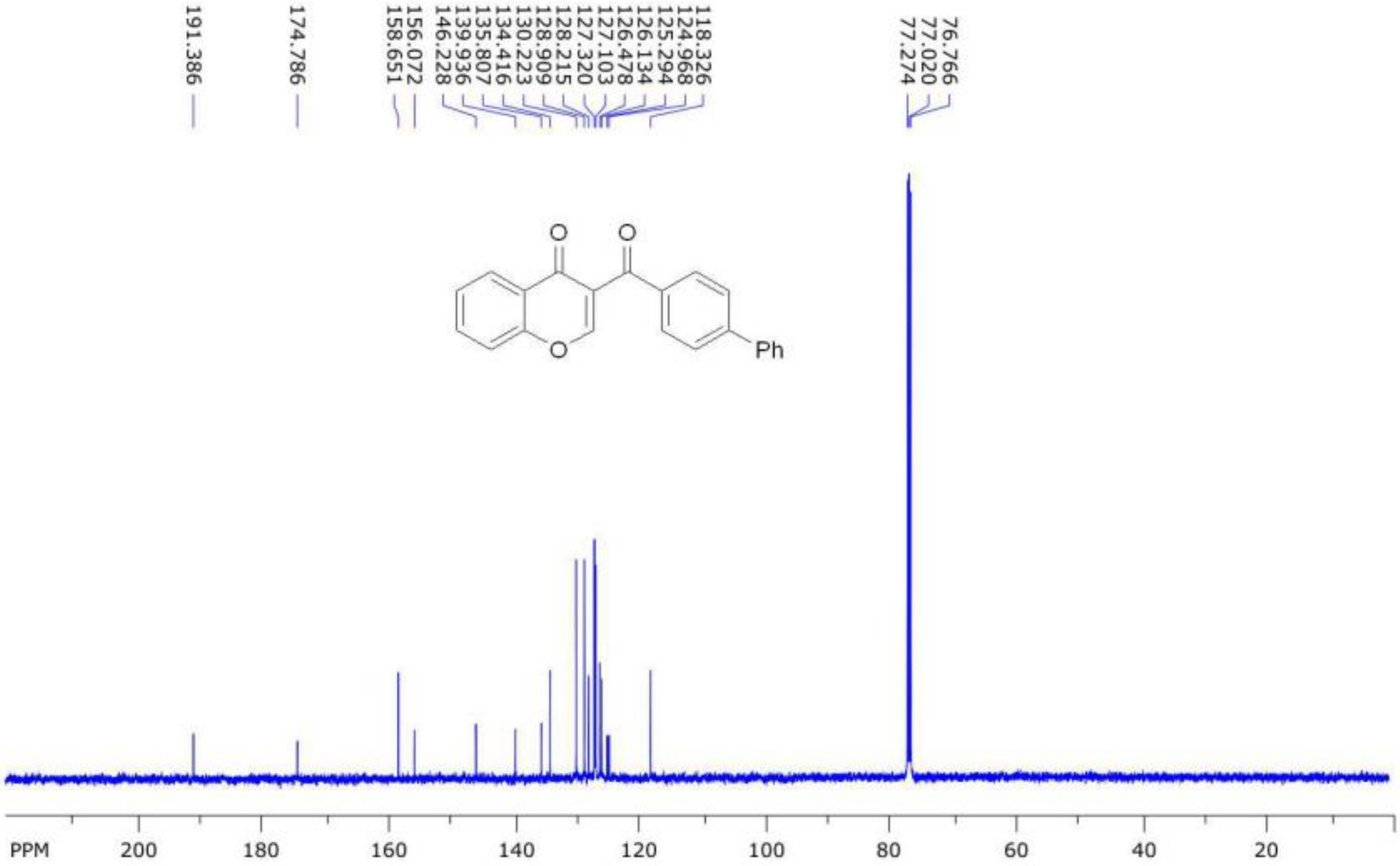

file: D:\NAPO\NMR\500-1\mkr10806\20\fid expt: <zgpg30>
transmitter freq.: 125.772879 MHz
time domain size: 65536 points
width: 36057.69 Hz = 286.6889 ppm = 0.550197 Hz/pt
number of scans: 512

freq. of 0 ppm: 125.757795 MHz
processed size: 32768 complex points
LB: 2.000 GF: 0.0000
Hz/cm: 1112.045 ppm/cm: 8.84169

Compound 2x

SpinWorks 4: IVA 1315 1H CDCl3

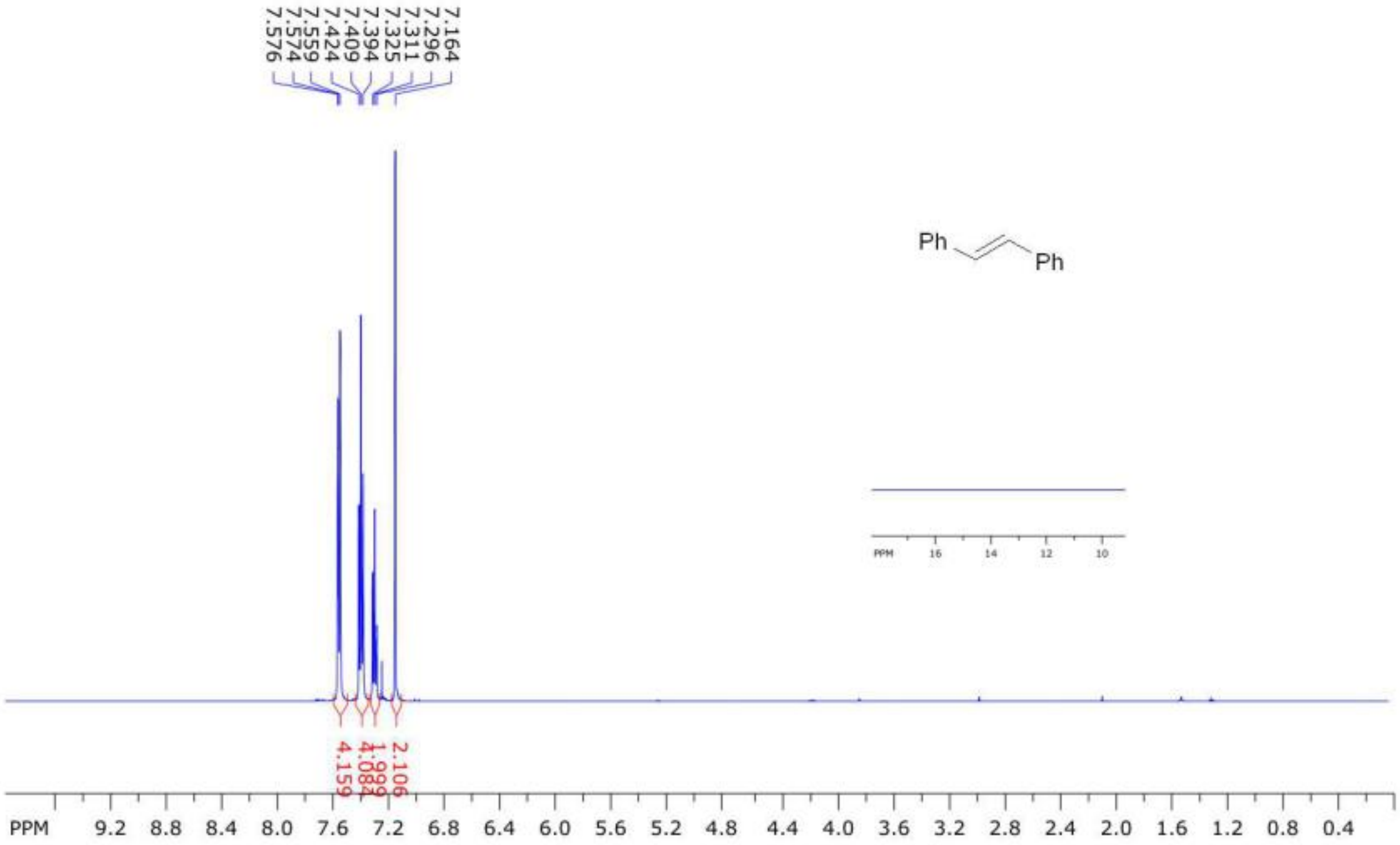

file: D:\NAPO\NMR\500-1\mkr11611\11\fid expt: <zg30>
transmitter freq.: 500.133001 MHz
time domain size: 65536 points
width: 12335.53 Hz = 24.6645 ppm = 0.188225 Hz/pt
number of scans: 24

freq. of 0 ppm: 500.130023 MHz
processed size: 65536 complex points
LB: 0.300 GF: 0.0000
Hz/cm: 198.678 ppm/cm: 0.39725

Compound 2x

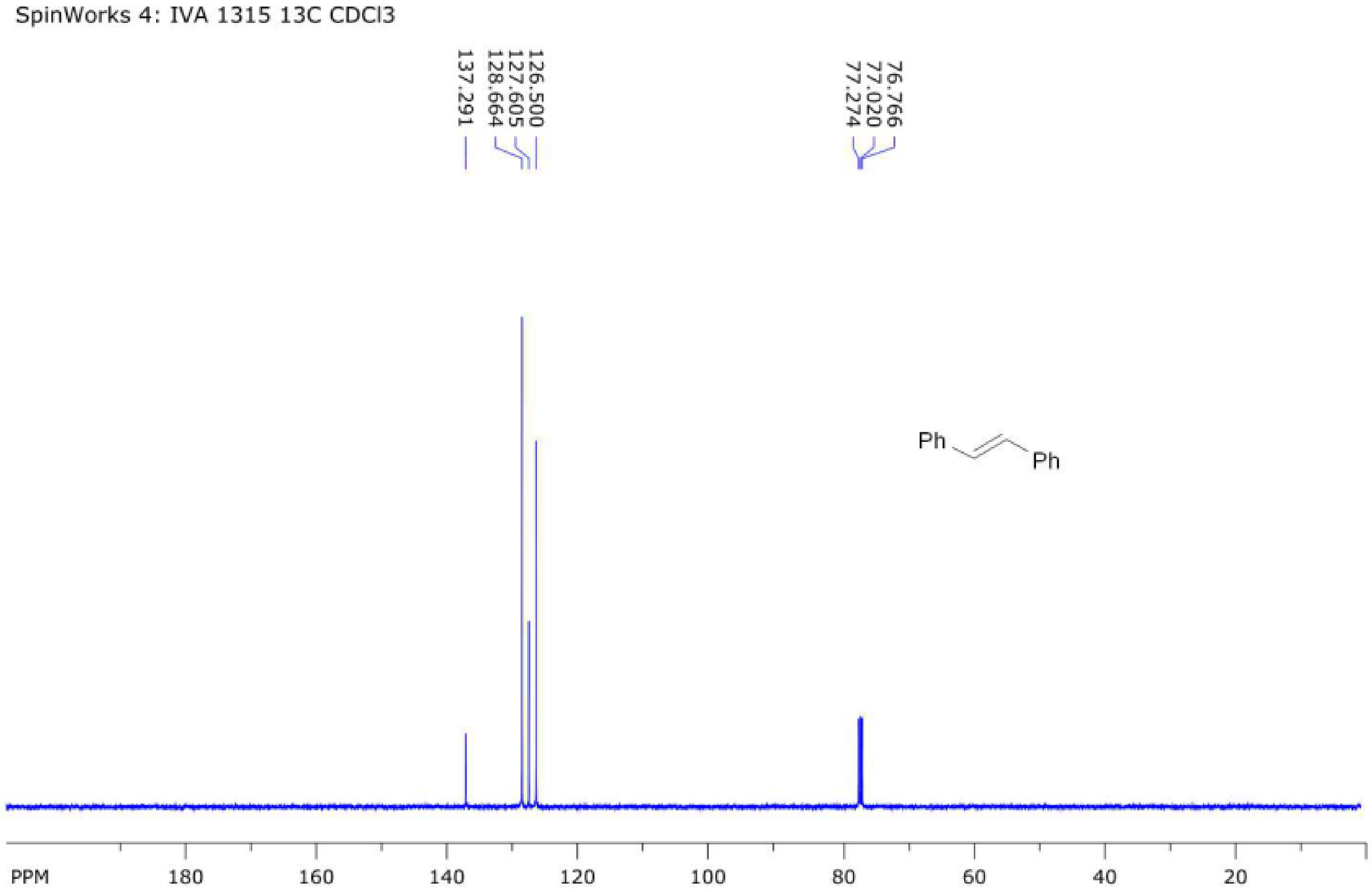

file: D:\NAPO\NMR\500-1\mkr11611\12\fid expt: <zgpg30>
transmitter freq.: 125.772879 MHz
time domain size: 65536 points
width: 36057.69 Hz = 286.6889 ppm = 0.550197 Hz/pt
number of scans: 128

freq. of 0 ppm: 125.757801 MHz
processed size: 32768 complex points
LB: 2.000 GF: 0.0000
Hz/cm: 1040.248 ppm/cm: 8.27085

Compound 2y

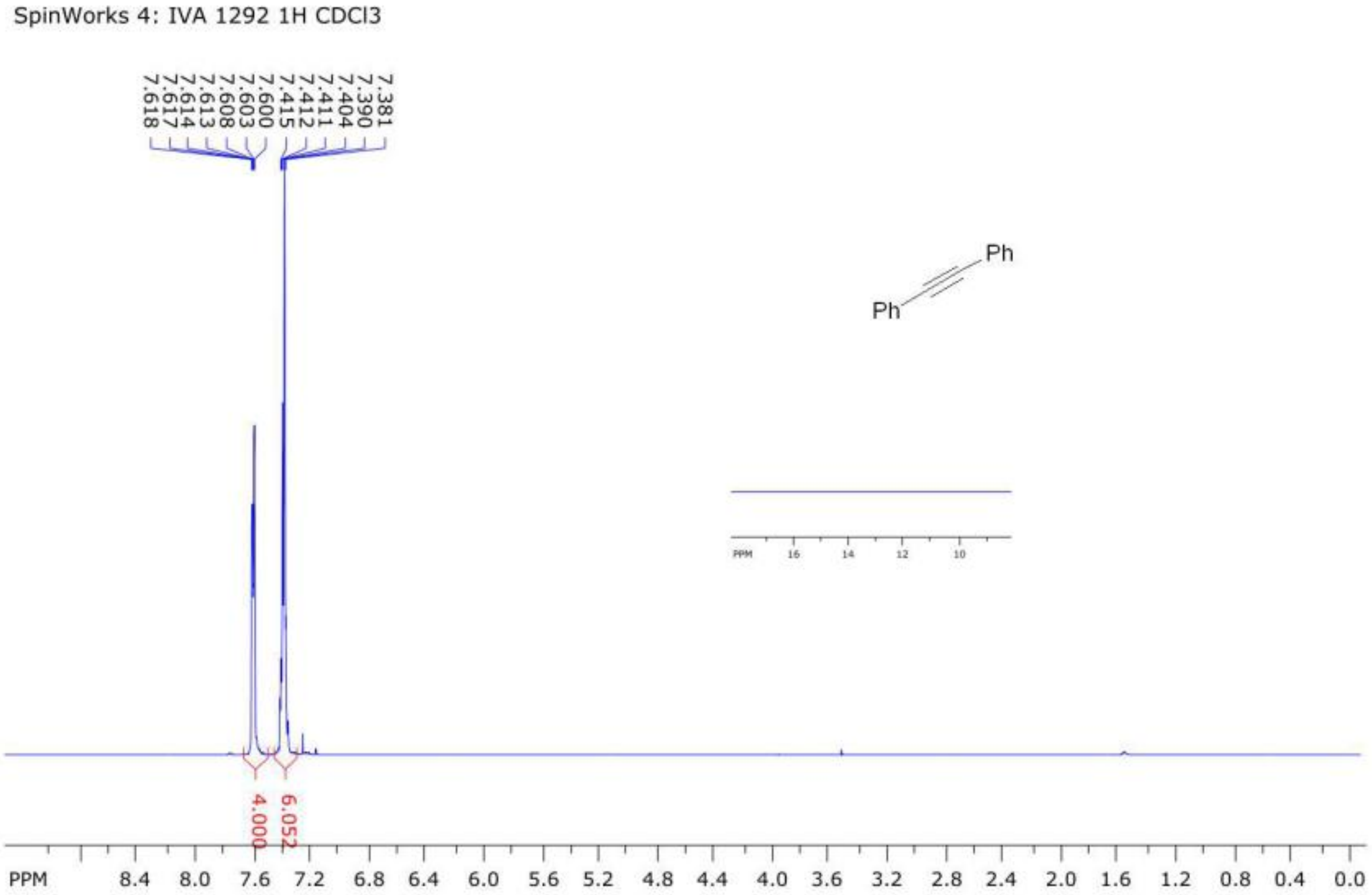

file: D:\NAPO\NMR\500-1\mkr10911\45\fid expt: <zg30>
transmitter freq.: 500.133001 MHz
time domain size: 65536 points
width: 12335.53 Hz = 24.6645 ppm = 0.188225 Hz/pt
number of scans: 24

freq. of 0 ppm: 500.130022 MHz
processed size: 65536 complex points
LB: 0.300 GF: 0.0000
Hz/cm: 188.854 ppm/cm: 0.37761

Compound 2y

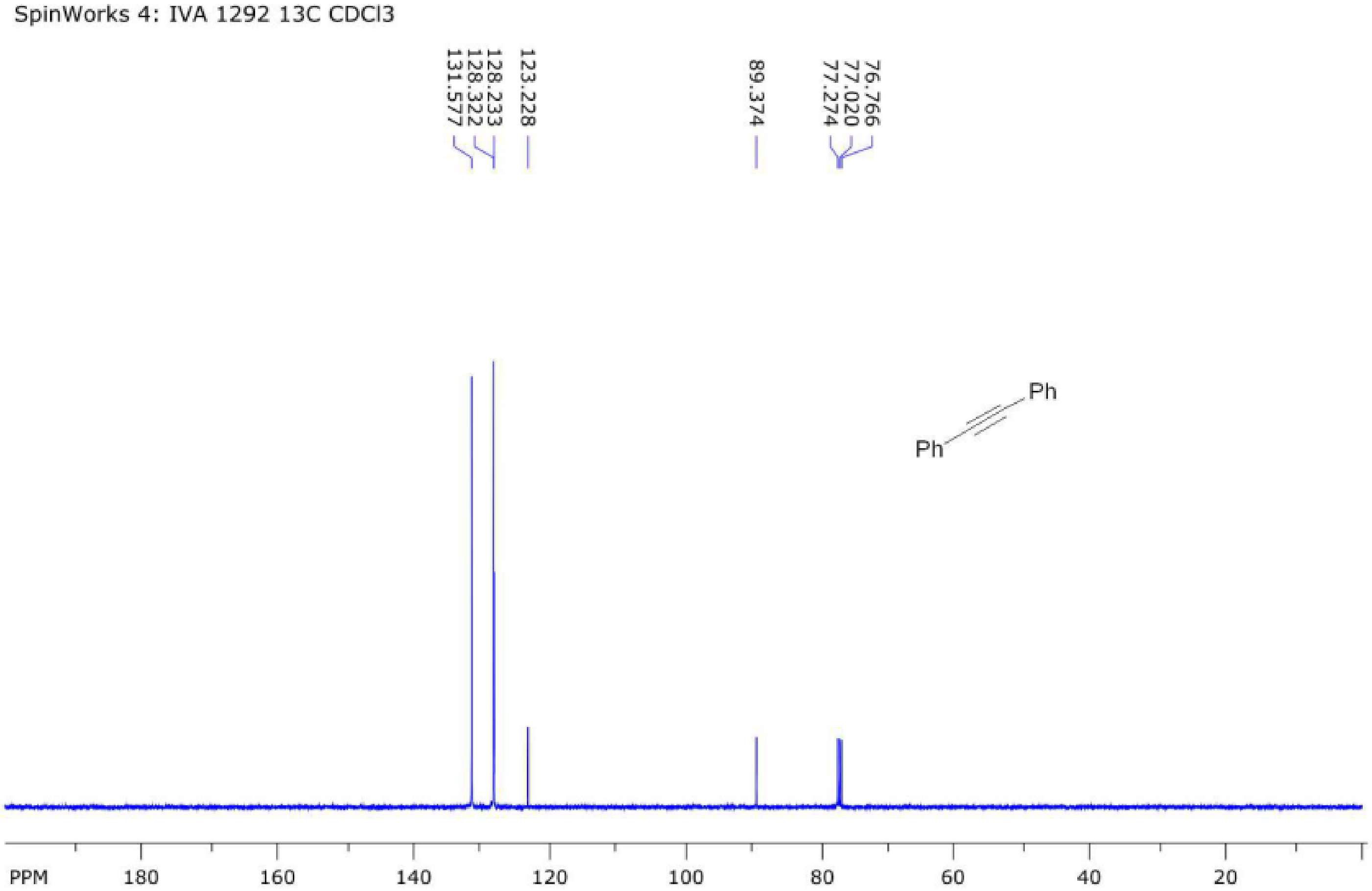

file: D:\NAPO\NMR\500-1\mkr10911\46\fid expt: <zgpg30>
transmitter freq.: 125.772879 MHz
time domain size: 65536 points
width: 36057.69 Hz = 286.6889 ppm = 0.550197 Hz/pt
number of scans: 64

freq. of 0 ppm: 125.757805 MHz
processed size: 32768 complex points
LB: 2.000 GF: 0.0000
Hz/cm: 1011.530 ppm/cm: 8.04251

Compound 2z

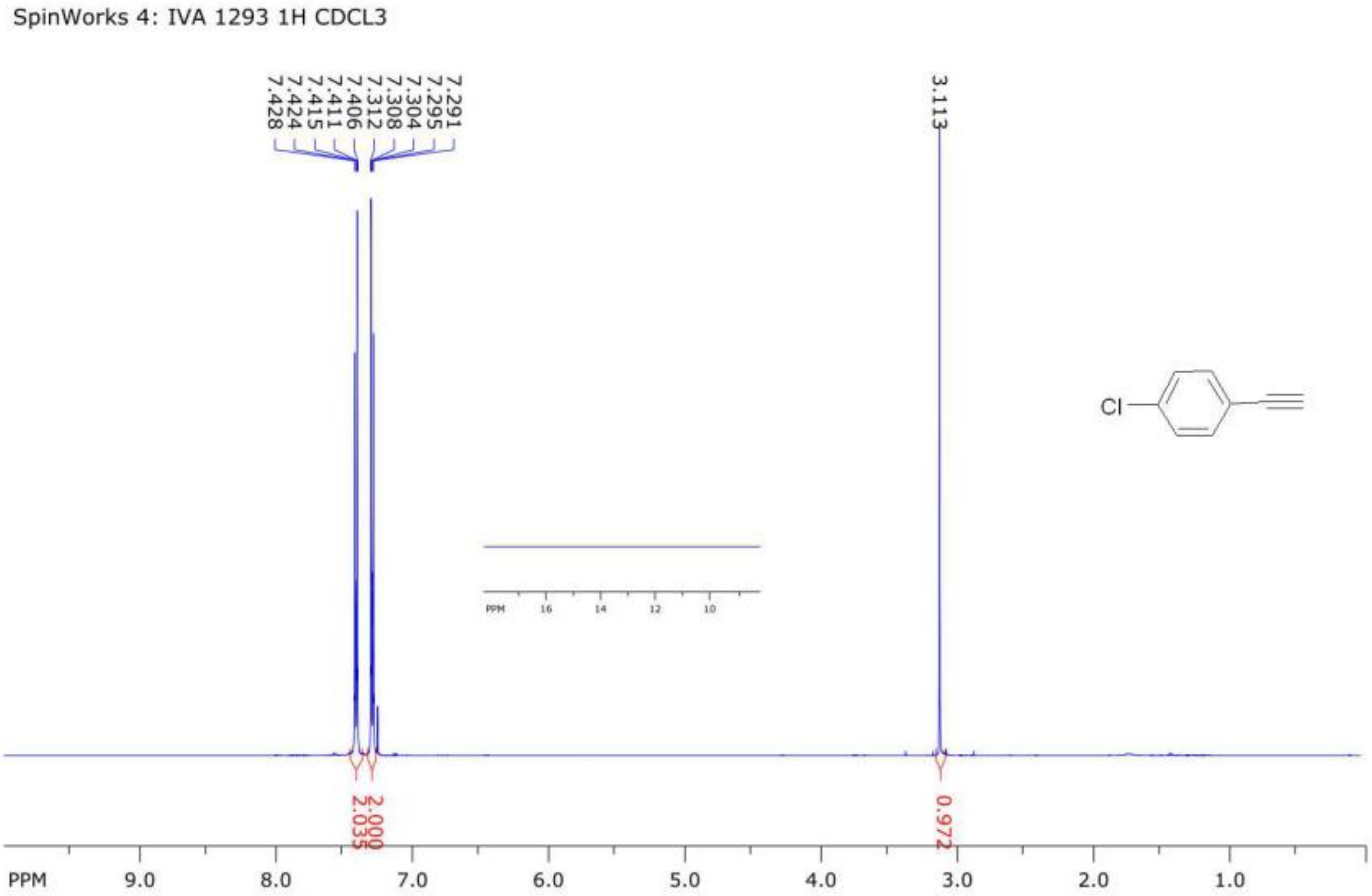

file: D:\NAPO\NMR\500-1\mkr10911\27\fid expt: <zg30>
transmitter freq.: 500.133001 MHz
time domain size: 65536 points
width: 12335.53 Hz = 24.6645 ppm = 0.188225 Hz/pt
number of scans: 24

freq. of 0 ppm: 500.130023 MHz
processed size: 65536 complex points
LB: 0.300 GF: 0.0000
Hz/cm: 200.316 ppm/cm: 0.40053

Compound 2z

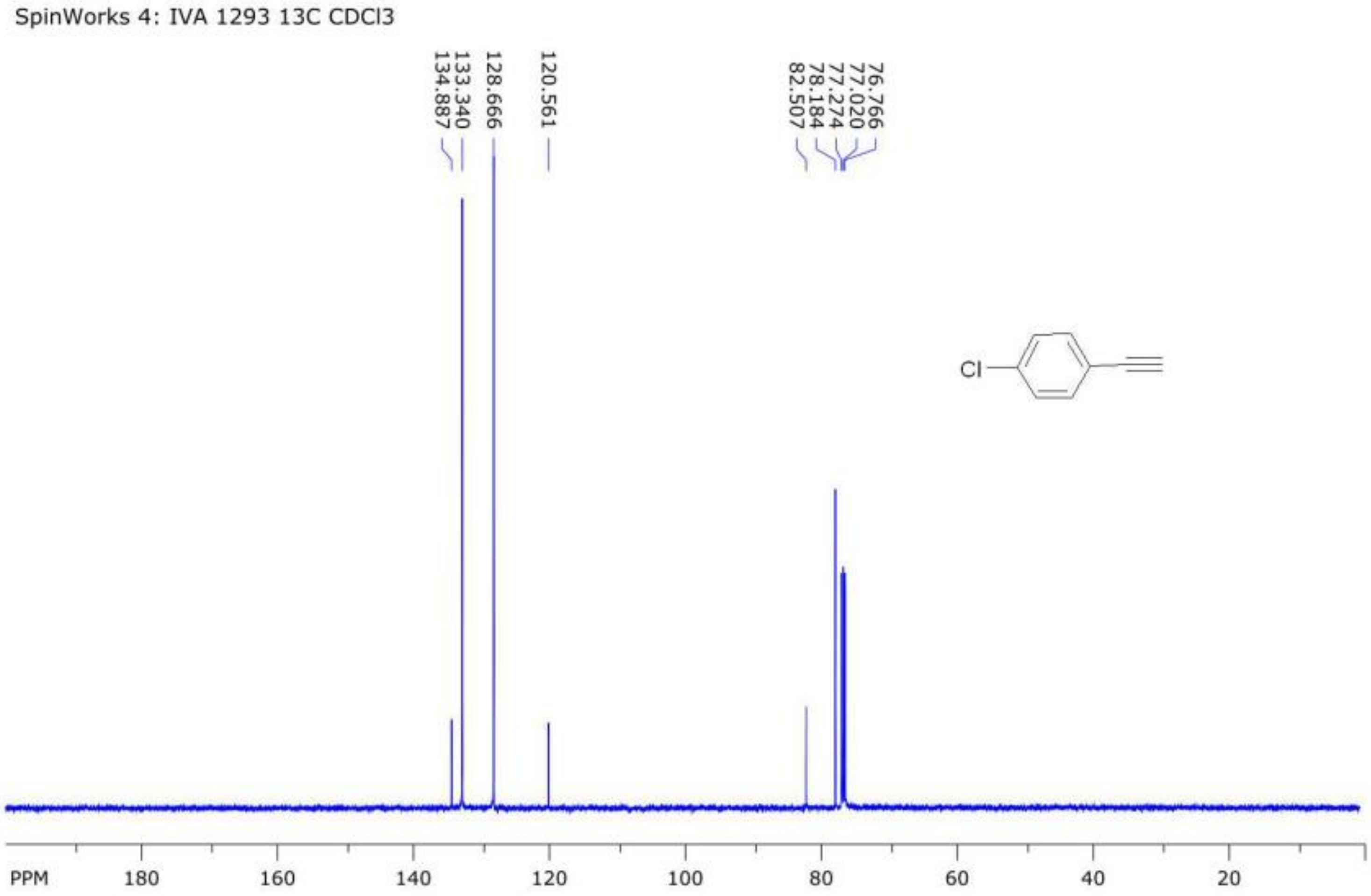

file: D:\NAPO\NMR\500-1\mkr10911\28\fid expt: <zgpg30>
transmitter freq.: 125.772879 MHz
time domain size: 65536 points
width: 36057.69 Hz = 286.6889 ppm = 0.550197 Hz/pt
number of scans: 512

freq. of 0 ppm: 125.757794 MHz
processed size: 32768 complex points
LB: 2.000 GF: 0.0000
Hz/cm: 1006.744 ppm/cm: 8.00446

Compound 6a

SpinWorks 4: SVS 230 1h CDCL3

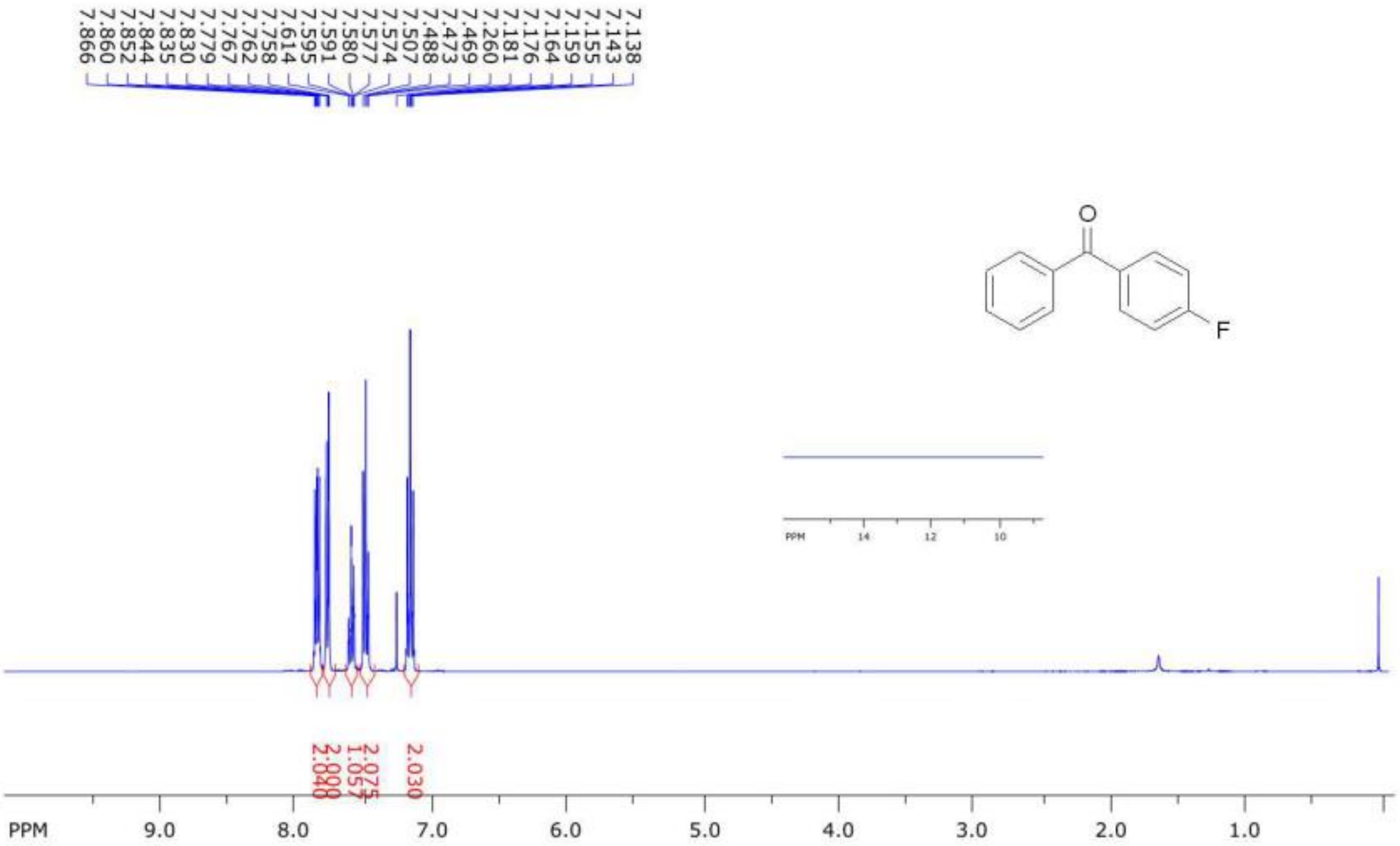

file: ...NAPO\NMR\JELA\nmr\jn-SM-S230\1\fid expt: <zg30>
transmitter freq.: 400.132471 MHz
time domain size: 65536 points
width: 8196.72 Hz = 20.4850 ppm = 0.125072 Hz/pt
number of scans: 16

freq. of 0 ppm: 400.130009 MHz
processed size: 65536 complex points
LB: 0.300 GF: 0.0000
Hz/cm: 163.934 ppm/cm: 0.40970

Compound 6a

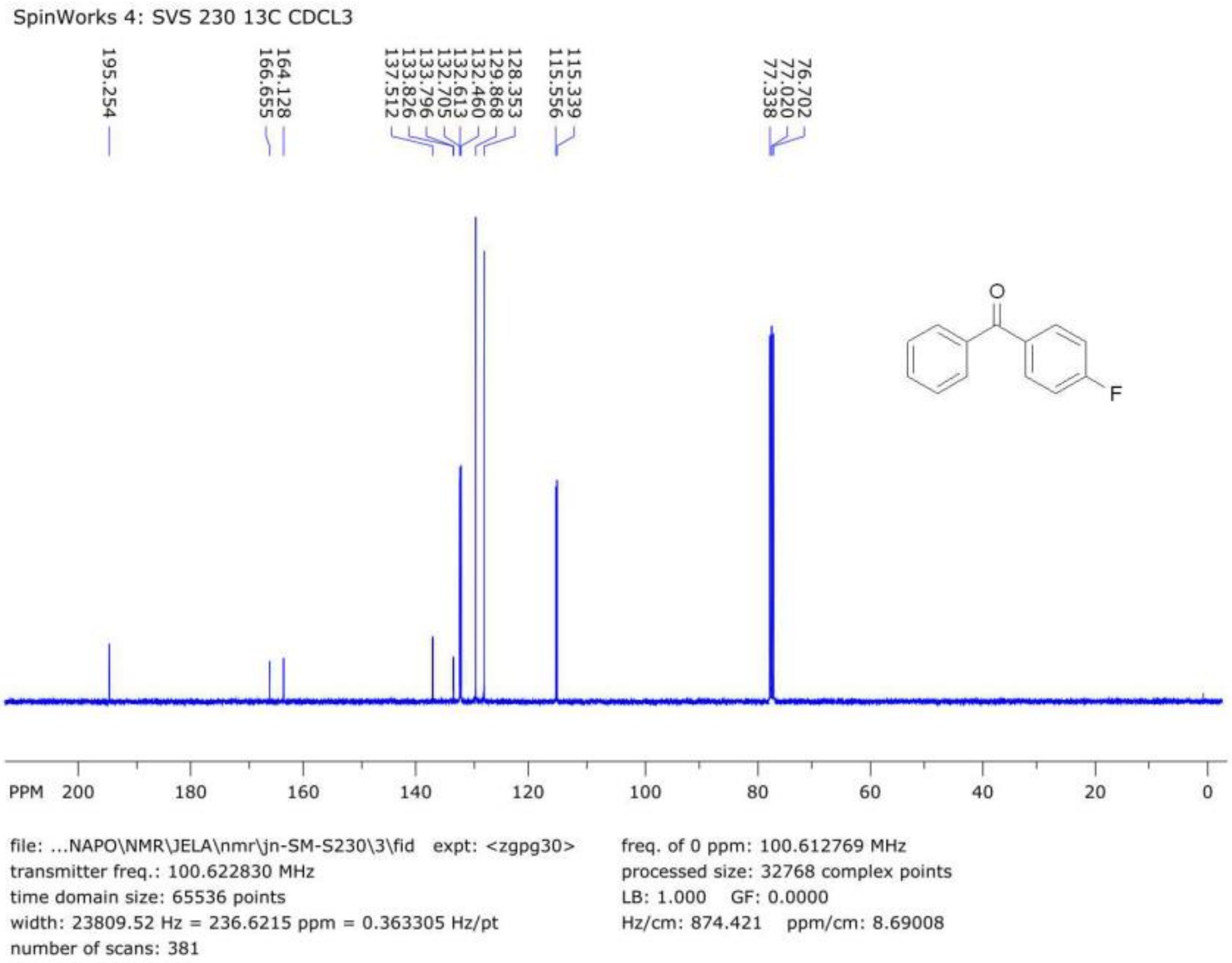
SpinWorks 4: SVS 230 13C CDCL3
195.254
164.128
166.655
128.353
129.868
132.460
132.613
132.705
133.796
133.826
137.512
115.339
115.556
76.702
77.020
77.338
PPM 200 180 160 140 120 100 80 60 40 20 0
F
O
file: ...NAPO\NMR\JELA\nmr\jn-SM-S230\3\fid expt: <zgpg30>
transmitter freq.: 100.622830 MHz
time domain size: 65536 points
width: 23809.52 Hz = 236.6215 ppm = 0.363305 Hz/pt
number of scans: 381
freq. of 0 ppm: 100.612769 MHz
processed size: 32768 complex points
LB: 1.000 GF: 0.0000
Hz/cm: 874.421 ppm/cm: 8.69008

Compound 6b

SpinWorks 4: IVA 1596 1H DMSO

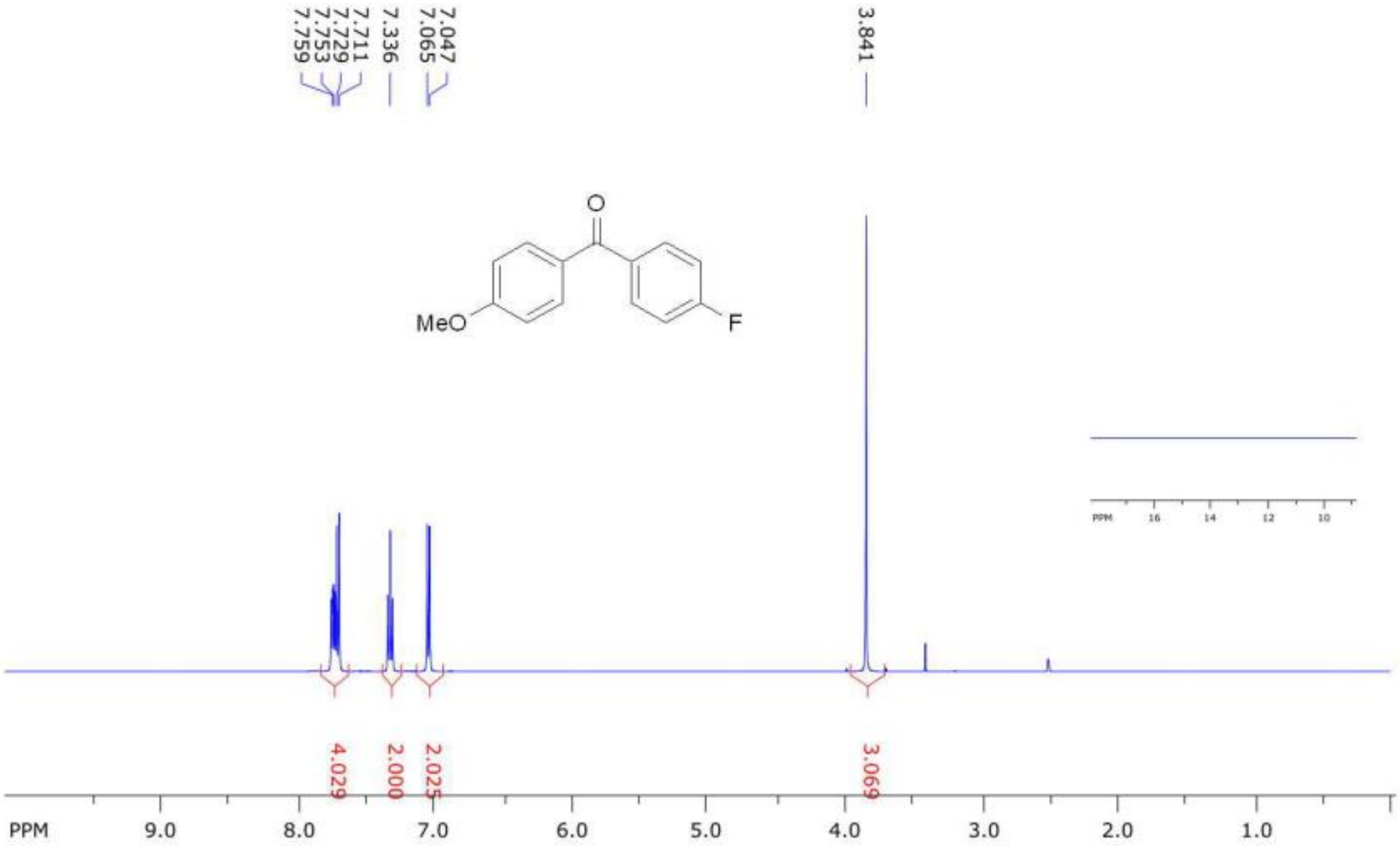

file: ...APO\NMR\500-2\mkr13107\23 1596\fid expt: <zg30>
transmitter freq.: 500.133001 MHz
time domain size: 65536 points
width: 12335.53 Hz = 24.6645 ppm = 0.188225 Hz/pt
number of scans: 24

freq. of 0 ppm: 500.130003 MHz
processed size: 65536 complex points
LB: 0.300 GF: 0.0000
Hz/cm: 203.591 ppm/cm: 0.40707

Compound 6b

SpinWorks 4: IVA 1596 13C DMSO

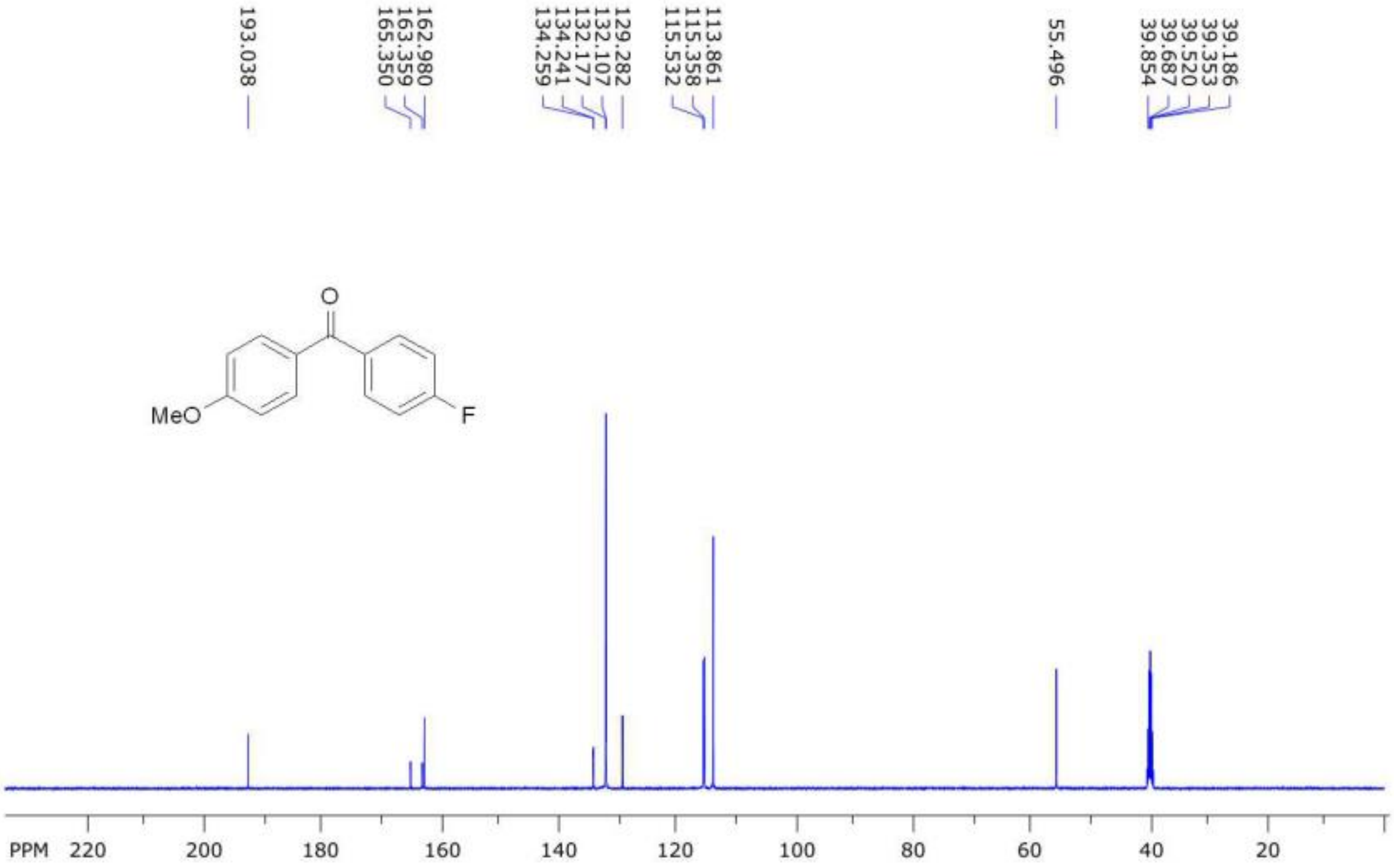

file: D:\NAPO\NMR\500-2\mkr13107\24\fid expt: <zgpg30>
transmitter freq.: 125.772879 MHz
time domain size: 65536 points
width: 36057.69 Hz = 286.6889 ppm = 0.550197 Hz/pt
number of scans: 512

freq. of 0 ppm: 125.757842 MHz
processed size: 32768 complex points
LB: 2.000 GF: 0.0000
Hz/cm: 1180.650 ppm/cm: 9.38716

Compound 6c

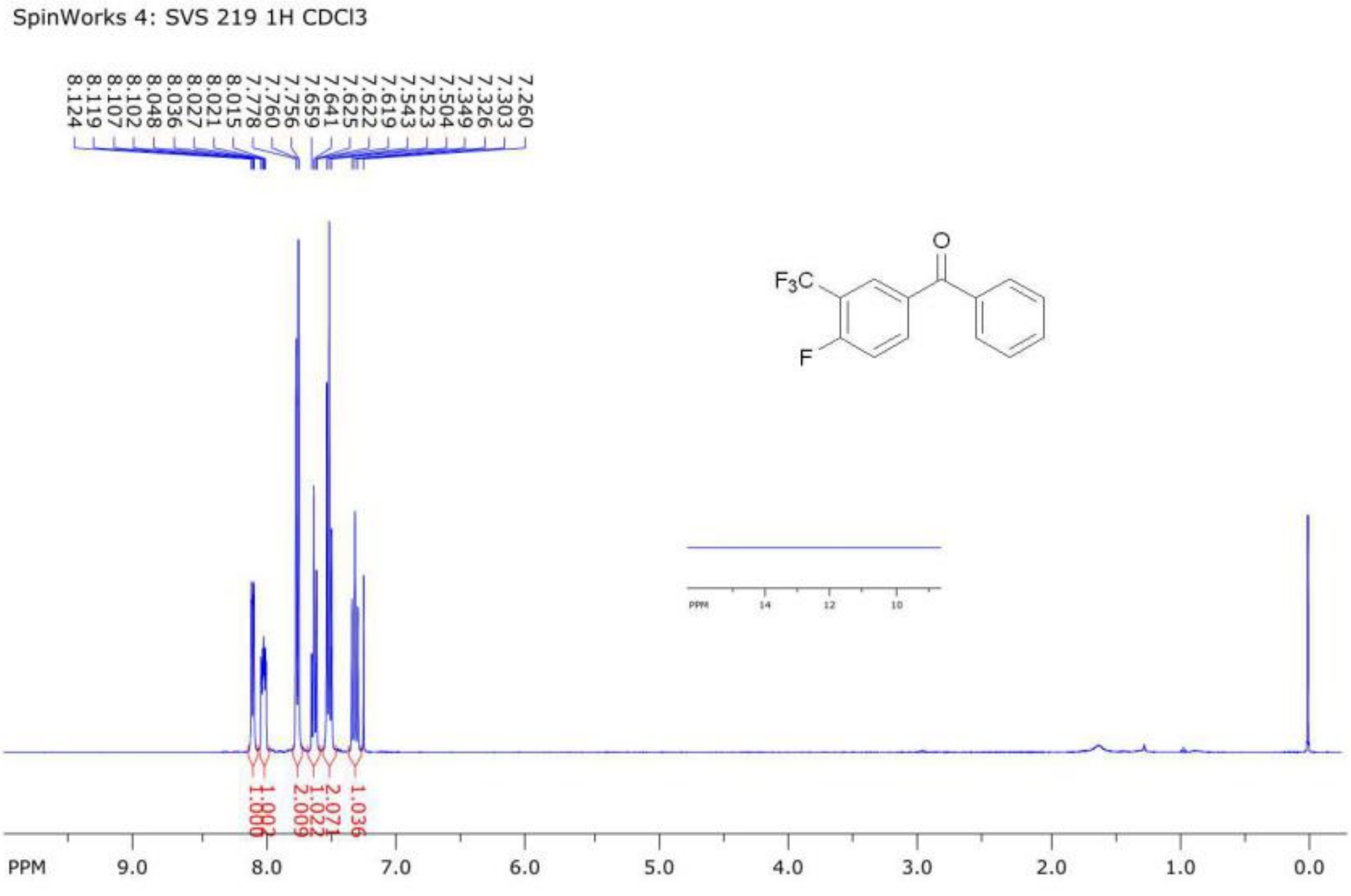

file: D:\NAPO\NMR\JELA\nmr\jn-219\1\fid expt: <zg30>
transmitter freq.: 400.132471 MHz
time domain size: 65536 points
width: 8196.72 Hz = 20.4850 ppm = 0.125072 Hz/pt
number of scans: 16

freq. of 0 ppm: 400.130009 MHz
processed size: 65536 complex points
LB: 0.300 GF: 0.0000
Hz/cm: 164.660 ppm/cm: 0.41151

Compound 6c

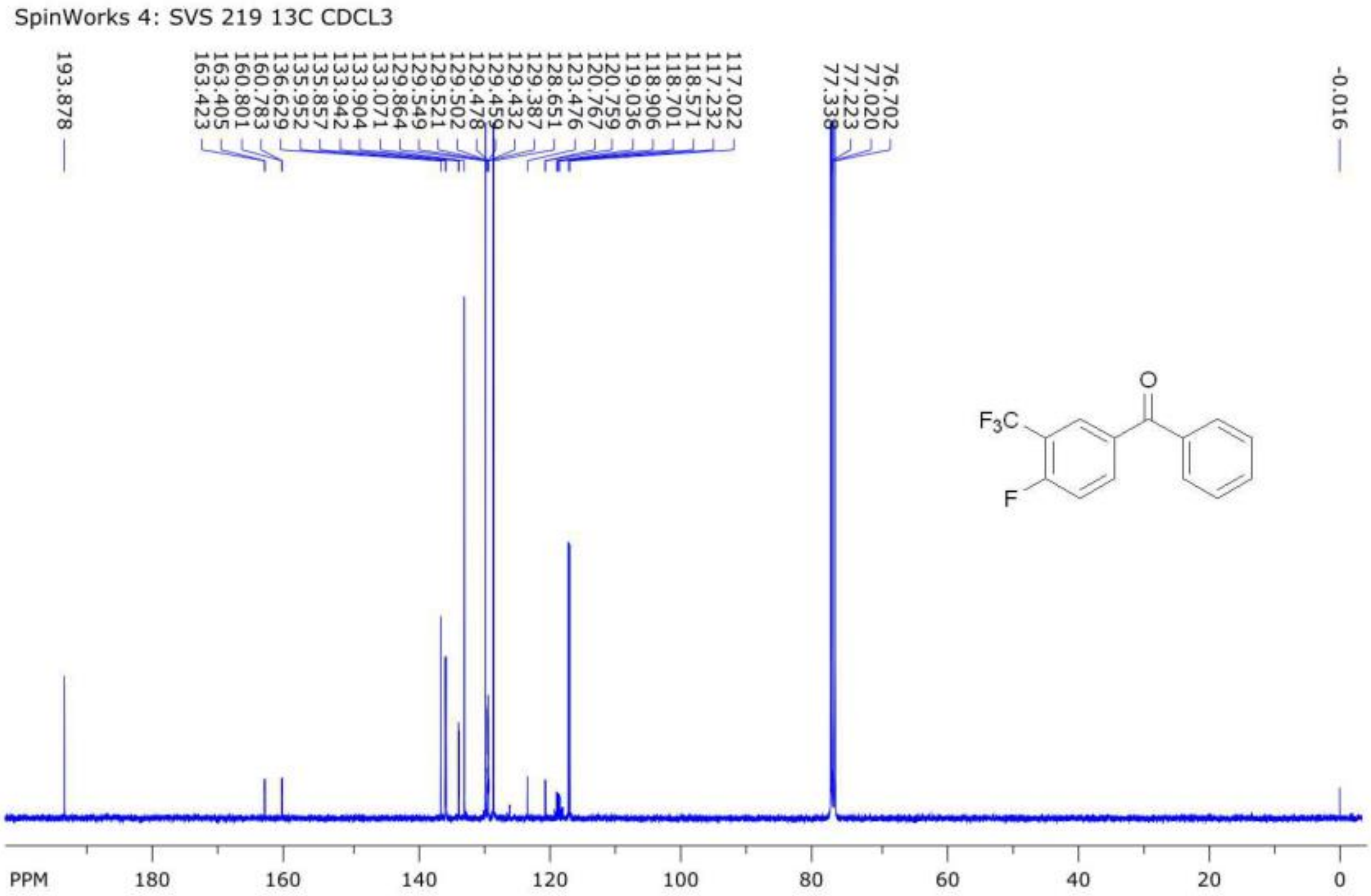

file: D:\NAPO\NMR\JELA\nmr\jn-219\2\fid expt: <zgpg30>
transmitter freq.: 100.622830 MHz
time domain size: 65536 points
width: 23809.52 Hz = 236.6215 ppm = 0.363305 Hz/pt
number of scans: 3500

freq. of 0 ppm: 100.612767 MHz
processed size: 32768 complex points
LB: 1.000 GF: 0.0000
Hz/cm: 830.173 ppm/cm: 8.25034

Compound 6d

SpinWorks 4:

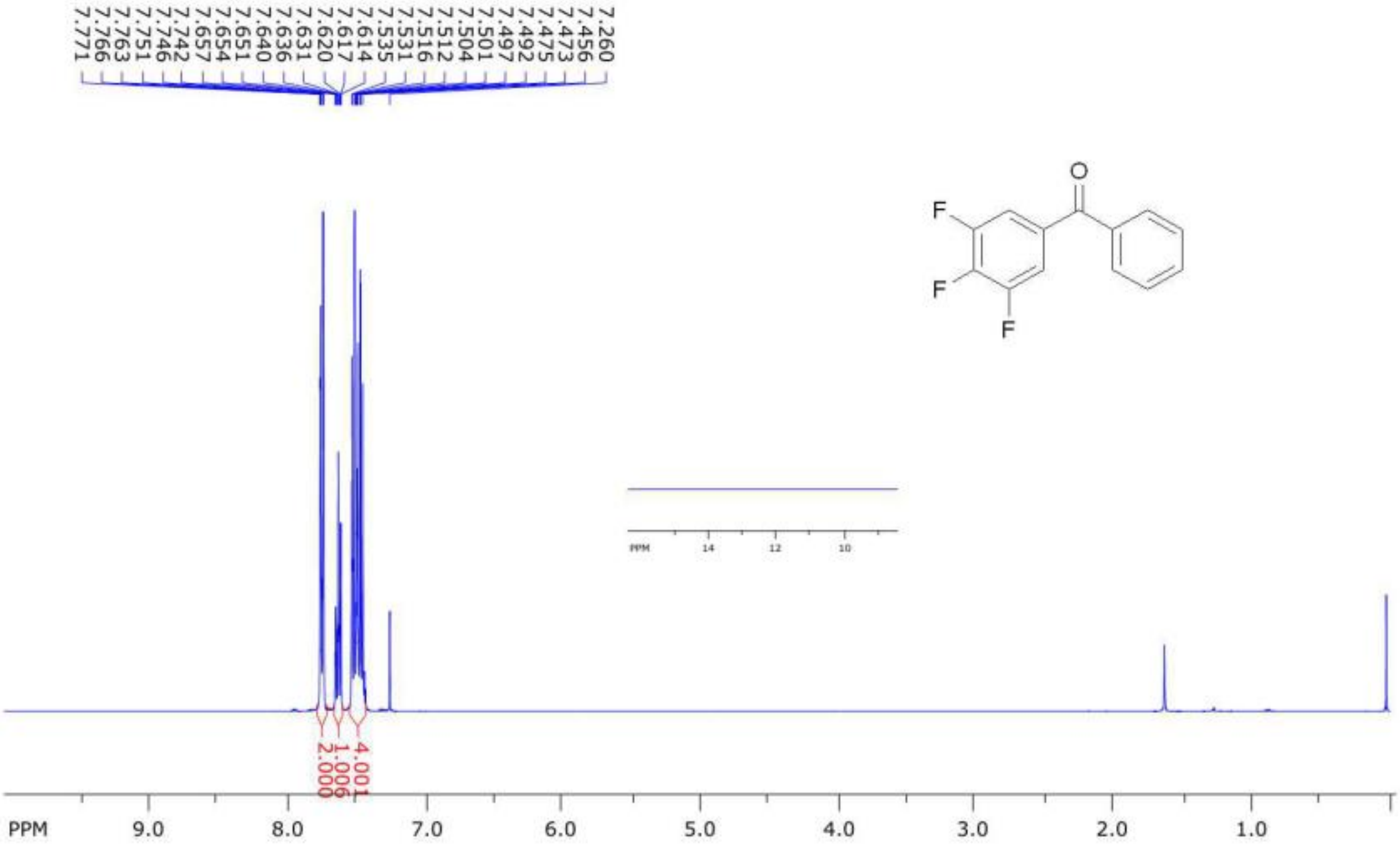

file: ...:\NAPO\NMR\JELA\nmr\jn-S-216\1\fid expt: <zg30>
transmitter freq.: 400.132471 MHz
time domain size: 65536 points
width: 8196.72 Hz = 20.4850 ppm = 0.125072 Hz/pt
number of scans: 16

freq. of 0 ppm: 400.130009 MHz
processed size: 65536 complex points
LB: 0.300 GF: 0.0000
Hz/cm: 161.758 ppm/cm: 0.40426

Compound 6d

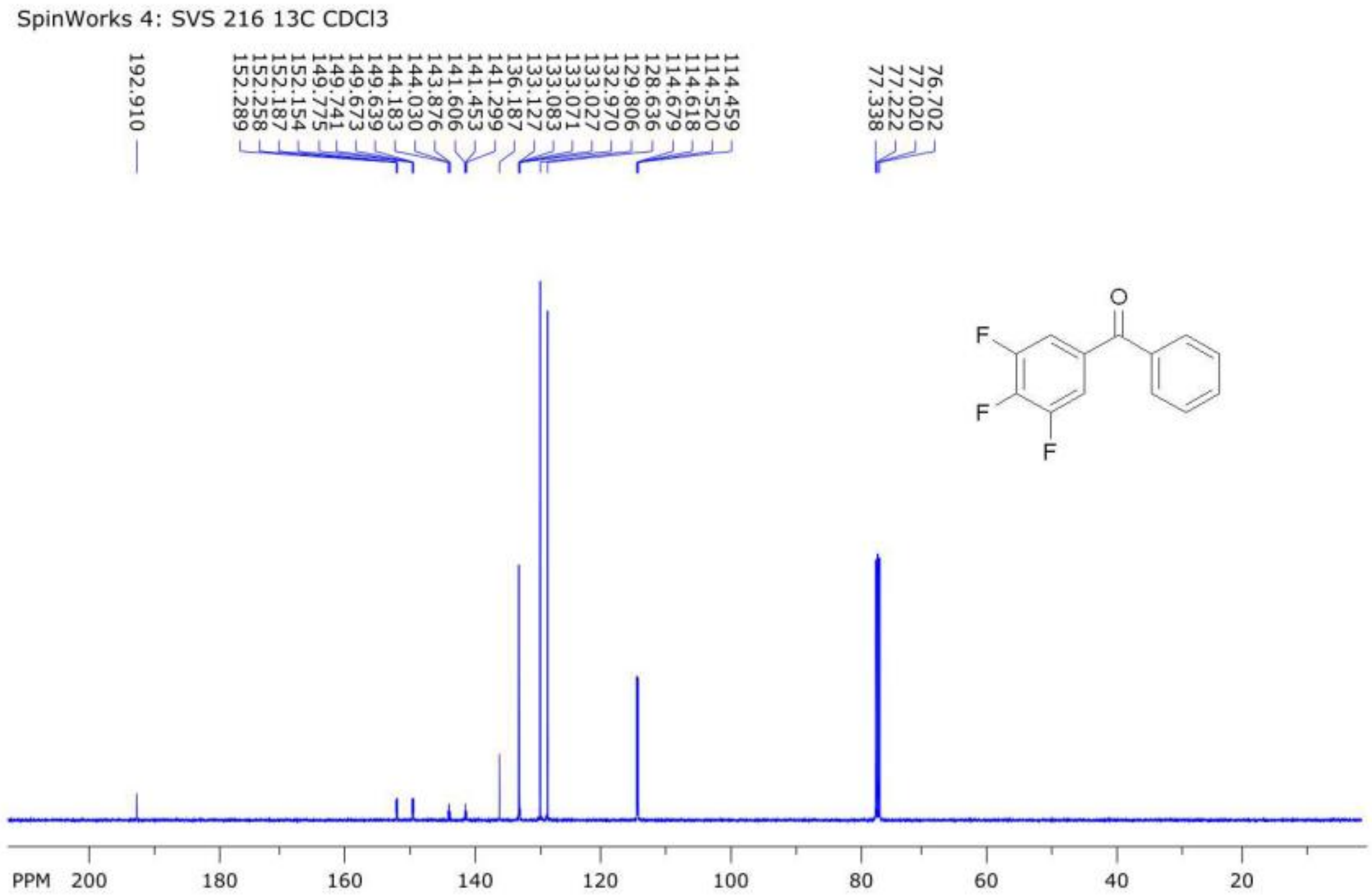

file: ...:\NAPO\NMR\JELA\nmr\jn-S-216\2\fid expt: <zgpg30>
transmitter freq.: 100.622830 MHz
time domain size: 65536 points
width: 23809.52 Hz = 236.6215 ppm = 0.363305 Hz/pt
number of scans: 2000

freq. of 0 ppm: 100.612769 MHz
processed size: 32768 complex points
LB: 1.000 GF: 0.0000
Hz/cm: 852.297 ppm/cm: 8.47021

Compound 6e

SpinWorks 4: IVS 1614 1H CDCl3

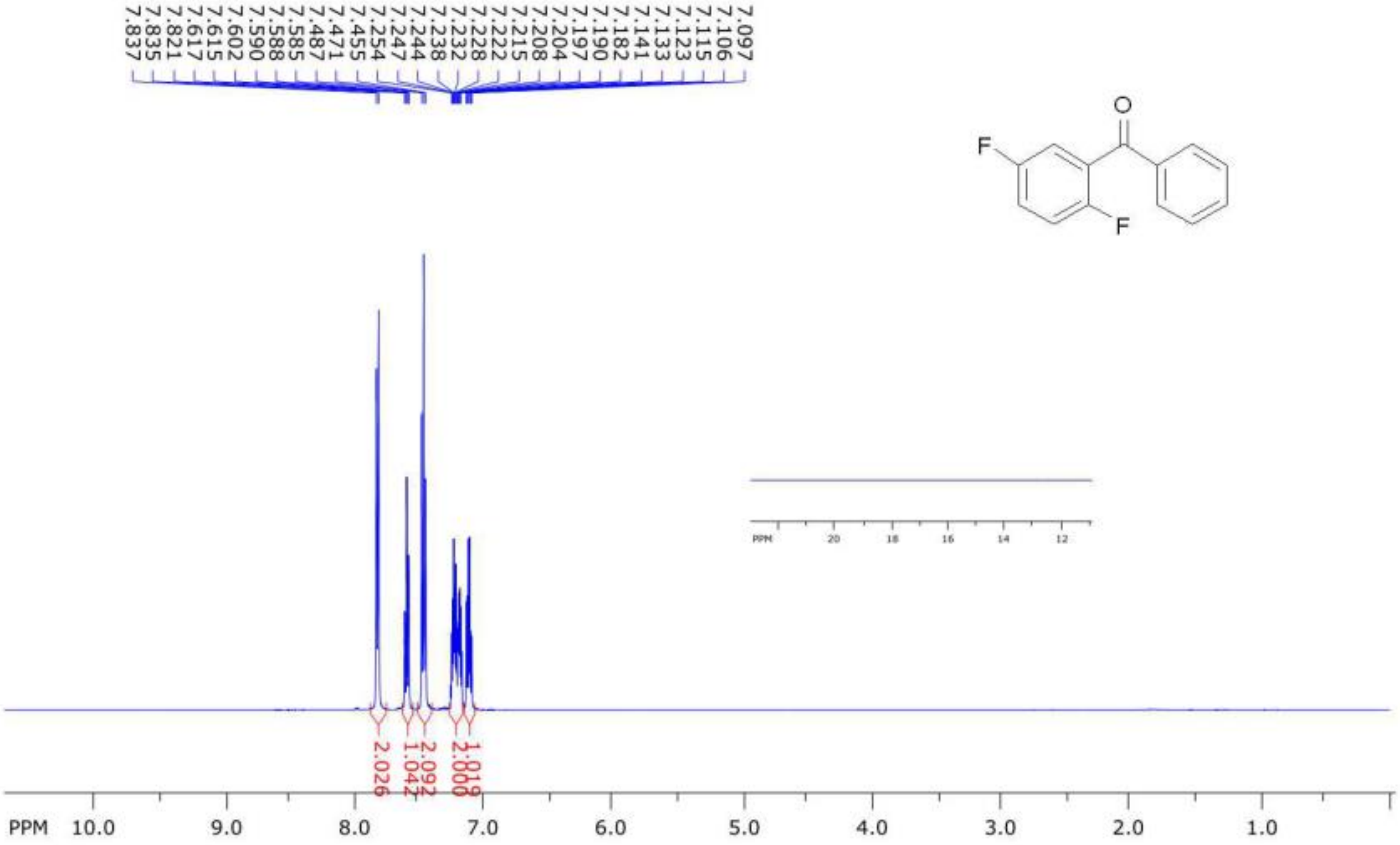

file: D:\NAPO\NMR\500-2\mkr10402\25\fid expt: <zg30>
transmitter freq.: 500.133001 MHz
time domain size: 65536 points
width: 12335.53 Hz = 24.6645 ppm = 0.188225 Hz/pt
number of scans: 24

freq. of 0 ppm: 500.127637 MHz
processed size: 65536 complex points
LB: 0.300 GF: 0.0000
Hz/cm: 215.053 ppm/cm: 0.42999

Compound 6e

SpinWorks 4: IVA 1614 13C CDCL3

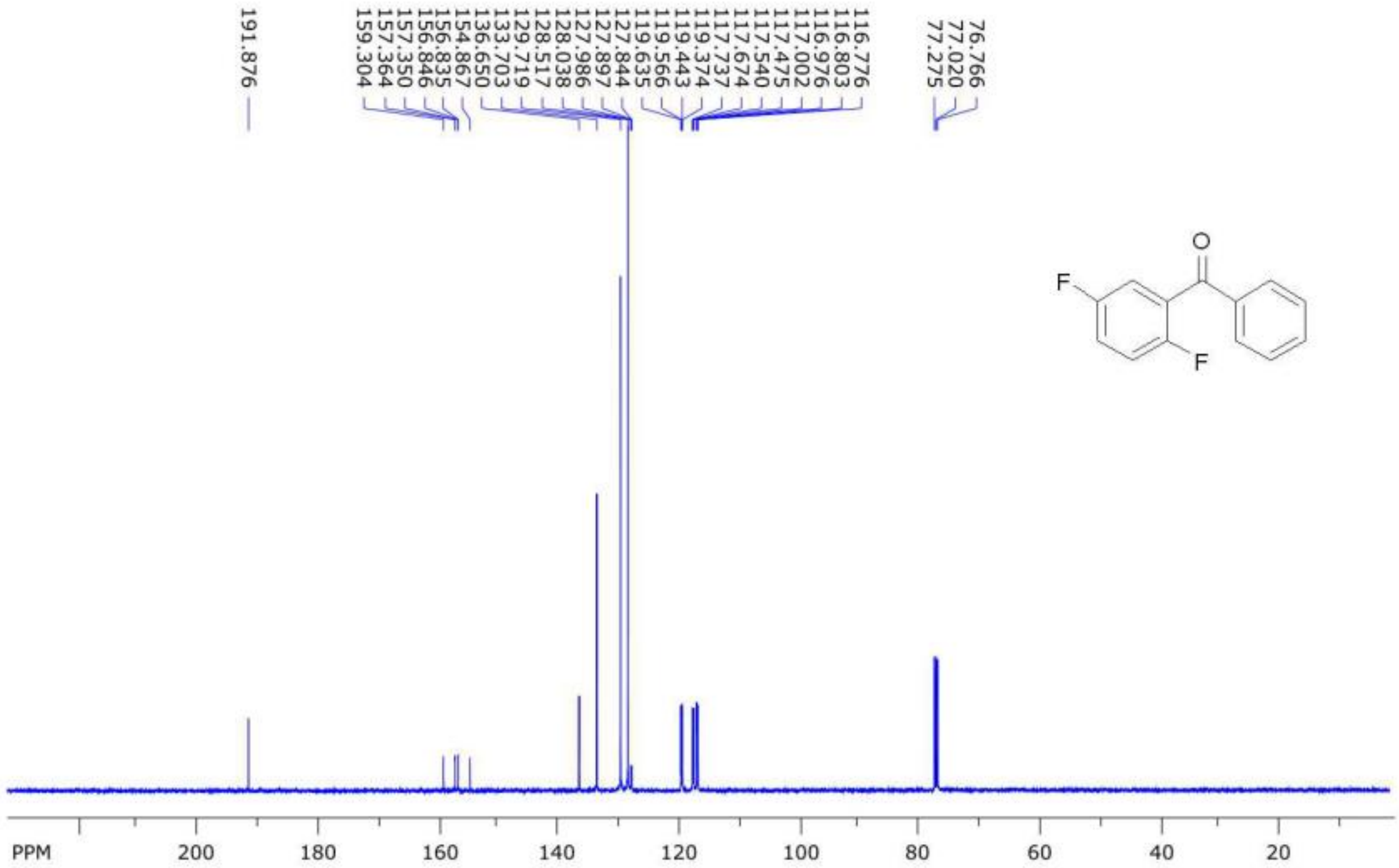

file: D:\NAPO\NMR\500-2\mkr10402\26\fid expt: <zgpg30>
transmitter freq.: 125.772879 MHz
time domain size: 65536 points
width: 36057.69 Hz = 286.6889 ppm = 0.550197 Hz/pt
number of scans: 512

freq. of 0 ppm: 125.757202 MHz
processed size: 32768 complex points
LB: 2.000 GF: 0.0000
Hz/cm: 1161.504 ppm/cm: 9.23494

Compound 6f

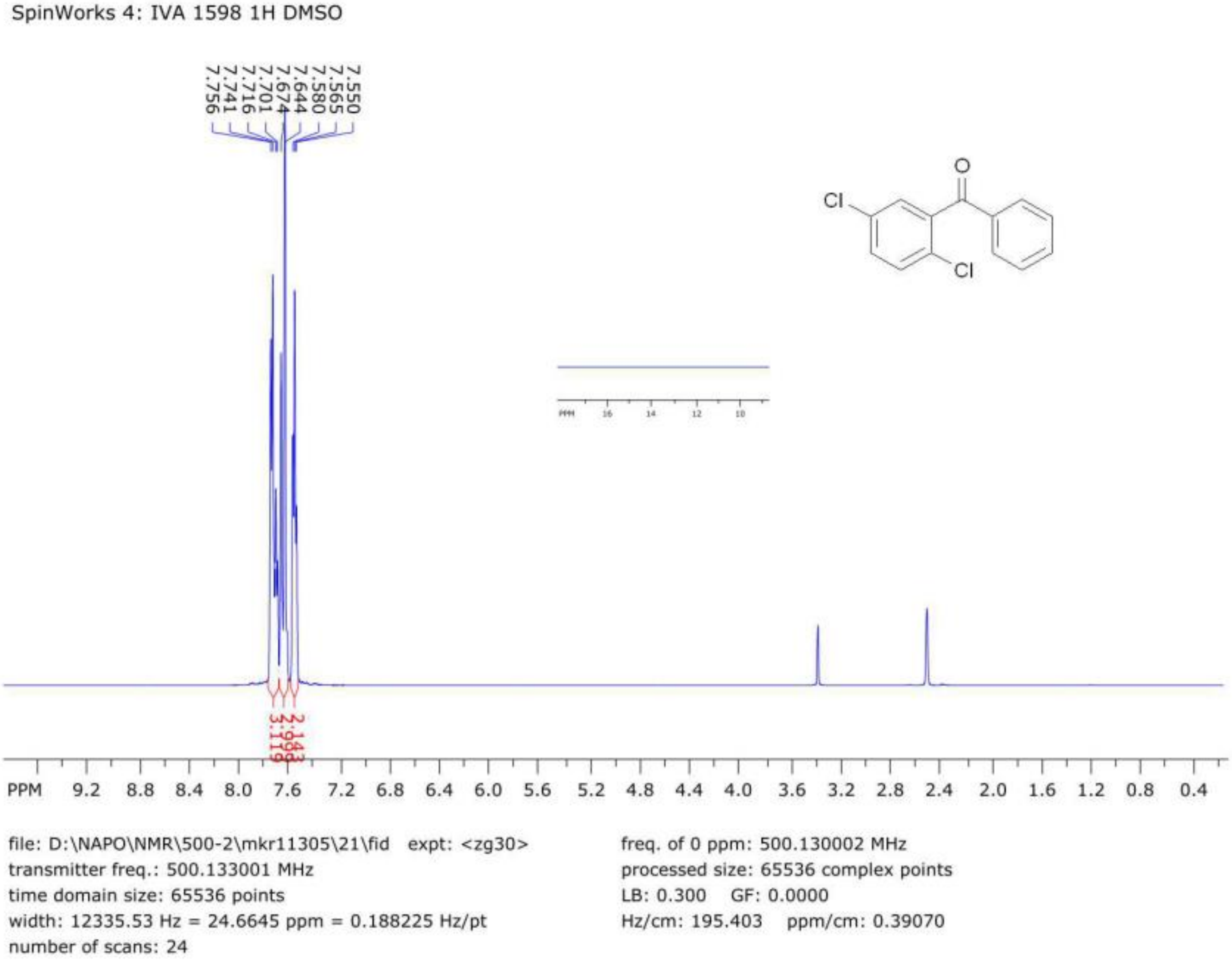
SpinWorks 4: IVA 1598 1H DMSO
7.756
7.741
7.716
7.701
7.674
7.644
7.580
7.565
7.550
Cl
O
Cl
2.143
3.119
PPM 9.2 8.8 8.4 8.0 7.6 7.2 6.8 6.4 6.0 5.6 5.2 4.8 4.4 4.0 3.6 3.2 2.8 2.4 2.0 1.6 1.2 0.8 0.4
file: D:\NAPO\NMR\500-2\mkr11305\21\fid expt: <zg30>
transmitter freq.: 500.133001 MHz
time domain size: 65536 points
width: 12335.53 Hz = 24.6645 ppm = 0.188225 Hz/pt
number of scans: 24
freq. of 0 ppm: 500.130002 MHz
processed size: 65536 complex points
LB: 0.300 GF: 0.0000
Hz/cm: 195.403 ppm/cm: 0.39070

Compound 6f

SpinWorks 4: IVA 1598 13C DMSO

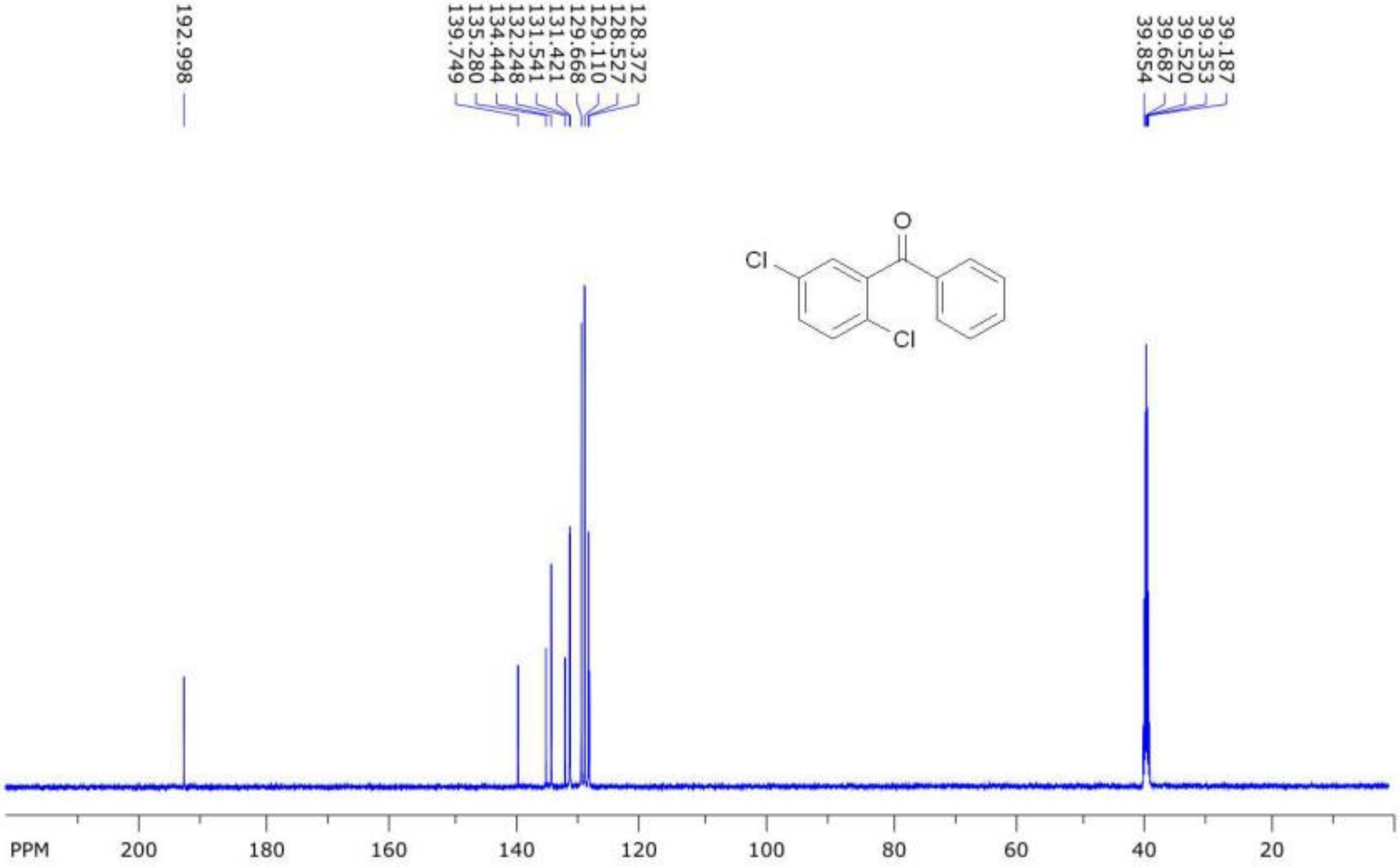

file: D:\NAPO\NMR\500-2\mkr11305\22\fid expt: <zgpg30>
transmitter freq.: 125.772879 MHz
time domain size: 65536 points
width: 36057.69 Hz = 286.6889 ppm = 0.550197 Hz/pt
number of scans: 512

freq. of 0 ppm: 125.757847 MHz
processed size: 32768 complex points
LB: 2.000 GF: 0.0000
Hz/cm: 1109.782 ppm/cm: 8.82370

Compound 6g

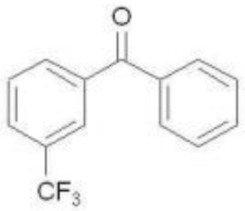

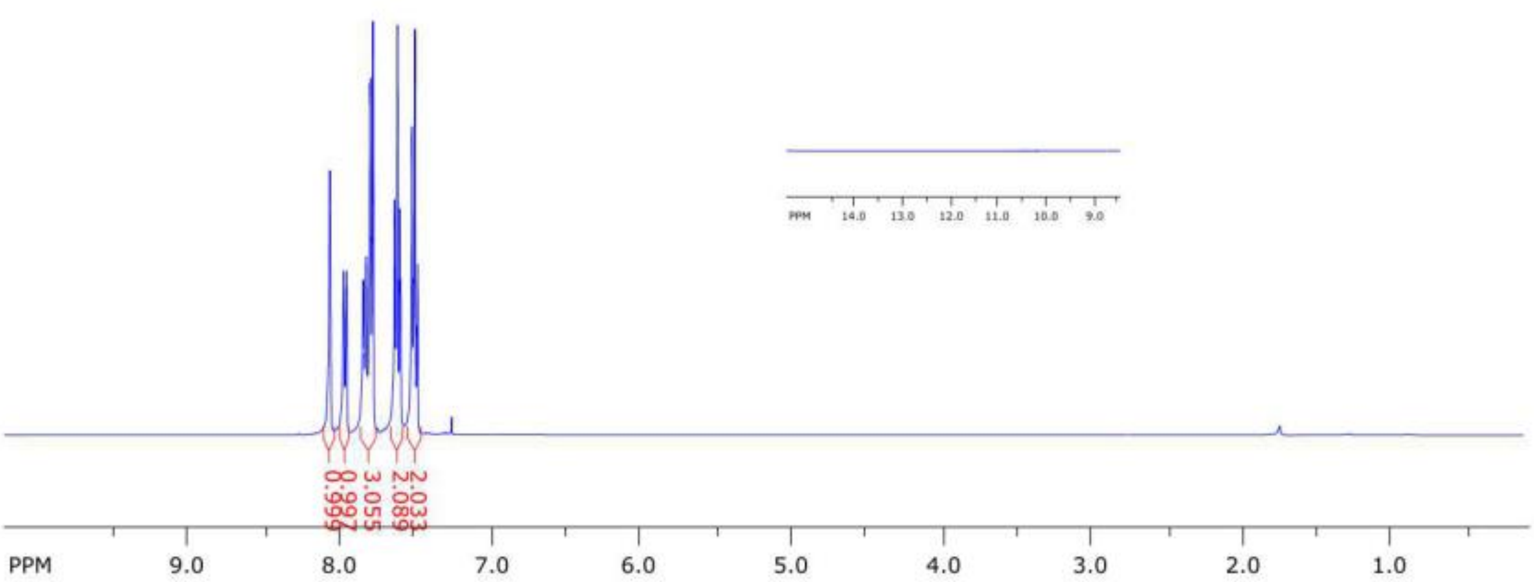

file: ...hael Slovakia\NMR\Ivab-3671\25\fid expt: <zg30>
transmitter freq.: 400.133001 MHz
time domain size: 65536 points
width: 6393.86 Hz = 15.9793 ppm = 0.097563 Hz/pt
number of scans: 64

freq. of 0 ppm: 400.130009 MHz
processed size: 65536 complex points
LB: 0.300 GF: 0.0000
Hz/cm: 162.393 ppm/cm: 0.40585

Compound 6g

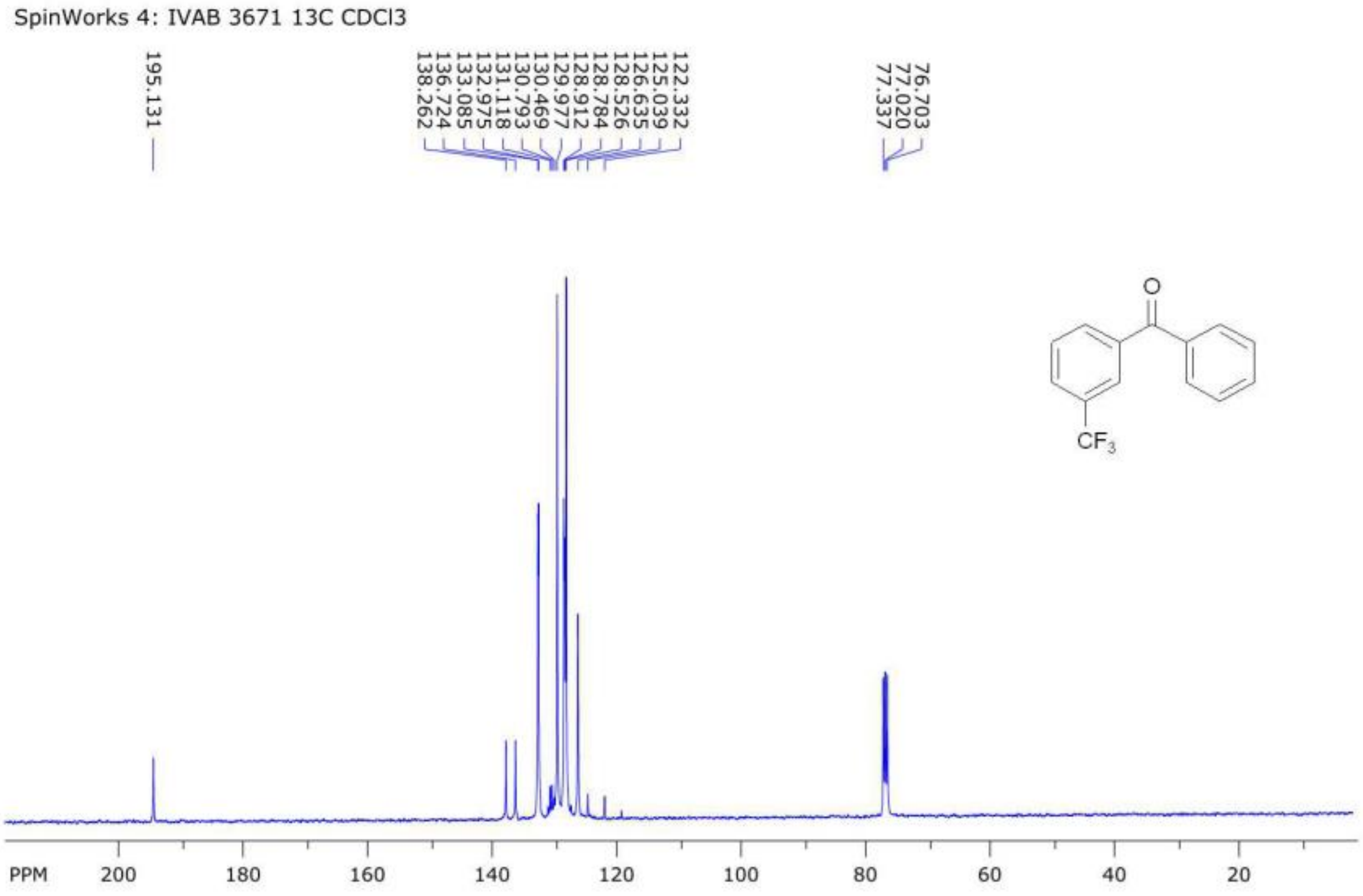

file: ...hael Slovakia\NMR\Ivab-3671\26\fid expt: <zgpg30>
transmitter freq.: 100.623836 MHz
time domain size: 65536 points
width: 24038.46 Hz = 238.8943 ppm = 0.366798 Hz/pt
number of scans: 1001

freq. of 0 ppm: 100.612772 MHz
processed size: 32768 complex points
LB: 10.000 GF: 0.0000
Hz/cm: 875.383 ppm/cm: 8.69956

Compound 6h

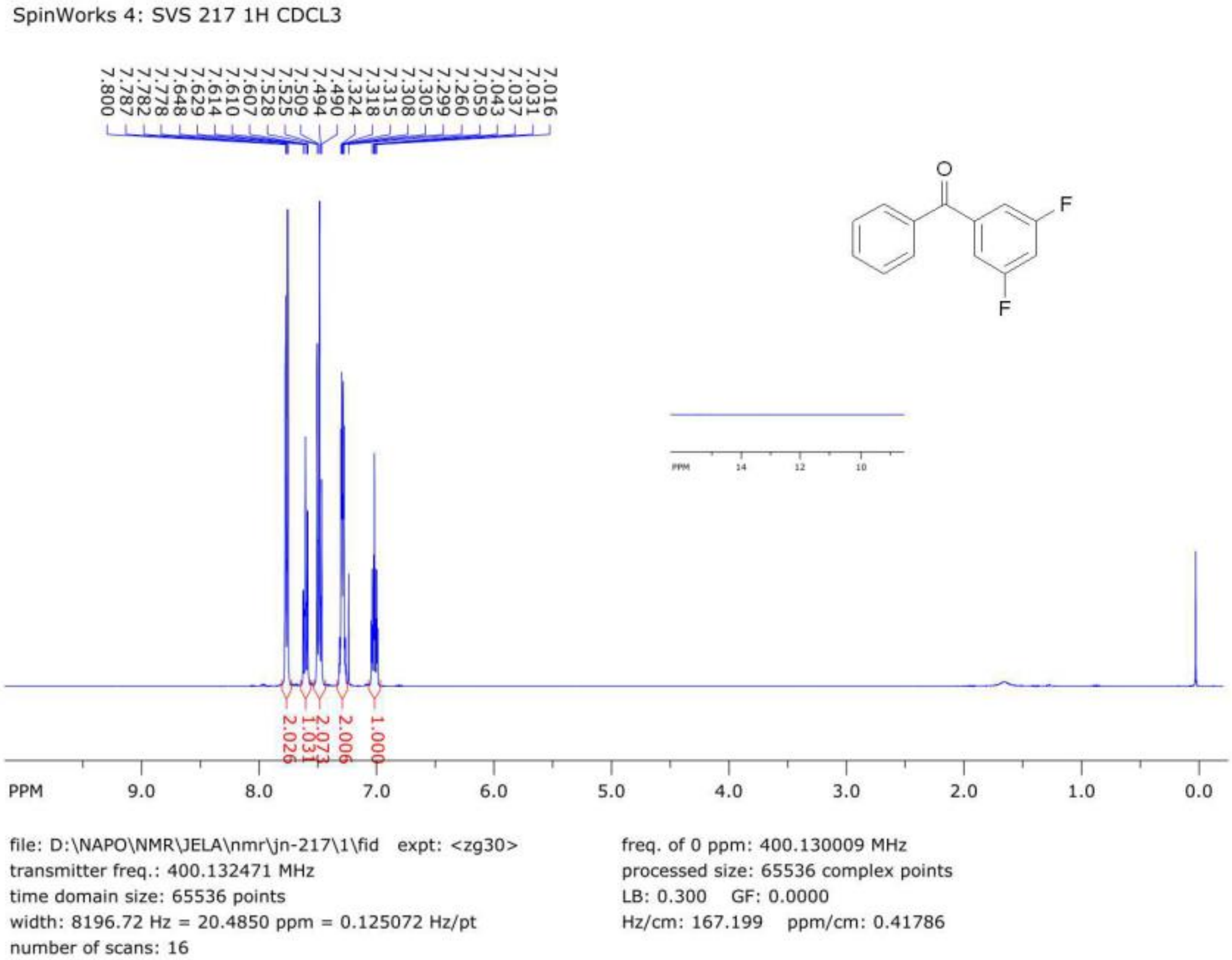
SpinWorks 4: SVS 217 1H CDCL3
7.800
7.787
7.782
7.778
7.648
7.629
7.614
7.610
7.607
7.528
7.525
7.509
7.494
7.490
7.324
7.318
7.315
7.308
7.305
7.299
7.260
7.059
7.043
7.037
7.031
7.016
2.026
1.031
2.073
2.006
1.000
PPM
9.0
8.0
7.0
6.0
5.0
4.0
3.0
2.0
1.0
0.0
O
F
F
file: D:\NAPO\NMR\JELA\nmr\jn-217\1\fid expt: <zg30>
transmitter freq.: 400.132471 MHz
time domain size: 65536 points
width: 8196.72 Hz = 20.4850 ppm = 0.125072 Hz/pt
number of scans: 16
freq. of 0 ppm: 400.130009 MHz
processed size: 65536 complex points
LB: 0.300 GF: 0.0000
Hz/cm: 167.199 ppm/cm: 0.41786

Compound 6h

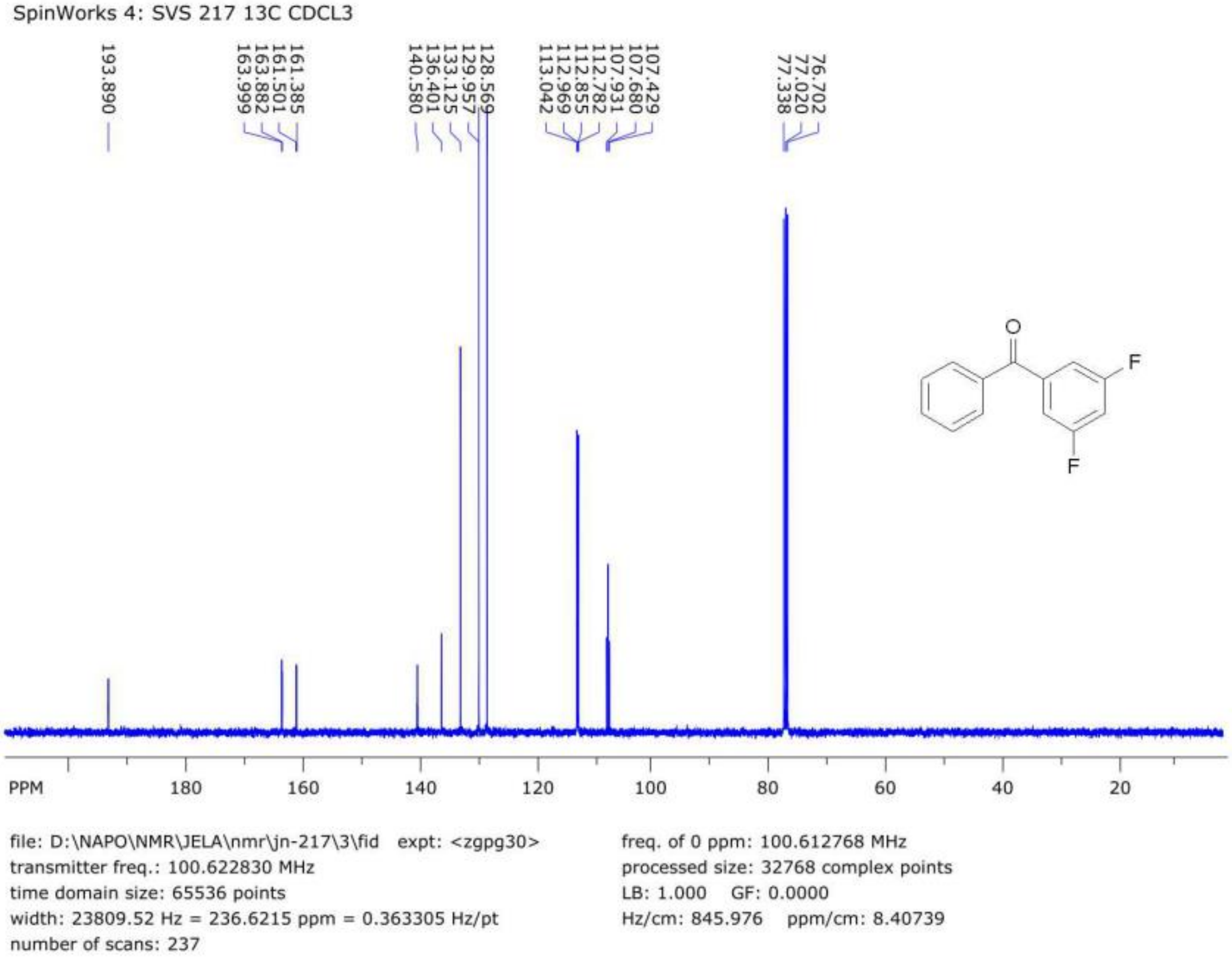
SpinWorks 4: SVS 217 13C CDCL3
193.890
161.385
161.501
163.882
163.999
128.569
129.957
133.125
136.401
140.580
107.429
107.680
107.931
112.782
112.855
112.969
113.042
76.702
77.020
77.338
O
F
F
PPM
180
160
140
120
100
80
60
40
20
file: D:\NAPO\NMR\JELA\nmr\jn-217\3\fid expt: <zgpg30>
transmitter freq.: 100.622830 MHz
time domain size: 65536 points
width: 23809.52 Hz = 236.6215 ppm = 0.363305 Hz/pt
number of scans: 237
freq. of 0 ppm: 100.612768 MHz
processed size: 32768 complex points
LB: 1.000 GF: 0.0000
Hz/cm: 845.976 ppm/cm: 8.40739

Compound 6i

SpinWorks 4: SVS 231 1H CDCL3

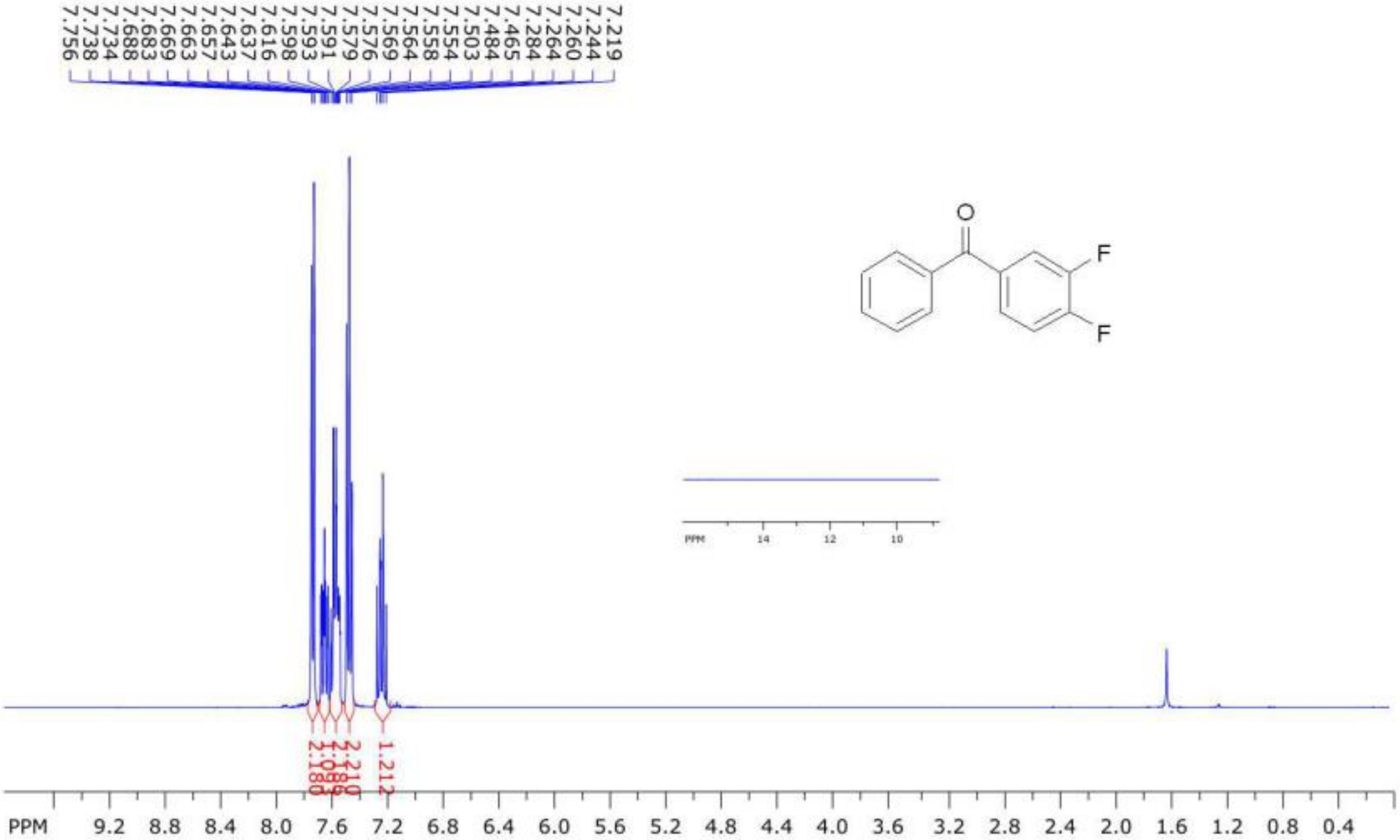

file: D:\NAPO\NMR\JELA\nmr\jn-231\1\fid expt: <zg30>
transmitter freq.: 400.132471 MHz
time domain size: 65536 points
width: 8196.72 Hz = 20.4850 ppm = 0.125072 Hz/pt
number of scans: 16

freq. of 0 ppm: 400.130016 MHz
processed size: 65536 complex points
LB: 0.300 GF: 0.0000
Hz/cm: 159.219 ppm/cm: 0.39792

Compound 6i

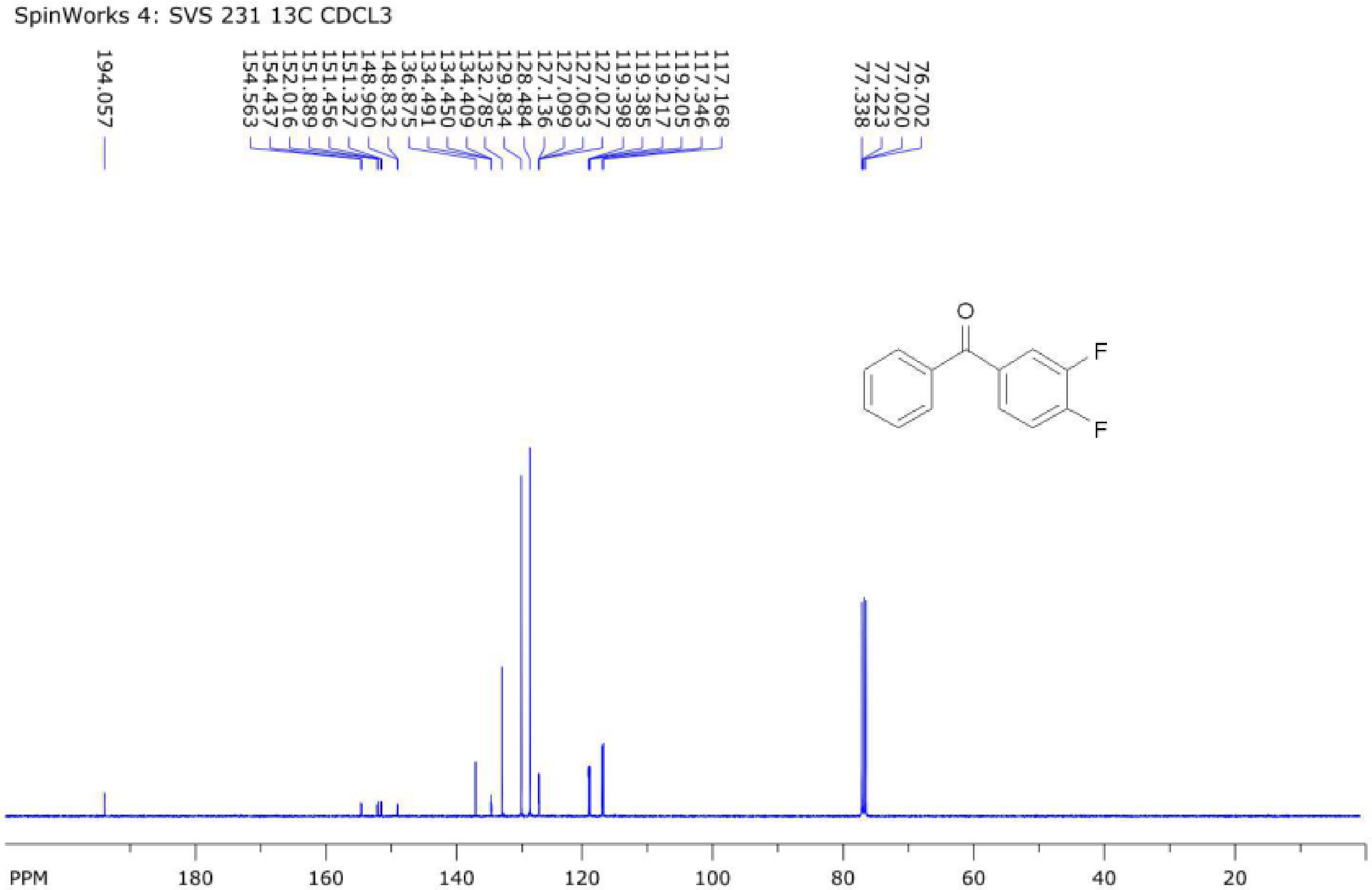

file: D:\NAPO\NMR\JELA\nmr\jn-231\2\fid expt: <zgpg30>
transmitter freq.: 100.622830 MHz
time domain size: 65536 points
width: 23809.52 Hz = 236.6215 ppm = 0.363305 Hz/pt
number of scans: 5000

freq. of 0 ppm: 100.612769 MHz
processed size: 32768 complex points
LB: 1.000 GF: 0.0000
Hz/cm: 840.708 ppm/cm: 8.35504

(D) DFT Study.

1. Computational methodology

DFT calculations are performed to gain insight into the mechanism of Ru-catalyzed activation of free phenols in a one-step Suzuki Miyaura cross-coupling under mechanochemical conditions. Herein, all the density functional theory (DFT) calculations are carried out using Gaussian 09 suite of programs.[1] The geometry optimizations are simulated at B3LYP functional of DFT without any constraints. B3LYP is computationally low cost functional, which is frequently employed for the quantum chemical calculations of reaction mechanisms, and geometric and electronic properties.[2–4] Additionally, in the domain of catalysis, B3LYP functional is an accurate and a reliable functional.[5,6] All studied structures are relaxed with Pople-typ basis set 6-31G(d,p) for lighter atoms (C, O, N, H and B) and Los Almos pseudopotential basis set LanL2DZ for heavy atoms (Ru and Zr).[7] Moreover, the structures of intermediates (minima) and transition states (saddle points) over potential energy surface are confirmed *via* frequency analysis i.e., the presence of one imaginary frequency in the Hessian matrix validates a saddle point (transition state), whereas absence of imaginary (negative) frequencies confirms the minima nature of found intermediate species. Additionally, transition state is also identified through animation of negative frequency, where the motion of the eigen vector corresponds to reaction axis.[8] All the reported energy values are in kcal/mol, while the discussed bond distances are reported in angstrom (Å), for all the studied geometries.

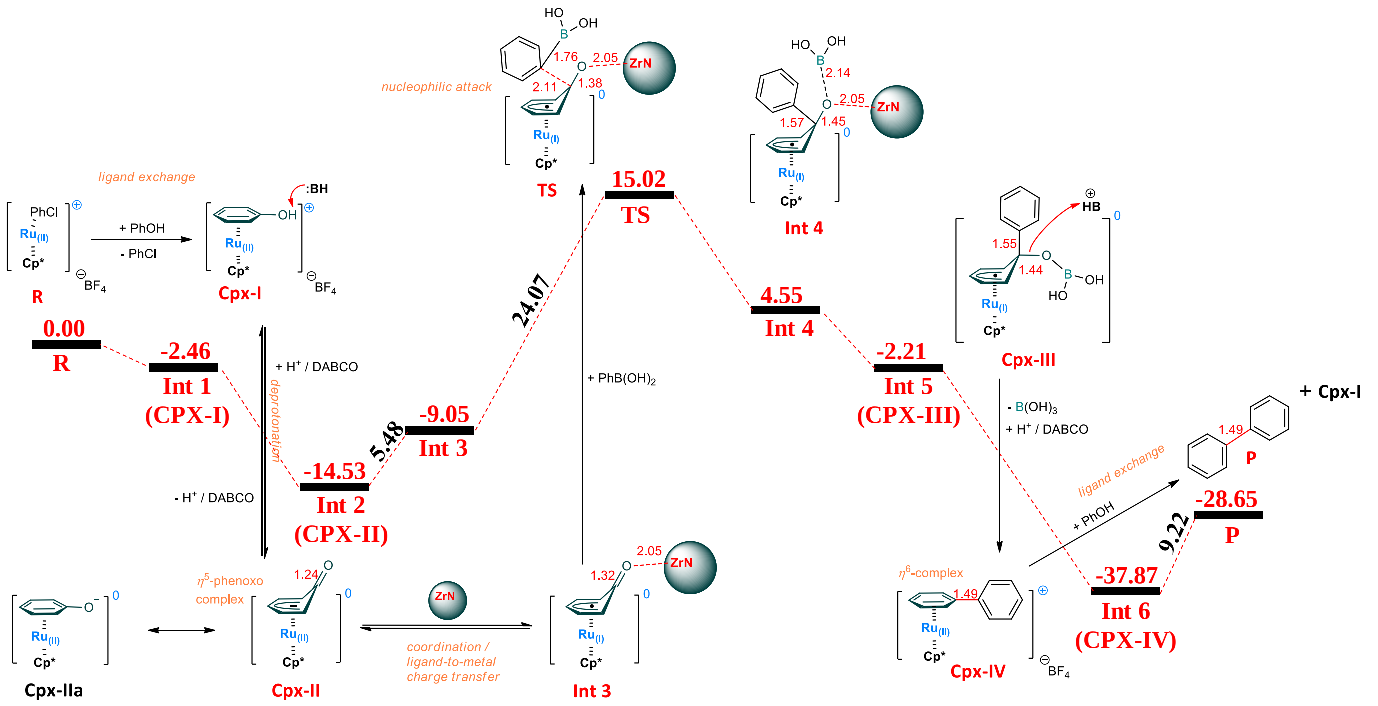

Figure S1. Energy profile for Ru-catalyzed formal activation of C—OH bond (free phenols) to biphenyl (Ar-Ar). All the reported energy values are presented in kcal/mol with reference initial reactant (R) at 0.00 kcal/mol at B3LYP functional with Pople style basis set 6-31G(d,p) for lighter atoms (C, O, N, H & B) and Los Almos pseudopotential basis set LanL2DZ for heavy atoms (Ru & Zr). Measured bond lengths are presented in Angstroms (Å).

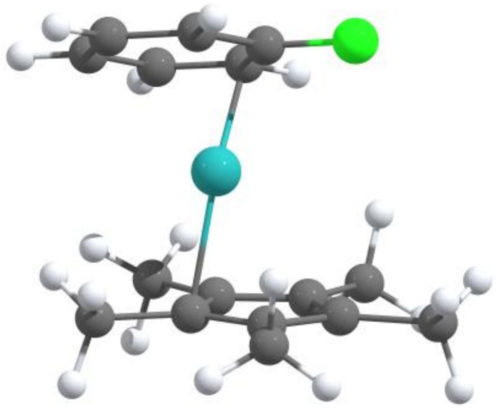	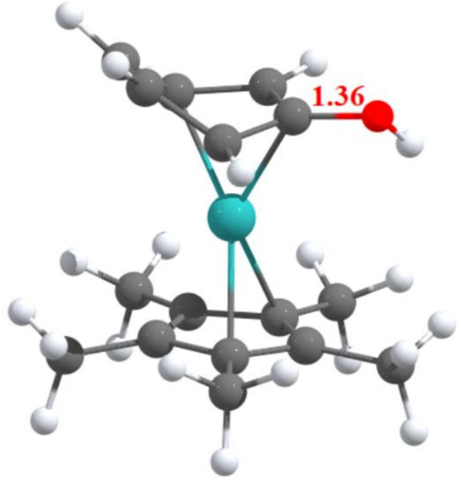
**CPX-0 (R)**	**CPX-I (Int 1)**
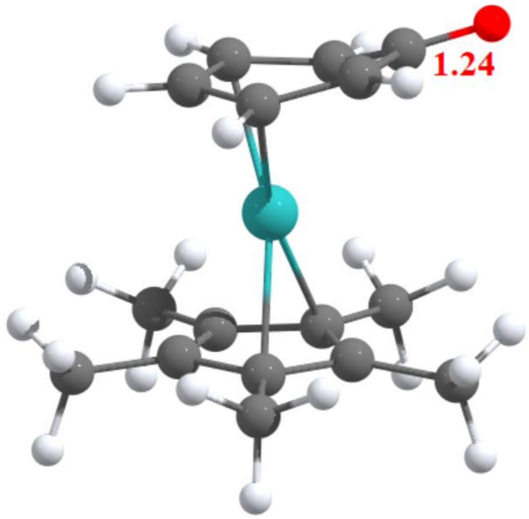	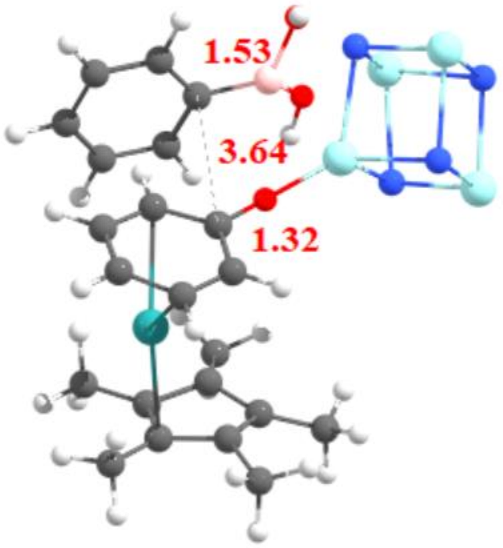
**CPX-II (Int 2)**	**Int 3**

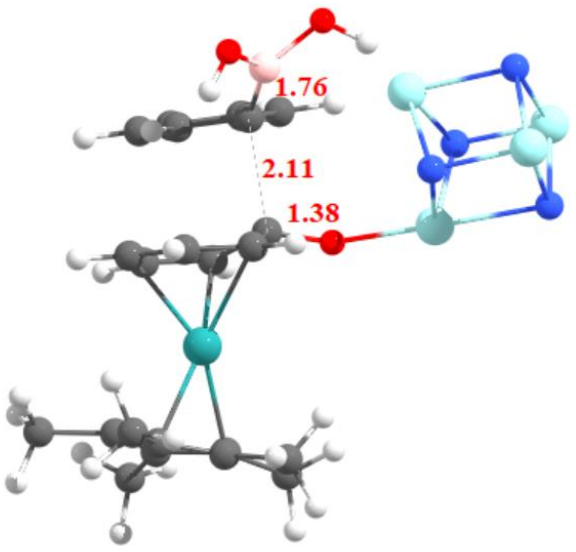

TS

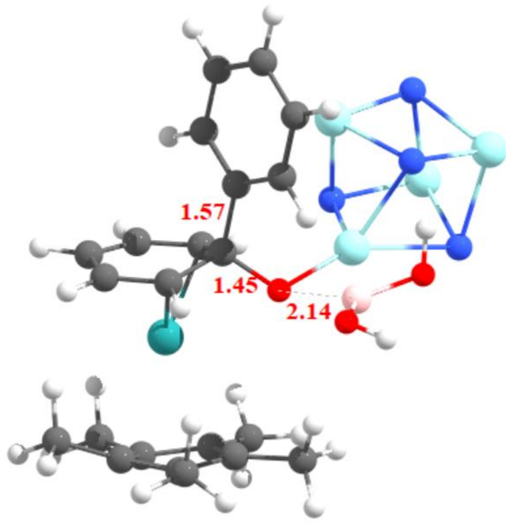

Int 4

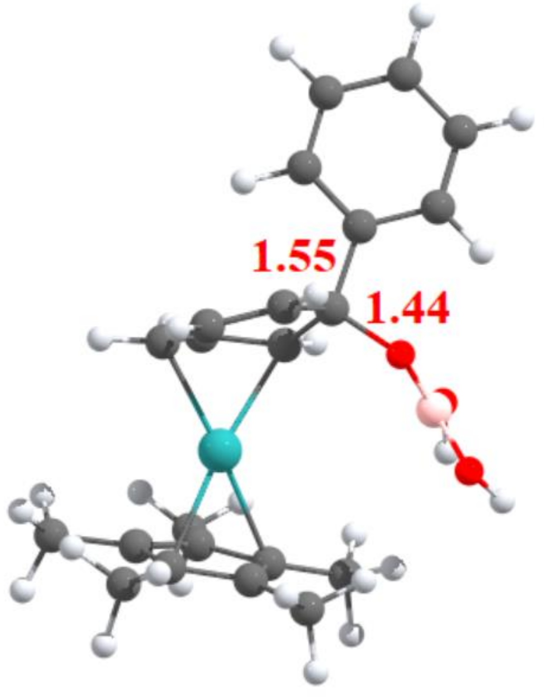

CPX-III (Int 5)

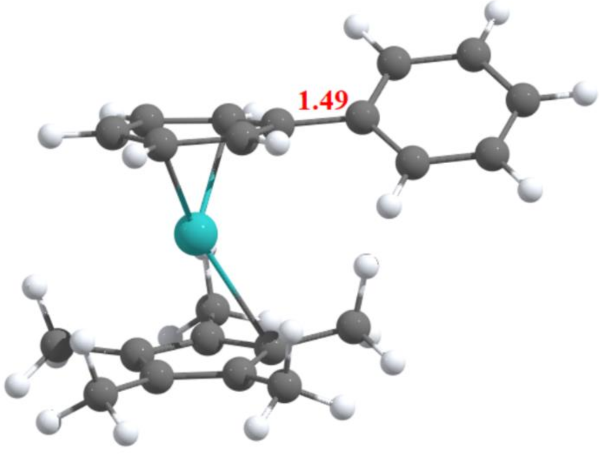

CPX-IV (Int 6)

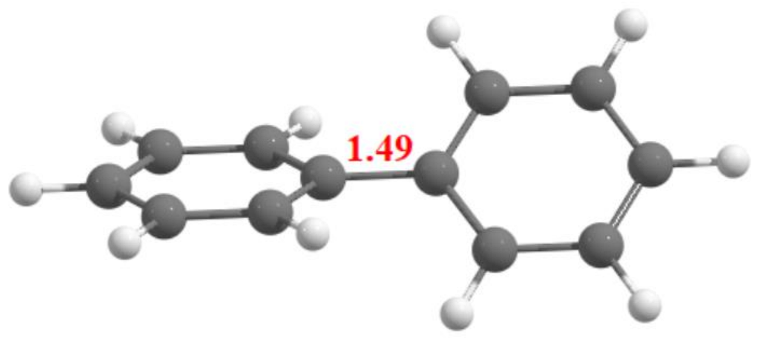

Final product

Figure S2: Optimized geometries of reactants, intermediates, transitions state and final product along with important bond lengths.

Table S4: Summary of electronic energy (SCF energy) and Gibbs free energy of optimized reacting species in Scheme 4.

Optimized Geometry	SCF energy (au)	Gibbs free energy (au)
**CPX-0 (R)**	-673.91	-673.70
**CPX-I (Int1)**	-595.49	-595.32
**CPX-II (Int 2)**	-594.87	-594.72
**Int 3**	-1605.42	-1605.31
**TS**	-1605.38	-1605.26
**Int 4**	-1605.40	-1605.29
**CPX-III (Int 5)**	-1003.16	-1002.89
**CPX-IV (Int 6)**	-751.33	-751.09
**Final product**	-463.32	-463.17

References

1. Frisch, M., et al., *01; Gaussian, Inc.* Wallingford, CT, 2009.
2. Bibi, S., et al., *Structure and electronic characterization of pristine and functionalized single wall carbon nanotube interacting with sulfide ion: A density functional theory approach.* Journal of Molecular Liquids, 2022. **366**: p. 120144.
3. Mkrtchyan, S., et al., *Mechanochemical arylative detrifluoromethylation of trifluoromethylarenes.* Organic & Biomolecular Chemistry, 2023. **21**(32): p. 6549-6555.
4. Mkrtchyan, S., et al., *Metal-Free Supramolecular Reduction of Nitro Compounds into the Cucurbit[7]uril Cavity: Testing the Enabling Technique in Aqueous Media.* ACS Sustainable Chemistry & Engineering, 2023. **11**(23): p. 8406-8412.

5. Sarfaraz, S., et al., *Transition Metal-Doped C20 Fullerene-Based Single-Atom Catalysts with High Catalytic Activity for Hydrogen Dissociation Reaction.* ACS Omega, 2023. **8**(15): p. 14077-14088.
6. Sarfaraz, S., et al., *Adsorption and dissociation of H2 molecule over first-row transition metal doped C24 nanocage as remarkable SACs: A comparative study.* International Journal of Hydrogen Energy, 2023.
7. Danish, M., et al., *Synthesis, single-crystal X-ray diffraction, and in vitro biological evaluation of sodium, cobalt, and tin complexes of o-nitro-/o-methoxyphenylacetic acid: experimental and theoretical investigation.* Monatshefte für Chemie-Chemical Monthly, 2020. **151**(11): p. 1727-1736.
8. Mukhtar, A., S. Sarfaraz, and K. Ayub, *Organic transformations in the confined space of porous organic cage CC2; catalysis or inhibition.* RSC advances, 2022. **12**(37): p. 24397-24411.

2. The inner-sphere mechanism analysis.

The inner-sphere mechanism adopted for the activation of C-OH bond (free phenols) to biphenyl (Ar-Ar) is presented in Figure S3. As per the energy profile, the inner-sphere mechanism for the conversion of free phenols to biphenyl undergoes three transition states. As presented in Figure S3, the activation barrier for transition states (TS1) and (TS3) is 29.4 and 23.3 kcal/mol, respectively. These activation barriers are somehow achievable under mechanochemical conditions. However, TS2 is located for the simultaneous addition of aryl and removal of OH group and is observed at the barrier height of 58.1 kcal/mol. This step is thermodynamically not feasible to achieve under mechanochemical conditions. Therefore, outer sphere mechanism of Ru-catalyzed activation of phenols in a one-step under mechanochemical conditions to biphenyl (Ar-Ar) is considered as more plausible reaction mechanism.

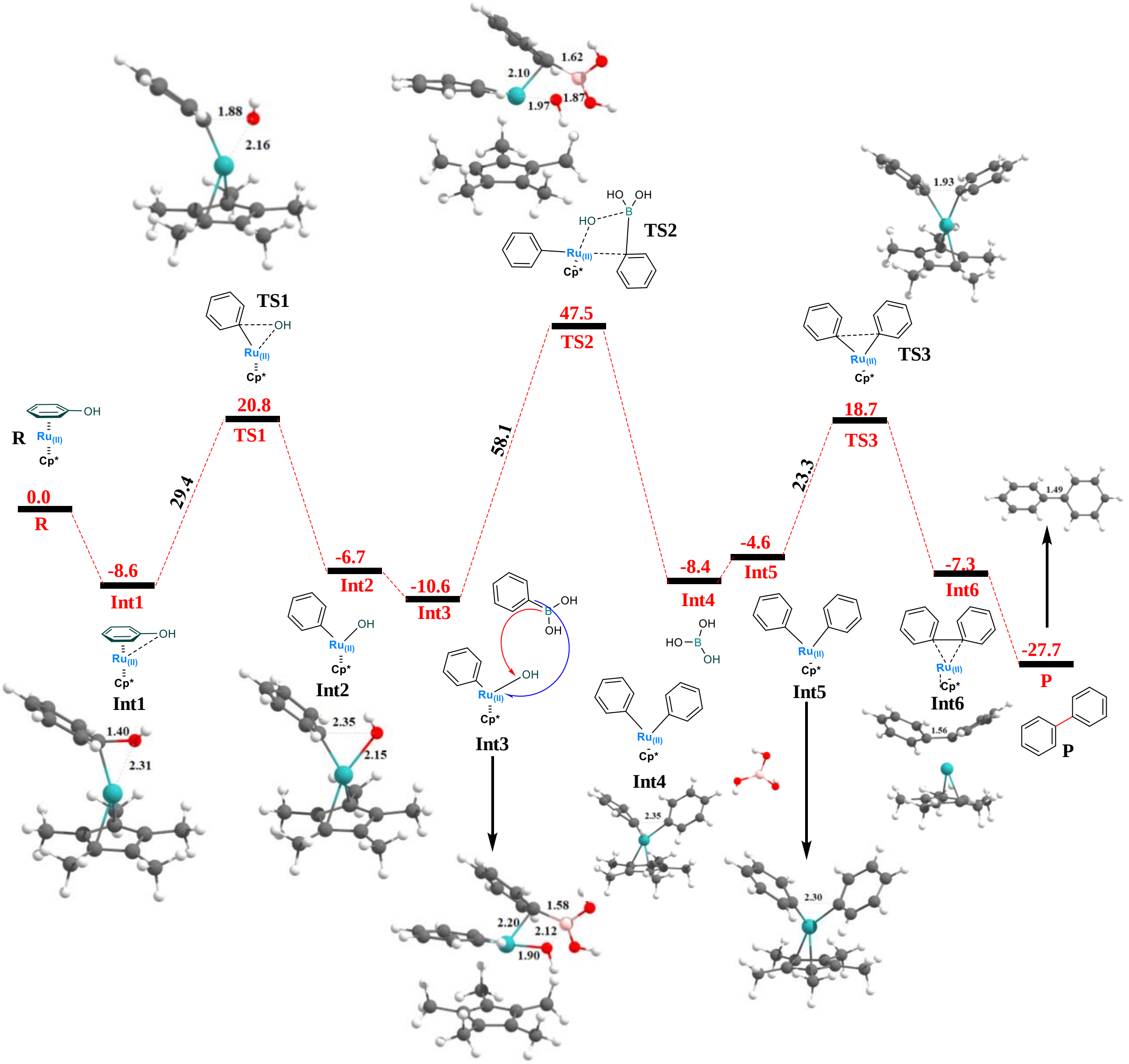

Figure S3. Energy profile for Ru-catalyzed formal activation of C—OH bond (free phenols) to biphenyl (Ar-Ar) through an inner-sphere mechanism. All the reported energy values are presented in kcal/mol with reference initial reactant (R) at 0.00 kcal/mol at B3LYP functional with Pople style basis set 6-31G(d,p) for lighter atoms (C, O, H, and B) and LanL2DZ for Ru.

Cartesian coordinates of reactant, product, transition state, and intermediates

CPX-0

C	1.33124100	-0.00013400	1.68262100
C	1.80466200	-1.15841300	0.96179400
C	2.61520500	-0.71632700	-0.14287200
C	1.80436000	1.15838700	0.96201600
C	2.61500500	0.71672000	-0.14275600
Ru	0.42784100	-0.00004600	-0.37457400
C	-0.67159700	-0.71191200	-2.07387000
C	-1.28950800	-1.31361500	-0.90233700
C	-2.53582600	-0.71088100	-0.38785100
C	-2.53579300	0.71084900	-0.38786400
C	-1.28945700	1.31350700	-0.90240600
C	-0.67158500	0.71171400	-2.07391800
H	-3.64471500	-2.48673600	0.10543600
H	-0.22596200	-1.29282800	-2.87925100
H	-1.20473400	-2.39678200	-0.80386200
C	-3.64426200	-1.39703900	0.10745700
C	-3.64421100	1.39706900	0.10739400
H	-1.20462700	2.39667400	-0.80398800
H	-0.22589800	1.29257100	-2.87931300
C	-4.74064500	0.69548000	0.64522600
C	-4.74066200	-0.69538600	0.64527100
H	-3.64462900	2.48676600	0.10531000
H	-5.58164800	1.24411900	1.06633600
H	-5.58167800	-1.24397400	1.06642100
C	1.61269300	-2.58647000	1.39048700
H	1.62327200	-3.26690200	0.53268200
H	2.40139800	-2.92011500	2.08370200

H	0.65142900	-2.71981500	1.89572200
C	0.62205900	-0.00037400	3.00660300
H	-0.02197700	0.87825000	3.11313400
H	-0.02196900	-0.87904100	3.11283000
H	1.32076100	-0.00051700	3.86156600
C	1.61206200	2.58632200	1.39096700
H	2.40073200	2.92003800	2.08418600
H	1.62242000	3.26690200	0.53327700
H	0.65079500	2.71935000	1.89628300
C	3.39334000	1.59812600	-1.07762500
H	3.48144700	1.14856300	-2.07290800
H	2.90934000	2.57179700	-1.20518900
H	4.41751300	1.79028100	-0.71688100
C	3.39365200	-1.59737400	-1.07799300
H	3.48151900	-1.14761600	-2.07320800
H	4.41792100	-1.78932900	-0.71741400
H	2.90988200	-2.57114500	-1.20565900

CPX-I

C	2.31970683	-1.42495886	-0.60338067
C	2.22089795	-0.10014136	-1.04831627
C	1.62070847	0.87723365	-0.25296632
C	1.11122404	0.53421623	1.01249267
C	1.21741016	-0.78214331	1.47054999
C	1.81956764	-1.76039322	0.66384799
H	2.76732985	-2.17623524	-1.22421458
H	2.60780632	0.16199072	-2.00991108
H	0.64392870	1.27778893	1.62526212
H	0.83343447	-1.03920886	2.44313604
H	1.90559672	-2.77163659	1.00765814

C	-2.58808347	0.77915990	0.66569399
C	-2.63111974	1.18650270	-0.67588268
C	-2.35292167	0.06290392	-1.48062529
C	-2.28293906	-0.58844801	0.68815201
C	-2.14490321	-1.02246124	-0.63617758
Ru	-0.19415400	-0.21760300	-0.05195700
O	1.52279270	2.22716606	-0.72095610
H	0.71764214	2.62489983	-0.37600192
C	-2.82831144	1.67843564	1.89584086
H	-1.89499998	2.10679619	2.21849821
H	-3.50161806	2.46280438	1.63064337
H	-3.24862607	1.09940303	2.69290293
C	-2.90906969	2.61709514	-1.17869565
H	-2.39043462	2.77239596	-2.09990701
H	-3.96380319	2.73408173	-1.32694333
H	-2.56658648	3.33991802	-0.45502670
C	-2.29657269	0.02348249	-3.01200660
H	-3.00947655	0.74724817	-3.39705047
H	-1.27522000	0.27284344	-3.33837151
H	-2.56006776	-0.98084753	-3.36312704
C	-1.81217672	-2.46218837	-1.07196887
H	-2.71812576	-2.99996999	-1.22361098
H	-1.25127900	-2.44765586	-1.98086373
H	-1.23005810	-2.93884294	-0.30983466
C	-2.12548118	-1.46879816	1.94546626
H	-3.07692872	-1.90537075	2.20944147
H	-1.41973174	-2.24932230	1.74763139
H	-1.77611380	-0.86431954	2.76563750

CPX-II

C	0.91647642	0.07254552	-0.31808111
O	0.76813608	1.41515522	-0.78750054
C	-0.40569310	-2.05966392	-0.78018726
C	0.19687948	-0.89403823	-1.27236671
C	0.33646971	-0.04881739	1.10024111
C	-0.27037074	-1.24064606	1.51911644
C	-0.58086718	-2.25563052	0.59944135
H	-0.74523835	-2.80754158	-1.46594306
H	0.16340830	-0.67983175	-2.32015056
H	0.40418925	0.77900031	1.77474025
H	-0.50860157	-1.37582381	2.55343062
H	-0.97343703	-3.18574731	0.95385017
C	-3.97803556	0.61328302	0.54763520
C	-3.39882135	1.61423080	-0.24388866
C	-3.23909556	1.10402861	-1.53918678
C	-4.17635937	-0.51565929	-0.25877216
C	-3.71961434	-0.21223265	-1.54846325
Ru	-1.76259800	-0.41681400	0.05664500
C	5.13033666	-0.97093498	-0.19649612
C	4.23714399	-1.55589895	0.71125918
C	2.87735197	-1.21909300	0.67219110
C	2.41079978	-0.29722876	-0.27458191
C	3.30403941	0.28815676	-1.18201145
C	4.66377823	-0.04886390	-1.14307692
H	6.16849253	-1.22829966	-0.16684339
H	4.59339236	-2.25968094	1.43412479
H	2.19539340	-1.66574860	1.36522237
H	2.94784523	0.99235463	-1.90466673
H	5.34571732	0.39785672	-1.83608176

B	1.50430016	2.37591501	0.16468493
O	1.63756332	2.02700673	1.65878335
H	2.44805956	2.40722995	2.00531381
O	2.10704703	3.68539803	-0.37719639
H	2.09864978	4.35176850	0.31379543
C	-3.00652363	3.02690218	0.22683986
H	-2.17162429	3.37440588	-0.34516641
H	-3.83408887	3.69113155	0.08972436
H	-2.74120871	2.99670611	1.26298016
C	-4.33400091	0.73305345	2.04123056
H	-4.25681416	-0.22842858	2.50457167
H	-3.65754023	1.41296106	2.51571021
H	-5.33490885	1.09781022	2.14119855
C	-4.78856456	-1.85453870	0.19293164
H	-5.84885155	-1.82764352	0.05147730
H	-4.37145904	-2.65122060	-0.38700943
H	-4.57079329	-2.01506304	1.22817955
C	-3.74174701	-1.15906522	-2.76278397
H	-2.82849455	-1.71540369	-2.79785408
H	-4.56734634	-1.83386398	-2.67384779
H	-3.84485910	-0.58577565	-3.66035805
C	-2.64053130	1.85757909	-2.74144806
H	-1.58346208	1.69508876	-2.77376932
H	-3.08469125	1.49683060	-3.64559519
H	-2.83755471	2.90442994	-2.64032560

Int3

N	-1.15051500	-1.21300700	0.83023300
Zr	-2.04077600	-3.01724500	0.29239100
N	-3.40286800	0.31047700	-0.00383800

Zr	-4.26617400	-1.32110100	-0.93643400
N	-3.91104300	-2.21252100	0.85635400
Zr	-2.96871600	-0.60787500	1.76128200
N	-2.34191400	-2.08083700	-1.46628100
Zr	-1.45495000	-0.26391300	-0.95218300
C	4.73335300	-0.75824800	1.64239500
C	3.43376500	-1.36243100	1.50178900
C	3.39192400	-2.03578400	0.22737800
C	5.49795300	-1.05768400	0.45512600
C	4.66919200	-1.84544100	-0.42108900
Ru	3.70234900	0.12437100	-0.12858000
C	6.94109000	-0.71144700	0.22951300
H	7.59152200	-1.50310700	0.62560200
H	7.16917600	-0.60210500	-0.83448600
H	7.21519700	0.22073100	0.73103100
C	5.24034300	-0.03153800	2.85415100
H	5.72042300	-0.73290500	3.54914300
H	5.98067800	0.72909200	2.58979500
H	4.42943200	0.46241100	3.39482500
C	5.09766500	-2.46297900	-1.72106800
H	5.85553000	-1.85787000	-2.22711400
H	5.52771600	-3.45841400	-1.54895100
H	4.25320100	-2.58488700	-2.40443100
C	2.24928200	-2.85980200	-0.28610300
H	2.23071400	-2.89090300	-1.37994300
H	2.32714300	-3.89517700	0.07626400
H	1.29051900	-2.45975300	0.05756500
C	2.33420800	-1.36322200	2.52001300
H	2.39964300	-2.29510600	3.10070100

H	2.39470400	-0.53112400	3.22599700
H	1.35202000	-1.32088300	2.04373900
C	1.66203500	0.93821300	-1.25905600
O	0.54447200	0.38723200	-1.59040800
C	3.99150700	1.59311100	-1.85104600
C	2.80319000	0.87205700	-2.13122700
C	1.88590100	1.53966400	0.03474600
C	3.07374200	2.26550700	0.31514900
C	4.13241900	2.30796900	-0.63056600
H	4.81124900	1.56671000	-2.56129800
H	2.70209100	0.31447500	-3.05633500
H	1.09724300	1.46801500	0.77650900
H	3.18993900	2.75277700	1.27805700
H	5.04916400	2.84242900	-0.40954900
C	-0.89429367	7.05195282	0.94544783
C	-1.64121467	5.87436982	0.86866183
C	-1.51879867	5.04061782	-0.24281917
C	-0.65674667	5.36032782	-1.31128217
C	0.08578933	6.55270982	-1.20420817
C	-0.02532467	7.39102982	-0.09545417
H	-0.98775967	7.70233382	1.81150483
H	-2.31712967	5.60266182	1.67581283
H	-2.10315467	4.12218282	-0.27245117
H	0.77616933	6.84438282	-1.99649717
H	0.56103133	8.30496382	-0.04145117
B	-0.49648667	4.40921782	-2.55412217
O	-1.06477767	3.15632082	-2.61686217
H	-1.78177767	2.89267982	-1.97517617
O	0.27782933	4.71549182	-3.64221617

H	0.68177133	5.58969882	-3.59014517

TS

N	-1.72784600	-0.73391000	0.54449300
Zr	-3.11017600	-1.55537600	-0.70485900
N	-3.35493900	1.62524700	0.84407500
Zr	-4.53657200	1.24005600	-0.79522600
N	-4.71359700	-0.64756300	0.09333300
Zr	-3.47742900	-0.19801900	1.85057200
N	-2.97814100	0.28068500	-1.66142500
Zr	-1.54040100	1.06945000	-0.23315800
C	2.72543700	-3.29075800	1.00722500
C	1.30597500	-3.13646200	0.74882200
C	1.12261000	-3.09189000	-0.68624300
C	3.40748400	-3.35730700	-0.26754500
C	2.42873200	-3.21428500	-1.29615900
Ru	2.43572900	-1.39916500	0.00332100
C	4.86538800	-3.63561400	-0.44797400
H	5.07070000	-4.71295000	-0.49537500
H	5.24917700	-3.17725700	-1.37182400
H	5.47376700	-3.22221500	0.36815700
C	3.35582300	-3.44549500	2.36037000
H	3.38078400	-4.49940700	2.66762400
H	4.37243800	-3.05501200	2.37026600
H	2.78993000	-2.90175300	3.12693100
C	2.69930900	-3.30240200	-2.78018300
H	3.70735200	-2.93551600	-3.02976700
H	2.62826300	-4.34963700	-3.12616000
H	1.98412100	-2.72367500	-3.36202200
C	-0.12669900	-2.99100200	-1.53295200

H	0.03380100	-2.14503400	-2.33654500
H	-0.34211400	-4.00332600	-2.06193200
H	-1.07186800	-2.64245400	-0.90955100
C	0.24899800	-3.12644900	1.80377800
H	-0.09340600	-4.15772100	2.01825600
H	0.63252100	-2.72177500	2.75554900
H	-0.60888800	-2.51447900	1.53034800
C	1.72746400	1.41355000	-0.02120700
O	0.41596200	1.78068000	-0.14343200
C	3.57712300	0.12361900	-1.11314800
C	2.25897400	0.64268200	-1.16302200
C	2.05158300	0.66257100	1.18764400
C	3.34787900	0.10263300	1.35050600
C	4.16739400	-0.06169400	0.17909100
H	4.10285400	-0.18841700	-2.01640200
H	1.68596800	0.60409900	-2.10144800
H	1.31620900	0.57548500	2.00430400
H	3.71302600	-0.25319100	2.31623500
H	5.20139100	-0.39835800	0.26067500
C	5.66779800	3.78716800	0.10709100
C	4.94050400	3.64993800	1.28048000
C	3.56270900	3.40294500	1.25518300
C	2.78264800	3.26727200	0.03691200
C	3.61844300	3.40640700	-1.14559700
C	4.97704800	3.65944300	-1.12525400
H	6.73657500	3.98550700	0.13598900
H	5.43393600	3.75307100	2.24309200
H	3.04389200	3.31154400	2.20596100
H	3.15013400	3.26147500	-2.12136400

H	5.52682900	3.74036600	-2.05451300
B	1.47890600	4.18849900	0.02259900
O	0.81851500	4.57168400	1.17411300
H	0.86743900	3.88045200	1.88533100
O	0.96951300	4.72296200	-1.15052200
H	1.38070700	4.32849500	-1.95556100

Int4

N	-2.10225700	-1.13952100	0.89997100
Zr	-2.94766200	-1.98370500	-0.77763200
N	-3.52396100	1.22261400	0.76550500
Zr	-4.21791500	0.80385300	-1.13507800
N	-4.69972100	-1.01422100	-0.22523400
Zr	-3.93970300	-0.53849300	1.61899500
N	-2.48499000	-0.26234100	-1.67607900
Zr	-1.52451200	0.67026200	0.04494300
C	3.46686100	-2.94745800	0.95507000
C	2.04433600	-2.84438200	0.77791400
C	1.78586400	-2.68346600	-0.62456600
C	4.08908400	-2.87904200	-0.33683900
C	3.05518800	-2.69473500	-1.31665200
Ru	3.04612500	-0.89262300	0.01308800
C	5.55240800	-3.06471100	-0.63140800
H	5.79750700	-4.12498100	-0.78246900
H	5.84532400	-2.52822200	-1.53967000
H	6.18052000	-2.69765200	0.18667200
C	4.16913000	-3.24319000	2.25118100
H	4.21302600	-4.32664000	2.43120700
H	5.19304600	-2.86933900	2.25123400
H	3.65090800	-2.79342200	3.10211700

C	3.22344000	-2.69186700	-2.81181700
H	4.19384900	-2.28861800	-3.11117200
H	3.14649200	-3.70685400	-3.22460800
H	2.45212300	-2.08173200	-3.29033900
C	0.42748700	-2.76597400	-1.26886000
H	0.33452000	-2.08440900	-2.13257600
H	0.23045100	-3.79899300	-1.61820600
H	-0.33302800	-2.48453100	-0.51900100
C	1.02147000	-3.09373100	1.84312500
H	0.93553700	-4.17729900	2.02321300
H	1.29899200	-2.63735200	2.80180700
H	0.03483800	-2.73971600	1.54952200
C	1.74525800	1.61382000	0.10463900
O	0.36122900	1.32720100	0.02765000
C	3.87482600	0.84659500	-1.07234700
C	2.45841700	0.97316700	-1.08132400
C	2.35211300	0.82937200	1.25569400
C	3.77182600	0.70615200	1.36637300
C	4.55795800	0.73089800	0.17944200
H	4.42930300	0.72749700	-1.99881600
H	1.97099700	1.06300800	-2.04981600
H	1.76536500	0.74144400	2.17074600
H	4.24230500	0.46540900	2.31580800
H	5.62358300	0.53316900	0.21021700
C	2.87090800	5.39285600	-1.48762900
C	3.61094600	4.88515000	-0.39478700
C	3.14069600	3.84902900	0.37456400
C	1.79449300	3.21833900	0.18369000
C	1.11022800	3.77734400	-1.02757000

C	1.62016800	4.79933400	-1.78892300
H	3.26004200	6.20428100	-2.08966200
H	4.58343400	5.31843700	-0.16788400
H	3.76076100	3.48226900	1.19223400
H	0.16015900	3.32236300	-1.30330300
H	1.06057600	5.15261800	-2.65047200
B	0.88508000	3.46467900	1.50304900
O	1.19795200	2.88099800	2.70760700
H	2.08158200	2.48413100	2.70854500
O	-0.28347800	4.18383600	1.47917300
H	-0.36323100	4.65748600	0.63601500

CPX-III

C	-1.95973200	-0.00646800	0.55874300
O	-1.84292900	1.39431500	0.97017600
C	-0.70706500	-1.80061600	1.78870500
C	-1.62664300	-0.79935100	1.80278700
C	-0.98563200	-0.36330100	-0.57421100
C	-0.44321700	-1.69693600	-0.66643900
C	-0.06199300	-2.29115900	0.58379500
H	-0.43971600	-2.27738600	2.73267900
H	-2.09374600	-0.46456200	2.72462700
H	-1.23787000	0.12295100	-1.51279200
H	-0.31258700	-2.21263200	-1.61719900
H	0.40583600	-3.27234000	0.60974300
C	2.77748300	0.00657300	-1.48327300
C	2.46231400	1.20710700	-0.74899300
C	2.81229500	1.00604400	0.62542000
C	3.28410500	-0.95481300	-0.54954500
C	3.28655900	-0.34509900	0.76206400

Ru	1.12438700	-0.50189300	0.05999200
C	-6.07301600	-0.78224000	-0.68651900
C	-5.14982300	-1.82394800	-0.53833500
C	-3.84519800	-1.55642900	-0.13152900
C	-3.41970300	-0.24271500	0.13527100
C	-4.35427700	0.79055400	-0.01341200
C	-5.66537100	0.52430400	-0.42043200
H	-7.09314600	-0.98977900	-1.00174600
H	-5.45102600	-2.85018400	-0.73897100
H	-3.13313600	-2.36727700	-0.01340200
H	-4.04891800	1.80779900	0.20001900
H	-6.37081300	1.34643000	-0.52615700
B	-1.26303700	2.39370100	0.26926400
O	-1.40692000	2.53748200	-1.10514900
H	-0.67803700	3.02466500	-1.50242700
O	-0.64703200	3.39291600	1.02342800
H	-0.45428100	4.16839400	0.48683000
C	2.02841000	2.50904500	-1.36185600
H	1.49793800	3.12647500	-0.62994700
H	2.88211800	3.10164400	-1.72880900
H	1.36454500	2.34226000	-2.21830400
C	2.68204600	-0.16260200	-2.97264000
H	2.52391100	-1.21141400	-3.24604800
H	1.84281600	0.40616000	-3.38765000
H	3.59239800	0.17442100	-3.49588000
C	3.83299200	-2.31203100	-0.88735200
H	4.91224000	-2.28547400	-1.11115100
H	3.69194300	-3.01545000	-0.06011100
H	3.33183400	-2.73959900	-1.76258900

C	3.87737200	-0.95017400	2.00558000
H	3.39743200	-0.55805400	2.90790200
H	3.74697900	-2.03702500	2.01938000
H	4.95823100	-0.75000100	2.09673000
C	2.74721700	2.04146400	1.71359500
H	1.85886500	2.67304500	1.61767500
H	2.69561700	1.57155900	2.70081600
H	3.63305100	2.69918800	1.71463100

CPX-IV

C	1.31386400	-1.35444000	-0.40998800
C	-0.71141000	-1.98496400	-1.62417700
C	0.53784500	-1.30572900	-1.65990500
C	1.27349300	-2.67009400	0.29321000
C	0.07527800	-3.27677800	0.34537800
C	-1.03761700	-2.55680800	-0.32139000
H	-1.36465500	-2.03143100	-2.49485300
H	0.90521300	-0.84226600	-2.57417100
H	2.19306300	-3.16501900	0.60802100
H	-0.05422600	-4.29049600	0.73024400
H	-2.04124800	-2.96645300	-0.20118500
C	-1.63363900	0.66741800	1.57135400
C	-0.63818000	1.53910800	1.00741100
C	-1.05484800	1.93103600	-0.31237800
C	-2.64457200	0.47146300	0.55374300
C	-2.29414600	1.26968500	-0.59503700
Ru	-0.74986100	-0.37633300	-0.21666200
C	4.78348800	1.18306300	0.12976400
C	4.50354000	0.14277300	1.02010500
C	3.38412000	-0.66827700	0.84545700

C	2.47994300	-0.48204800	-0.22962600
C	2.77649600	0.59299100	-1.10701900
C	3.89881500	1.39806500	-0.93345700
H	5.65782700	1.81499000	0.26510800
H	5.16525800	-0.04049900	1.86548500
H	3.18632000	-1.45738400	1.56401600
H	2.09685200	0.80443700	-1.92565000
H	4.08476000	2.20788000	-1.63749300
C	-1.68936800	0.17068800	2.98787200
H	-2.20485200	-0.79306100	3.05057200
H	-0.68497400	0.02193300	3.39668900
H	-2.21708500	0.86867600	3.66023700
C	-3.92779600	-0.28916500	0.73635100
H	-4.72421100	0.33682900	1.16966300
H	-4.30093900	-0.67817000	-0.21697900
H	-3.78583500	-1.14712500	1.40097100
C	-3.12918700	1.45527100	-1.82956100
H	-3.74048200	0.57058600	-2.03471400
H	-3.81635400	2.31291400	-1.74285800
H	-2.50474000	1.62895400	-2.71268100
C	-0.35905200	2.92598500	-1.19694100
H	-0.52569600	2.70608700	-2.25751600
H	-0.71071800	3.95633900	-1.02187700
H	0.72189800	2.91901300	-1.02980300
C	0.58193700	2.05169100	1.71902600
H	1.42925300	2.16144200	1.03614500
H	0.39760700	3.03145100	2.18640900
H	0.89781900	1.36343100	2.50821100

Product

C	-0.74285200	-0.00018200	-0.00008600
C	-2.85923600	1.13933000	-0.39385800
C	-1.46541600	1.13859400	-0.39453200
C	-1.46558500	-1.13868900	0.39464100
C	-2.85945800	-1.13916800	0.39387000
C	-3.56302500	0.00001500	-0.00022200
H	-3.39642400	2.02886800	-0.71043800
H	-0.92803600	2.02106200	-0.72873400
H	-0.92848400	-2.02118300	0.72919500
H	-3.39666300	-2.02846600	0.71109200
H	-4.64901900	0.00026700	-0.00004700
C	3.56302900	0.00008600	0.00000000
C	2.85948700	-1.13917100	-0.39388900
C	1.46556600	-1.13870400	-0.39457400
C	0.74286500	-0.00011900	-0.00010700
C	1.46542100	1.13860000	0.39459000
C	2.85920800	1.13930600	0.39393700
H	4.64902200	0.00035100	0.00010200
H	3.39665600	-2.02865800	-0.71063100
H	0.92850400	-2.02132100	-0.72882200
H	0.92797100	2.02096100	0.72894700
H	3.39645200	2.02873800	0.71072400
